# Clonal haematopoiesis of indeterminate potential and mortality in coronary artery disease

**DOI:** 10.1093/eurheartj/ehaf602

**Published:** 2025-09-03

**Authors:** Moritz von Scheidt, Shaunak S Adkar, Johannes Krefting, Gregor Hoermann, Manja Meggendorfer, Sabine Bauer, Shaneice Mitchell, Irina Pugach, Christian Friess, Angela Ma, Ke Hao, Sophia Steigerwald, Maria Wahle, Thorsten Kessler, Marius Schwab, Felix Voll, Michal Mokry, Charalampos Sofokleous, Kaylin C A Palm, Dario Bongiovanni, Julia Fleig, Lilith Oldenbuettel, Zhifen Chen, Judith S Hecker, Florian Bassermann, Lars Maegdefessel, Matthias Graw, Arno Ruusalepp, Ingo Hilgendorf, Florian Leuschner, Hendrik B Sager, J Brett Heimlich, Wolfgang Koenig, Sebastian Cremer, David M Leistner, Wesley T Abplanalp, Stefanie Dimmeler, Andreas M Zeiher, Sander W van der Laan, Gerard Pasterkamp, Christian Braun, Siddhartha Jaiswal, Jason C Kovacic, Wolfgang Kern, Claudia Haferlach, Matthias Mann, Salvatore Cassese, Adnan Kastrati, Torsten Haferlach, Nicholas J Leeper, Johan L M Björkegren, Heribert Schunkert

**Affiliations:** Department of Cardiology, TUM Klinikum Deutsches Herzzentrum, Technical University Munich, Lazarettstr. 36, Munich D-80636, Germany; Deutsches Zentrum für Herz- und Kreislaufforschung (DZHK), Partner Site Munich Heart Alliance, Munich, Germany; Division of Vascular Surgery, Department of Surgery, Stanford University School of Medicine, Stanford, CA, USA; Department of Cardiology, TUM Klinikum Deutsches Herzzentrum, Technical University Munich, Lazarettstr. 36, Munich D-80636, Germany; Deutsches Zentrum für Herz- und Kreislaufforschung (DZHK), Partner Site Munich Heart Alliance, Munich, Germany; MLL Munich Leukemia Laboratory, Munich, Germany; MLL Munich Leukemia Laboratory, Munich, Germany; Department of Cardiology, TUM Klinikum Deutsches Herzzentrum, Technical University Munich, Lazarettstr. 36, Munich D-80636, Germany; Deutsches Zentrum für Herz- und Kreislaufforschung (DZHK), Partner Site Munich Heart Alliance, Munich, Germany; Department of Pathology, Stanford University, Stanford, CA, USA; Department of Cardiology, TUM Klinikum Deutsches Herzzentrum, Technical University Munich, Lazarettstr. 36, Munich D-80636, Germany; Deutsches Zentrum für Herz- und Kreislaufforschung (DZHK), Partner Site Munich Heart Alliance, Munich, Germany; Department of Cardiology, TUM Klinikum Deutsches Herzzentrum, Technical University Munich, Lazarettstr. 36, Munich D-80636, Germany; Deutsches Zentrum für Herz- und Kreislaufforschung (DZHK), Partner Site Munich Heart Alliance, Munich, Germany; Department of Genetics and Genomic Sciences, Institute of Genomics and Multiscale Biology, Icahn School of Medicine at Mount Sinai, New York, NY, USA; Department of Genetics and Genomic Sciences, Institute of Genomics and Multiscale Biology, Icahn School of Medicine at Mount Sinai, New York, NY, USA; Department of Proteomics and Signal Transduction, Max Planck Institute of Biochemistry, Martinsried, Germany; Department of Proteomics and Signal Transduction, Max Planck Institute of Biochemistry, Martinsried, Germany; Department of Cardiology, TUM Klinikum Deutsches Herzzentrum, Technical University Munich, Lazarettstr. 36, Munich D-80636, Germany; Deutsches Zentrum für Herz- und Kreislaufforschung (DZHK), Partner Site Munich Heart Alliance, Munich, Germany; Department of Cardiology, TUM Klinikum Deutsches Herzzentrum, Technical University Munich, Lazarettstr. 36, Munich D-80636, Germany; Deutsches Zentrum für Herz- und Kreislaufforschung (DZHK), Partner Site Munich Heart Alliance, Munich, Germany; Department of Cardiology, TUM Klinikum Deutsches Herzzentrum, Technical University Munich, Lazarettstr. 36, Munich D-80636, Germany; Laboratory of Experimental Cardiology, Department of Cardiology, University Medical Center Utrecht, University Utrecht, Utrecht, Netherlands; Central Diagnostics Laboratory, University Medical Center Utrecht, Utrecht, Netherlands; Central Diagnostics Laboratory, University Medical Center Utrecht, Utrecht, Netherlands; Central Diagnostics Laboratory, University Medical Center Utrecht, Utrecht, Netherlands; Department of Internal Medicine I, Cardiology, University Hospital Augsburg, University of Augsburg, Augsburg, Germany; Department of Cardiovascular Medicine, Humanitas Clinical and Research Center IRCCS, Humanitas University, Rozzano, Milan, Italy; Department of Cardiology, TUM Klinikum Deutsches Herzzentrum, Technical University Munich, Lazarettstr. 36, Munich D-80636, Germany; Department of Cardiology, TUM Klinikum Deutsches Herzzentrum, Technical University Munich, Lazarettstr. 36, Munich D-80636, Germany; Department of Cardiology, TUM Klinikum Deutsches Herzzentrum, Technical University Munich, Lazarettstr. 36, Munich D-80636, Germany; Deutsches Zentrum für Herz- und Kreislaufforschung (DZHK), Partner Site Munich Heart Alliance, Munich, Germany; Department of Medicine III, TUM Klinikum Rechts der Isar, Munich, Germany and TranslaTUM, Center for Translational Cancer Research, Technical University of Munich (TUM), Munich, Germany; Department of Medicine III, TUM Klinikum Rechts der Isar, Munich, Germany and TranslaTUM, Center for Translational Cancer Research, Technical University of Munich (TUM), Munich, Germany; Deutsches Zentrum für Herz- und Kreislaufforschung (DZHK), Partner Site Munich Heart Alliance, Munich, Germany; Institute of Molecular Vascular Medicine, TUM Klinikum Rechts der Isar, Technical University Munich, Munich, Germany; Institute of Legal Medicine, Faculty of Medicine, LMU Munich, Munich, Germany; Department of Cardiac Surgery, The Heart Clinic, Tartu University Hospital, Tartu, Estonia; Clinical Gene Networks AB, Stockholm, Sweden; Institute of Clinical Medicine, Faculty of Medicine, Tartu University, Tartu, Estonia; Department of Cardiology and Angiology, University Heart Center Freiburg-Bad Krozingen, University of Freiburg, Freiburg, Germany; Department of Medicine III, University of Heidelberg, German Centre for Cardiovascular Research (DZHK), Heidelberg, Germany; Department of Cardiology, TUM Klinikum Deutsches Herzzentrum, Technical University Munich, Lazarettstr. 36, Munich D-80636, Germany; Deutsches Zentrum für Herz- und Kreislaufforschung (DZHK), Partner Site Munich Heart Alliance, Munich, Germany; Department of Medicine, Vanderbilt University Medical Center, Nashville, TN, USA; Department of Cardiology, TUM Klinikum Deutsches Herzzentrum, Technical University Munich, Lazarettstr. 36, Munich D-80636, Germany; Deutsches Zentrum für Herz- und Kreislaufforschung (DZHK), Partner Site Munich Heart Alliance, Munich, Germany; Department of Cardiology, Goethe University Frankfurt, Frankfurt am Main, Germany; Department of Cardiology, Goethe University Frankfurt, Frankfurt am Main, Germany; Institute for Cardiovascular Regeneration, Goethe University Frankfurt am Main, Frankfurt am Main, Germany; Institute for Cardiovascular Regeneration, Goethe University Frankfurt am Main, Frankfurt am Main, Germany; Department of Cardiology, Goethe University Frankfurt, Frankfurt am Main, Germany; Institute for Cardiovascular Regeneration, Goethe University Frankfurt am Main, Frankfurt am Main, Germany; Laboratory of Experimental Cardiology, Department of Cardiology, University Medical Center Utrecht, University Utrecht, Utrecht, Netherlands; Central Diagnostics Laboratory, University Medical Center Utrecht, Utrecht, Netherlands; Laboratory of Experimental Cardiology, Department of Cardiology, University Medical Center Utrecht, University Utrecht, Utrecht, Netherlands; Central Diagnostics Laboratory, University Medical Center Utrecht, Utrecht, Netherlands; Institute of Legal Medicine, Faculty of Medicine, LMU Munich, Munich, Germany; Department of Pathology, Stanford University, Stanford, CA, USA; Victor Chang Cardiac Research Institute, Darlinghurst, Australia; St. Vincent's Clinical School, University of New South Wales, Sydney, Australia; Cardiovascular Research Institute, Icahn School of Medicine at Mount Sinai, New York, NY, USA; MLL Munich Leukemia Laboratory, Munich, Germany; MLL Munich Leukemia Laboratory, Munich, Germany; Department of Proteomics and Signal Transduction, Max Planck Institute of Biochemistry, Martinsried, Germany; Department of Cardiology, TUM Klinikum Deutsches Herzzentrum, Technical University Munich, Lazarettstr. 36, Munich D-80636, Germany; Department of Cardiology, TUM Klinikum Deutsches Herzzentrum, Technical University Munich, Lazarettstr. 36, Munich D-80636, Germany; Deutsches Zentrum für Herz- und Kreislaufforschung (DZHK), Partner Site Munich Heart Alliance, Munich, Germany; MLL Munich Leukemia Laboratory, Munich, Germany; Division of Vascular Surgery, Department of Surgery, Stanford University School of Medicine, Stanford, CA, USA; Stanford Cardiovascular Institute, Stanford University, Stanford, CA, USA; Department of Genetics and Genomic Sciences, Institute of Genomics and Multiscale Biology, Icahn School of Medicine at Mount Sinai, New York, NY, USA; Clinical Gene Networks AB, Stockholm, Sweden; Department of Medicine, Huddinge, Karolinska Institutet, Karolinska Universitetssjukhuset, Stockholm, Sweden; Department of Cardiology, TUM Klinikum Deutsches Herzzentrum, Technical University Munich, Lazarettstr. 36, Munich D-80636, Germany; Deutsches Zentrum für Herz- und Kreislaufforschung (DZHK), Partner Site Munich Heart Alliance, Munich, Germany

**Keywords:** Aging, Atherosclerotic cardiovascular disease, Clonal haematopoiesis of indeterminate potential, Cardiovascular disease, Coronary artery disease, Inflammation, Macrophages, Mortality, LDLR, TET2

## Abstract

**Background and Aims:**

Clonal haematopoiesis of indeterminate potential (CHIP) has been associated with cardiovascular risk, but its prognostic relevance and mechanistic role in coronary artery disease (CAD) remains incompletely understood. This study investigated the association between CHIP and all-cause mortality in CAD and explored the cellular and molecular mechanisms, focusing on *TET2* mutations.

**Methods:**

Targeted deep sequencing of 13 CHIP driver genes in 8612 patients with angiographically confirmed CAD was performed. Clonal haematopoiesis of indeterminate potential carriers (variant allele frequency ≥2%) were propensity-score matched 1:1 to non-carriers. Mortality was assessed over 3 years. Mechanistic insights were derived from post-mortem high-sensitivity plaque proteomics (MISSION), RNA sequencing from carotid plaques (Athero-Express), monocyte-derived macrophage transcriptomes (STARNET), and CRISPR/Cas9-generated *TET2*^+/−^ macrophages *in vitro*.

**Results:**

Clonal haematopoiesis of indeterminate potential was associated with increased 3-year mortality (hazard ratio 1.39, 95% confidence interval 1.16–1.65, *P* < .001) in 2389 matched pairs. Mutations in *TET2*, *ASXL1*, *DNMT3A*, *JAK2*, *PPM1D*, *SF3B1*, *SRSF2*, and *U2AF1* individually conferred higher mortality risk. In human plaques, CHIP mutations were found in lesional macrophages. *TET2* CHIP carriers showed increased necrotic core size, inflammation, and reduced plaque stability. Multi-omics profiling revealed up-regulation of lipid metabolism and inflammatory pathways. *TET2*^+/−^ macrophages exhibited increased *LDLR* expression and lipid uptake, linked to enhanced chromatin accessibility at the *LDLR* promoter. These findings were confirmed in carotid plaques, which showed increased *LDLR* and inflammasome-related gene expression in *TET2* CHIP carriers.

**Conclusions:**

Clonal haematopoiesis of indeterminate potential is a predictor of mortality in CAD patients. *TET2* mutations promote a pro-atherogenic macrophage phenotype via *LDLR* up-regulation and inflammatory activation, linking epigenetic dysregulation to adverse outcomes in CAD.

## Structured Graphical Abstract

**Structured Graphical Abstract:**
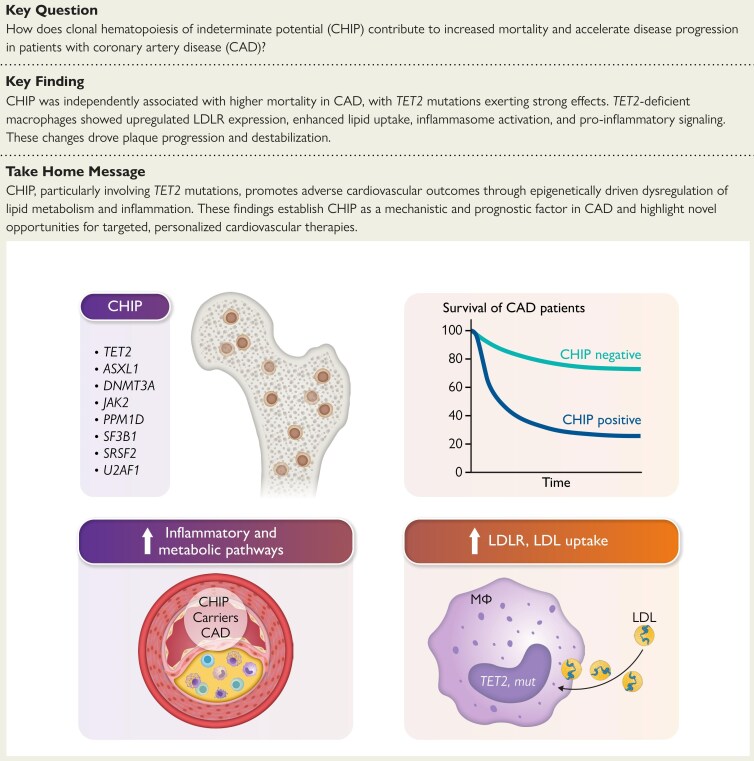
Clonal haematopoiesis increases mortality risk and drives plaque vulnerability in coronary artery disease (CAD). Clonal haematopoiesis of indeterminate potential (CHIP) is independently associated with increased all-cause mortality in patients with CAD. Eight CHIP driver genes—including *TET2*, *ASXL1*, *DNMT3A*, *JAK2*, *PPM1D*, *SF3B1*, *SRSF2*, and *U2AF1*—were individually linked to higher mortality risk. Histological and molecular profiling of human atherosclerotic plaques revealed a more advanced and vulnerable phenotype in *TET2*-mutated cases. Functional studies in *TET2*-deficient macrophages demonstrated up-regulated LDL receptor expression, increased lipid uptake, and activation of inflammatory pathways, establishing a mechanistic link between CHIP, altered macrophage function, and atherosclerotic progression.

**See the editorial comment for this article ‘Clonal haematopoiesis and cardiovascular diseases: challenges and opportunities’, by C. De Jeronimo Diaz**  ***et al*****., https://doi.org/10.1093/eurheartj/ehaf812.**

## Introduction

Cardiovascular disease (CVD) remains the leading cause of mortality worldwide, with coronary artery disease (CAD) representing its most prevalent and life-threatening manifestation.^1,2^ Despite advances in risk stratification and treatment, the molecular drivers that contribute to CAD progression and adverse outcomes remain incompletely elucidated. Emerging evidence suggests that clonal haematopoiesis of indeterminate potential (CHIP)—a phenomenon characterized by the age-related expansion of haematopoietic stem cells harbouring somatic mutations—may play a pivotal role in cardiovascular pathology,^3–9^ including increased risk of cardiovascular events.^10–13^

Clonal haematopoiesis of indeterminate potential is defined by somatic mutations in genes regulating epigenetic remodelling, inflammation, and immune cell function, including *TET2*, *DNMT3A*, and *ASXL1*.^10,14^ These mutations alter leukocyte behaviour, promoting a pro-inflammatory and metabolically active macrophage phenotype, which has been implicated in accelerated atherogenesis.^8,15–21^ Experimental data suggest that CHIP-mutated macrophages exhibit increased inflammasome activation, cytokine secretion, and metabolic perturbation, leading to plaque progression and instability—key determinants of CAD severity and mortality.^22^

While previous studies have linked CHIP to an increased risk of cardiovascular events, the extent to which specific CHIP mutations contribute to mortality and plaque vulnerability in CAD remains incompletely defined. In this study, we comprehensively assessed the prognostic impact of CHIP in a large cohort of CAD patients and investigated the functional consequences of CHIP-associated mutations in atherosclerotic plaques and macrophages. Leveraging deep sequencing, high-sensitivity proteomics, and transcriptomic profiling, we identified *TET2* mutations as a key driver of *LDLR* up-regulation, heightened lipid accumulation, and inflammasome activation in macrophages—mechanisms that promote CAD complexity and mortality.

Our findings establish CHIP, particularly *TET2* mutations, as an independent risk factor for worsened CAD outcomes, providing novel insights into its pathophysiological role in inflammation, lipid metabolism, and plaque instability. These results highlight CHIP as a potential therapeutic target, paving the way for personalized risk stratification and intervention strategies in CAD patients.

## Methods

### Ethics approval

The institutional review board and Ethics Committee of the Technical University of Munich, Germany, approved our work on CHIP in patients and post-mortem tissue (273/18 S—06/27/2018, 2018-325-S-KK—08/22/2018, and 2023-54-S-KK—03/24/2023). The use of human Stockholm-Tartu Atherosclerosis Reverse Networks Engineering Task (STARNET) samples has been approved by the Estonian Bioethics and Human Research Committee (Ministry of Social Affairs) (IRB 385/M-1—12/18/2023). The use of human Athero-Express samples has been approved by the Medical Research Ethics Committee of University Medical Center Utrecht (TME/C-01.18). The studies are based on the written informed consent of donors, in accordance with the guidelines and regulations for the use of biological material of human origin. All studies were conducted in accordance with the provisions of the Declaration of Helsinki and the International Conference on Harmonization guidelines for good clinical practice.

### Data accessibility

The data used in this study are available in permanent repositories. Human CAD patient data and data from Munich cardIovaScular StudIes biObaNk (MISSION) can be requested by qualified researchers at the German Heart Center Munich from the corresponding authors. Human data from STARNET are accessible through the Database of Genotypes and Phenotypes (dbGAP). *In vitro* data of human monocyte THP-1 cell experiments and human CVD patient data from Athero-Express can be requested by qualified researchers from the corresponding authors.

### Clinical cohort from Munich

**Figure 1:**
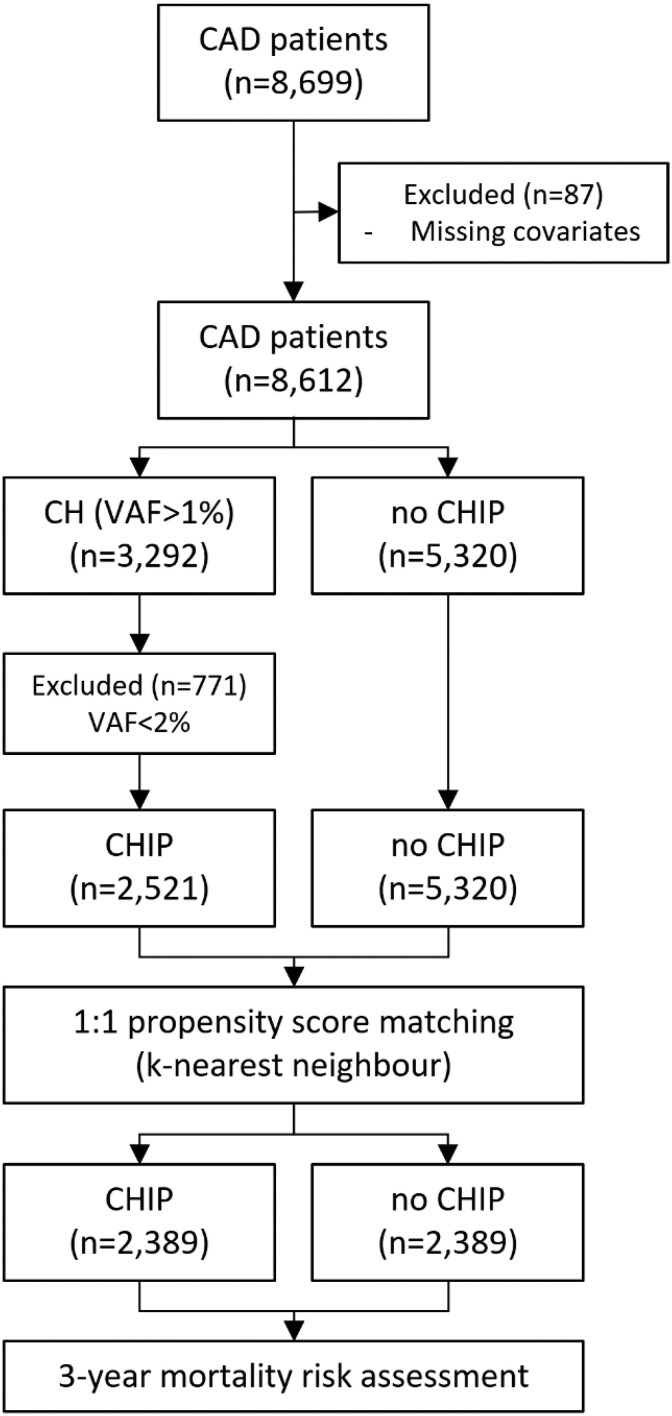
Flow diagram 3-year mortality analysis in CHIP CAD patients. Patients without known malignancies were screened for CHIP-defining mutations based on angiogram-confirmed CAD. Patients with complete covariates and VAF ≥ 2%, were considered CHIP positive for further analysis. Potential bias was minimized using propensity score matching. Mortality risk was compared between matched CHIP-positive and CHIP-negative individuals. CHIP, clonal haematopoiesis of indeterminate potential; CAD, coronary artery disease; VAF, variant allele frequency

The German Heart Center Munich DNA biobank consists of >40 000 individuals. Most relevant phenotypes within this biobank are acute and chronic CAD, as well as valvular and rhythm disorders. The CHIP screening cohort at the German Heart Center Munich was retrospectively established based on a valid biobank agreement using the hospital information system based on chronic CAD (ICD-10 code I25) confirmed angiographically and self-reported ancestry. Exclusion criteria at the time point of DNA sampling were acute myocardial infarction (ICD-10 codes I21–I24) and active malignancies (ICD-10 codes C01–D90). Individuals with relevant covariate data missing were excluded. After preselection, CHIP mutation carrier status was assessed in >8600 individuals with European ancestry, angiographically confirmed chronic CAD, and without known active malignancies (*Table 1* and *Figure 1*). Patients received guideline-recommended medication, including anti-platelet and cholesterol-lowering therapy. Mortality status was queried in the German National Population Registry.

**Table 1:** Characteristics of coronary artery disease patients at baseline of the unmatched cohort and after propensity score matching

	Unmatched cohort			Matched cohort		
	CHIP (*n* = 2521)	No CHIP (*n* = 5320)	SMD	CHIP (*n* = 2389)	No CHIP (*n* = 2389)	SMD
Age (years)	74.2 ± 9.1	67.8 ± 11.0	0.61	73.9 ± 9.1	74.0 ± 9.1	0.00
Male sex	1883 (74.7%)	4136 (77.7%)	−0.07	1785 (74.7%)	1790 (74.9%)	0.00
BMI (kg/m²)	27.3 ± 4.4	27.6 ± 4.6	−0.08	27.3 ± 4.4	27.1 ± 4.4	0.04
Height (m)	1.7 ± .1	1.7 ± 0.1	−0.15	1.7 ± 0.1	1.7 ± 0.1	−0.01
Weight (kg)	81.0 ± 15.2	83.3 ± 16.2	−0.15	81.1 ± 15.3	80.7 ± 15.3	0.03
Hypertension	2236 (88.7%)	4549 (85.5%)	0.09	2124 (88.9%)	2134 (89.3%)	−0.01
Smoking ever	938 (37.2%)	2256 (42.4%)	−0.11	885 (37.0%)	870 (36.4%)	0.01
Diabetes	675 (26.8%)	1314 (24.7%)	0.05	642 (26.9%)	621 (26.0%)	0.02
Family history	647 (25.7%)	1664 (31.3%)	−0.12	609 (25.5%)	595 (24.9%)	0.01
Normal LV function	1643 (65.2%)	3421 (64.3%)	0.02	1561 (65.3%)	1576 (66.0%)	−0.01
Reduced LV function	878 (34.8%)	1899 (35.7%)	−0.02	828 (34.7%)	813 (34.0%)	0.01
Prior MI	606 (24.0%)	1430 (26.9%)	−0.06	577 (24.2%)	556 (23.3%)	0.02
Prior stroke	11 (0.4%)	21 (0.4%)	0.01	11 (0.5%)	13 (0.5%)	−0.01
CKD	248 (9.8%)	393 (7.4%)	0.09	242 (10.1%)	233 (9.8%)	0.01
COPD	154 (6.1%)	232 (4.4%)	0.08	149 (6.2%)	128 (5.4%)	0.04
Hemoglobin (g/dL)	13.4 ± 1.8	13.8 ± 1.7	−0.23	13.4 ± 1.8	13.5 ± 1.8	−0.05
MCV (fl)	89.3 ± 6.0	88.6 ± 5.9	0.12	89.3 ± 6.0	88.8 ± 6.4	0.07
RDW (fl)	45.7 ± 5.6	44.2 ± 4.7	0.31	45.7 ± 5.6	44.8 ± 4.9	0.16
Leukocytes (10^9/L)	7.6 ± 2.8	7.6 ± 2.7	0.02	7.6 ± 2.8	7.5 ± 2.9	0.06
Thrombocytes (10^9/L)	213.3 ± 71.7	213.6 ± 59.2	−0.01	214.1 ± 72.2	209.2 ± 58.7	0.07
Creatinine (mg/dL)	1.1 ± 0.4	1.1 ± 0.4	0.12	1.1 ± 0.4	1.1 ± 0.4	0.01
GFR (MDRD) (mL/min)	70.0 ± 21.0	76.0 ± 21.9	−0.28	70.3 ± 20.9	70.9 ± 21.6	−0.03
ALT (U/L)	27.0 ± 57.6	29.5 ± 24.9	−0.07	27.1 ± 59.0	26.8 ± 23.5	0.01
Total chol. (mg/dL)	164.7 ± 44.6	169.6 ± 47.2	−0.11	164.7 ± 44.7	167.1 ± 44.0	−0.05
LDL-cholesterol (mg/dL)	93.9 ± 38.2	97.4 ± 40.3	−0.09	94.0 ± 38.2	94.5 ± 37.7	−0.01
HDL-cholesterol (mg/dL)	53.2 ± 15.5	52.6 ± 15.6	0.04	53.1 ± 15.5	54.6 ± 15.7	−0.10
Triglycerides (mg/dL)	127.3 ± 87.3	137.3 ± 111.0	−0.10	128.0 ± 88.5	126.4 ± 73.3	0.02
CRP (mg/L)	6.0 ± 15.1	5.3 ± 15.7	0.04	5.7 ± 13.7	5.1 ± 12.9	0.04

Plus–minus values are means ± SD. BMI, body mass index; ALT, alanine transaminase; CI, confidence interval; CKD, chronic kidney disease; COPD, chronic obstructive pulmonary disease; CRP, C-reactive protein; GFR, glomerular filtration rate; HDL, high-density lipoprotein; LDL, low-density lipoprotein; LV, left ventricular; MCV, mean corpuscular volume; MDRD, modification of diet in renal disease; MI, myocardial infarction; RDW, red blood cell distribution width; SMD, standardized mean difference.

### Propensity score matching and mortality analysis (clinical cohort)

We estimated the average treatment effect on the treated by defining CHIP-positive CAD patients as the exposed group. These individuals were matched to CHIP-negative CAD patients (unexposed group) using nearest-neighbour caliper matching without replacement, based on propensity scores derived from a multivariable logistic regression model. This direction of matching was chosen to evaluate the impact of CHIP on mortality within the CHIP-positive population. Propensity scores were estimated using a logistic regression model with age, sex, diabetes mellitus, arterial hypertension, family history of CVD, smoking status, left ventricular function, prior myocardial infarction, atrial fibrillation, chronic obstructive pulmonary disease, chronic kidney disease, and prior stroke as predictors. The caliper width was set to 20% of the standard deviation of the propensity score log-odds. To assess covariate balance between groups before and after matching, standardized mean differences (SMDs) were calculated. The SMD was defined as the difference in means (for continuous variables) or proportions (for categorical variables) between groups, divided by the pooled standard deviation. An SMD < 0.1 was considered indicative of adequate balance. Gene-specific sub-cohorts were matched to paired controls (1:2 ratio if ≥500 subjects, otherwise 1:5 ratio). Survival rates were visualized using Kaplan–Meier curves over a 3-year follow-up period, with the study entry date being the CHIP sequencing date and the endpoint being all-cause mortality. Hazard ratios (HR) were calculated using Cox proportional hazards models for each matched set, as well as subsets with variant allele frequency (VAF) ≥2% and VAF ≥10%, with CHIP status as the sole predictor. To estimate the effect of VAF on cumulative hazard, we fitted a Cox proportional hazards model, including maximal VAF and relevant clinical covariates. Cumulative hazard was visualized using the plot_partial_effects_on_outcome function from the lifelines package, modelling hypothetical subjects across a VAF range of 0%–45% while holding other covariates constant. The proportional hazards assumption was assessed by examining Schoenfeld residuals. Data analysis and matching were performed using Python (version 3.12.2). The statsmodels package (version 0.14.1) provided the logistic regression model for propensity score estimation. Kaplan–Meier and Cox’s analyses were conducted using the lifelines package (version 0.27.8).

### Munich post-mortem biobank (MISSION)

The MISSION was initiated in 2019 and comprises cardiovascular-relevant post-mortem tissues, including whole blood, plasma, liver, myocardium, coronary, and carotid artery from >1300 deceased individuals sampled in formalin-fixed paraffin embedded (FFPE) and fresh frozen at −80°C. Due to legal regulations, no clinical information is available. MISSION provides the full spectrum of coronary phenotypes from healthy to severe CAD. Prior to freezing and sequencing, tissues underwent washing steps and were subsequently conserved in phosphate-buffered saline (PBS) and dimethyl sulfoxide. Histological assessment of FFPE samples was performed by applying the main classification stages from the Modified American Heart Association classification based on four histological staining methods.^23–25^ For this study, 540 individuals with histologically confirmed CAD were selected and further analysed by deep DNA sequencing.

### DNA extraction from blood and tissue (MISSION)

DNA of blood and cardiovascular tissues, including the proximal part of atherosclerosis-affected left anterior descending (LAD) coronary, atherosclerosis-affected carotid artery, and left ventricular heart muscle, was extracted following an adapted Maxwell DNA Blood Kit (Promega) protocol. Very condensed blood was mixed with lysis buffer and proteinase K, incubated at room temperature and 65°C with rotation, and processed in the Maxwell RSC 48 system. For tissue samples (50 mg of coronary artery, carotid, and left ventricle), tissues were homogenized in incubation buffer with 1-thioglycerol and proteinase K, incubated at 65°C, and processed similarly. DNA was isolated using the Maxwell RSC 48 system and quantified with a Qubit 3.0 fluorometer (ThermoScientific).

### Clonal haematopoiesis of indeterminate potential gene sequencing (clinical cohort, MISSION, and Athero-Express)

A targeted gene panel included the 13 most frequently mutated CHIP genes (*ASXL1*, *CALR*, *CBL*, *DNMT3A*, *JAK2*, *MPL*, *PPM1D*, *SF3B1*, *SRSF2*, *TET2*, *TP53*, *U2AF2*, and *ZRSR2*). The library preparation for enrichment was performed from 150 ng DNA per sample with the Illumina DNA Prep Kit (Illumina, San Diego, CA, USA) using Unique Dual Indices and enzymatic DNA fragmentation. Target enrichment was done using the IDT Hybridization Capture Protocol and a corresponding lockdown gene panel (IDT Integrated DNA Technologies, Coralville, IA, USA). Sequencing was performed on a NovaSeq 6000 (Illumina) with paired-end sequencing mode (2 × 101 cycles) and a target coverage of 2000×. The mean sequencing depth across all individuals from Munich and Utrecht was >3500-fold with a minimum sequencing depth of >1000-fold. De-duplication was performed to eliminate duplicated, identical PCR reads before alignment to improve the accuracy of the calculated VAF. Variant calling of single nucleotide variants and small InDels (<50 bp) was carried out with the variant callers Dragen, Pisces (Illumina) and Pindel (Wellcome Sanger Institute, Cambridgeshire, UK) followed by a manual annotation and classification of the variants by in-house routine accredited (ISO 15189 and College of American Pathologists) workflow of the Munich Leukemia Laboratory (MLL, Munich, Germany). This workflow uses open-access databases such as COSMIC, IRAC, ClinVAR, dbSNP, and gnomAD, as well as the in-house databases specialized in haematologic diseases. Protein-truncating and consensus splice-site-affecting variants were classified as pathogenic variants. Non-synonymous changes were included, considering different aspects: variants were well annotated for pathogenicity in haematologic malignancies (several definite submissions to COSMIC, IRAC, ClinVAR, or MLL database), within a functional domain of the protein, or likely somatic, based on a VAF <25%; for *ASXL1*, only protein-truncating variants were considered pathogenic. Other rare non-protein-truncating variants were defined as variants of uncertain significance and were not considered in further analyses. Sequencing was performed at a limit of detection of 1% VAF, and detected variants with a VAF <5% were independently confirmed by amplicon sequencing on a MiSeq (Illumina). For MISSION samples, a previously established panel containing 594 amplicons in 56 genes was used in the Illumina TruSeq Custom Amplicon Low Input assay.^26^

### Visualization of clonal haematopoiesis of indeterminate potential mutations in human plaques (MISSION)

Mutation-specific Fluorescence In-Situ Hybridization (mutaFISH^™^) analysis on the *DNMT3A* gene was performed on 3–5 µm thick FFPE tissue sections from human coronary and carotid arteries of individuals harboring *DNMT3A* c.2245C>T and c.2333T>G mutations. c.2245C>T (R749C) is a well-characterized CHIP driver mutation, known to impair *DNMT3A* enzymatic function, alter DNA methylation, and promote myeloid expansion. c.2333T>G (V778G), though less frequently reported, is a loss-of-function variant with structural modelling suggesting disruption of *DNMT3A*-mediated DNA methylation, selected in collaboration with the MutaFISH^™^ manufacturer to ensure optimal probe design. *In situ* rolling circle technology was performed using custom-made dual colour *DNMT3A* mutation and wild-type-specific FISH probes (Abnova, Taiwan, for details see supplement).^27,28^ It is based on the manufacturer’s protocol for mutaFISH^™^ RNA Accessory Kit and the protocol for muta FISH^™^ HER2wt RNA probes with some modifications in target retrieval, washing buffer, and incubation. Wild-type *DNMT3A* served as a control probe. Negative controls were used to establish unspecific binding and background. All reagents were prepared with RNAse-free (diethyl pyrocarbonate-treated) water/PBS. For hybridization, a Boekel Scientific Slide Moat oven was used. Images were acquired at the ZEISS Axioscan 7 slide scanner, and analysis was performed using Zeiss Software ZEN 3.5 blue edition. MutaFISH^™^ serves as a tool for the spatial visualization of CHIP mutations at the RNA level, providing insights into the localization of CHIP-mutated cells within human plaques.

### High-sensitivity proteomics on human artery plaques (MISSION)

Formalin-fixed paraffin-embedded tissue sections were mounted on slides, and artery tissue was hematoxylin and eosin stained for pathology assessment. Tissue slides were baked at 55°C for 20 min and deparaffinized. The tissue was scraped off, collected in a 96-well plate, and lysed by adding 42 µL lysis buffer (70 mM triethylammonium bicarbonate, 0.013% n-dodecyl β-D-maltoside) followed by heating to 90°C for 60 min. Samples were sonicated (LE220-plus Covaris Focused-ultrasonicator) and further lysed by adding 7 µL acetonitrile and incubating at 75°C for 60 min. After cooling, samples underwent overnight proteolytic digestion at 37°C with Trypsin and LysC. Post-digestion, samples were centrifuged, transferred to a new plate, and quenched with 6 µL 10% TFA. Peptide content was quantified using a tryptophan assay. Approximately 40 µg of each sample were purified using styrene–divinylbenzene reversed-phaseS stage tips, eluted, dried, and resuspended in buffer EvoA (0.1% formic acid).^29^ Peptide concentration was re-determined, and 500 ng were loaded on Evotip Pure.

For DIA LC–MS Acquisition, peptides were separated using the Evosep One LC system with a 44 min gradient over a 15 cm PepSep Series column (Bruker Daltonics) at 50°C, coupled to a timsTOF HT instrument. The data-independent acquisition – parallel accumulation–serial fragmentation (dia-PASEF) method, generated using py_diAID, employed two IM windows per dia-PASEF scan with variable isolation window widths covering over 95% of measurable precursors.^30,31^ Twenty isolation windows spanned an *m/z* range of 350–1200 and an ion mobility range between 1.3 and 0.7 V cm^−2^, with collision energy decreasing from 59 to 20 eV.

MS raw data were analysed using DIA-NN version 1.8.1 in library-free mode against the human reference proteome database (Uniprot, November 2023) with default search parameters. Match-between-run was enabled, heuristic protein inference was disabled, and protein intensities were re-quantified using directLFQ.^32,33^ False discovery rate (FDR) was set to 1% at both peptide precursor and protein levels.

### Proteomics data analysis (MISSION)

Protein intensities were log2 transformed, and the proteome dataset was filtered for 70% valid values across all samples. In total, 5189 proteins were quantified and filtering for 70% valid values across all samples resulted in a dataset of 3442 proteins with a data completeness of >94%. Missing values were imputed using a downshifted normal distribution, assuming that missingness was not at random, but primarily reflected low-abundance proteins falling below the detection threshold of the instrument.^34^ Batch correction was performed with pyCombat. All subsequent analyses were conducted using R version 4.3.2. Age and sex matching between *TET2* CHIP mutation carriers and controls was performed using the MatchIt package with a caliper width of 0.2.^35^ Differentially expressed proteins were identified using the eBayes function from the limma package.^36^ Gene set enrichment analysis (GSEA) was performed with the gseGO function from the clusterProfiler package,^37,38^ applying a Benjamini-Hochberg adjusted *P*-value cutoff of .05. Results were visualized with the DOSE, ggplot2, and forcats packages,^39–41^ highlighting normalized enrichment scores for the top enriched gene ontology (GO) terms. Markov clustering and network visualization of enriched GO terms were conducted using the aPEAR package.^42^ Differential expression of specific proteins of interest was further validated with the ggstatsplot package.^43^

### Whole-genome sequence and macrophage RNA-seq data analysis (STARNET)

Coronary artery disease patients from the STARNET undergoing coronary artery bypass grafting (*n* = 941) donated tissue samples, including blood-derived monocytes.^44^ DNA from whole blood was isolated with the QIAmp DNA Blood Midi kit (Qiagen). Library preparation and sequencing were performed at Beijing Genomic Institute (BGI). Genomic DNA samples were randomly fragmented by Covaris technology, and 350 bp fragments were selected. End repair of DNA fragments was performed and an ‘A’ base was added at the 3′ end of each strand. Adapters were then ligated to both ends of the end-repaired/dA-tailed DNA fragments and amplified by ligation-mediated PCR, followed by single-strand separation and cyclization. Rolling circle amplification was performed to produce DNA nanoballs (DNBs). The qualified DNBs were loaded into patterned nanoarrays and pair-end reads were read through on the BGISEQ-500 platform. High-throughput sequencing was performed for each library to ensure that each sample met the average sequencing coverage requirement of 35×. Sequencing-derived raw image files were processed by BGISEQ-500 base-calling software with default parameters, and the sequence data of each individual was generated as paired-end reads. Raw data in FASTQ format was filtered and raw reads with low quality were removed. Data were aligned to the human reference genome (GRCh37/HG19) by Burrows–Wheeler Aligner (BWA) v0.7.12^45,46^ and variant calling was performed by Genome Analysis Toolkit (v3.3.0)^47^ with duplicate reads removed by Picard tools v1.118.^48^ The HaplotypeCaller of GATK was used to call both single nucleotide polymorphism (SNP) and InDels simultaneously via local *de novo* assembly of haplotypes in a region showing signs of variation.^49,50^ Base quality scores were recalibrated using GATK BaseRecalibrator, and SNPs recalibration was performed using GATK VariantRecalibrator function.^51,52^

### Somatic variant identification and validation in STARNET whole genome sequencing

Heterozygous missense, nonsense, InDel, and splicing variants of 1 or 2 bp in coding regions of *TET2* defining CHIP mutations were identified by filtering data from vcf files according to: (1) genotype quality >30; (2) minor allele frequency <1% in 1000 genome database; and (3) minor allele frequency <1% in the cohort. Variant analysis in RNA-seq data in monocyte-derived macrophages from STARNET subjects was performed for the identification and validation of somatic mutations identified via whole-genome sequencing.

### Macrophage RNA sequencing and data processing in STARNET

Somatic mutations identified via whole genome sequencing (WGS) were further validated, and the effects of CHIP mutations on gene expression were explored in RNA-seq data from monocyte-derived macrophages. After centrifugation of whole blood, the cell pellet was washed with 1× PBS, and the cells were plated. After 3–6 h, monocytes adhered to the plate, and non-adherent erythrocytes and lymphocytes could be washed off during adhesion purification. The monocytes were subsequently cultured in human serum, stimulated, and differentiated into macrophages over 48–72 h. Finally, macrophages were harvested, and RNA was isolated.^44,53^

RNA library preparation was based on the Ribo-Zero library preparation method using the Illumina TruSeq non-stranded mRNA kit. Samples were randomized to prevent batch effects. Sequencing was performed with paired-end reads of 100 bp on an Illumina HiSeq, and quality control was performed using FASTQC3. GENCODE was used to quantify the expression of genes and isoforms and mapped to the human genome using STAR4. The average coverage was >40 million reads per sample. Only samples with more than 1 million unambiguously assigned reads were used for further analysis.^53^

### Genetics, imputation, and expression quantitative trait loci analysis in STARNET

Genetic data were analysed on a genome build 37 background using GenomeStudio (Illumina). After genetic sex confirmation, quality control was performed using PLINK v 2.05. Data were imputed using the HRC r1.1 2016 reference panel using minimac4. *Cis*- and *trans*-regulated expression quantitative trait loci (eQTLs) in macrophages were determined using R package matrix eQTL v.2.1.1. Adjustments were made for age, sex, body mass index, and the first five genetic principal components. *Cis*-regulatory SNPs within 1 megabase of the respective gene were determined by a linear regression model (hg19 genomes). An FDR <5% was considered statistically significant.

### Co-expression network modules and their associations with clinical traits in STARNET

Correlation patterns between gene expression were analysed using the R package Weighted Gene Co-expression Network Analysis to construct correlation networks and identify co-expression modules. Correlations of gene expression with clinical traits were calculated using Spearman correlation. The association of co-expression modules with clinical traits was analysed using Fisher’s exact test to calculate the enrichment of the number of significantly correlated genes in each module. Regulatory networks were reconstructed using GENIE3, based on a random forests ensemble method.^54^ The network was enriched with transcription factors and *cis*-eQTL-regulated genes from macrophages as candidate regulators.^55,56^

### Differential gene expression and pathway analysis in STARNET

Differences in gene expression between CHIP mutation carriers and controls were investigated using the R package limma.^36^ Covariates in the linear regression model were age, sex, body mass index, renal function, and dyslipidemia. Adjusted *P*-values <.05 were considered statistically significant. Differentially expressed genes were analysed for overrepresentation of genes from genome-wide association studies to CAD using PhenoScanner.^57^ Differentially expressed genes were analysed by binomial test for enrichment of GO terms and by GSEA. The maximum size of a gene set in GSEA was set to 3000. Bonferroni-corrected *P*-values <.05 were considered statistically significant.

### Generation of genome-edited TET2 THP-1 monocytes

Cas9 and single guide RNA ribonucleoprotein complex targeting the *TET2* open reading frame were transiently transfected into THP-1 cells via electroporation (Lonza). The AAVS1 safe harbor locus was targeted to generate control lines. Per cohort, three independent clonally derived lines were isolated and expanded. Mono-allelic editing was confirmed by amplicon sequencing (Illumina) with analysis using the ampliCan pipeline.^58^

### In-vitro cholesterol loading of TET2 CHIP macrophages

*TET2* and control AAVS1 edited THP-1 monocytes were differentiated into macrophages using 150 nM phorbol 12-myristate 13-acetate for 24 h. Cell lines were then treated with 5 μg/mL phrodo-LDL. The uptake of pHrodo-LDL (Thermofisher L34356) was measured every hour using live-cell imaging (Incucyte, Sartorius AG). Lipid uptake was determined by measuring the total integrated intensity of pHrodo normalized to cell confluency. Oxidized-LDL (oxLDL) uptake was measured after treatment with fluorescently conjugated DiI-oxLDL (Thermofisher L34358) by flow cytometry.

### RNA sequencing of TET2 clonal haematopoiesis of indeterminate potential macrophages

RNA was extracted using the RNeasy Mini Kit (Qiagen) following the manufacturer’s instructions. RNA integrity was assessed using the Agilent 2100 Bioanalyzer, with samples showing an RNA integrity number >7.0 proceeding to library preparation. RNA libraries were prepared using the Illumina TruSeq Stranded mRNA Library Prep Kit with poly-A selection, following the manufacturer’s protocol. Libraries were pooled and randomized to mitigate batch effects. Sequencing was performed on an Illumina HiSeq 4000 platform, generating 100 bp paired-end reads, with a target depth exceeding 40 million reads per sample. Quality control was performed using FastQC to assess read quality, adapter content, and sequence duplication levels. Reads were aligned to the GRCh38/hg38 human reference genome using the STAR aligner (v2.7.3a) with default parameters. Transcript quantification was performed using GENCODE v35 gene annotations. Only samples with more than 1 million uniquely mapped and assigned reads were retained for downstream analysis. Normalization and differential gene expression analyses were conducted using DESeq2 (R v1.44.0) with multiple testing correction.^59^  *P*-values <.05 were considered statistically significant.

### Athero-Express Biobank Study and TET2 clonal haematopoiesis of indeterminate potential status assessment

The Athero-Express Biobank Study (AE-Biobank) is a longitudinal biobank enrolling patients undergoing carotid endarterectomy^60^ at two Dutch hospitals. The study collects clinical data, blood samples, and atherosclerotic plaques following standardized protocols. Samples were snap-frozen and stored at −80°C for RNA sequencing. The cohort study design has been previously described in detail.^61–63^ Clonal haematopoiesis of indeterminate potential status was determined using the same sequencing methodology as in MISSION Biobank in a total of 470 individuals with available DNA and carotid endarterectomy samples. After assessing the CHIP status, *TET2* CHIP carriers were matched to non-*TET2* carriers based on age, sex, body mass index, dyslipidemia, and kidney disease. Propensity score matching was conducted using logistic regression, with a 1:3 matching ratio.^35^

### Plaque RNA sequencing and data processing in Athero-Express

Bulk RNA sequencing samples were assessed for the atherosclerotic plaques. RNA was isolated using standardized protocols, and CEL-seq2 library preparation enabled 3′ end sequencing with unique molecular identifiers.^64,65^ Sequencing was performed on an Illumina NextSeq 500 platform, generating paired-end 2 × 75 bp reads. Reads were aligned to the human cDNA reference genome (BWA v0.7.13), and UMI-based correction eliminated duplicates. Data were quantile-normalized before analysis. Differential gene expression was analysed using DESeq2 (R v1.44.0) with multiple testing correction.^59^  *P*-values <.05 were considered statistically significant.

## Results

### Mortality analysis in clonal haematopoiesis of indeterminate potential-carrying coronary artery disease patients

**Figure 2:**
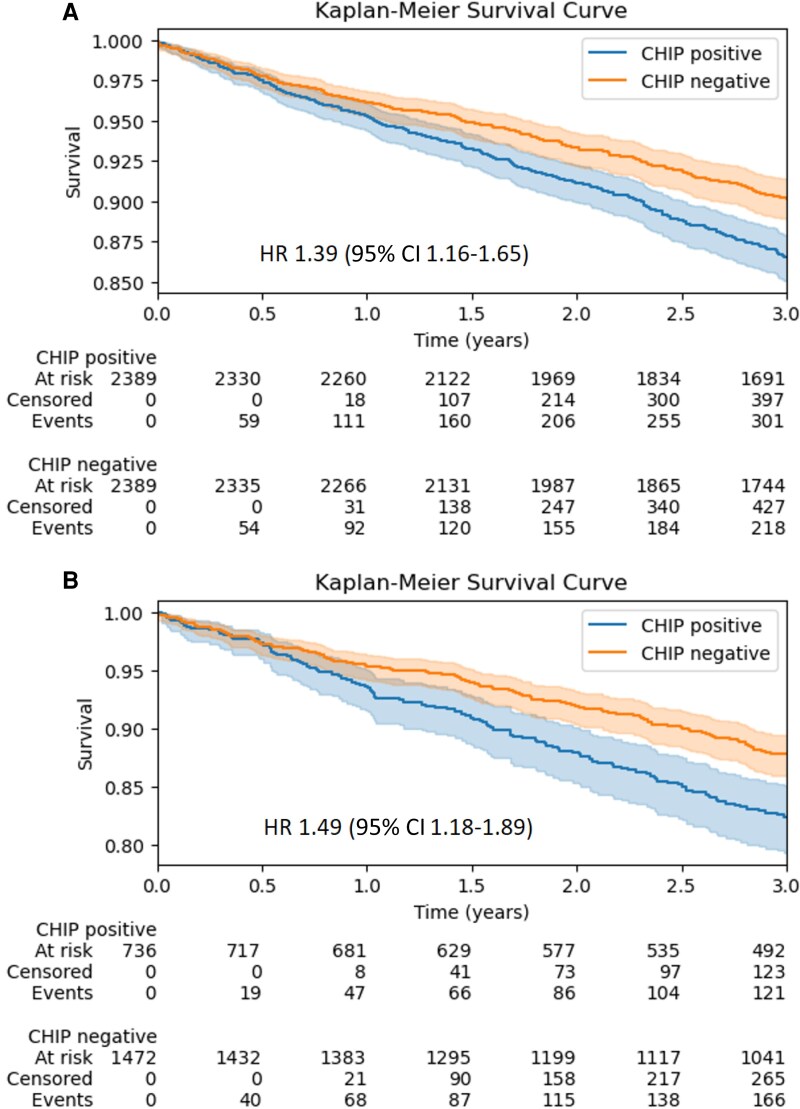
CHIP is associated with increased mortality risk in CAD patients. (*A*) Kaplan–Meier curves show the cumulative survival probability in matched CHIP CAD vs no CHIP CAD patients over 3 years for all CHIP patients with a VAF ≥ 2%. (*B*) Kaplan–Meier curves show the cumulative survival probability in matched CHIP CAD vs no CHIP CAD patients over 3 years for all CHIP patients with a VAF ≥ 10%. CHIP, clonal haematopoiesis of indeterminate potential; CAD, coronary artery disease; VAF, variant allele frequency

We identified 3507 CHIP-defining mutations with a VAF ≥ 2% in 2521 (29.3%) of 8612 CAD patients (see Supplementary data online, *Figure S1* and Supplementary data online, *Table S1*). The mean age of CHIP mutation carriers was 74.2 ± 11.0 years compared with 67.8 ± 9.1 years in non-carriers. Pairwise matching was possible for 2389 CHIP mutation carriers and 2389 non-carriers (*Figure 1* and *Table 1*). Three-year all-cause mortality was increased in CAD patients carrying any CHIP mutation (*n* = 301; 12.6%) compared with matched CAD controls (*n* = 218; 9.1%) [HR 1.39, 95% confidence interval (CI) 1.16–1.65] (*Figure 2* ). Further, mortality risk was increased individually for eight CHIP genes including *ASXL1* [HR 1.43 (95% CI 1.01–2.03)], *DNMT3A* [HR 1.24 (95% CI 1.01–1.52)], *JAK2* [HR 1.93 (95% CI 1.09–3.42)], *PPM1D* [HR 1.74 (95% CI 1.19–2.56)], *SF3B1* [HR 1.67 (95% CI 1.07–2.59)], *SRSF2* [HR 1.90 (95% CI 1.18–3.05)], *TET2* [HR 1.53 (95% CI 1.20–1.95)], and *U2AF1* [HR 1.99 (95% CI 1.02–3.87)]. Given limited power, *CALR* [HR 4.09 (95% CI 0.68–24.56)] and *ZRSR2* [HR 1.91 (95% CI 0.92–3.96)] did not reach statistical significance at VAF ≥2%. However, relative mortality risk was increased across all 13 genes of the customized panel (HR 1.24–4.09).

When restricted to carriers with large clone sizes defined as CHIP VAF ≥10% (*n* = 736), mortality risk increased in CHIP (*n* = 121 deaths; 16.4%) compared with matched non-CHIP (11.3%) patients across the combined 13 gene panel [HR 1.49 (95% CI 1.18–1.89)] (*Figure 2*). With respect to increasing VAFs, we observed a quantitative effect on mortality [HR 1.02 (95% CI 1.01–1.03)] (see Supplementary data online, *Figure S2*). No violations of the proportional hazards assumption were detected based on the examination of Schoenfeld residuals in any of the analyses.

### CHIP in human post-mortem samples (MISSION)

The MISSION tissue bank encompassed 540 individuals, deceased at an average age of 75.1 years, with histologically confirmed CAD. Among those, we identified 217 individuals (40.2%) with a total of 329 individual CHIP mutations (VAF ≥2%) (see Supplementary data online, *Table S2*). The prevalence of CHIP increased with age (*R* = .78; *P* < .001).

We selected 26 *TET2* CHIP CAD mutation carriers for CHIP detection in left ventricular myocardium and atherosclerosis-affected carotid and coronary artery samples. The corresponding CHIP mutation was detected in 68 of 78 (87.2%) tissue samples (see Supplementary data online, *Figure S3*), whereas no *de novo* mutation was detected at the tissue level.

Tissue-specific signatures of atherosclerosis-affected LAD coronary arteries were assessed in 26 *TET2* CHIP CAD mutation carriers and 13 paired-matched controls without CHIP (see Supplementary data online, *Table S3*). Histological assessment revealed a more severe atherosclerotic phenotype in *TET2* CHIP CAD carriers. Plaques of *TET2* mutation carriers in comparison to matched controls showed larger necrotic core size (*P* = .04), increased inflammatory cell infiltration (*P* = .01), reduced elastin integrity (*P* = .04) and more severe calcification (*P* = .03) (see Supplementary data online, *Table S3* and Supplementary data online, *Figure S4*). These findings suggest that *TET2* CHIP CAD carriers exhibit tissue-level features and protein signatures consistent with more vulnerable and inflammatory plaques, which may promote adverse cardiovascular outcomes.

**Figure 3:**
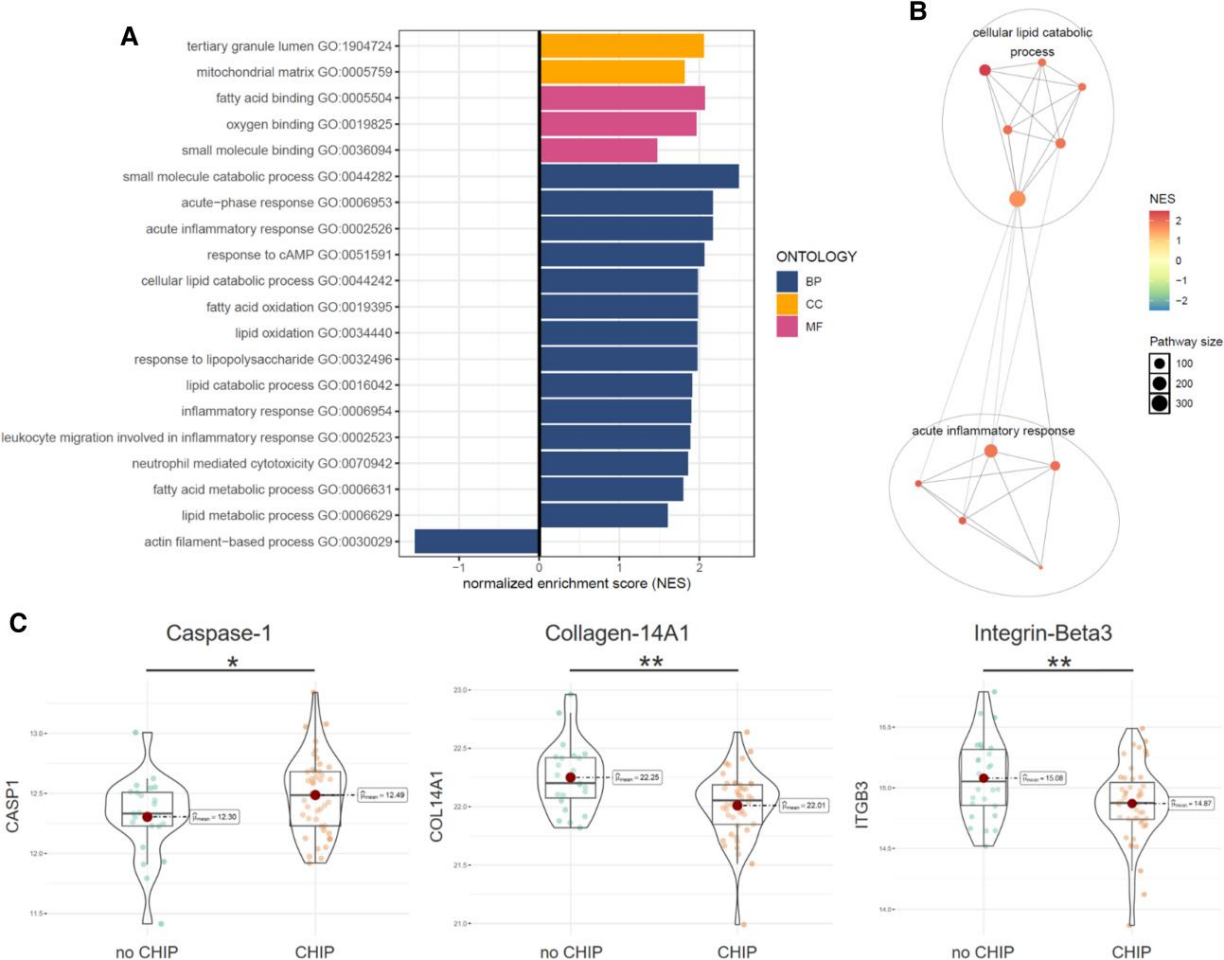
TET2 CHIP is associated with up-regulated inflammatory and metabolic pathways in human atherosclerotic plaques. (*A*) Gene set enrichment analysis highlighting the top 20 enriched gene ontologies for differentially expressed genes in *TET2* CHIP mutation carriers vs non-mutation controls. Bonferroni corrected *P* < .05 was considered statistically significant. (*B*) Inflammatory and lipid metabolism-related pathways showed dense interconnection in *TET2* CHIP mutation carriers. (*C*) differential expression of Caspase-1, Collagen-14A1, and Integrin-Beta3—linked to NLRP3 inflammasome activation, plaque stability, and atheroprotection. Boxplots display the interquartile range (IQR), the median (line), and whiskers extending to 1.5 × IQR; values beyond this range are shown as outliers. *P*-values were calculated using Welch’s *t*-test. The selected genes represent well-established markers of plaque stability. *P*-values are shown without Bonferroni correction for all mass-spec measured proteins (*n* = 5189) and reflect predefined testing across 52 plaque stability-related proteins. CHIP, clonal haematopoiesis of indeterminate potential; GO, Gene Ontology; BP, biological process; CC, cellular component; MF, molecular function; NES, normalized enrichment score. *P < .05; **P < .01

Gene set enrichment analysis of differentially expressed tissue proteins, measured using high-sensitivity mass spectrometry, revealed significant up-regulation of several interconnected pro-inflammatory (e.g. GO:0006953, *P* < .001; GO:0002526, *P* < .001; GO:0006954, *P* = .01; GO:0032496, *P* = .006; GO:0002523, *P* = .01; GO:0070942, *P* = .01) and lipid metabolism-related pathways (e.g. GO:0044242, *P* = .002; GO:0034440, *P* = .002 GO:0016042, *P* = .01; and GO:0006629, *P* = .03) in *TET2* CHIP mutation carriers. Caspase-1 (CASP1), the key enzyme in the NLRP3-inflammasome activating the pro-form of *IL1β* and *IL18* into the active cytokine, was up-regulated (*P* = .03). Whereas plaque stability markers like collagen-14A1 (COL14A1) (*P* = .005) or integrin-beta3 (ITGB3) (*P* = .002) were down-regulated in *TET2* mutation carriers (*Figure 3*).

Of note, 32 mitochondrial proteins were differentially expressed based on *TET2* CHIP status, revealing up-regulation of the mitochondrial pathways fatty acid oxidation and amino acid metabolism, indicating increased energy metabolism in human atherosclerotic plaques (see Supplementary data online, *Figure S5*). These findings indicate enhanced energy metabolism in human atherosclerotic plaques of *TET2* CHIP CAD carriers, potentially contributing to the more inflammatory and metabolically active plaque phenotype associated with this mutation.

### Intraplaque visualization of clonal haematopoiesis of indeterminate potential mutations (MISSION)

**Figure 4:**
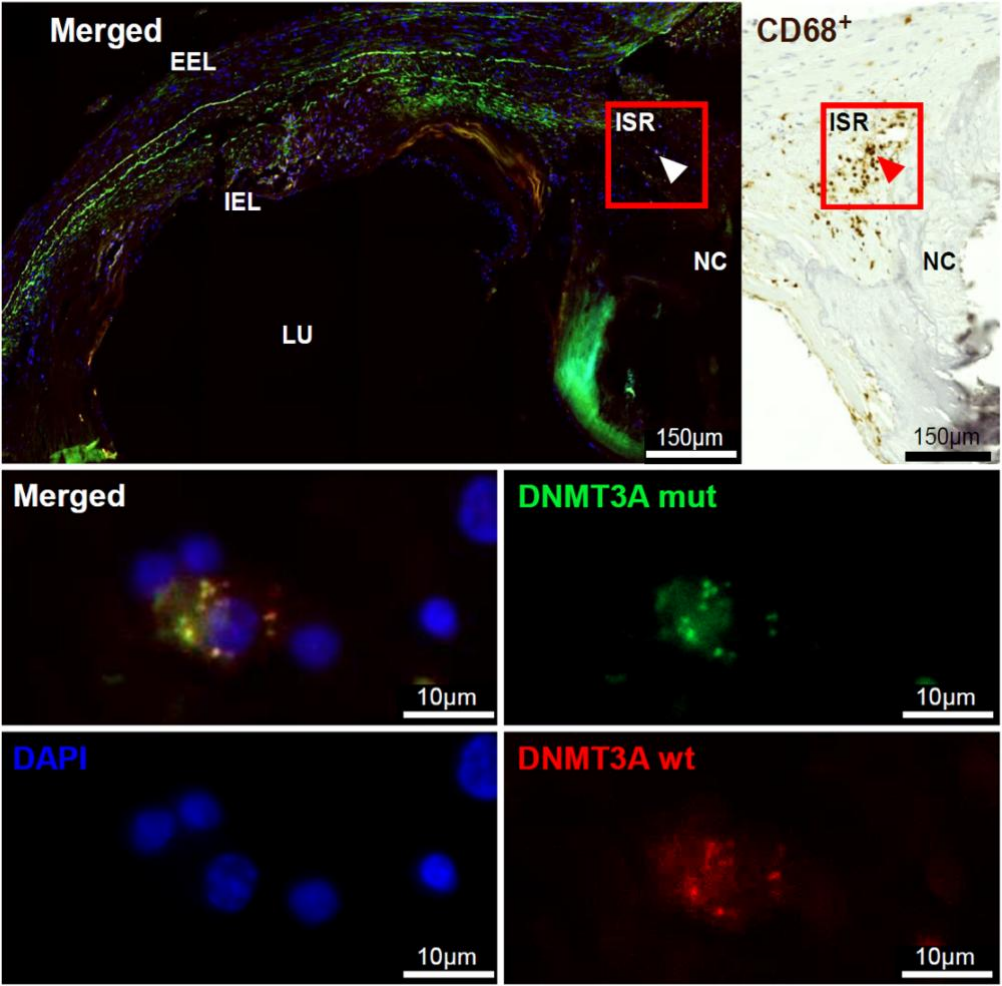
CHIP-mutated macrophage visualized in human atherosclerotic plaque. CHIP-mutated cells have the potential to invade from the peripheral blood into human atherosclerotic plaques. mutaFISH™ allowed the detection of single-nucleotide exchanges at RNA level in single cells. Staining for the specific *DNMT3A* CHIP mutation c.2333G>T (mut) and wild type (wt) was performed *in situ* in advanced atherosclerotic plaques of human FFPE artery tissue. Upper panel: Merged overview of an advanced atherosclerotic plaque. Red boxes highlight the inflammatory shoulder region of the plaque in two neighbouring slides stained for the CHIP mutation and the macrophage marker CD68. Cells of interest are highlighted (white and red arrows). Lower panel: The DNMTA3 CHIP mutation c.2333G>T was detected on single cell resolution via the green signal, the DNMT3A wild type via the red signal, and the cell nuclei (DAPI) via the blue signal. CHIP, clonal haematopoiesis of indeterminate potential; DAPI, 4′,6-diamidin-2-phenylindole; EEL, external elastic lamina; FFPE, formalin-fixed paraffin embedded; IEL, internal elastic lamina; LU, lumen; mutaFISH, mutation-specific fluorescence *in situ* hybridization

Clonal haematopoiesis of indeterminate potential-specific point mutations were visualized at the RNA level by custom-designed mutaFISH™ probes (Abnova, Taiwan). Based on high VAF and probe design feasibility, two distinct *DNMT3A* CHIP mutations (c.2245T>C and c.2333G>T) were targeted as representative examples. Consecutive staining for CD68 revealed that CHIP-affected leukocytes were macrophages (*Figure 4*). Although mutaFISH™ is not designed for quantification purposes, CHIP-mutated macrophages were identified in shoulder regions of advanced and inflammation-rich plaques of human coronary and carotid samples (see Supplementary data online, *Figure S6*). This qualitative finding complements our quantitative histological analyses, which demonstrated that *TET2* CHIP carriers exhibit increased inflammatory cell infiltration and features of plaque vulnerability.

### Clinical relevance of *TET2* mutations (STARNET)

Overall, 24 specific *TET2* CHIP mutations were identified in STARNET CAD patient WGS data. The corresponding *TET2* CHIP mutations were also identified on the RNA-seq level in monocyte-derived cultured macrophages of three individuals. Notably, cultures contained a mixture of macrophages with and without CHIP mutations. Macrophage RNA-seq data of *TET2* CHIP CAD mutation carriers (*n* = 3; c.6819G>T; c.6834C>T and c.7698T> ) were matched to CAD patients without CHIP mutation (*n* = 21) (see Supplementary data online, *Table S4*).

**Figure 5:**
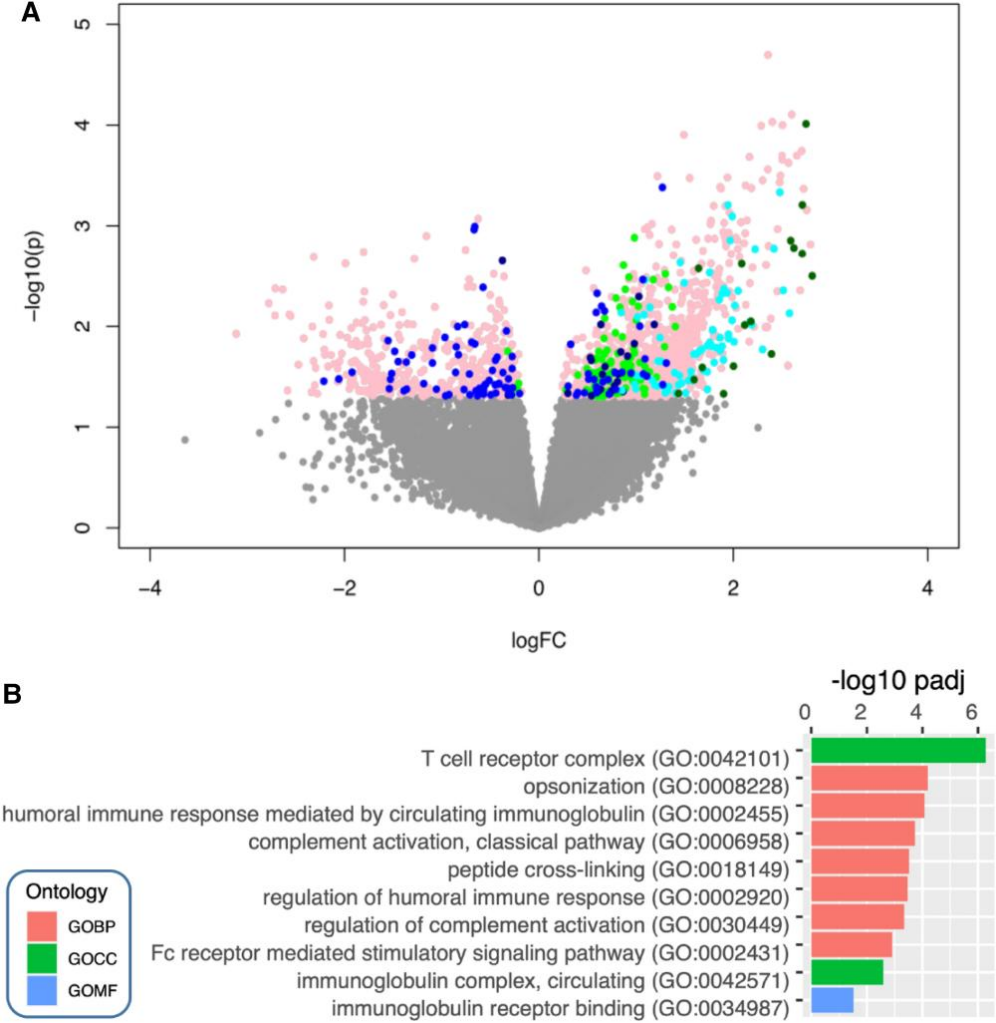
TET2 CHIP status is associated with CAD in STARNET. (*A*) Volcano plot of differentially expressed macrophage RNA-seq data between *TET2* CHIP mutation carriers (*n* = 3) vs age and sex matched non-mutation controls (*n* = 21) in STARNET CAD cases. Differentially expressed genes of significantly associated regulatory co-expression modules are highlighted in cyan, green, darkgreen, midnightblue, and blue, respectively. (*B*) Gene set enrichment analysis highlighting the top 10 enriched gene ontologies for differentially expressed genes in *TET2* CHIP mutation carriers vs non-mutation controls. Bonferroni corrected *P* < .05 was considered statistically significant. CHIP, clonal haematopoiesis of indeterminate potential; CAD, coronary artery disease; GO, Gene Ontology; BP, biological pathway; CC, cellular component; MF, molecular function

In total, 1523 genes were differentially expressed in macrophages of *TET2* mutation carriers compared with controls. 1098 genes were up-regulated and 425 genes were down-regulated. Gene set enrichment analysis based on GO revealed immune system and inflammation-associated pathways to be significantly up-regulated (*Figure 5*).

Independent of transcriptomic profiling, *TET2* CHIP status in STARNET was directly associated with greater CAD burden, including a higher number of atherosclerotic lesions (*P* < .001), increased CAD complexity (SYNTAX score; *P* < .001), and more affected coronary vessels (*P* < .001). In parallel, differential gene expression analysis of monocyte-derived macrophages from *TET2* CHIP carriers revealed significant up-regulation of immune and inflammatory pathways, providing mechanistic insight into these clinical associations.

### *TET2* deficiency promotes *LDLR* de-repression and enhances lipid uptake in macrophages *in vitro*

Proteomic profiling of atherosclerotic plaques from *TET2* CHIP carriers in the MISSION Biobank revealed enrichment of pathways related to lipid metabolism and inflammation, suggesting a mechanistic link between epigenetic dysregulation and accelerated atherosclerosis. To explore the functional relevance of *TET2* deficiency, we generated CRISPR/Cas9-edited *TET2^+/−^* monocytes and macrophages, modeling the heterozygous loss-of-function mutations observed in CHIP carriers.

**Figure 6:**
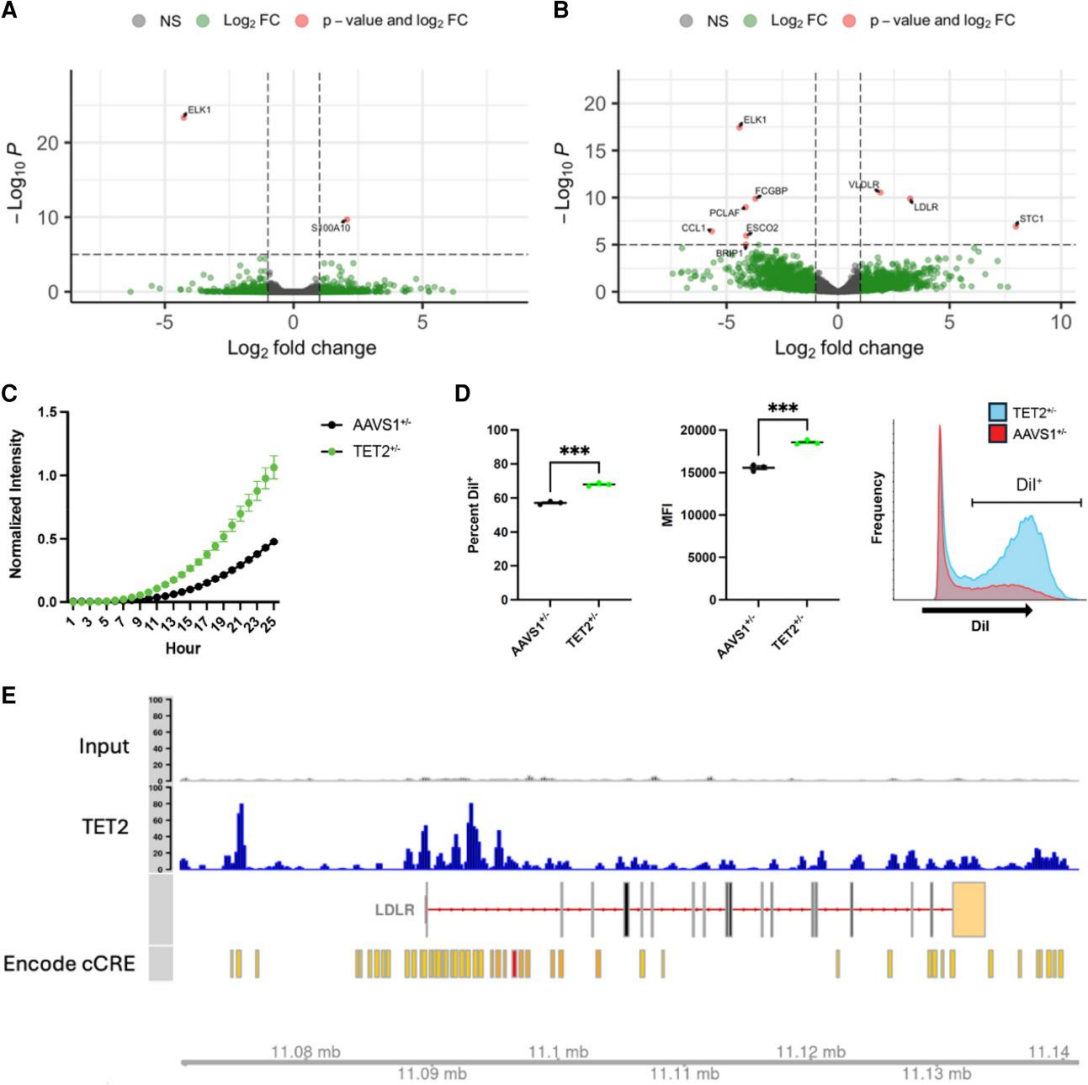
TET2 deficiency increases *LDLR* expression and enhances lipid uptake in macrophages *in vitro*. (*A*) Volcano plot of differentially expressed genes between *TET2^+/−^* THP-1 monocytes and controls. (*B*) Volcano plot of differentially expressed genes between *TET2^+/−^* THP-1 macrophages and controls after oxLDL stimulation. (*C*) pHrodo-LDL uptake in TET2^+/−^ THP-1 macrophages compared with controls. Circles represent the mean normalized fluorescence intensity at each time point for AAVS1^+/−^ (black) and TET2^+/−^ (green) macrophages, with error bars indicating the standard error of the mean (SEM). (*D*) fluorescent DiI-oxLDL uptake as measured by percent DiI-positive cells (left) and mean fluorescence intensity (MFI, middle). Presented are individual data points with mean values for AAVS1^+/−^ (black) and TET2^+/−^ (green) macrophages, with error bars showing SEM. The histogram (right) displays DiI fluorescence distribution, with TET2^+/−^ shown in blue fill and AAVS1^+/−^ in red outline; the DiI⁺ gate is indicated. (*E*) chromatin accessibility changes at the *LDLR* locus. Re-analysis of publicly available CHIP-seq data reveals enrichment of *TET2* binding near the *LDLR* promoter. ENCODE cCRE annotations (yellow/red) further indicate that these accessibility changes occur within regulatory regions, influencing *LDLR* regulation. ***P < .001

Bulk RNA sequencing of these *TET2^+/−^* macrophages confirmed significant down-regulation of *TET2* (*P* < .001) and up-regulation of *LDLR* expression (*P* < .001) compared with control macrophages after stimulation with oxLDL. Of note, this signature was not present in *TET2^+/−^* monocytes without lipid stimulation. Functionally, *TET2^+/−^* macrophages exhibited increased uptake of native LDL and oxLDL over 24 h, as demonstrated by elevated mean fluorescence intensity of fluorescently labeled oxLDL (*Figure 6*). This enhanced lipid uptake was accompanied by greater intracellular lipid accumulation, consistent with foam cell formation, a key feature of atherogenesis. These findings support a causal role for *TET2* deficiency in promoting a pro-atherogenic macrophage phenotype.

To explore the underlying regulatory mechanism, we analysed publicly available ENCODE epigenomic data, identifying candidate *cis*-regulatory elements (cCREs) within the *LDLR* locus.^66^ Chromatin accessibility analysis following *TET2* knockdown revealed increased accessibility at the *LDLR* locus, suggesting that *TET2* acts as a transcriptional repressor of *LDLR* (*Figure 6*). Loss of *TET2* may therefore lead to epigenetic de-repression of *LDLR*, enhancing lipid uptake in macrophages and contributing to plaque progression and instability in *TET2* CHIP carriers.

### Confirmation of *LDLR* dysregulation and pro-inflammatory signatures in human *TET2* clonal haematopoiesis of indeterminate potential plaques on RNA level

To validate our findings in an independent human cohort at the transcriptomic level, we analysed *TET2* CHIP-associated gene expression changes in carotid atherosclerotic plaques from the AE-Biobank. Clonal haematopoiesis of indeterminate potential carrier status was determined using the same sequencing methodology as in MISSION Biobank. Among 470 individuals with available plaque specimens, we identified 30 *TET2* CHIP carriers. RNA-seq profiles from *TET2* CHIP carriers were matched to 90 non-CHIP carriers. Baseline characteristics are provided in Supplementary data online, *Table S5*.

Bulk RNA sequencing confirmed reduced *TET2* expression (*P* = .04) and up-regulated *LDLR* expression (*P* = .02) in plaques from *TET2* CHIP carriers. Key NLRP3-inflammasome-associated inflammatory pathways were also dysregulated in *TET2* CHIP carriers, including up-regulation of *IL1R2* (*P* = .006) and *IL18RAP* (*P* < .001), which encode receptors for *IL1β* and *IL18*, respectively. Additionally, *IL1RN*, an anti-inflammatory cytokine that antagonizes *IL1α* and *IL1β* signaling, was down-regulated (*P* = .04), further supporting a pro-inflammatory macrophage phenotype.

Markers of plaque stability were altered, with *COL14A1* down-regulated (*P* = .002), aligning with our proteomic findings in MISSION Biobank, suggesting a weaker extracellular matrix structure in *TET2* CHIP carriers. Clinically, *TET2* mutation carriers in AE-Biobank had a two-fold higher prevalence of prior myocardial infarction or stroke compared with non-carriers (23.3% vs 11.1%; *P* = .13), and a two-fold higher risk of cardiovascular events during follow-up (40.0% vs 20.0%; *P* = .05) reinforcing the potential role of *TET2* CHIP in driving plaque vulnerability and adverse cardiovascular outcomes.

## Discussion

Our study on >8600 CAD patients demonstrates that CHIP is a frequent and clinically relevant phenomenon in CAD patients, with almost one-third being affected. Clonal haematopoiesis of indeterminate potential was associated with increased 3-year all-cause mortality risk, and this effect was consistent across multiple CHIP mutations, including *TET2*, *ASXL1*, *DNMT3A*, *JAK2, PPM1D*, *SF3B1*, *SRSF2*, and *U2AF1*, independent of traditional cardiovascular risk factors. Notably, patients with large clone sizes (VAF ≥ 10%) exhibited a markedly elevated mortality risk, suggesting that the burden of mutated cells is a determinant of clinical outcomes (*Structured Graphical Abstract*).

Using post-mortem samples from the MISSION Biobank, we confirmed the presence of CHIP-mutated leukocytes within atherosclerotic plaques. Clonal haematopoiesis of indeterminate potential-specific mutations was visualized at the RNA level using custom mutaFISH™ probes, identifying these cells as macrophages present in inflammatory shoulder regions of coronary and carotid plaques, supporting the hypothesis that CHIP contributes to plaque instability via macrophage-driven inflammation.^67^ These findings align with reports linking CHIP to increased monocyte-driven inflammation and tissue damage in other disease contexts, including acute kidney injury.^6^

In patients with *TET2* CHIP, proteomic profiling of atherosclerotic plaques revealed significant up-regulation of inflammatory pathways and metabolic processes, including elevated levels of *CASP1*, a key activator of the NLRP3 inflammasome, and concurrent down-regulation of markers associated with plaque stability.^68–72^ Transcriptomic analyses of monocyte-derived macrophages of CAD patients with *TET2* mutations (STARNET)^44,53,73^ revealed profound alterations in immune and inflammatory pathways, correlating with a more complex CAD phenotype.

To dissect the functional impact of *TET2* CHIP, *in-vitro* experiments using human monocyte THP-1 cells mimicking the human condition of *TET2* CHIP mutations demonstrated enhanced uptake of LDL and oxLDL, leading to increased intracellular lipid accumulation. This phenotype was associated with up-regulated cholesterol metabolism and pro-inflammatory gene expression, reinforcing the notion that *TET2* CHIP drives a pro-atherogenic macrophage phenotype. These findings align with recent reports that *TET2*-mutated macrophages exhibit excessive inflammatory activation, further contributing to atherosclerosis progression.^22^

Transcriptomic validation in an independent cohort confirmed reduced *TET2* expression, increased *LDLR* levels, and up-regulation of key inflammasome-related genes in *TET2* CHIP plaques, consistent with a pro-inflammatory macrophage phenotype. Down-regulation of extracellular matrix markers further suggested compromised plaque stability. Clinically, *TET2* CHIP carriers showed a trend toward higher cardiovascular risk, reinforcing the role of *TET2* CHIP in plaque vulnerability and adverse outcomes.

### Clonal haematopoiesis of indeterminate potential and cardiovascular therapy—a knowledge gap

The potential interaction of CHIP and cardiovascular therapies remains largely unexplored. The retrospective analysis of five TIMI trials (PEGASUS, ENGAGE, FOURIER, DECLARE, and SAVOR) assessed whether CHIP influences the efficacy of ticagrelor, edoxaban, evolocumab, dapagliflozin, or saxagliptin. While CHIP was associated with modestly increased cardiovascular risk, no significant interaction was observed between CHIP status and the efficacy of these therapies.^74^ Importantly, no anti-inflammatory trials were included, leaving the question of whether CHIP carriers might benefit from targeted anti-inflammatory interventions unanswered.

### Clinical implications and future directions

Given the strong pro-inflammatory and pro-atherogenic effects of *TET2* CHIP, anti-inflammatory therapy could hold promise for affected patients.^75^ Indeed, *IL1β* inhibitors or targeting the NLRP3 inflammasome axis may reduce plaque inflammation and improve plaque stability.^76–81^ Additionally, aggressive lipid-lowering strategies (high-intensity statins or PCSK9 inhibitors) may counteract the lipid metabolism disturbances in atherosclerotic plaques induced by CHIP. Personalized treatment approaches considering CHIP mutation status could therefore refine cardiovascular risk stratification and therapeutic strategies.

### Limitations

Despite its strengths, this study has inherent limitations that warrant consideration. The retrospective design inherently limits causal inference and introduces potential for unmeasured confounding. Although we adjusted for relevant clinical covariates and employed matching strategies, residual confounding due to unrecorded or unknown variables cannot be fully excluded. Moreover, HRs from Cox models may be affected by selection bias, particularly when event risk changes over time and more susceptible individuals experience early events. Additionally, the propensity score-matched analysis estimates the average treatment effect in the treated, reflecting the effect of CHIP in mutation carriers had they not carried the mutation. This limits generalizability to the broader CAD population, especially in the presence of effect heterogeneity across subgroups. Given its retrospective design, the precise duration and temporal dynamics of CHIP mutations in individual patients remain undetermined, limiting insights into their cumulative impact on disease progression. The sequencing depth used in STARNET may have resulted in undetected mutations, potentially underestimating the true CHIP burden and its contribution to cardiovascular pathology. Furthermore, while CHIP is conventionally defined by a VAF ≥ 2%, emerging evidence suggests that even lower VAFs in *DNMT3A* and *TET2* may be clinically relevant and associated with adverse cardiovascular outcomes.^82,83^ The implications of low-VAF CHIP require further investigation to refine risk stratification. Although *TET2* mutations were strongly linked to inflammation, lipid dysregulation, and atherogenesis, other CHIP mutations may exert distinct effects on leukocyte function and cardiovascular risk, necessitating a more comprehensive analysis of mutation-specific pathophysiology. Additionally, while MutaFISH™ successfully visualized CHIP-mutant macrophages within atherosclerotic plaques, it does not enable precise quantification of their burden. Future studies employing single-cell sequencing or ddPCR-based approaches will be essential to accurately determine the prevalence and functional impact of CHIP-mutated cells in the vascular wall. Moreover, while plaque analyses were standardized anatomically and matched for age and sex, we did not stratify molecular comparisons by plaque severity. As such, we cannot entirely exclude the possibility that some transcriptomic or proteomic differences may partially reflect variation in plaque stage. Future investigations using larger cohorts will be needed to enable stage-matched molecular comparisons that can disentangle CHIP-related biology from overall plaque burden. Finally, prospective studies with cause-specific adjudication and longer-term follow-up are needed to distinguish between competing causes of death in CHIP carriers. These limitations underscore the need for prospective validation, standardized sequencing methodologies, and targeted mechanistic studies to fully elucidate the role of CHIP in CVD and guide the development of precision-targeted interventions.

## Conclusions

Our study provides compelling evidence that CHIP is a significant acquired genetic factor influencing CAD severity and mortality. The presence of CHIP-mutated macrophages within atherosclerotic plaques is associated with increased inflammation and a higher burden of unstable lesions. In particular, *TET2* CHIP exacerbates CAD through dysregulated lipid metabolism and excessive inflammation, offering novel insights into disease mechanisms.

From a translational perspective, our findings highlight CHIP as a potential biomarker for risk stratification and suggest that targeting inflammatory and metabolic pathways in CHIP carriers could improve clinical outcomes. Future prospective trials should assess CHIP-specific therapeutic strategies to determine whether anti-inflammatory or lipid-lowering interventions can mitigate the heightened cardiovascular risk associated with CHIP.

## Supplementary Material

ehaf602_Supplementary_Data

## Declarations

### Data Availability

Data used in this study are available in permanent repositories. Human CAD patient data and data from MISSION can be requested by qualified researchers at the German Heart Center Munich from the corresponding authors. Human data from STARNET are accessible through the Database of Genotypes and Phenotypes (dbGAP). *In vitro* data of human monocyte THP-1 cell experiments can be requested by qualified researchers from the corresponding authors.
