# Supplementary material for: Clonal haematopoiesis of indeterminate potential and mortality in coronary artery disease
Source: Eur Heart J. 2025 Sep 3;47(4):453–69. doi: 10.1093/eurheartj/ehaf602 (PMC12831187; doi:10.1093/eurheartj/ehaf602)
Supplement: ehaf602_Supplementary_Data [file ehaf602_supplementary_data.pdf]

Supplement

Clonal Hematopoiesis of Indeterminate Potential and Mortality in Coronary Artery Disease

von Scheidt et al.

Content

Supplemental Table 1 – List of CHIP mutations in the clinical cohort (Munich)

Supplemental Table 2 – List of CHIP mutations in MISSION

Supplemental Table 3 – MISSION patient characteristics

Supplemental Table 4 – STARNET patient characteristics

Supplemental Table 5 – AtheroExpress patient characteristics

Supplemental Figure 1 – Distribution of CHIP mutations in the clinical cohort (Munich)

Supplemental Figure 2 – Large clone size and mortality risk in CAD patients

Supplemental Figure 3 – CHIP mutations in left ventricular myocardium, and atherosclerotic coronary and carotid samples

Supplemental Figure 4 – Histological assessment

Supplemental Figure 5 – Upregulation of mitochondrial pathways

Supplemental Figure 6 – mutaFISH^{TM} visualization of CHIP mutations in human atherosclerotic plaque

Protocol – adapted mutaFISH^{TM} protocol

Supplemental Table 1 – List of 3,507 unique CHIP mutations identified in the clinical cohort from Munich based on deep-DNA-sequencing. Provided are gene name, confirmation of CHIP mutation, change on DNA level, change on amino acid (AA) level and variant allele frequency (VAF) provided as percentages (%). Polymorphisms, variants, synonymous and uncertain mutations were excluded.

Gene	CHIP	DNA	AA	VAF
ASXL1	mutated	c.1175C>G	p.Ser392*	12.20
ASXL1	mutated	c.1178_1188del	p.Val393Alafs*13	2.52
ASXL1	mutated	c.1186C>T	p.Gln396*	8.30
ASXL1	mutated	c.1208dup	p.Arg404Alafs*6	2.54
ASXL1	mutated	c.1210C>T	p.Arg404*	3.21
ASXL1	mutated	c.1210C>T	p.Arg404*	4.13
ASXL1	mutated	c.1210C>T	p.Arg404*	5.60
ASXL1	mutated	c.1210C>T	p.Arg404*	6.60
ASXL1	mutated	c.1210C>T	p.Arg404*	6.80
ASXL1	mutated	c.1210C>T	p.Arg404*	18.90
ASXL1	mutated	c.1210C>T	p.Arg404*	25.50
ASXL1	mutated	c.1210C>T	p.Arg404*	27.20
ASXL1	mutated	c.1228A>T	p.Lys410*	3.35
ASXL1	mutated	c.1249C>T	p.Arg417*	3.18
ASXL1	mutated	c.1249C>T	p.Arg417*	5.30
ASXL1	mutated	c.1249C>T	p.Arg417*	6.70
ASXL1	mutated	c.1249C>T	p.Arg417*	16.80
ASXL1	mutated	c.1249C>T	p.Arg417*	29.50
ASXL1	mutated	c.1253_1319dup	p.Lys440Asnfs*20	2.25
ASXL1	mutated	c.1272_1273del	p.Tyr425Glnfs*12	10.30
ASXL1	mutated	c.1272_1273del	p.Tyr425Glnfs*12	14.70
ASXL1	mutated	c.1275_1276delinsG	p.Tyr425*	25.00
ASXL1	mutated	c.1281dup	p.Gln428Thrfs*10	2.00
ASXL1	mutated	c.1281dup	p.Gln428Thrfs*10	5.30
ASXL1	mutated	c.1285G>T	p.Glu429*	10.00
ASXL1	mutated	c.1289C>G	p.Ser430*	4.88
ASXL1	mutated	c.1291G>T	p.Glu431*	10.00
ASXL1	mutated	c.1291G>T	p.Glu431*	13.80
ASXL1	mutated	c.1294C>T	p.Gln432*	3.30
ASXL1	mutated	c.1294C>T	p.Gln432*	7.30
ASXL1	mutated	c.1331C>A	p.Ser444*	16.60
ASXL1	mutated	c.1373del	p.Pro458Glnfs*4	22.10
ASXL1	mutated	c.1388dup	p.Ser463Argfs*22	28.90
ASXL1	mutated	c.1423_1431delinsAGGTCCA	p.Asp475Argfs*9	8.10
ASXL1	mutated	c.1423_1431delinsAGGTCCA	p.Asp475Argfs*9	9.50
ASXL1	mutated	c.1438G>T	p.Glu480*	2.31
ASXL1	mutated	c.1442dup	p.Val483Serfs*2	3.44
ASXL1	mutated	c.1457_1458del	p.Val486Glyfs*7	2.19
ASXL1	mutated	c.1471C>T	p.Gln491*	3.21

ASXL1	mutated	c.1471C>T	p.Gln491*	20.80
ASXL1	mutated	c.1501_1531dup	p.Pro511Leufs*9	11.00
ASXL1	mutated	c.1516A>T	p.Arg506*	39.60
ASXL1	mutated	c.1517_1518del	p.Arg506Asnfs*3	20.50
ASXL1	mutated	c.1534C>T	p.Gln512*	2.12
ASXL1	mutated	c.1534C>T	p.Gln512*	2.58
ASXL1	mutated	c.1534C>T	p.Gln512*	3.18
ASXL1	mutated	c.1534C>T	p.Gln512*	6.00
ASXL1	mutated	c.1534C>T	p.Gln512*	9.30
ASXL1	mutated	c.1534C>T	p.Gln512*	14.40
ASXL1	mutated	c.1534C>T	p.Gln512*	26.20
ASXL1	mutated	c.1537G>T	p.Glu513*	17.40
ASXL1	mutated	c.1544_1545del	p.Val515Glyfs*13	12.70
ASXL1	mutated	c.1567A>T	p.Lys523*	2.22
ASXL1	mutated	c.1585C>T	p.Gln529*	5.60
ASXL1	mutated	c.1605dup	p.Pro536Serfs*8	3.15
ASXL1	mutated	c.1644dup	p.Arg549Serfs*2	3.88
ASXL1	mutated	c.1644dup	p.Arg549Serfs*2	10.70
ASXL1	mutated	c.1723C>T	p.Gln575*	3.12
ASXL1	mutated	c.1730C>A	p.Ser577*	17.80
ASXL1	mutated	c.1748G>A	p.Trp583*	4.85
ASXL1	mutated	c.1749_1753del	p.Trp583Cysfs*2	16.00
ASXL1	mutated	c.1758dup	p.Gly587Argfs*32	2.38
ASXL1	mutated	c.1762C>T	p.Gln588*	3.17
ASXL1	mutated	c.1762C>T	p.Gln588*	6.90
ASXL1	mutated	c.1762C>T	p.Gln588*	27.80
ASXL1	mutated	c.1770_1771del	p.Tyr591Profs*27	2.00
ASXL1	mutated	c.1771_1772insC	p.Tyr591Serfs*28	3.21
ASXL1	mutated	c.1772dup	p.Tyr591*	2.20
ASXL1	mutated	c.1772dup	p.Tyr591*	2.60
ASXL1	mutated	c.1772dup	p.Tyr591*	3.77
ASXL1	mutated	c.1772dup	p.Tyr591*	6.90
ASXL1	mutated	c.1772dup	p.Tyr591*	9.70
ASXL1	mutated	c.1772dup	p.Tyr591*	11.00
ASXL1	mutated	c.1772dup	p.Tyr591*	11.00
ASXL1	mutated	c.1772dup	p.Tyr591*	11.00
ASXL1	mutated	c.1772dup	p.Tyr591*	21.50
ASXL1	mutated	c.1772dup	p.Tyr591*	25.70
ASXL1	mutated	c.1772dup	p.Tyr591*	26.70
ASXL1	mutated	c.1773C>A	p.Tyr591*	2.09
ASXL1	mutated	c.1773C>A	p.Tyr591*	2.89
ASXL1	mutated	c.1773C>A	p.Tyr591*	26.40
ASXL1	mutated	c.1773C>G	p.Tyr591*	2.29
ASXL1	mutated	c.1773C>G	p.Tyr591*	2.83
ASXL1	mutated	c.1773C>G	p.Tyr591*	6.10
ASXL1	mutated	c.1773C>G	p.Tyr591*	11.20
ASXL1	mutated	c.1774C>T	p.Gln592*	2.14

ASXL1	mutated	c.1774C>T	p.Gln592*	36.30
ASXL1	mutated	c.1779dup	p.Cys594Metfs*25	3.06
ASXL1	mutated	c.1782C>A	p.Cys594*	4.40
ASXL1	mutated	c.1782C>A	p.Cys594*	5.90
ASXL1	mutated	c.1786dup	p.Arg596Profs*23	4.56
ASXL1	mutated	c.1803_1804delinsAT	p.Glu602*	5.20
ASXL1	mutated	c.1815C>A	p.Cys605*	32.40
ASXL1	mutated	c.1841_1878del	p.Leu614Argfs*8	4.22
ASXL1	mutated	c.1844_1881del	p.Ala615Glufs*7	17.00
ASXL1	mutated	c.1879dup	p.Ala627Glyfs*8	21.40
ASXL1	mutated	c.1884_1885del	p.Gly629Serfs*5	2.71
ASXL1	mutated	c.1900_1922del	p.Glu635Argfs*15	2.00
ASXL1	mutated	c.1900_1922del	p.Glu635Argfs*15	2.03
ASXL1	mutated	c.1900_1922del	p.Glu635Argfs*15	2.06
ASXL1	mutated	c.1900_1922del	p.Glu635Argfs*15	2.12
ASXL1	mutated	c.1900_1922del	p.Glu635Argfs*15	2.16
ASXL1	mutated	c.1900_1922del	p.Glu635Argfs*15	2.19
ASXL1	mutated	c.1900_1922del	p.Glu635Argfs*15	2.23
ASXL1	mutated	c.1900_1922del	p.Glu635Argfs*15	2.39
ASXL1	mutated	c.1900_1922del	p.Glu635Argfs*15	2.59
ASXL1	mutated	c.1900_1922del	p.Glu635Argfs*15	2.71
ASXL1	mutated	c.1900_1922del	p.Glu635Argfs*15	2.87
ASXL1	mutated	c.1900_1922del	p.Glu635Argfs*15	2.94
ASXL1	mutated	c.1900_1922del	p.Glu635Argfs*15	3.02
ASXL1	mutated	c.1900_1922del	p.Glu635Argfs*15	3.23
ASXL1	mutated	c.1900_1922del	p.Glu635Argfs*15	3.31
ASXL1	mutated	c.1900_1922del	p.Glu635Argfs*15	3.50
ASXL1	mutated	c.1900_1922del	p.Glu635Argfs*15	4.24
ASXL1	mutated	c.1900_1922del	p.Glu635Argfs*15	4.55
ASXL1	mutated	c.1900_1922del	p.Glu635Argfs*15	5.50
ASXL1	mutated	c.1900_1922del	p.Glu635Argfs*15	5.70
ASXL1	mutated	c.1900_1922del	p.Glu635Argfs*15	6.00
ASXL1	mutated	c.1900_1922del	p.Glu635Argfs*15	7.40
ASXL1	mutated	c.1900_1922del	p.Glu635Argfs*15	8.20
ASXL1	mutated	c.1900_1922del	p.Glu635Argfs*15	8.70
ASXL1	mutated	c.1900_1922del	p.Glu635Argfs*15	10.00
ASXL1	mutated	c.1900_1922del	p.Glu635Argfs*15	10.00
ASXL1	mutated	c.1900_1922del	p.Glu635Argfs*15	14.70
ASXL1	mutated	c.1900_1922del	p.Glu635Argfs*15	15.30
ASXL1	mutated	c.1900_1922del	p.Glu635Argfs*15	16.00
ASXL1	mutated	c.1900_1922del	p.Glu635Argfs*15	18.00
ASXL1	mutated	c.1900_1922del	p.Glu635Argfs*15	18.00
ASXL1	mutated	c.1900_1922del	p.Glu635Argfs*15	18.30
ASXL1	mutated	c.1900_1922del	p.Glu635Argfs*15	19.00
ASXL1	mutated	c.1900_1922del	p.Glu635Argfs*15	19.00
ASXL1	mutated	c.1900_1922del	p.Glu635Argfs*15	21.00
ASXL1	mutated	c.1900_1922del	p.Glu635Argfs*15	25.00

ASXL1	mutated	c.1900_1922del	p.Glu635Argfs*15	27.00
ASXL1	mutated	c.1900_1922del	p.Glu635Argfs*15	29.00
ASXL1	mutated	c.1900_1922del	p.Glu635Argfs*15	31.00
ASXL1	mutated	c.1900_1922del	p.Glu635Argfs*15	33.00
ASXL1	mutated	c.1900_1922del	p.Glu635Argfs*15	38.00
ASXL1	mutated	c.1902_1924del	p.Glu635Argfs*15	15.00
ASXL1	mutated	c.1924G>T	p.Gly642*	18.30
ASXL1	mutated	c.1926dup	p.Gly643Argfs*15	3.25
ASXL1	mutated	c.1927_1928insA	p.Gly643Glufs*15	30.00
ASXL1	mutated	c.1932_1933insC	p.Gly645Argfs*13	5.30
ASXL1	mutated	c.1933_1934insT	p.Gly645Valfs*13	14.30
ASXL1	mutated	c.1934dup	p.Gly646Trpfs*12	5.30
ASXL1	mutated	c.1934dup	p.Gly646Trpfs*12	5.30
ASXL1	mutated	c.1934dup	p.Gly646Trpfs*12	5.30
ASXL1	mutated	c.1934dup	p.Gly646Trpfs*12	5.30
ASXL1	mutated	c.1934dup	p.Gly646Trpfs*12	5.40
ASXL1	mutated	c.1934dup	p.Gly646Trpfs*12	5.50
ASXL1	mutated	c.1934dup	p.Gly646Trpfs*12	5.60
ASXL1	mutated	c.1934dup	p.Gly646Trpfs*12	5.80
ASXL1	mutated	c.1934dup	p.Gly646Trpfs*12	5.90
ASXL1	mutated	c.1934dup	p.Gly646Trpfs*12	5.90
ASXL1	mutated	c.1934dup	p.Gly646Trpfs*12	6.10
ASXL1	mutated	c.1934dup	p.Gly646Trpfs*12	6.30
ASXL1	mutated	c.1934dup	p.Gly646Trpfs*12	6.60
ASXL1	mutated	c.1934dup	p.Gly646Trpfs*12	6.70
ASXL1	mutated	c.1934dup	p.Gly646Trpfs*12	6.80
ASXL1	mutated	c.1934dup	p.Gly646Trpfs*12	6.90
ASXL1	mutated	c.1934dup	p.Gly646Trpfs*12	6.90
ASXL1	mutated	c.1934dup	p.Gly646Trpfs*12	7.20
ASXL1	mutated	c.1934dup	p.Gly646Trpfs*12	7.30
ASXL1	mutated	c.1934dup	p.Gly646Trpfs*12	7.60
ASXL1	mutated	c.1934dup	p.Gly646Trpfs*12	7.90
ASXL1	mutated	c.1934dup	p.Gly646Trpfs*12	8.10
ASXL1	mutated	c.1934dup	p.Gly646Trpfs*12	8.30
ASXL1	mutated	c.1934dup	p.Gly646Trpfs*12	8.30
ASXL1	mutated	c.1934dup	p.Gly646Trpfs*12	8.50
ASXL1	mutated	c.1934dup	p.Gly646Trpfs*12	8.70
ASXL1	mutated	c.1934dup	p.Gly646Trpfs*12	8.80
ASXL1	mutated	c.1934dup	p.Gly646Trpfs*12	10.00
ASXL1	mutated	c.1934dup	p.Gly646Trpfs*12	10.00
ASXL1	mutated	c.1934dup	p.Gly646Trpfs*12	10.30
ASXL1	mutated	c.1934dup	p.Gly646Trpfs*12	10.70
ASXL1	mutated	c.1934dup	p.Gly646Trpfs*12	10.80
ASXL1	mutated	c.1934dup	p.Gly646Trpfs*12	11.00
ASXL1	mutated	c.1934dup	p.Gly646Trpfs*12	11.50
ASXL1	mutated	c.1934dup	p.Gly646Trpfs*12	12.00
ASXL1	mutated	c.1934dup	p.Gly646Trpfs*12	12.40

ASXL1	mutated	c.1934dup	p.Gly646Trpfs*12	13.00
ASXL1	mutated	c.1934dup	p.Gly646Trpfs*12	14.00
ASXL1	mutated	c.1934dup	p.Gly646Trpfs*12	14.50
ASXL1	mutated	c.1934dup	p.Gly646Trpfs*12	14.60
ASXL1	mutated	c.1934dup	p.Gly646Trpfs*12	14.80
ASXL1	mutated	c.1934dup	p.Gly646Trpfs*12	15.10
ASXL1	mutated	c.1934dup	p.Gly646Trpfs*12	15.90
ASXL1	mutated	c.1934dup	p.Gly646Trpfs*12	16.30
ASXL1	mutated	c.1934dup	p.Gly646Trpfs*12	16.40
ASXL1	mutated	c.1934dup	p.Gly646Trpfs*12	17.30
ASXL1	mutated	c.1934dup	p.Gly646Trpfs*12	17.80
ASXL1	mutated	c.1934dup	p.Gly646Trpfs*12	17.80
ASXL1	mutated	c.1934dup	p.Gly646Trpfs*12	18.00
ASXL1	mutated	c.1934dup	p.Gly646Trpfs*12	18.20
ASXL1	mutated	c.1934dup	p.Gly646Trpfs*12	19.10
ASXL1	mutated	c.1934dup	p.Gly646Trpfs*12	19.10
ASXL1	mutated	c.1934dup	p.Gly646Trpfs*12	19.60
ASXL1	mutated	c.1934dup	p.Gly646Trpfs*12	20.60
ASXL1	mutated	c.1934dup	p.Gly646Trpfs*12	20.70
ASXL1	mutated	c.1934dup	p.Gly646Trpfs*12	20.90
ASXL1	mutated	c.1934dup	p.Gly646Trpfs*12	21.60
ASXL1	mutated	c.1934dup	p.Gly646Trpfs*12	21.90
ASXL1	mutated	c.1934dup	p.Gly646Trpfs*12	22.00
ASXL1	mutated	c.1934dup	p.Gly646Trpfs*12	22.30
ASXL1	mutated	c.1934dup	p.Gly646Trpfs*12	22.70
ASXL1	mutated	c.1934dup	p.Gly646Trpfs*12	23.00
ASXL1	mutated	c.1934dup	p.Gly646Trpfs*12	24.30
ASXL1	mutated	c.1934dup	p.Gly646Trpfs*12	24.70
ASXL1	mutated	c.1934dup	p.Gly646Trpfs*12	26.40
ASXL1	mutated	c.1972G>T	p.Gly658*	35.80
ASXL1	mutated	c.1986_1990del	p.Ser663Glnfs*3	2.68
ASXL1	mutated	c.2025_2041del	p.Pro677Hisfs*35	2.36
ASXL1	mutated	c.2025dup	p.Glu676*	4.32
ASXL1	mutated	c.2030_2038delinsT	p.Pro677Leufs*38	2.63
ASXL1	mutated	c.2060_2061del	p.Cys687Tyrfs*30	5.10
ASXL1	mutated	c.2061T>A	p.Cys687*	3.39
ASXL1	mutated	c.2074C>T	p.Gln692*	9.30
ASXL1	mutated	c.2077C>T	p.Arg693*	2.11
ASXL1	mutated	c.2077C>T	p.Arg693*	4.29
ASXL1	mutated	c.2077C>T	p.Arg693*	5.60
ASXL1	mutated	c.2077C>T	p.Arg693*	10.20
ASXL1	mutated	c.2077C>T	p.Arg693*	16.00
ASXL1	mutated	c.2077C>T	p.Arg693*	24.40
ASXL1	mutated	c.2077C>T	p.Arg693*	25.80
ASXL1	mutated	c.2083C>T	p.Gln695*	2.28
ASXL1	mutated	c.2083C>T	p.Gln695*	2.32
ASXL1	mutated	c.2100_2119del	p.Leu702Glyfs*9	22.00

ASXL1	mutated	c.2113del	p.Glu705Serfs*20	2.34
ASXL1	mutated	c.2113del	p.Glu705Serfs*20	2.43
ASXL1	mutated	c.2113G>T	p.Glu705*	27.60
ASXL1	mutated	c.2118dup	p.Thr707Tyrfs*11	10.70
ASXL1	mutated	c.2119dup	p.Thr707Asnfs*11	2.00
ASXL1	mutated	c.2122C>T	p.Gln708*	2.26
ASXL1	mutated	c.2122C>T	p.Gln708*	2.31
ASXL1	mutated	c.2125del	p.Ala709Profs*16	6.20
ASXL1	mutated	c.2128G>T	p.Gly710*	8.30
ASXL1	mutated	c.2128G>T	p.Gly710*	11.00
ASXL1	mutated	c.2154_2155insT	p.Glu719*	4.35
ASXL1	mutated	c.2167del	p.Ser723Leufs*2	15.70
ASXL1	mutated	c.2182G>T	p.Glu728*	3.92
ASXL1	mutated	c.2184_2185dup	p.Ser729Lysfs*16	2.00
ASXL1	mutated	c.2197C>T	p.Gln733*	3.19
ASXL1	mutated	c.2197C>T	p.Gln733*	37.80
ASXL1	mutated	c.2238del	p.Ser747Profs*25	12.50
ASXL1	mutated	c.2254dup	p.Ala752Glyfs*22	21.50
ASXL1	mutated	c.2266del	p.Asp756Thrfs*16	21.90
ASXL1	mutated	c.2269C>T	p.Gln757*	2.44
ASXL1	mutated	c.2269C>T	p.Gln757*	14.60
ASXL1	mutated	c.2285del	p.Leu762Cysfs*10	4.13
ASXL1	mutated	c.2290del	p.Leu764Tyrfs*8	14.00
ASXL1	mutated	c.2295_2308del	p.Gln768*	2.94
ASXL1	mutated	c.2302C>T	p.Gln768*	2.33
ASXL1	mutated	c.2317G>T	p.Glu773*	3.50
ASXL1	mutated	c.2322del	p.Arg774Serfs*2	2.71
ASXL1	mutated	c.2324T>G	p.Leu775*	5.40
ASXL1	mutated	c.2324T>G	p.Leu775*	8.10
ASXL1	mutated	c.2324T>G	p.Leu775*	9.60
ASXL1	mutated	c.2328_2329delinsTT	p.Glu777*	16.00
ASXL1	mutated	c.2337dup	p.Gln780Serfs*7	3.89
ASXL1	mutated	c.2356dup	p.Arg786Lysfs*3	30.70
ASXL1	mutated	c.2367_2368del	p.Cys789*	2.51
ASXL1	mutated	c.2367T>A	p.Cys789*	6.10
ASXL1	mutated	c.2376del	p.Thr793Profs*25	33.30
ASXL1	mutated	c.2385del	p.Trp796Glyfs*22	3.43
ASXL1	mutated	c.2385del	p.Trp796Glyfs*22	19.50
ASXL1	mutated	c.2389del	p.Glu797Lysfs*21	6.30
ASXL1	mutated	c.2394_2395del	p.Ser798Argfs*2	3.63
ASXL1	mutated	c.2404G>T	p.Glu802*	2.92
ASXL1	mutated	c.2407C>T	p.Gln803*	24.20
ASXL1	mutated	c.2455_2461del	p.Gly819Ilefs*3	2.65
ASXL1	mutated	c.2459_2460dup	p.Asp821Metfs*4	4.22
ASXL1	mutated	c.2464dup	p.Thr822Asnfs*11	2.11
ASXL1	mutated	c.2468T>A	p.Leu823*	2.69
ASXL1	mutated	c.2468T>A	p.Leu823*	3.83

ASXL1	mutated	c.2485C>T	p.Gln829*	2.94
ASXL1	mutated	c.2485C>T	p.Gln829*	10.70
ASXL1	mutated	c.2487_2488insTA	p.Ala830*	4.11
ASXL1	mutated	c.2525del	p.Asn842Metfs*2	2.91
ASXL1	mutated	c.2535del	p.Ser846Valfs*21	3.92
ASXL1	mutated	c.2535del	p.Ser846Valfs*21	4.29
ASXL1	mutated	c.2541del	p.Thr848Hisfs*19	10.00
ASXL1	mutated	c.2541del	p.Thr848Hisfs*19	12.60
ASXL1	mutated	c.2542dup	p.Thr848Asnfs*3	2.03
ASXL1	mutated	c.2543del	p.Thr848Asnfs*19	4.28
ASXL1	mutated	c.2555C>G	p.Ser852*	9.70
ASXL1	mutated	c.2563delinsAA	p.Asp855Lysfs*9	3.05
ASXL1	mutated	c.2568C>A	p.Cys856*	2.21
ASXL1	mutated	c.2571_2572delinsCT	p.Gln858*	3.43
ASXL1	mutated	c.2572C>T	p.Gln858*	2.82
ASXL1	mutated	c.2578A>T	p.Arg860*	12.40
ASXL1	mutated	c.2583dup	p.Phe862Ilefs*2	3.60
ASXL1	mutated	c.2588del	p.Asp863Valfs*4	3.26
ASXL1	mutated	c.2593G>T	p.Glu865*	35.10
ASXL1	mutated	c.2630_2633del	p.Glu877Valfs*8	2.20
ASXL1	mutated	c.2641_2680dup	p.Ser894Lysfs*13	12.00
ASXL1	mutated	c.2644C>T	p.Gln882*	2.88
ASXL1	mutated	c.2674_2692del	p.Ser892Glyfs*10	7.20
ASXL1	mutated	c.2693G>A	p.Trp898*	2.33
ASXL1	mutated	c.2705_2708dup	p.Asn904Ilefs*3	25.10
ASXL1	mutated	c.2708del	p.Ser903*	2.97
ASXL1	mutated	c.2749G>T	p.Glu917*	30.90
ASXL1	mutated	c.2757dup	p.Pro920Thrfs*4	5.40
ASXL1	mutated	c.2798del	p.Ala933Valfs*12	2.28
ASXL1	mutated	c.2873C>A	p.Ser958*	3.64
ASXL1	mutated	c.2874_2878del	p.Leu959Aspfs*9	2.33
ASXL1	mutated	c.2879G>A	p.Trp960*	25.60
ASXL1	mutated	c.2893C>T	p.Arg965*	2.00
ASXL1	mutated	c.2929C>T	p.Gln977*	5.40
ASXL1	mutated	c.2967_2968del	p.Glu990Serfs*7	5.60
ASXL1	mutated	c.3020del	p.Gly1007Valfs*17	5.00
ASXL1	mutated	c.3025_3029del	p.Leu1009Glyfs*5	7.30
ASXL1	mutated	c.3119_3167dup	p.Asp1057Cysfs*9	3.40
ASXL1	mutated	c.3177_3186del	p.Met1059Ilefs*47	6.00
ASXL1	mutated	c.3187C>T	p.Gln1063*	2.01
ASXL1	mutated	c.3202C>T	p.Arg1068*	12.50
ASXL1	mutated	c.3424C>T	p.Gln1142*	2.29
ASXL1	mutated	c.3424C>T	p.Gln1142*	4.23
ASXL1	mutated	c.3437C>A	p.Ser1146*	5.70
ASXL1	mutated	c.3514del	p.Ala1172Leufs*2	17.50
ASXL1	mutated	c.4127dup	p.Pro1377Serfs*3	2.79
ASXL1	mutated	c.4127dup	p.Pro1377Serfs*3	11.50

ASXL1	mutated	c.4232G>A	p.Trp1411*	2.73
ASXL1	mutated	c.4232G>A	p.Trp1411*	3.89
ASXL1	mutated	c.4234A>T	p.Lys1412*	3.05
ASXL1	mutated	c.4243C>T	p.Arg1415*	2.43
ASXL1	mutated	c.4243C>T	p.Arg1415*	3.26
ASXL1	mutated	c.4243C>T	p.Arg1415*	9.10
CALR	mutated	c.1099_1150del	p.Leu367Thrfs*46	3.10
CALR	mutated	c.1099_1150del	p.Leu367Thrfs*46	3.35
CALR	mutated	c.1099_1150del	p.Leu367Thrfs*46	17.00
CALR	mutated	c.1099_1150del	p.Leu367Thrfs*46	29.90
CALR	mutated	c.1099_1150del	p.Leu367Thrfs*46	39.20
CALR	mutated	c.1154_1155insTTGTC	p.Lys385Asnfs*47	6.50
CALR	mutated	c.1154_1155insTTGTC	p.Lys385Asnfs*47	11.30
CALR	mutated	c.1154_1155insTTGTC	p.Lys385Asnfs*47	15.90
CALR	mutated	c.1155_1156insTGTCG	p.Glu386Cysfs*46	39.10
CALR	mutated	c.1176T>G	p.Asp392Glu	9.90
CBL	mutated	c.1096G>A	p.Glu366Lys	7.90
CBL	mutated	c.1099C>A	p.Gln367Lys	8.20
CBL	mutated	c.1111T>A	p.Tyr371Asn	6.10
CBL	mutated	c.1136A>G	p.Gln379Arg	4.35
CBL	mutated	c.1139T>C	p.Leu380Pro	2.39
CBL	mutated	c.1139T>C	p.Leu380Pro	3.44
CBL	mutated	c.1139T>C	p.Leu380Pro	3.62
CBL	mutated	c.1139T>C	p.Leu380Pro	4.12
CBL	mutated	c.1139T>C	p.Leu380Pro	13.90
CBL	mutated	c.1142G>A	p.Cys381Tyr	3.52
CBL	mutated	c.1149A>G	p.Ile383Met	33.30
CBL	mutated	c.1150T>A	p.Cys384Ser	3.33
CBL	mutated	c.1150T>C	p.Cys384Arg	2.41
CBL	mutated	c.1150T>C	p.Cys384Arg	5.20
CBL	mutated	c.1150T>C	p.Cys384Arg	18.40
CBL	mutated	c.1151G>A	p.Cys384Tyr	2.12
CBL	mutated	c.1151G>A	p.Cys384Tyr	2.26
CBL	mutated	c.1151G>A	p.Cys384Tyr	2.83
CBL	mutated	c.1151G>A	p.Cys384Tyr	8.90
CBL	mutated	c.1151G>A	p.Cys384Tyr	12.20
CBL	mutated	c.1151G>T	p.Cys384Phe	4.13
CBL	mutated	c.1160A>T	p.Asn387Ile	2.02
CBL	mutated	c.1165A>G	p.Lys389Glu	3.27
CBL	mutated	c.1176G>T	p.Lys392Asn	4.71
CBL	mutated	c.1184C>T	p.Pro395Leu	3.19
CBL	mutated	c.1187G>A	p.Cys396Tyr	3.29
CBL	mutated	c.1187G>A	p.Cys396Tyr	31.70
CBL	mutated	c.1192C>T	p.His398Tyr	2.70
CBL	mutated	c.1192C>T	p.His398Tyr	25.40
CBL	mutated	c.1193A>C	p.His398Pro	5.20
CBL	mutated	c.1193A>G	p.His398Arg	2.45

CBL	mutated	c.1193A>G	p.His398Arg	5.70
CBL	mutated	c.1193A>G	p.His398Arg	6.80
CBL	mutated	c.1211G>A	p.Cys404Tyr	2.48
CBL	mutated	c.1211G>A	p.Cys404Tyr	3.16
CBL	mutated	c.1211G>A	p.Cys404Tyr	3.46
CBL	mutated	c.1211G>A	p.Cys404Tyr	3.73
CBL	mutated	c.1211G>A	p.Cys404Tyr	3.94
CBL	mutated	c.1211G>A	p.Cys404Tyr	5.30
CBL	mutated	c.1211G>A	p.Cys404Tyr	6.00
CBL	mutated	c.1211G>A	p.Cys404Tyr	28.00
CBL	mutated	c.1220C>T	p.Ser407Phe	5.10
CBL	mutated	c.1224G>T	p.Trp408Cys	6.20
CBL	mutated	c.1237G>A	p.Gly413Ser	3.17
CBL	mutated	c.1247G>C	p.Cys416Ser	2.99
CBL	mutated	c.1250C>T	p.Pro417Leu	2.03
CBL	mutated	c.1250C>T	p.Pro417Leu	33.80
CBL	mutated	c.1258C>G	p.Arg420Gly	2.00
CBL	mutated	c.1258C>G	p.Arg420Gly	5.00
CBL	mutated	c.1258C>T	p.Arg420*	44.80
CBL	mutated	c.1259G>A	p.Arg420Gln	6.50
CBL	mutated	c.1302T>A	p.Phe434Leu	3.27
CBL	mutated	c.1384C>T	p.Arg462*	8.40
CBL	mutated	c.1384C>T	p.Arg462*	41.20
CBL	mutated	c.1612_1615dup	p.Leu539Hisfs*47	4.02
CBL	mutated	c.2406G>A	p.Trp802*	5.20
CBL	mutated	c.2447G>A	p.Gly816Asp	3.03
CBL	mutated	c.421T>G	p.Tyr141Asp	16.50
CBL	mutated	c.446G>A	p.Arg149Gln	11.80
CBL	mutated	c.616C>T	p.Arg206*	5.30
DNMT3A	mutated	c.1005del	p.Lys335Asnfs*10	3.94
DNMT3A	mutated	c.1009T>C	p.Ser337Pro	9.50
DNMT3A	mutated	c.1010C>T	p.Ser337Leu	5.50
DNMT3A	mutated	c.1010C>T	p.Ser337Leu	16.20
DNMT3A	mutated	c.1010C>T	p.Ser337Leu	36.50
DNMT3A	mutated	c.1016T>A	p.Val339Glu	2.16
DNMT3A	mutated	c.1031T>A	p.Leu344Gln	8.50
DNMT3A	mutated	c.1031T>C	p.Leu344Pro	2.65
DNMT3A	mutated	c.1031T>C	p.Leu344Pro	3.03
DNMT3A	mutated	c.1031T>C	p.Leu344Pro	28.40
DNMT3A	mutated	c.1031T>G	p.Leu344Arg	2.60
DNMT3A	mutated	c.1040T>G	p.Leu347Arg	2.17
DNMT3A	mutated	c.1040T>G	p.Leu347Arg	14.30
DNMT3A	mutated	c.1051del	p.Cys351Alafs*56	2.03
DNMT3A	mutated	c.1051dup	p.Cys351Leufs*42	4.70
DNMT3A	mutated	c.1051dup	p.Cys351Leufs*42	5.20
DNMT3A	mutated	c.1054del	p.Ser352Valfs*55	2.29
DNMT3A	mutated	c.1056del	p.Ser352Argfs*55	8.20

DNMT3A	mutated	c.1061T>C	p.Phe354Ser	2.52
DNMT3A	mutated	c.1061T>C	p.Phe354Ser	7.50
DNMT3A	mutated	c.1061T>C	p.Phe354Ser	10.20
DNMT3A	mutated	c.1063del	p.His355Thrfs*52	2.46
DNMT3A	mutated	c.1066C>A	p.Gln356Lys	10.30
DNMT3A	mutated	c.1066C>T	p.Gln356*	2.17
DNMT3A	mutated	c.1066C>T	p.Gln356*	6.60
DNMT3A	mutated	c.1076A>G	p.Tyr359Cys	3.96
DNMT3A	mutated	c.1077_1078delinsAC	p.Tyr359_Asn360delins*	7.70
DNMT3A	mutated	c.1077C>A	p.Tyr359*	2.68
DNMT3A	mutated	c.1077C>A	p.Tyr359*	5.80
DNMT3A	mutated	c.1084del	p.Gln362Serfs*45	3.22
DNMT3A	mutated	c.1093T>G	p.Tyr365Asp	2.44
DNMT3A	mutated	c.1093T>G	p.Tyr365Asp	11.30
DNMT3A	mutated	c.1094A>G	p.Tyr365Cys	2.39
DNMT3A	mutated	c.1094A>G	p.Tyr365Cys	2.97
DNMT3A	mutated	c.1096C>A	p.Arg366Ser	2.17
DNMT3A	mutated	c.1097G>A	p.Arg366His	5.40
DNMT3A	mutated	c.1102G>A	p.Ala368Thr	5.00
DNMT3A	mutated	c.1103C>A	p.Ala368Asp	8.70
DNMT3A	mutated	c.1103C>T	p.Ala368Val	13.90
DNMT3A	mutated	c.1104delC	p.Ile369Serfs*38	2.42
DNMT3A	mutated	c.1107del	p.Tyr370Thrfs*37	2.87
DNMT3A	mutated	c.1110C>G	p.Tyr370*	5.60
DNMT3A	mutated	c.1111G>T	p.Glu371*	7.40
DNMT3A	mutated	c.1118T>C	p.Leu373Pro	5.40
DNMT3A	mutated	c.1120C>T	p.Gln374*	2.48
DNMT3A	mutated	c.1120C>T	p.Gln374*	9.70
DNMT3A	mutated	c.1123-1_1127del	p.splice site mutation	8.80
DNMT3A	mutated	c.1123-9_1123del	p.splice site mutation	2.52
DNMT3A	mutated	c.112C>T	p.Arg38Cys	41.90
DNMT3A	mutated	c.1135C>T	p.Arg379Cys	2.11
DNMT3A	mutated	c.1135C>T	p.Arg379Cys	4.02
DNMT3A	mutated	c.1135C>T	p.Arg379Cys	40.10
DNMT3A	mutated	c.1136G>A	p.Arg379His	2.86
DNMT3A	mutated	c.1136G>A	p.Arg379His	3.14
DNMT3A	mutated	c.1136G>A	p.Arg379His	24.30
DNMT3A	mutated	c.1144A>T	p.Lys382*	7.30
DNMT3A	mutated	c.1154del	p.Pro385Argfs*22	3.73
DNMT3A	mutated	c.1154del	p.Pro385Argfs*22	6.50
DNMT3A	mutated	c.1159_1160del	p.Cys387Profs*5	16.80
DNMT3A	mutated	c.1174G>T	p.Glu392*	2.79
DNMT3A	mutated	c.1175dup	p.Ser393Glufs*2	2.36
DNMT3A	mutated	c.1179dup	p.Asp394*	4.91
DNMT3A	mutated	c.1195G>T	p.Val399Leu	2.85
DNMT3A	mutated	c.1215dup	p.Met406Hisfs*3	2.58
DNMT3A	mutated	c.1220_1234del	p.Ile407_Gly412delinsArg	2.90

DNMT3A	mutated	c.1220T>C	p.Ile407Thr	2.51
DNMT3A	mutated	c.1220T>C	p.Ile407Thr	3.25
DNMT3A	mutated	c.1225del	p.Trp409Glyfs*242	29.60
DNMT3A	mutated	c.1226G>A	p.Trp409*	6.50
DNMT3A	mutated	c.1227G>A	p.Trp409*	11.30
DNMT3A	mutated	c.1227G>C	p.Trp409Cys	5.60
DNMT3A	mutated	c.1228G>A	p.Ala410Thr	4.80
DNMT3A	mutated	c.1228G>A	p.Ala410Thr	5.30
DNMT3A	mutated	c.1228G>A	p.Ala410Thr	11.40
DNMT3A	mutated	c.1229C>T	p.Ala410Val	3.35
DNMT3A	mutated	c.1238_1247del	p.Gly413Valfs*235	2.00
DNMT3A	mutated	c.1238del	p.Gly413Alafs*238	3.28
DNMT3A	mutated	c.1238del	p.Gly413Alafs*238	6.80
DNMT3A	mutated	c.1238del	p.Gly413Alafs*238	6.90
DNMT3A	mutated	c.1238dup	p.Phe414Leufs*7	22.20
DNMT3A	mutated	c.1240T>C	p.Phe414Leu	16.10
DNMT3A	mutated	c.1240T>G	p.Phe414Val	4.47
DNMT3A	mutated	c.1241T>C	p.Phe414Ser	9.70
DNMT3A	mutated	c.1243_1250del	p.Gln415Trpfs*3	5.40
DNMT3A	mutated	c.1243C>T	p.Gln415*	2.64
DNMT3A	mutated	c.1243C>T	p.Gln415*	11.00
DNMT3A	mutated	c.1250del	p.Ser417Leufs*234	2.51
DNMT3A	mutated	c.1252_1253delinsTT	p.Gly418Phe	3.25
DNMT3A	mutated	c.1252_1258del	p.Gly418Argfs*231	4.42
DNMT3A	mutated	c.1253del	p.Gly418Alafs*233	4.66
DNMT3A	mutated	c.1263_1264del	p.Leu422Glyfs*22	24.60
DNMT3A	mutated	c.1263_1266delinsTA	p.Leu422Argfs*22	33.00
DNMT3A	mutated	c.1276G>T	p.Glu426*	19.30
DNMT3A	mutated	c.1279G>A	p.Glu427Lys	2.99
DNMT3A	mutated	c.1292C>A	p.Pro431His	2.15
DNMT3A	mutated	c.1293del	p.Tyr432Thrfs*219	2.80
DNMT3A	mutated	c.1297A>T	p.Lys433*	2.38
DNMT3A	mutated	c.1300G>T	p.Glu434*	2.27
DNMT3A	mutated	c.1300G>T	p.Glu434*	38.60
DNMT3A	mutated	c.1308C>G	p.Tyr436*	11.00
DNMT3A	mutated	c.1319G>A	p.Trp440*	2.20
DNMT3A	mutated	c.1319G>A	p.Trp440*	9.90
DNMT3A	mutated	c.1320G>A	p.Trp440*	10.90
DNMT3A	mutated	c.1321del	p.Val441Trpfs*210	3.12
DNMT3A	mutated	c.1324del	p.Glu442Asnfs*209	12.00
DNMT3A	mutated	c.1331del	p.Glu444Glyfs*207	9.10
DNMT3A	mutated	c.1346_1367delinsTG	p.Ala449Valfs*17	3.16
DNMT3A	mutated	c.1360del	p.Ala454Profs*197	2.20
DNMT3A	mutated	c.1363A>T	p.Lys455*	2.32
DNMT3A	mutated	c.1368del	p.Lys456Asnfs*195	3.27
DNMT3A	mutated	c.1377del	p.Ser460Alafs*191	13.20
DNMT3A	mutated	c.1385del	p.Ala462Glyfs*189	3.85

DNMT3A	mutated	c.1386_1387insA	p.Glu463Argfs*10	3.79
DNMT3A	mutated	c.1387G>T	p.Glu463*	8.20
DNMT3A	mutated	c.1399del	p.Val467Serfs*184	2.51
DNMT3A	mutated	c.1401del	p.Lys468Argfs*183	3.91
DNMT3A	mutated	c.1403A>G	p.Lys468Arg	2.78
DNMT3A	mutated	c.1403A>G	p.Lys468Arg	6.50
DNMT3A	mutated	c.1406A>T	p.Glu469Val	8.90
DNMT3A	mutated	c.1410_1416del	p.Ile470Metfs*179	2.13
DNMT3A	mutated	c.1416_1417insA	p.Glu473Argfs*19	7.60
DNMT3A	mutated	c.1417G>T	p.Glu473*	2.13
DNMT3A	mutated	c.1417G>T	p.Glu473*	5.00
DNMT3A	mutated	c.1423_1424delinsTG	p.Thr475*	2.24
DNMT3A	mutated	c.1425_1426del	p.Glu477Alafs*14	2.07
DNMT3A	mutated	c.1428del	p.Glu477Serfs*174	8.20
DNMT3A	mutated	c.1429G>A	p.Glu477Lys	6.00
DNMT3A	mutated	c.1430-19_1433delinsT	p.splice site mutation	9.10
DNMT3A	mutated	c.1432C>A	p.Arg478Arg	44.00
DNMT3A	mutated	c.1438del	p.Val480Cysfs*171	2.32
DNMT3A	mutated	c.1447del	p.Val483Cysfs*168	2.70
DNMT3A	mutated	c.1464del	p.Asn489Thrfs*162	13.00
DNMT3A	mutated	c.1470del	p.Ile490Metfs*161	7.00
DNMT3A	mutated	c.1472_1474+1del	p.splice site mutation	2.20
DNMT3A	mutated	c.1479dup	p.Cys494Leufs*52	4.21
DNMT3A	mutated	c.1480T>G	p.Cys494Gly	2.59
DNMT3A	mutated	c.1481G>T	p.Cys494Phe	2.76
DNMT3A	mutated	c.1481G>T	p.Cys494Phe	2.78
DNMT3A	mutated	c.1482C>G	p.Cys494Trp	3.04
DNMT3A	mutated	c.1490G>A	p.Cys497Tyr	8.20
DNMT3A	mutated	c.1490G>A	p.Cys497Tyr	14.60
DNMT3A	mutated	c.1490G>T	p.Cys497Phe	5.60
DNMT3A	mutated	c.1494del	p.Ser499Alafs*152	22.30
DNMT3A	mutated	c.1498del	p.Leu500Serfs*151	14.00
DNMT3A	mutated	c.1500_1501dup	p.Asn501Thrfs*151	8.90
DNMT3A	mutated	c.1506_1511del	p.Thr503_Leu504del	5.30
DNMT3A	mutated	c.1507dup	p.Thr503Asnfs*43	7.70
DNMT3A	mutated	c.1510del	p.Leu504Trpfs*147	4.52
DNMT3A	mutated	c.1510del	p.Leu504Trpfs*147	6.70
DNMT3A	mutated	c.1510del	p.Leu504Trpfs*147	21.10
DNMT3A	mutated	c.1511del	p.Leu504Argfs*147	14.00
DNMT3A	mutated	c.1513del	p.Glu505Asnfs*146	6.80
DNMT3A	mutated	c.1517A>G	p.His506Arg	3.02
DNMT3A	mutated	c.1520C>G	p.Pro507Arg	3.56
DNMT3A	mutated	c.1522dup	p.Leu508Profs*38	5.70
DNMT3A	mutated	c.1523T>C	p.Leu508Pro	6.50
DNMT3A	mutated	c.1531G>A	p.Gly511Arg	3.91
DNMT3A	mutated	c.1531G>C	p.Gly511Arg	4.32
DNMT3A	mutated	c.1535G>A	p.Gly512Glu	2.00

DNMT3A	mutated	c.1537dup	p.Met513Asnfs*33	6.50
DNMT3A	mutated	c.1540del	p.Cys514Alafs*137	5.50
DNMT3A	mutated	c.1540T>C	p.Cys514Arg	3.39
DNMT3A	mutated	c.1541G>A	p.Cys514Tyr	8.20
DNMT3A	mutated	c.1550G>A	p.Cys517Tyr	6.40
DNMT3A	mutated	c.1551C>G	p.Cys517Trp	2.90
DNMT3A	mutated	c.1551C>G	p.Cys517Trp	3.57
DNMT3A	mutated	c.1559del	p.Cys520Serfs*131	9.60
DNMT3A	mutated	c.1559del	p.Cys520Serfs*131	29.30
DNMT3A	mutated	c.1560C>A	p.Cys520*	4.84
DNMT3A	mutated	c.1560C>A	p.Cys520*	36.60
DNMT3A	mutated	c.1567G>T	p.Glu523*	4.80
DNMT3A	mutated	c.1567G>T	p.Glu523*	6.10
DNMT3A	mutated	c.1579C>T	p.Gln527*	2.10
DNMT3A	mutated	c.1579C>T	p.Gln527*	6.10
DNMT3A	mutated	c.1579C>T	p.Gln527*	10.40
DNMT3A	mutated	c.1579C>T	p.Gln527*	10.60
DNMT3A	mutated	c.1581G>C	p.Gln527His	2.83
DNMT3A	mutated	c.1583_1603dup	p.Tyr528_Gln534dup	6.00
DNMT3A	mutated	c.1583_1603dup	p.Tyr528_Gln534dup	25.00
DNMT3A	mutated	c.1584del	p.Tyr528*	2.61
DNMT3A	mutated	c.1585G>A	p.Asp529Asn	3.37
DNMT3A	mutated	c.1585G>A	p.Asp529Asn	14.20
DNMT3A	mutated	c.1591G>A	p.Asp531Asn	2.33
DNMT3A	mutated	c.1591G>A	p.Asp531Asn	2.45
DNMT3A	mutated	c.1592A>G	p.Asp531Gly	3.09
DNMT3A	mutated	c.1592A>T	p.Asp531Val	4.03
DNMT3A	mutated	c.1594G>A	p.Gly532Ser	29.40
DNMT3A	mutated	c.1598A>G	p.Tyr533Cys	3.16
DNMT3A	mutated	c.1598A>G	p.Tyr533Cys	4.74
DNMT3A	mutated	c.1599C>G	p.Tyr533*	2.02
DNMT3A	mutated	c.1600C>T	p.Gln534*	2.87
DNMT3A	mutated	c.1600del	p.Gln534Serfs*117	2.12
DNMT3A	mutated	c.1602G>T	p.Gln534His	3.61
DNMT3A	mutated	c.1605del	p.Tyr536Thrfs*115	6.70
DNMT3A	mutated	c.1608_1610delinsTA	p.Cys537Thrfs*114	3.85
DNMT3A	mutated	c.1609T>A	p.Cys537Ser	4.50
DNMT3A	mutated	c.1611C>G	p.Cys537Trp	4.13
DNMT3A	mutated	c.1616_1617delinsAG	p.Ile539Lys	8.40
DNMT3A	mutated	c.1627G>A	p.Gly543Ser	36.90
DNMT3A	mutated	c.1628G>A	p.Gly543Asp	3.08
DNMT3A	mutated	c.1628G>T	p.Gly543Val	2.00
DNMT3A	mutated	c.1628G>T	p.Gly543Val	4.38
DNMT3A	mutated	c.1633G>T	p.Glu545*	15.40
DNMT3A	mutated	c.163C>T	p.Arg55Cys	3.78
DNMT3A	mutated	c.1642_1643del	p.Met548Valfs*29	2.18
DNMT3A	mutated	c.1642_1643del	p.Met548Valfs*29	5.90

DNMT3A	mutated	c.1642dup	p.Met548Asnfs*30	2.69
DNMT3A	mutated	c.1643_1644delinsAT	p.Met548Asn	11.80
DNMT3A	mutated	c.1643T>C	p.Met548Thr	2.04
DNMT3A	mutated	c.1643T>C	p.Met548Thr	4.48
DNMT3A	mutated	c.1643T>C	p.Met548Thr	16.00
DNMT3A	mutated	c.1643T>C	p.Met548Thr	22.70
DNMT3A	mutated	c.1644G>A	p.Met548Ile	12.00
DNMT3A	mutated	c.1647C>A	p.Cys549*	2.19
DNMT3A	mutated	c.1647C>T	p.Cys549Cys	47.50
DNMT3A	mutated	c.1648G>A	p.Gly550Arg	2.78
DNMT3A	mutated	c.1648G>A	p.Gly550Arg	3.16
DNMT3A	mutated	c.1648G>A	p.Gly550Arg	5.10
DNMT3A	mutated	c.1655dup	p.Asn552Lysfs*26	8.10
DNMT3A	mutated	c.1656del	p.Asn552Lysfs*99	2.76
DNMT3A	mutated	c.1663T>G	p.Cys555Gly	8.20
DNMT3A	mutated	c.1666A>G	p.Arg556Gly	2.32
DNMT3A	mutated	c.1666A>T	p.Arg556Trp	26.00
DNMT3A	mutated	c.1668-5_1686del	p.splice site mutation	7.80
DNMT3A	mutated	c.1668G>C	p.Arg556Ser	3.17
DNMT3A	mutated	c.1668G>T	p.Arg556Ser	20.30
DNMT3A	mutated	c.1669T>C	p.Cys557Arg	3.00
DNMT3A	mutated	c.1670G>A	p.Cys557Tyr	2.74
DNMT3A	mutated	c.1670G>A	p.Cys557Tyr	5.80
DNMT3A	mutated	c.1671_1680del	p.Cys557Trpfs*91	3.64
DNMT3A	mutated	c.1671C>A	p.Cys557*	5.90
DNMT3A	mutated	c.1671C>G	p.Cys557Trp	2.32
DNMT3A	mutated	c.1673T>G	p.Phe558Cys	2.80
DNMT3A	mutated	c.1674_1713del	p.Phe558Leufs*80	11.00
DNMT3A	mutated	c.1675del	p.Cys559Alafs*92	2.50
DNMT3A	mutated	c.1675del	p.Cys559Alafs*92	3.36
DNMT3A	mutated	c.1675del	p.Cys559Alafs*92	17.40
DNMT3A	mutated	c.1677C>A	p.Cys559*	2.16
DNMT3A	mutated	c.1677C>A	p.Cys559*	2.37
DNMT3A	mutated	c.1678del	p.Val560Trpfs*91	32.90
DNMT3A	mutated	c.1685del	p.Cys562Leufs*89	23.00
DNMT3A	mutated	c.1685G>A	p.Cys562Tyr	2.52
DNMT3A	mutated	c.1685G>A	p.Cys562Tyr	3.11
DNMT3A	mutated	c.1685G>A	p.Cys562Tyr	3.19
DNMT3A	mutated	c.1685G>A	p.Cys562Tyr	3.25
DNMT3A	mutated	c.1685G>A	p.Cys562Tyr	4.52
DNMT3A	mutated	c.1685G>A	p.Cys562Tyr	6.80
DNMT3A	mutated	c.1685G>A	p.Cys562Tyr	11.20
DNMT3A	mutated	c.1687G>A	p.Val563Met	2.37
DNMT3A	mutated	c.1687G>A	p.Val563Met	29.90
DNMT3A	mutated	c.1688_1689del	p.Val563Glyfs*14	4.44
DNMT3A	mutated	c.1688_1689del	p.Val563Glyfs*14	5.80
DNMT3A	mutated	c.1694T>C	p.Leu565Pro	2.92

DNMT3A	mutated	c.1697dup	p.Leu566Phefs*12	13.50
DNMT3A	mutated	c.1697T>A	p.Leu566*	11.10
DNMT3A	mutated	c.1700_1703del	p.Val567Glyfs*83	15.00
DNMT3A	mutated	c.1704del	p.Pro569Argfs*82	4.29
DNMT3A	mutated	c.1709G>A	p.Gly570Glu	2.33
DNMT3A	mutated	c.1711del	p.Ala571Leufs*80	2.92
DNMT3A	mutated	c.1715C>A	p.Ala572Asp	4.40
DNMT3A	mutated	c.1717del	p.Gln573Argfs*78	10.70
DNMT3A	mutated	c.1720G>C	p.Ala574Pro	27.60
DNMT3A	mutated	c.1727T>A	p.Ile576Asn	2.70
DNMT3A	mutated	c.1735dup	p.Asp579Glyfs*33	13.30
DNMT3A	mutated	c.1736A>G	p.Asp579Gly	2.03
DNMT3A	mutated	c.1736del	p.Asp579Alafs*72	11.00
DNMT3A	mutated	c.1738_1746del	p.Pro580_Asn582del	2.07
DNMT3A	mutated	c.1740del	p.Trp581Glyfs*70	5.30
DNMT3A	mutated	c.1740del	p.Trp581Glyfs*70	16.70
DNMT3A	mutated	c.1741T>A	p.Trp581Arg	4.23
DNMT3A	mutated	c.1741T>A	p.Trp581Arg	5.00
DNMT3A	mutated	c.1742_1743delinsTC	p.Trp581Phe	17.00
DNMT3A	mutated	c.1742G>A	p.Trp581*	2.08
DNMT3A	mutated	c.1742G>A	p.Trp581*	2.26
DNMT3A	mutated	c.1742G>A	p.Trp581*	3.26
DNMT3A	mutated	c.1742G>A	p.Trp581*	8.80
DNMT3A	mutated	c.1743G>A	p.Trp581*	2.58
DNMT3A	mutated	c.1743G>A	p.Trp581*	6.40
DNMT3A	mutated	c.1743G>C	p.Trp581Cys	5.50
DNMT3A	mutated	c.1743G>C	p.Trp581Cys	6.00
DNMT3A	mutated	c.1747_1750delinsGGCC	p.Cys583_Tyr584delinsGlyHis	7.70
DNMT3A	mutated	c.1747T>A	p.Cys583Ser	2.22
DNMT3A	mutated	c.1749C>A	p.Cys583*	5.70
DNMT3A	mutated	c.1749C>A	p.Cys583*	8.10
DNMT3A	mutated	c.1753_1772del	p.Met585Leufs*20	3.22
DNMT3A	mutated	c.1757G>A	p.Cys586Tyr	16.70
DNMT3A	mutated	c.1757G>A	p.Cys586Tyr	20.70
DNMT3A	mutated	c.1757G>T	p.Cys586Phe	3.34
DNMT3A	mutated	c.1758C>G	p.Cys586Trp	2.91
DNMT3A	mutated	c.1768_1769del	p.Gly590Tyrfs*21	8.90
DNMT3A	mutated	c.1769del	p.Gly590Valfs*61	3.56
DNMT3A	mutated	c.1776C>A	p.Tyr592*	2.16
DNMT3A	mutated	c.1776C>A	p.Tyr592*	2.74
DNMT3A	mutated	c.1776C>G	p.Tyr592*	3.05
DNMT3A	mutated	c.1776C>G	p.Tyr592*	3.07
DNMT3A	mutated	c.1792C>T	p.Arg598*	2.08
DNMT3A	mutated	c.1792C>T	p.Arg598*	2.09
DNMT3A	mutated	c.1792C>T	p.Arg598*	3.10
DNMT3A	mutated	c.1792C>T	p.Arg598*	3.78
DNMT3A	mutated	c.1792C>T	p.Arg598*	6.10

DNMT3A	mutated	c.1792C>T	p.Arg598*	7.40
DNMT3A	mutated	c.1792C>T	p.Arg598*	17.40
DNMT3A	mutated	c.1792C>T	p.Arg598*	24.70
DNMT3A	mutated	c.1793_1813del	p.Arg598_Arg604del	5.20
DNMT3A	mutated	c.1802G>A	p.Trp601*	3.55
DNMT3A	mutated	c.1803G>A	p.Trp601*	5.90
DNMT3A	mutated	c.1810del	p.Arg604Glyfs*47	2.43
DNMT3A	mutated	c.1812del	p.Leu605Serfs*46	2.76
DNMT3A	mutated	c.1814del	p.Leu605Profs*46	9.00
DNMT3A	mutated	c.1827del	p.Phe609Leufs*42	6.70
DNMT3A	mutated	c.1829C>G	p.Ala610Gly	2.86
DNMT3A	mutated	c.1843C>T	p.Gln615*	2.46
DNMT3A	mutated	c.1843C>T	p.Gln615*	4.66
DNMT3A	mutated	c.1856del	p.Pro619Leufs*32	30.40
DNMT3A	mutated	c.1862del	p.Lys621Argfs*30	13.10
DNMT3A	mutated	c.1872_1879del	p.Pro625Serfs*2	5.40
DNMT3A	mutated	c.1879_1880del	p.Pro627Serfs*2	4.11
DNMT3A	mutated	c.1880del	p.Pro627Glnfs*24	5.10
DNMT3A	mutated	c.1883del	p.Ala628Valfs*23	7.10
DNMT3A	mutated	c.1891A>T	p.Arg631Trp	4.00
DNMT3A	mutated	c.1892G>T	p.Arg631Met	4.56
DNMT3A	mutated	c.1894A>T	p.Lys632*	2.20
DNMT3A	mutated	c.1898C>T	p.Pro633Leu	3.09
DNMT3A	mutated	c.1900A>T	p.Ile634Phe	8.70
DNMT3A	mutated	c.1901_1926del	p.Ile634Asnfs*25	2.44
DNMT3A	mutated	c.1901T>C	p.Ile634Thr	5.70
DNMT3A	mutated	c.1903C>G	p.Arg635Gly	6.90
DNMT3A	mutated	c.1903C>T	p.Arg635Trp	2.13
DNMT3A	mutated	c.1903C>T	p.Arg635Trp	2.15
DNMT3A	mutated	c.1903C>T	p.Arg635Trp	2.29
DNMT3A	mutated	c.1903C>T	p.Arg635Trp	2.44
DNMT3A	mutated	c.1903C>T	p.Arg635Trp	2.45
DNMT3A	mutated	c.1903C>T	p.Arg635Trp	2.46
DNMT3A	mutated	c.1903C>T	p.Arg635Trp	2.49
DNMT3A	mutated	c.1903C>T	p.Arg635Trp	2.77
DNMT3A	mutated	c.1903C>T	p.Arg635Trp	2.78
DNMT3A	mutated	c.1903C>T	p.Arg635Trp	3.35
DNMT3A	mutated	c.1903C>T	p.Arg635Trp	3.75
DNMT3A	mutated	c.1903C>T	p.Arg635Trp	4.11
DNMT3A	mutated	c.1903C>T	p.Arg635Trp	4.65
DNMT3A	mutated	c.1903C>T	p.Arg635Trp	6.10
DNMT3A	mutated	c.1903C>T	p.Arg635Trp	6.30
DNMT3A	mutated	c.1903C>T	p.Arg635Trp	7.10
DNMT3A	mutated	c.1903C>T	p.Arg635Trp	8.10
DNMT3A	mutated	c.1903C>T	p.Arg635Trp	8.40
DNMT3A	mutated	c.1903C>T	p.Arg635Trp	8.60
DNMT3A	mutated	c.1903C>T	p.Arg635Trp	8.70

DNMT3A	mutated	c.1903C>T	p.Arg635Trp	8.80
DNMT3A	mutated	c.1903C>T	p.Arg635Trp	10.70
DNMT3A	mutated	c.1903C>T	p.Arg635Trp	11.80
DNMT3A	mutated	c.1903C>T	p.Arg635Trp	15.30
DNMT3A	mutated	c.1903C>T	p.Arg635Trp	17.60
DNMT3A	mutated	c.1903C>T	p.Arg635Trp	22.70
DNMT3A	mutated	c.1903C>T	p.Arg635Trp	33.30
DNMT3A	mutated	c.1903C>T	p.Arg635Trp	33.80
DNMT3A	mutated	c.1903C>T	p.Arg635Trp	39.40
DNMT3A	mutated	c.1904G>A	p.Arg635Gln	2.03
DNMT3A	mutated	c.1904G>A	p.Arg635Gln	2.61
DNMT3A	mutated	c.1904G>A	p.Arg635Gln	3.18
DNMT3A	mutated	c.1904G>A	p.Arg635Gln	3.47
DNMT3A	mutated	c.1904G>A	p.Arg635Gln	4.37
DNMT3A	mutated	c.1904G>A	p.Arg635Gln	5.20
DNMT3A	mutated	c.1904G>A	p.Arg635Gln	5.40
DNMT3A	mutated	c.1904G>A	p.Arg635Gln	5.70
DNMT3A	mutated	c.1904G>A	p.Arg635Gln	5.80
DNMT3A	mutated	c.1904G>A	p.Arg635Gln	13.50
DNMT3A	mutated	c.1904G>T	p.Arg635Leu	2.40
DNMT3A	mutated	c.1906del	p.Val636Cysfs*15	2.20
DNMT3A	mutated	c.1906G>A	p.Val636Met	3.41
DNMT3A	mutated	c.1906G>A	p.Val636Met	5.30
DNMT3A	mutated	c.1906G>A	p.Val636Met	6.10
DNMT3A	mutated	c.1906G>A	p.Val636Met	6.80
DNMT3A	mutated	c.1906G>A	p.Val636Met	7.00
DNMT3A	mutated	c.1906G>A	p.Val636Met	10.40
DNMT3A	mutated	c.1906G>T	p.Val636Leu	2.43
DNMT3A	mutated	c.1910T>A	p.Leu637Gln	3.33
DNMT3A	mutated	c.1910T>A	p.Leu637Gln	8.50
DNMT3A	mutated	c.1910T>C	p.Leu637Pro	20.30
DNMT3A	mutated	c.1912T>C	p.Ser638Pro	13.60
DNMT3A	mutated	c.1913C>T	p.Ser638Phe	2.01
DNMT3A	mutated	c.1913C>T	p.Ser638Phe	4.15
DNMT3A	mutated	c.1913del	p.Ser638Phefs*13	4.73
DNMT3A	mutated	c.1914del	p.Leu639Serfs*12	25.80
DNMT3A	mutated	c.1915C>T	p.Leu639Phe	4.84
DNMT3A	mutated	c.1915C>T	p.Leu639Phe	6.80
DNMT3A	mutated	c.1916del	p.Leu639Profs*12	11.60
DNMT3A	mutated	c.1916dup	p.Phe640Leufs*2	10.00
DNMT3A	mutated	c.1917_1918del	p.Phe640*	4.73
DNMT3A	mutated	c.1917_1918del	p.Phe640*	19.00
DNMT3A	mutated	c.1922A>T	p.Asp641Val	3.43
DNMT3A	mutated	c.1924G>A	p.Gly642Arg	2.18
DNMT3A	mutated	c.1924G>T	p.Gly642*	7.60
DNMT3A	mutated	c.1933A>G	p.Thr645Ala	3.15
DNMT3A	mutated	c.1936G>A	p.Gly646Arg	3.82

DNMT3A	mutated	c.1940T>C	p.Leu647Pro	2.62
DNMT3A	mutated	c.1940T>C	p.Leu647Pro	7.50
DNMT3A	mutated	c.1940T>C	p.Leu647Pro	16.00
DNMT3A	mutated	c.1940T>G	p.Leu647Arg	2.37
DNMT3A	mutated	c.1940T>G	p.Leu647Arg	10.10
DNMT3A	mutated	c.1943T>C	p.Leu648Pro	3.36
DNMT3A	mutated	c.1945G>A	p.Val649Met	3.44
DNMT3A	mutated	c.1945G>A	p.Val649Met	12.00
DNMT3A	mutated	c.1945G>C	p.Val649Leu	3.09
DNMT3A	mutated	c.1945G>C	p.Val649Leu	6.80
DNMT3A	mutated	c.1949T>A	p.Leu650Gln	2.92
DNMT3A	mutated	c.1949T>C	p.Leu650Pro	3.07
DNMT3A	mutated	c.1949T>G	p.Leu650Arg	3.26
DNMT3A	mutated	c.1958T>A	p.Leu653*	2.23
DNMT3A	mutated	c.1958T>C	p.Leu653Ser	38.50
DNMT3A	mutated	c.1958T>G	p.Leu653Trp	4.48
DNMT3A	mutated	c.1961G>T	p.Gly654Val	2.14
DNMT3A	mutated	c.1964T>A	p.Ile655Asn	5.70
DNMT3A	mutated	c.1964T>C	p.Ile655Thr	2.98
DNMT3A	mutated	c.1964T>C	p.Ile655Thr	3.12
DNMT3A	mutated	c.1969G>A	p.Val657Met	2.16
DNMT3A	mutated	c.1969G>A	p.Val657Met	2.17
DNMT3A	mutated	c.1969G>A	p.Val657Met	2.22
DNMT3A	mutated	c.1969G>A	p.Val657Met	2.48
DNMT3A	mutated	c.1969G>A	p.Val657Met	2.77
DNMT3A	mutated	c.1969G>A	p.Val657Met	3.78
DNMT3A	mutated	c.1969G>A	p.Val657Met	4.24
DNMT3A	mutated	c.1969G>A	p.Val657Met	4.51
DNMT3A	mutated	c.1969G>A	p.Val657Met	4.61
DNMT3A	mutated	c.1969G>A	p.Val657Met	5.20
DNMT3A	mutated	c.1969G>A	p.Val657Met	7.00
DNMT3A	mutated	c.1969G>A	p.Val657Met	7.10
DNMT3A	mutated	c.1969G>A	p.Val657Met	7.40
DNMT3A	mutated	c.1969G>A	p.Val657Met	9.00
DNMT3A	mutated	c.1969G>A	p.Val657Met	9.70
DNMT3A	mutated	c.1969G>A	p.Val657Met	15.20
DNMT3A	mutated	c.1969G>A	p.Val657Met	39.00
DNMT3A	mutated	c.1973A>G	p.Asp658Gly	2.19
DNMT3A	mutated	c.1973A>T	p.Asp658Val	5.90
DNMT3A	mutated	c.1975C>G	p.Arg659Gly	6.30
DNMT3A	mutated	c.1976G>A	p.Arg659His	2.41
DNMT3A	mutated	c.1976G>A	p.Arg659His	2.98
DNMT3A	mutated	c.1976G>A	p.Arg659His	5.10
DNMT3A	mutated	c.1976G>A	p.Arg659His	5.40
DNMT3A	mutated	c.1976G>A	p.Arg659His	7.20
DNMT3A	mutated	c.1976G>A	p.Arg659His	18.30
DNMT3A	mutated	c.1978T>A	p.Tyr660Asn	5.60

DNMT3A	mutated	c.1979A>G	p.Tyr660Cys	2.30
DNMT3A	mutated	c.1979A>G	p.Tyr660Cys	3.10
DNMT3A	mutated	c.1979A>G	p.Tyr660Cys	11.00
DNMT3A	mutated	c.1981A>C	p.Ile661Leu	2.18
DNMT3A	mutated	c.1981del	p.Ile661Leufs*44	3.03
DNMT3A	mutated	c.1984_1985delinsAG	p.Ala662Ser	4.81
DNMT3A	mutated	c.1985C>A	p.Ala662Asp	5.60
DNMT3A	mutated	c.1988C>G	p.Ser663Trp	3.54
DNMT3A	mutated	c.1990G>C	p.Glu664Gln	2.24
DNMT3A	mutated	c.1991A>G	p.Glu664Gly	2.50
DNMT3A	mutated	c.1991A>G	p.Glu664Gly	5.70
DNMT3A	mutated	c.1993del	p.Val665Cysfs*40	6.20
DNMT3A	mutated	c.1998T>A	p.Cys666*	2.19
DNMT3A	mutated	c.2006C>T	p.Ser669Phe	2.18
DNMT3A	mutated	c.2007dup	p.Ile670Hisfs*43	2.31
DNMT3A	mutated	c.2007dup	p.Ile670Hisfs*43	2.78
DNMT3A	mutated	c.2007dup	p.Ile670Hisfs*43	2.90
DNMT3A	mutated	c.2007dup	p.Ile670Hisfs*43	10.80
DNMT3A	mutated	c.2011del	p.Thr671Argfs*34	2.54
DNMT3A	mutated	c.2011del	p.Thr671Argfs*34	13.00
DNMT3A	mutated	c.2015_2018del	p.Val672Alafs*32	13.00
DNMT3A	mutated	c.2017G>C	p.Gly673Arg	4.08
DNMT3A	mutated	c.2023G>A	p.Val675Met	2.26
DNMT3A	mutated	c.2023G>A	p.Val675Met	2.39
DNMT3A	mutated	c.2023G>A	p.Val675Met	5.10
DNMT3A	mutated	c.2023G>A	p.Val675Met	6.10
DNMT3A	mutated	c.2026C>T	p.Arg676Trp	2.41
DNMT3A	mutated	c.2026C>T	p.Arg676Trp	31.10
DNMT3A	mutated	c.2030_2031del	p.His677Profs*35	5.90
DNMT3A	mutated	c.2032C>T	p.Gln678*	4.63
DNMT3A	mutated	c.2033del	p.Gln678Argfs*27	10.60
DNMT3A	mutated	c.2037del	p.Lys680Argfs*25	7.10
DNMT3A	mutated	c.2043del	p.Met682Cysfs*23	2.58
DNMT3A	mutated	c.2043del	p.Met682Cysfs*23	3.60
DNMT3A	mutated	c.2043del	p.Met682Cysfs*23	3.60
DNMT3A	mutated	c.2043del	p.Met682Cysfs*23	18.90
DNMT3A	mutated	c.2047T>A	p.Tyr683Asn	8.90
DNMT3A	mutated	c.2049C>G	p.Tyr683*	2.13
DNMT3A	mutated	c.2050_2051delinsCC	p.Val684Pro	15.00
DNMT3A	mutated	c.2053G>A	p.Gly685Arg	2.73
DNMT3A	mutated	c.2053G>A	p.Gly685Arg	3.48
DNMT3A	mutated	c.2053G>A	p.Gly685Arg	3.78
DNMT3A	mutated	c.2053G>A	p.Gly685Arg	4.63
DNMT3A	mutated	c.2053G>A	p.Gly685Arg	18.60
DNMT3A	mutated	c.2054G>A	p.Gly685Glu	2.42
DNMT3A	mutated	c.2054G>C	p.Gly685Ala	2.47
DNMT3A	mutated	c.2054G>C	p.Gly685Ala	2.89

DNMT3A	mutated	c.2054G>C	p.Gly685Ala	11.00
DNMT3A	mutated	c.2055_2063del	p.Asp686_Arg688del	8.60
DNMT3A	mutated	c.2057A>T	p.Asp686Val	2.88
DNMT3A	mutated	c.2057A>T	p.Asp686Val	4.21
DNMT3A	mutated	c.2060T>G	p.Val687Gly	3.62
DNMT3A	mutated	c.2060T>G	p.Val687Gly	7.30
DNMT3A	mutated	c.2062C>T	p.Arg688Cys	2.35
DNMT3A	mutated	c.2062C>T	p.Arg688Cys	7.10
DNMT3A	mutated	c.2063G>A	p.Arg688His	2.04
DNMT3A	mutated	c.2063G>A	p.Arg688His	2.08
DNMT3A	mutated	c.2063G>A	p.Arg688His	3.68
DNMT3A	mutated	c.2063G>A	p.Arg688His	7.20
DNMT3A	mutated	c.2063G>T	p.Arg688Leu	8.20
DNMT3A	mutated	c.2069T>A	p.Val690Asp	4.01
DNMT3A	mutated	c.2071dup	p.Thr691Asnfs*22	25.40
DNMT3A	mutated	c.2072C>T	p.Thr691Ile	3.76
DNMT3A	mutated	c.2074_2075del	p.Gln692Glufs*20	12.20
DNMT3A	mutated	c.2074C>T	p.Gln692*	4.41
DNMT3A	mutated	c.2074C>T	p.Gln692*	9.20
DNMT3A	mutated	c.2077_2079del	p.Lys693del	19.00
DNMT3A	mutated	c.2080C>T	p.His694Tyr	2.25
DNMT3A	mutated	c.2080C>T	p.His694Tyr	6.00
DNMT3A	mutated	c.2081dup	p.His694Glnfs*19	27.10
DNMT3A	mutated	c.2084T>A	p.Ile695Asn	2.44
DNMT3A	mutated	c.2084T>C	p.Ile695Thr	3.71
DNMT3A	mutated	c.2084T>C	p.Ile695Thr	12.40
DNMT3A	mutated	c.2084T>G	p.Ile695Ser	2.33
DNMT3A	mutated	c.2086C>T	p.Gln696*	3.62
DNMT3A	mutated	c.2086C>T	p.Gln696*	16.50
DNMT3A	mutated	c.2089G>T	p.Glu697*	2.87
DNMT3A	mutated	c.2091del	p.Glu697Aspfs*8	3.30
DNMT3A	mutated	c.2092T>C	p.Trp698Arg	2.75
DNMT3A	mutated	c.2093G>A	p.Trp698*	3.62
DNMT3A	mutated	c.2093G>A	p.Trp698*	4.40
DNMT3A	mutated	c.2095G>A	p.Gly699Ser	5.90
DNMT3A	mutated	c.2095G>C	p.Gly699Arg	4.35
DNMT3A	mutated	c.2096G>A	p.Gly699Asp	2.51
DNMT3A	mutated	c.2096G>A	p.Gly699Asp	3.96
DNMT3A	mutated	c.2096G>A	p.Gly699Asp	5.10
DNMT3A	mutated	c.2096G>T	p.Gly699Val	5.50
DNMT3A	mutated	c.2098C>A	p.Pro700Thr	2.76
DNMT3A	mutated	c.2099C>A	p.Pro700Gln	6.50
DNMT3A	mutated	c.2099C>T	p.Pro700Leu	2.53
DNMT3A	mutated	c.2099C>T	p.Pro700Leu	5.20
DNMT3A	mutated	c.2099C>T	p.Pro700Leu	23.60
DNMT3A	mutated	c.209C>T	p.Ala70Val	44.50
DNMT3A	mutated	c.2101_2122del	p.Phe701Valfs*71	27.00

DNMT3A	mutated	c.2104G>T	p.Asp702Tyr	20.10
DNMT3A	mutated	c.2105A>T	p.Asp702Val	5.80
DNMT3A	mutated	c.2108T>A	p.Leu703Gln	2.63
DNMT3A	mutated	c.2111T>C	p.Val704Ala	4.52
DNMT3A	mutated	c.2114T>C	p.Ile705Thr	2.29
DNMT3A	mutated	c.2114T>C	p.Ile705Thr	2.60
DNMT3A	mutated	c.2114T>C	p.Ile705Thr	2.82
DNMT3A	mutated	c.2114T>C	p.Ile705Thr	3.67
DNMT3A	mutated	c.2114T>C	p.Ile705Thr	5.00
DNMT3A	mutated	c.2114T>C	p.Ile705Thr	5.40
DNMT3A	mutated	c.2114T>C	p.Ile705Thr	5.50
DNMT3A	mutated	c.2114T>C	p.Ile705Thr	6.00
DNMT3A	mutated	c.2114T>C	p.Ile705Thr	6.30
DNMT3A	mutated	c.2114T>G	p.Ile705Ser	2.31
DNMT3A	mutated	c.2116G>A	p.Gly706Arg	2.10
DNMT3A	mutated	c.2116G>A	p.Gly706Arg	6.50
DNMT3A	mutated	c.2116G>T	p.Gly706Trp	8.20
DNMT3A	mutated	c.2117G>A	p.Gly706Glu	2.19
DNMT3A	mutated	c.2119G>T	p.Gly707Cys	23.50
DNMT3A	mutated	c.2120dup	p.Ser708Glnfs*5	2.77
DNMT3A	mutated	c.2120dup	p.Ser708Glnfs*5	14.50
DNMT3A	mutated	c.2120G>A	p.Gly707Asp	3.75
DNMT3A	mutated	c.2120G>A	p.Gly707Asp	5.20
DNMT3A	mutated	c.2120G>T	p.Gly707Val	7.90
DNMT3A	mutated	c.2129G>A	p.Cys710Tyr	28.50
DNMT3A	mutated	c.2137_2138delinsA	p.Leu713Thrfs*66	2.94
DNMT3A	mutated	c.2139del	p.Ser714Profs*65	4.61
DNMT3A	mutated	c.2141C>G	p.Ser714Cys	2.05
DNMT3A	mutated	c.2141C>G	p.Ser714Cys	2.12
DNMT3A	mutated	c.2141C>G	p.Ser714Cys	2.14
DNMT3A	mutated	c.2141C>G	p.Ser714Cys	2.37
DNMT3A	mutated	c.2141C>G	p.Ser714Cys	2.97
DNMT3A	mutated	c.2141C>G	p.Ser714Cys	3.07
DNMT3A	mutated	c.2141C>G	p.Ser714Cys	3.43
DNMT3A	mutated	c.2141C>G	p.Ser714Cys	5.30
DNMT3A	mutated	c.2141C>G	p.Ser714Cys	24.20
DNMT3A	mutated	c.2141C>T	p.Ser714Phe	2.09
DNMT3A	mutated	c.2141C>T	p.Ser714Phe	2.79
DNMT3A	mutated	c.2141C>T	p.Ser714Phe	3.46
DNMT3A	mutated	c.2141C>T	p.Ser714Phe	6.20
DNMT3A	mutated	c.2143_2144del	p.Ile715Argfs*18	8.00
DNMT3A	mutated	c.2146G>A	p.Val716Ile	5.80
DNMT3A	mutated	c.2147T>A	p.Val716Asp	2.30
DNMT3A	mutated	c.2150A>G	p.Asn717Ser	2.01
DNMT3A	mutated	c.2150A>G	p.Asn717Ser	2.40
DNMT3A	mutated	c.2150A>T	p.Asn717Ile	3.55
DNMT3A	mutated	c.2150A>T	p.Asn717Ile	13.20

DNMT3A	mutated	c.2150dup	p.Asn717Lysfs*17	5.80
DNMT3A	mutated	c.2157_2173+75del	p.splice site mutation	4.83
DNMT3A	mutated	c.2158C>A	p.Arg720Ser	10.40
DNMT3A	mutated	c.2160dup	p.Lys721Glnfs*13	41.30
DNMT3A	mutated	c.2161A>G	p.Lys721Glu	3.52
DNMT3A	mutated	c.2171A>G	p.Tyr724Cys	2.41
DNMT3A	mutated	c.2172C>G	p.Tyr724*	35.60
DNMT3A	mutated	c.2177del	p.Gly726Alafs*53	2.91
DNMT3A	mutated	c.2183G>A	p.Gly728Asp	2.10
DNMT3A	mutated	c.2185C>G	p.Arg729Gly	2.25
DNMT3A	mutated	c.2185C>G	p.Arg729Gly	2.56
DNMT3A	mutated	c.2185C>G	p.Arg729Gly	2.89
DNMT3A	mutated	c.2185C>G	p.Arg729Gly	3.18
DNMT3A	mutated	c.2185C>G	p.Arg729Gly	3.52
DNMT3A	mutated	c.2185C>G	p.Arg729Gly	4.21
DNMT3A	mutated	c.2185C>G	p.Arg729Gly	13.30
DNMT3A	mutated	c.2185C>T	p.Arg729Trp	2.38
DNMT3A	mutated	c.2185C>T	p.Arg729Trp	2.50
DNMT3A	mutated	c.2185C>T	p.Arg729Trp	2.79
DNMT3A	mutated	c.2185C>T	p.Arg729Trp	2.91
DNMT3A	mutated	c.2185C>T	p.Arg729Trp	3.14
DNMT3A	mutated	c.2185C>T	p.Arg729Trp	3.21
DNMT3A	mutated	c.2185C>T	p.Arg729Trp	3.35
DNMT3A	mutated	c.2185C>T	p.Arg729Trp	3.44
DNMT3A	mutated	c.2185C>T	p.Arg729Trp	4.11
DNMT3A	mutated	c.2185C>T	p.Arg729Trp	5.20
DNMT3A	mutated	c.2185C>T	p.Arg729Trp	5.80
DNMT3A	mutated	c.2185C>T	p.Arg729Trp	5.90
DNMT3A	mutated	c.2185C>T	p.Arg729Trp	6.30
DNMT3A	mutated	c.2185C>T	p.Arg729Trp	6.60
DNMT3A	mutated	c.2185C>T	p.Arg729Trp	6.60
DNMT3A	mutated	c.2185C>T	p.Arg729Trp	7.30
DNMT3A	mutated	c.2185C>T	p.Arg729Trp	8.60
DNMT3A	mutated	c.2185C>T	p.Arg729Trp	10.10
DNMT3A	mutated	c.2186G>A	p.Arg729Gln	3.21
DNMT3A	mutated	c.2186G>A	p.Arg729Gln	5.70
DNMT3A	mutated	c.2186G>A	p.Arg729Gln	16.20
DNMT3A	mutated	c.2188del	p.Leu730Serfs*49	2.14
DNMT3A	mutated	c.2190_2194del	p.Phe731*	3.30
DNMT3A	mutated	c.2191T>C	p.Phe731Leu	5.10
DNMT3A	mutated	c.2192T>A	p.Phe731Tyr	3.10
DNMT3A	mutated	c.2192T>G	p.Phe731Cys	3.06
DNMT3A	mutated	c.2193_2195del	p.Phe732del	2.24
DNMT3A	mutated	c.2193_2195del	p.Phe732del	3.67
DNMT3A	mutated	c.2193_2195del	p.Phe732del	7.10
DNMT3A	mutated	c.2193_2195del	p.Phe732del	7.30
DNMT3A	mutated	c.2193_2195del	p.Phe732del	9.90

DNMT3A	mutated	c.2193_2195del	p.Phe732del	30.40
DNMT3A	mutated	c.2193C>A	p.Phe731Leu	3.08
DNMT3A	mutated	c.2193C>A	p.Phe731Leu	5.60
DNMT3A	mutated	c.2194_2196del	p.Phe732del	2.72
DNMT3A	mutated	c.2194T>A	p.Phe732Ile	2.09
DNMT3A	mutated	c.2194T>C	p.Phe732Leu	4.79
DNMT3A	mutated	c.2194T>C	p.Phe732Leu	5.10
DNMT3A	mutated	c.2194T>C	p.Phe732Leu	19.10
DNMT3A	mutated	c.2195_2203del	p.Phe732_Phe734del	9.40
DNMT3A	mutated	c.2195T>C	p.Phe732Ser	2.51
DNMT3A	mutated	c.2195T>C	p.Phe732Ser	2.60
DNMT3A	mutated	c.2195T>C	p.Phe732Ser	4.46
DNMT3A	mutated	c.2196dup	p.Glu733*	2.09
DNMT3A	mutated	c.2196dup	p.Glu733*	2.13
DNMT3A	mutated	c.2196dup	p.Glu733*	3.03
DNMT3A	mutated	c.2196dup	p.Glu733*	4.34
DNMT3A	mutated	c.2196dup	p.Glu733*	5.20
DNMT3A	mutated	c.2197G>A	p.Glu733Lys	2.99
DNMT3A	mutated	c.2200T>C	p.Phe734Leu	3.02
DNMT3A	mutated	c.2201T>C	p.Phe734Ser	2.56
DNMT3A	mutated	c.2203T>A	p.Tyr735Asn	12.20
DNMT3A	mutated	c.2204A>G	p.Tyr735Cys	2.09
DNMT3A	mutated	c.2204A>G	p.Tyr735Cys	2.21
DNMT3A	mutated	c.2204A>G	p.Tyr735Cys	2.25
DNMT3A	mutated	c.2204A>G	p.Tyr735Cys	2.65
DNMT3A	mutated	c.2204A>G	p.Tyr735Cys	2.75
DNMT3A	mutated	c.2204A>G	p.Tyr735Cys	2.92
DNMT3A	mutated	c.2204A>G	p.Tyr735Cys	3.11
DNMT3A	mutated	c.2204A>G	p.Tyr735Cys	3.12
DNMT3A	mutated	c.2204A>G	p.Tyr735Cys	3.25
DNMT3A	mutated	c.2204A>G	p.Tyr735Cys	3.35
DNMT3A	mutated	c.2204A>G	p.Tyr735Cys	4.07
DNMT3A	mutated	c.2204A>G	p.Tyr735Cys	4.30
DNMT3A	mutated	c.2204A>G	p.Tyr735Cys	4.34
DNMT3A	mutated	c.2204A>G	p.Tyr735Cys	4.74
DNMT3A	mutated	c.2204A>G	p.Tyr735Cys	5.10
DNMT3A	mutated	c.2204A>G	p.Tyr735Cys	5.10
DNMT3A	mutated	c.2204A>G	p.Tyr735Cys	5.20
DNMT3A	mutated	c.2204A>G	p.Tyr735Cys	5.30
DNMT3A	mutated	c.2204A>G	p.Tyr735Cys	5.40
DNMT3A	mutated	c.2204A>G	p.Tyr735Cys	5.60
DNMT3A	mutated	c.2204A>G	p.Tyr735Cys	6.00
DNMT3A	mutated	c.2204A>G	p.Tyr735Cys	6.00
DNMT3A	mutated	c.2204A>G	p.Tyr735Cys	8.30
DNMT3A	mutated	c.2204A>G	p.Tyr735Cys	9.30
DNMT3A	mutated	c.2204A>G	p.Tyr735Cys	9.50
DNMT3A	mutated	c.2204A>G	p.Tyr735Cys	11.00

DNMT3A	mutated	c.2204A>G	p.Tyr735Cys	12.30
DNMT3A	mutated	c.2204A>G	p.Tyr735Cys	14.60
DNMT3A	mutated	c.2204A>G	p.Tyr735Cys	19.30
DNMT3A	mutated	c.2204A>G	p.Tyr735Cys	22.80
DNMT3A	mutated	c.2206C>A	p.Arg736Ser	31.30
DNMT3A	mutated	c.2206C>T	p.Arg736Cys	2.28
DNMT3A	mutated	c.2206C>T	p.Arg736Cys	2.53
DNMT3A	mutated	c.2206C>T	p.Arg736Cys	2.84
DNMT3A	mutated	c.2206C>T	p.Arg736Cys	3.87
DNMT3A	mutated	c.2206C>T	p.Arg736Cys	3.97
DNMT3A	mutated	c.2206C>T	p.Arg736Cys	4.10
DNMT3A	mutated	c.2206C>T	p.Arg736Cys	5.40
DNMT3A	mutated	c.2206C>T	p.Arg736Cys	6.10
DNMT3A	mutated	c.2206C>T	p.Arg736Cys	6.70
DNMT3A	mutated	c.2206C>T	p.Arg736Cys	10.20
DNMT3A	mutated	c.2206C>T	p.Arg736Cys	14.50
DNMT3A	mutated	c.2206C>T	p.Arg736Cys	17.10
DNMT3A	mutated	c.2206C>T	p.Arg736Cys	17.70
DNMT3A	mutated	c.2206C>T	p.Arg736Cys	22.00
DNMT3A	mutated	c.2206C>T	p.Arg736Cys	22.90
DNMT3A	mutated	c.2207G>A	p.Arg736His	2.10
DNMT3A	mutated	c.2207G>A	p.Arg736His	2.11
DNMT3A	mutated	c.2207G>A	p.Arg736His	2.22
DNMT3A	mutated	c.2207G>A	p.Arg736His	2.33
DNMT3A	mutated	c.2207G>A	p.Arg736His	2.39
DNMT3A	mutated	c.2207G>A	p.Arg736His	2.47
DNMT3A	mutated	c.2207G>A	p.Arg736His	2.84
DNMT3A	mutated	c.2207G>A	p.Arg736His	2.95
DNMT3A	mutated	c.2207G>A	p.Arg736His	3.05
DNMT3A	mutated	c.2207G>A	p.Arg736His	3.21
DNMT3A	mutated	c.2207G>A	p.Arg736His	3.84
DNMT3A	mutated	c.2207G>A	p.Arg736His	4.16
DNMT3A	mutated	c.2207G>A	p.Arg736His	4.20
DNMT3A	mutated	c.2207G>A	p.Arg736His	4.41
DNMT3A	mutated	c.2207G>A	p.Arg736His	4.93
DNMT3A	mutated	c.2207G>A	p.Arg736His	5.50
DNMT3A	mutated	c.2207G>A	p.Arg736His	5.60
DNMT3A	mutated	c.2207G>A	p.Arg736His	6.20
DNMT3A	mutated	c.2207G>A	p.Arg736His	6.80
DNMT3A	mutated	c.2207G>A	p.Arg736His	7.00
DNMT3A	mutated	c.2207G>A	p.Arg736His	7.80
DNMT3A	mutated	c.2207G>A	p.Arg736His	7.80
DNMT3A	mutated	c.2207G>A	p.Arg736His	13.10
DNMT3A	mutated	c.2207G>A	p.Arg736His	14.60
DNMT3A	mutated	c.2207G>A	p.Arg736His	17.30
DNMT3A	mutated	c.2207G>A	p.Arg736His	24.10
DNMT3A	mutated	c.2207G>A	p.Arg736His	29.40

DNMT3A	mutated	c.2207G>A	p.Arg736His	30.10
DNMT3A	mutated	c.2207G>A	p.Arg736His	30.70
DNMT3A	mutated	c.2207G>A	p.Arg736His	31.60
DNMT3A	mutated	c.2207G>A	p.Arg736His	37.60
DNMT3A	mutated	c.2207G>T	p.Arg736Leu	2.07
DNMT3A	mutated	c.2209C>T	p.Leu737Phe	2.29
DNMT3A	mutated	c.2210del	p.Leu737Profs*42	6.30
DNMT3A	mutated	c.2210T>A	p.Leu737His	2.21
DNMT3A	mutated	c.2210T>G	p.Leu737Arg	4.55
DNMT3A	mutated	c.2215del	p.His739Metfs*40	2.57
DNMT3A	mutated	c.2216A>C	p.His739Pro	8.20
DNMT3A	mutated	c.2221dup	p.Ala741Glyfs*8	4.66
DNMT3A	mutated	c.2221dup	p.Ala741Glyfs*8	6.80
DNMT3A	mutated	c.2222C>A	p.Ala741Glu	3.13
DNMT3A	mutated	c.2222C>G	p.Ala741Gly	2.59
DNMT3A	mutated	c.2222C>G	p.Ala741Gly	6.00
DNMT3A	mutated	c.2222C>G	p.Ala741Gly	7.30
DNMT3A	mutated	c.2222C>T	p.Ala741Val	6.70
DNMT3A	mutated	c.2224_2225dup	p.Pro743Glyfs*37	8.80
DNMT3A	mutated	c.2228C>T	p.Pro743Leu	12.60
DNMT3A	mutated	c.2229del	p.Lys744Argfs*35	2.86
DNMT3A	mutated	c.2230A>T	p.Lys744*	2.79
DNMT3A	mutated	c.2245_2253del	p.Arg749_Phe751del	3.03
DNMT3A	mutated	c.2245C>A	p.Arg749Ser	2.11
DNMT3A	mutated	c.2245C>T	p.Arg749Cys	2.32
DNMT3A	mutated	c.2245C>T	p.Arg749Cys	3.26
DNMT3A	mutated	c.2245C>T	p.Arg749Cys	3.34
DNMT3A	mutated	c.2245C>T	p.Arg749Cys	4.55
DNMT3A	mutated	c.2245C>T	p.Arg749Cys	6.10
DNMT3A	mutated	c.2245C>T	p.Arg749Cys	8.70
DNMT3A	mutated	c.2245C>T	p.Arg749Cys	36.10
DNMT3A	mutated	c.2246del	p.Arg749Profs*30	3.28
DNMT3A	mutated	c.2246G>A	p.Arg749His	2.47
DNMT3A	mutated	c.2246G>A	p.Arg749His	7.20
DNMT3A	mutated	c.2246G>A	p.Arg749His	8.40
DNMT3A	mutated	c.2246G>A	p.Arg749His	22.20
DNMT3A	mutated	c.2246G>T	p.Arg749Leu	5.10
DNMT3A	mutated	c.2248C>T	p.Pro750Ser	7.90
DNMT3A	mutated	c.2249_2260del	p.Pro750_Trp753del	3.97
DNMT3A	mutated	c.2250del	p.Phe751Serfs*28	2.27
DNMT3A	mutated	c.2250del	p.Phe751Serfs*28	11.00
DNMT3A	mutated	c.2253C>A	p.Phe751Leu	2.02
DNMT3A	mutated	c.2253C>A	p.Phe751Leu	14.10
DNMT3A	mutated	c.2254T>A	p.Phe752Ile	2.75
DNMT3A	mutated	c.2254T>G	p.Phe752Val	2.42
DNMT3A	mutated	c.2254T>G	p.Phe752Val	5.60
DNMT3A	mutated	c.2255_2257del	p.Phe752del	11.00

DNMT3A	mutated	c.2256C>G	p.Phe752Leu	18.90
DNMT3A	mutated	c.2257T>C	p.Trp753Arg	6.90
DNMT3A	mutated	c.2257T>G	p.Trp753Gly	12.70
DNMT3A	mutated	c.2258G>A	p.Trp753*	2.35
DNMT3A	mutated	c.2258G>A	p.Trp753*	3.25
DNMT3A	mutated	c.2258G>A	p.Trp753*	4.76
DNMT3A	mutated	c.2258G>T	p.Trp753Leu	2.87
DNMT3A	mutated	c.2261_2265del	p.Leu754Argfs*9	7.00
DNMT3A	mutated	c.2261T>C	p.Leu754Pro	8.80
DNMT3A	mutated	c.2261T>G	p.Leu754Arg	5.70
DNMT3A	mutated	c.2263T>C	p.Phe755Leu	19.00
DNMT3A	mutated	c.2263T>G	p.Phe755Val	3.58
DNMT3A	mutated	c.2264T>C	p.Phe755Ser	2.42
DNMT3A	mutated	c.2264T>C	p.Phe755Ser	2.54
DNMT3A	mutated	c.2264T>C	p.Phe755Ser	2.63
DNMT3A	mutated	c.2264T>C	p.Phe755Ser	2.98
DNMT3A	mutated	c.2264T>C	p.Phe755Ser	3.39
DNMT3A	mutated	c.2264T>C	p.Phe755Ser	4.42
DNMT3A	mutated	c.2264T>C	p.Phe755Ser	5.60
DNMT3A	mutated	c.2264T>C	p.Phe755Ser	6.80
DNMT3A	mutated	c.2264T>C	p.Phe755Ser	28.60
DNMT3A	mutated	c.2265_2278dup	p.Ala760Valfs*24	8.60
DNMT3A	mutated	c.2265del	p.Phe755Leufs*24	2.68
DNMT3A	mutated	c.2265del	p.Phe755Leufs*24	7.70
DNMT3A	mutated	c.2265dup	p.Glu756*	34.50
DNMT3A	mutated	c.2266G>A	p.Glu756Lys	5.60
DNMT3A	mutated	c.2267A>G	p.Glu756Gly	2.94
DNMT3A	mutated	c.2268G>C	p.Glu756Asp	2.08
DNMT3A	mutated	c.2268G>C	p.Glu756Asp	3.76
DNMT3A	mutated	c.2269_2272del	p.Asn757Trpfs*21	2.13
DNMT3A	mutated	c.2270A>T	p.Asn757Ile	2.53
DNMT3A	mutated	c.2271T>A	p.Asn757Lys	11.40
DNMT3A	mutated	c.2280_2286del	p.Met761Leufs*16	7.50
DNMT3A	mutated	c.2285del	p.Gly762Alafs*17	2.63
DNMT3A	mutated	c.2285G>T	p.Gly762Val	6.90
DNMT3A	mutated	c.2287_2288del	p.Val763*	5.50
DNMT3A	mutated	c.2289del	p.Ser764Valfs*15	6.60
DNMT3A	mutated	c.2294A>G	p.Asp765Gly	2.38
DNMT3A	mutated	c.2294A>G	p.Asp765Gly	2.58
DNMT3A	mutated	c.2294A>G	p.Asp765Gly	5.20
DNMT3A	mutated	c.2296_2297del	p.Lys766Glufs*15	4.00
DNMT3A	mutated	c.2299A>G	p.Arg767Gly	3.63
DNMT3A	mutated	c.2301_2302del	p.Asp768Hisfs*13	5.10
DNMT3A	mutated	c.2301_2302dup	p.Asp768Glyfs*12	19.00
DNMT3A	mutated	c.2302G>A	p.Asp768Asn	5.50
DNMT3A	mutated	c.2302G>T	p.Asp768Tyr	18.30
DNMT3A	mutated	c.2303A>G	p.Asp768Gly	2.21

DNMT3A	mutated	c.2303A>T	p.Asp768Val	2.90
DNMT3A	mutated	c.2305_2309del	p.Ile769Alafs*11	4.58
DNMT3A	mutated	c.2306T>C	p.Ile769Thr	2.40
DNMT3A	mutated	c.2309C>A	p.Ser770*	2.91
DNMT3A	mutated	c.2309C>A	p.Ser770*	3.54
DNMT3A	mutated	c.2309C>A	p.Ser770*	5.10
DNMT3A	mutated	c.2309C>A	p.Ser770*	7.30
DNMT3A	mutated	c.2309C>T	p.Ser770Leu	3.33
DNMT3A	mutated	c.2309C>T	p.Ser770Leu	3.40
DNMT3A	mutated	c.2309C>T	p.Ser770Leu	3.49
DNMT3A	mutated	c.2309C>T	p.Ser770Leu	5.20
DNMT3A	mutated	c.2309C>T	p.Ser770Leu	5.40
DNMT3A	mutated	c.2309C>T	p.Ser770Leu	7.10
DNMT3A	mutated	c.2309C>T	p.Ser770Leu	12.40
DNMT3A	mutated	c.2309C>T	p.Ser770Leu	12.70
DNMT3A	mutated	c.2311C>A	p.Arg771Arg	3.51
DNMT3A	mutated	c.2311C>T	p.Arg771*	2.32
DNMT3A	mutated	c.2311C>T	p.Arg771*	3.05
DNMT3A	mutated	c.2311C>T	p.Arg771*	3.54
DNMT3A	mutated	c.2311C>T	p.Arg771*	3.74
DNMT3A	mutated	c.2311C>T	p.Arg771*	5.60
DNMT3A	mutated	c.2311C>T	p.Arg771*	6.80
DNMT3A	mutated	c.2311C>T	p.Arg771*	7.40
DNMT3A	mutated	c.2311C>T	p.Arg771*	7.60
DNMT3A	mutated	c.2311C>T	p.Arg771*	8.90
DNMT3A	mutated	c.2311C>T	p.Arg771*	9.10
DNMT3A	mutated	c.2311C>T	p.Arg771*	12.50
DNMT3A	mutated	c.2311C>T	p.Arg771*	22.20
DNMT3A	mutated	c.2312G>A	p.Arg771Gln	2.79
DNMT3A	mutated	c.2312G>A	p.Arg771Gln	4.80
DNMT3A	mutated	c.2312G>A	p.Arg771Gln	21.90
DNMT3A	mutated	c.2312G>A	p.Arg771Gln	25.60
DNMT3A	mutated	c.2312G>C	p.Arg771Pro	2.07
DNMT3A	mutated	c.2312G>C	p.Arg771Pro	24.20
DNMT3A	mutated	c.2312G>T	p.Arg771Leu	3.71
DNMT3A	mutated	c.2317C>A	p.Leu773Ile	3.25
DNMT3A	mutated	c.2318T>A	p.Leu773His	4.41
DNMT3A	mutated	c.2318T>G	p.Leu773Arg	4.63
DNMT3A	mutated	c.2321A>G	p.Glu774Gly	4.49
DNMT3A	mutated	c.2321A>G	p.Glu774Gly	6.40
DNMT3A	mutated	c.2322+2T>C	p.splice site mutation	2.27
DNMT3A	mutated	c.2322G>C	p.Glu774Asp	5.10
DNMT3A	mutated	c.2322G>T	p.Glu774Asp	2.73
DNMT3A	mutated	c.2322G>T	p.Glu774Asp	24.00
DNMT3A	mutated	c.2323T>C	p.Ser775Pro	2.41
DNMT3A	mutated	c.2325del	p.Asn776Thrfs*3	4.15
DNMT3A	mutated	c.2329C>A	p.Pro777Thr	3.55

DNMT3A	mutated	c.2329C>A	p.Pro777Thr	12.80
DNMT3A	mutated	c.2329C>T	p.Pro777Ser	5.80
DNMT3A	mutated	c.2329C>T	p.Pro777Ser	19.10
DNMT3A	mutated	c.2330C>A	p.Pro777His	4.09
DNMT3A	mutated	c.2330C>A	p.Pro777His	7.80
DNMT3A	mutated	c.2330C>G	p.Pro777Arg	2.84
DNMT3A	mutated	c.2330C>G	p.Pro777Arg	4.12
DNMT3A	mutated	c.2330C>T	p.Pro777Leu	16.20
DNMT3A	mutated	c.2332G>A	p.Val778Met	3.30
DNMT3A	mutated	c.2337_2339del	p.Met779del	3.10
DNMT3A	mutated	c.2339T>C	p.Ile780Thr	2.23
DNMT3A	mutated	c.2339T>C	p.Ile780Thr	3.03
DNMT3A	mutated	c.2339T>C	p.Ile780Thr	6.80
DNMT3A	mutated	c.2339T>C	p.Ile780Thr	13.70
DNMT3A	mutated	c.2339T>C	p.Ile780Thr	29.20
DNMT3A	mutated	c.2350del	p.Glu784Lysfs*18	2.79
DNMT3A	mutated	c.2357C>A	p.Ser786*	2.52
DNMT3A	mutated	c.2358del	p.Ala787Leufs*15	3.64
DNMT3A	mutated	c.2372C>T	p.Ala791Val	2.69
DNMT3A	mutated	c.2375del	p.Arg792Profs*10	2.52
DNMT3A	mutated	c.2375G>A	p.Arg792His	4.42
DNMT3A	mutated	c.2377T>G	p.Tyr793Asp	2.42
DNMT3A	mutated	c.2381_2383del	p.Phe794del	19.70
DNMT3A	mutated	c.2381T>C	p.Phe794Ser	2.77
DNMT3A	mutated	c.2383T>A	p.Trp795Arg	5.20
DNMT3A	mutated	c.2384G>A	p.Trp795*	2.56
DNMT3A	mutated	c.2384G>A	p.Trp795*	8.00
DNMT3A	mutated	c.2384G>A	p.Trp795*	22.80
DNMT3A	mutated	c.2384G>C	p.Trp795Ser	5.40
DNMT3A	mutated	c.2385G>A	p.Trp795*	2.17
DNMT3A	mutated	c.2385G>A	p.Trp795*	3.17
DNMT3A	mutated	c.2385G>A	p.Trp795*	3.90
DNMT3A	mutated	c.2385G>A	p.Trp795*	5.80
DNMT3A	mutated	c.2385G>C	p.Trp795Cys	3.74
DNMT3A	mutated	c.2386G>T	p.Gly796Cys	4.00
DNMT3A	mutated	c.2387del	p.Gly796Valfs*6	2.71
DNMT3A	mutated	c.2387G>A	p.Gly796Asp	4.39
DNMT3A	mutated	c.2389A>C	p.Asn797His	4.28
DNMT3A	mutated	c.2389A>G	p.Asn797Asp	3.46
DNMT3A	mutated	c.2389A>G	p.Asn797Asp	11.90
DNMT3A	mutated	c.2390A>G	p.Asn797Ser	2.48
DNMT3A	mutated	c.2390A>T	p.Asn797Ile	4.35
DNMT3A	mutated	c.2392C>T	p.Leu798Phe	2.48
DNMT3A	mutated	c.2392del	p.Leu798Phefs*4	2.97
DNMT3A	mutated	c.2392del	p.Leu798Phefs*4	6.80
DNMT3A	mutated	c.2393T>C	p.Leu798Pro	4.64
DNMT3A	mutated	c.2393T>C	p.Leu798Pro	18.40

DNMT3A	mutated	c.2393T>G	p.Leu798Arg	3.19
DNMT3A	mutated	c.2394_2404del	p.Pro799Glnfs*9	2.77
DNMT3A	mutated	c.2395C>G	p.Pro799Ala	2.07
DNMT3A	mutated	c.2395C>G	p.Pro799Ala	2.31
DNMT3A	mutated	c.2395C>G	p.Pro799Ala	3.70
DNMT3A	mutated	c.2395C>G	p.Pro799Ala	11.10
DNMT3A	mutated	c.2395C>T	p.Pro799Ser	2.19
DNMT3A	mutated	c.2396C>A	p.Pro799His	2.92
DNMT3A	mutated	c.2398G>A	p.Gly800Ser	7.10
DNMT3A	mutated	c.2401A>G	p.Met801Val	2.61
DNMT3A	mutated	c.2401A>G	p.Met801Val	3.00
DNMT3A	mutated	c.2401A>G	p.Met801Val	5.40
DNMT3A	mutated	c.2401A>G	p.Met801Val	6.10
DNMT3A	mutated	c.2401A>G	p.Met801Val	6.60
DNMT3A	mutated	c.2401dup	p.Met801Asnfs*11	5.10
DNMT3A	mutated	c.2402T>C	p.Met801Thr	3.62
DNMT3A	mutated	c.2402T>C	p.Met801Thr	4.05
DNMT3A	mutated	c.2402T>C	p.Met801Thr	16.20
DNMT3A	mutated	c.2402T>G	p.Met801Arg	17.10
DNMT3A	mutated	c.2403_2407delinsACG	p.Met801Ilefs*10	4.27
DNMT3A	mutated	c.2403G>C	p.Met801Ile	2.44
DNMT3A	mutated	c.2403G>C	p.Met801Ile	5.60
DNMT3A	mutated	c.2407A>G	p.Arg803Gly	3.17
DNMT3A	mutated	c.2407A>G	p.Arg803Gly	3.54
DNMT3A	mutated	c.2408G>A	p.Arg803Lys	4.47
DNMT3A	mutated	c.2408G>C	p.Arg803Thr	19.50
DNMT3A	mutated	c.2409-6_2422delinsATG	p.splice site mutation	21.00
DNMT3A	mutated	c.2409del	p.Pro804Argfs*6	3.30
DNMT3A	mutated	c.2411C>T	p.Pro804Leu	3.33
DNMT3A	mutated	c.2411C>T	p.Pro804Leu	3.56
DNMT3A	mutated	c.2412_2413del	p.Leu805Glyfs*6	2.83
DNMT3A	mutated	c.2414T>A	p.Leu805*	2.82
DNMT3A	mutated	c.2418del	p.Ser807Profs*3	8.90
DNMT3A	mutated	c.2421del	p.Thr808Leufs*2	6.30
DNMT3A	mutated	c.2433dup	p.Lys812*	21.50
DNMT3A	mutated	c.2435_2459dup	p.His821Alafs*42	2.90
DNMT3A	mutated	c.2446C>T	p.Gln816*	3.00
DNMT3A	mutated	c.2446C>T	p.Gln816*	4.80
DNMT3A	mutated	c.2446C>T	p.Gln816*	22.20
DNMT3A	mutated	c.2447A>C	p.Gln816Pro	7.00
DNMT3A	mutated	c.2462A>G	p.His821Arg	2.65
DNMT3A	mutated	c.2462A>G	p.His821Arg	10.10
DNMT3A	mutated	c.2468G>T	p.Arg823Met	2.82
DNMT3A	mutated	c.2470del	p.Ile824*	2.08
DNMT3A	mutated	c.2475del	p.Lys826Serfs*5	6.90
DNMT3A	mutated	c.2477A>G	p.Lys826Arg	3.37
DNMT3A	mutated	c.2477A>G	p.Lys826Arg	5.10

DNMT3A	mutated	c.2477A>G	p.Lys826Arg	6.60
DNMT3A	mutated	c.2477A>G	p.Lys826Arg	31.40
DNMT3A	mutated	c.2478G>C	p.Lys826Asn	2.23
DNMT3A	mutated	c.2478G>T	p.Lys826Asn	3.74
DNMT3A	mutated	c.2481_2487del	p.Phe827Leufs*2	2.98
DNMT3A	mutated	c.2481del	p.Phe827Leufs*4	2.35
DNMT3A	mutated	c.2485A>G	p.Lys829Glu	2.70
DNMT3A	mutated	c.2485A>T	p.Lys829*	28.10
DNMT3A	mutated	c.2487del	p.Val830*	4.05
DNMT3A	mutated	c.2500dup	p.Thr834Asnfs*21	6.70
DNMT3A	mutated	c.2504C>T	p.Thr835Met	4.03
DNMT3A	mutated	c.2505del	p.Arg836Glyfs*5	5.00
DNMT3A	mutated	c.2506A>T	p.Arg836Trp	2.42
DNMT3A	mutated	c.2507G>C	p.Arg836Thr	2.15
DNMT3A	mutated	c.2510C>G	p.Ser837*	3.61
DNMT3A	mutated	c.2512A>G	p.Asn838Asp	2.51
DNMT3A	mutated	c.2512A>G	p.Asn838Asp	5.10
DNMT3A	mutated	c.2516C>T	p.Ser839Phe	4.74
DNMT3A	mutated	c.2517_2524del	p.Ile840Glyfs*12	9.90
DNMT3A	mutated	c.2522_2559del	p.Lys841Argfs*10	5.40
DNMT3A	mutated	c.2524C>T	p.Gln842*	2.89
DNMT3A	mutated	c.2524C>T	p.Gln842*	12.50
DNMT3A	mutated	c.2524del	p.Gln842Argfs*11	10.00
DNMT3A	mutated	c.2525A>G	p.Gln842Arg	2.30
DNMT3A	mutated	c.2525A>G	p.Gln842Arg	3.92
DNMT3A	mutated	c.2525A>G	p.Gln842Arg	4.42
DNMT3A	mutated	c.2525A>G	p.Gln842Arg	5.20
DNMT3A	mutated	c.2528del	p.Gly843Alafs*10	2.63
DNMT3A	mutated	c.2530_2561del	p.Lys844Glufs*9	6.20
DNMT3A	mutated	c.2530A>G	p.Lys844Glu	4.21
DNMT3A	mutated	c.2531A>G	p.Lys844Arg	4.08
DNMT3A	mutated	c.2537_2538insTTGCCCT	p.Gln846Hisfs*11	2.72
DNMT3A	mutated	c.2544del	p.Pro849Leufs*4	2.57
DNMT3A	mutated	c.2544del	p.Pro849Leufs*4	7.50
DNMT3A	mutated	c.2545C>T	p.Pro849Ser	5.70
DNMT3A	mutated	c.2546C>G	p.Pro849Arg	2.90
DNMT3A	mutated	c.2546C>T	p.Pro849Leu	2.76
DNMT3A	mutated	c.2554A>G	p.Met852Val	10.80
DNMT3A	mutated	c.2558del	p.Asn853Metfs*28	3.20
DNMT3A	mutated	c.2560G>T	p.Glu854*	3.03
DNMT3A	mutated	c.2560G>T	p.Glu854*	4.71
DNMT3A	mutated	c.2566del	p.Glu856Argfs*25	2.25
DNMT3A	mutated	c.2576T>A	p.Leu859*	2.27
DNMT3A	mutated	c.2576T>G	p.Leu859*	10.10
DNMT3A	mutated	c.2577A>T	p.Leu859Phe	3.83
DNMT3A	mutated	c.2577dup	p.Trp860Metfs*4	5.50
DNMT3A	mutated	c.2578T>C	p.Trp860Arg	2.03

DNMT3A	mutated	c.2578T>C	p.Trp860Arg	2.06
DNMT3A	mutated	c.2578T>C	p.Trp860Arg	2.22
DNMT3A	mutated	c.2578T>C	p.Trp860Arg	2.67
DNMT3A	mutated	c.2578T>C	p.Trp860Arg	3.06
DNMT3A	mutated	c.2578T>C	p.Trp860Arg	3.99
DNMT3A	mutated	c.2578T>C	p.Trp860Arg	5.30
DNMT3A	mutated	c.2578T>C	p.Trp860Arg	6.10
DNMT3A	mutated	c.2578T>C	p.Trp860Arg	6.10
DNMT3A	mutated	c.2578T>C	p.Trp860Arg	6.40
DNMT3A	mutated	c.2578T>C	p.Trp860Arg	8.90
DNMT3A	mutated	c.2578T>C	p.Trp860Arg	9.80
DNMT3A	mutated	c.2578T>C	p.Trp860Arg	11.00
DNMT3A	mutated	c.2578T>C	p.Trp860Arg	16.60
DNMT3A	mutated	c.2578T>C	p.Trp860Arg	32.80
DNMT3A	mutated	c.2579G>A	p.Trp860*	10.30
DNMT3A	mutated	c.2580G>A	p.Trp860*	2.58
DNMT3A	mutated	c.2580G>A	p.Trp860*	6.20
DNMT3A	mutated	c.2580G>T	p.Trp860Cys	2.82
DNMT3A	mutated	c.2583C>A	p.Cys861*	3.32
DNMT3A	mutated	c.2583C>A	p.Cys861*	4.64
DNMT3A	mutated	c.2587G>T	p.Glu863*	33.00
DNMT3A	mutated	c.2593_2594del	p.Glu865Lysfs*11	2.57
DNMT3A	mutated	c.2593G>T	p.Glu865*	4.13
DNMT3A	mutated	c.2594A>T	p.Glu865Val	4.19
DNMT3A	mutated	c.2595A>C	p.Glu865Asp	4.79
DNMT3A	mutated	c.2596dup	p.Arg866Lysfs*11	8.40
DNMT3A	mutated	c.2598-15_2598delinsT	p.splice site mutation	2.48
DNMT3A	mutated	c.2598-1G>A	p.splice site mutation	4.56
DNMT3A	mutated	c.2598-1G>A	p.splice site mutation	6.70
DNMT3A	mutated	c.2598-1G>A	p.splice site mutation	20.60
DNMT3A	mutated	c.2598-2A>G	p.splice site mutation	2.73
DNMT3A	mutated	c.2598-2A>G	p.splice site mutation	5.70
DNMT3A	mutated	c.2598-2A>G	p.splice site mutation	7.20
DNMT3A	mutated	c.2598-5_2598-1del	p.splice site mutation	22.70
DNMT3A	mutated	c.2599del	p.Val867Tyrfs*14	8.10
DNMT3A	mutated	c.2602T>C	p.Phe868Leu	3.23
DNMT3A	mutated	c.2602T>C	p.Phe868Leu	4.33
DNMT3A	mutated	c.2604del	p.Phe868Leufs*13	4.14
DNMT3A	mutated	c.2605G>A	p.Gly869Ser	2.21
DNMT3A	mutated	c.2606G>T	p.Gly869Val	2.71
DNMT3A	mutated	c.2606G>T	p.Gly869Val	3.01
DNMT3A	mutated	c.2612_2613insT	p.Val872Serfs*5	3.16
DNMT3A	mutated	c.2614del	p.Val872Serfs*9	5.80
DNMT3A	mutated	c.2617del	p.His873Thrfs*8	27.40
DNMT3A	mutated	c.2618A>G	p.His873Arg	3.10
DNMT3A	mutated	c.2621A>G	p.Tyr874Cys	2.14
DNMT3A	mutated	c.2622_2623dup	p.Thr875Ilefs*7	4.00

DNMT3A	mutated	c.2622T>A	p.Tyr874*	4.76
DNMT3A	mutated	c.2622T>A	p.Tyr874*	6.20
DNMT3A	mutated	c.2626G>A	p.Asp876Asn	2.41
DNMT3A	mutated	c.2627A>C	p.Asp876Ala	27.20
DNMT3A	mutated	c.2635A>G	p.Asn879Asp	8.70
DNMT3A	mutated	c.2638A>G	p.Met880Val	5.70
DNMT3A	mutated	c.2644C>A	p.Arg882Ser	2.12
DNMT3A	mutated	c.2644C>A	p.Arg882Ser	2.71
DNMT3A	mutated	c.2644C>A	p.Arg882Ser	3.36
DNMT3A	mutated	c.2644C>A	p.Arg882Ser	8.50
DNMT3A	mutated	c.2644C>A	p.Arg882Ser	11.30
DNMT3A	mutated	c.2644C>T	p.Arg882Cys	2.24
DNMT3A	mutated	c.2644C>T	p.Arg882Cys	2.26
DNMT3A	mutated	c.2644C>T	p.Arg882Cys	2.31
DNMT3A	mutated	c.2644C>T	p.Arg882Cys	2.36
DNMT3A	mutated	c.2644C>T	p.Arg882Cys	2.56
DNMT3A	mutated	c.2644C>T	p.Arg882Cys	2.72
DNMT3A	mutated	c.2644C>T	p.Arg882Cys	2.80
DNMT3A	mutated	c.2644C>T	p.Arg882Cys	2.90
DNMT3A	mutated	c.2644C>T	p.Arg882Cys	2.94
DNMT3A	mutated	c.2644C>T	p.Arg882Cys	3.17
DNMT3A	mutated	c.2644C>T	p.Arg882Cys	3.23
DNMT3A	mutated	c.2644C>T	p.Arg882Cys	3.38
DNMT3A	mutated	c.2644C>T	p.Arg882Cys	3.39
DNMT3A	mutated	c.2644C>T	p.Arg882Cys	3.46
DNMT3A	mutated	c.2644C>T	p.Arg882Cys	3.49
DNMT3A	mutated	c.2644C>T	p.Arg882Cys	3.53
DNMT3A	mutated	c.2644C>T	p.Arg882Cys	3.56
DNMT3A	mutated	c.2644C>T	p.Arg882Cys	3.65
DNMT3A	mutated	c.2644C>T	p.Arg882Cys	3.80
DNMT3A	mutated	c.2644C>T	p.Arg882Cys	3.82
DNMT3A	mutated	c.2644C>T	p.Arg882Cys	4.05
DNMT3A	mutated	c.2644C>T	p.Arg882Cys	4.84
DNMT3A	mutated	c.2644C>T	p.Arg882Cys	5.10
DNMT3A	mutated	c.2644C>T	p.Arg882Cys	5.10
DNMT3A	mutated	c.2644C>T	p.Arg882Cys	5.10
DNMT3A	mutated	c.2644C>T	p.Arg882Cys	5.20
DNMT3A	mutated	c.2644C>T	p.Arg882Cys	5.30
DNMT3A	mutated	c.2644C>T	p.Arg882Cys	5.40
DNMT3A	mutated	c.2644C>T	p.Arg882Cys	5.60
DNMT3A	mutated	c.2644C>T	p.Arg882Cys	5.70
DNMT3A	mutated	c.2644C>T	p.Arg882Cys	5.70
DNMT3A	mutated	c.2644C>T	p.Arg882Cys	5.80
DNMT3A	mutated	c.2644C>T	p.Arg882Cys	6.40
DNMT3A	mutated	c.2644C>T	p.Arg882Cys	7.00
DNMT3A	mutated	c.2644C>T	p.Arg882Cys	7.60
DNMT3A	mutated	c.2644C>T	p.Arg882Cys	7.90

DNMT3A	mutated	c.2644C>T	p.Arg882Cys	8.70
DNMT3A	mutated	c.2644C>T	p.Arg882Cys	9.40
DNMT3A	mutated	c.2644C>T	p.Arg882Cys	10.00
DNMT3A	mutated	c.2644C>T	p.Arg882Cys	11.30
DNMT3A	mutated	c.2644C>T	p.Arg882Cys	12.10
DNMT3A	mutated	c.2644C>T	p.Arg882Cys	12.60
DNMT3A	mutated	c.2644C>T	p.Arg882Cys	13.40
DNMT3A	mutated	c.2644C>T	p.Arg882Cys	14.80
DNMT3A	mutated	c.2644C>T	p.Arg882Cys	14.90
DNMT3A	mutated	c.2644C>T	p.Arg882Cys	18.30
DNMT3A	mutated	c.2644C>T	p.Arg882Cys	19.40
DNMT3A	mutated	c.2644C>T	p.Arg882Cys	19.80
DNMT3A	mutated	c.2644C>T	p.Arg882Cys	24.10
DNMT3A	mutated	c.2644C>T	p.Arg882Cys	26.10
DNMT3A	mutated	c.2644C>T	p.Arg882Cys	32.30
DNMT3A	mutated	c.2644C>T	p.Arg882Cys	33.10
DNMT3A	mutated	c.2644C>T	p.Arg882Cys	41.70
DNMT3A	mutated	c.2645G>A	p.Arg882His	2.00
DNMT3A	mutated	c.2645G>A	p.Arg882His	2.08
DNMT3A	mutated	c.2645G>A	p.Arg882His	2.10
DNMT3A	mutated	c.2645G>A	p.Arg882His	2.11
DNMT3A	mutated	c.2645G>A	p.Arg882His	2.14
DNMT3A	mutated	c.2645G>A	p.Arg882His	2.14
DNMT3A	mutated	c.2645G>A	p.Arg882His	2.20
DNMT3A	mutated	c.2645G>A	p.Arg882His	2.31
DNMT3A	mutated	c.2645G>A	p.Arg882His	2.41
DNMT3A	mutated	c.2645G>A	p.Arg882His	2.42
DNMT3A	mutated	c.2645G>A	p.Arg882His	2.46
DNMT3A	mutated	c.2645G>A	p.Arg882His	2.48
DNMT3A	mutated	c.2645G>A	p.Arg882His	3.02
DNMT3A	mutated	c.2645G>A	p.Arg882His	3.03
DNMT3A	mutated	c.2645G>A	p.Arg882His	3.22
DNMT3A	mutated	c.2645G>A	p.Arg882His	3.65
DNMT3A	mutated	c.2645G>A	p.Arg882His	3.71
DNMT3A	mutated	c.2645G>A	p.Arg882His	4.06
DNMT3A	mutated	c.2645G>A	p.Arg882His	4.06
DNMT3A	mutated	c.2645G>A	p.Arg882His	4.67
DNMT3A	mutated	c.2645G>A	p.Arg882His	4.85
DNMT3A	mutated	c.2645G>A	p.Arg882His	5.00
DNMT3A	mutated	c.2645G>A	p.Arg882His	5.00
DNMT3A	mutated	c.2645G>A	p.Arg882His	5.10
DNMT3A	mutated	c.2645G>A	p.Arg882His	5.20
DNMT3A	mutated	c.2645G>A	p.Arg882His	6.10
DNMT3A	mutated	c.2645G>A	p.Arg882His	6.20
DNMT3A	mutated	c.2645G>A	p.Arg882His	6.20
DNMT3A	mutated	c.2645G>A	p.Arg882His	6.40
DNMT3A	mutated	c.2645G>A	p.Arg882His	6.70

DNMT3A	mutated	c.2645G>A	p.Arg882His	7.20
DNMT3A	mutated	c.2645G>A	p.Arg882His	7.50
DNMT3A	mutated	c.2645G>A	p.Arg882His	8.00
DNMT3A	mutated	c.2645G>A	p.Arg882His	8.40
DNMT3A	mutated	c.2645G>A	p.Arg882His	9.20
DNMT3A	mutated	c.2645G>A	p.Arg882His	9.40
DNMT3A	mutated	c.2645G>A	p.Arg882His	9.50
DNMT3A	mutated	c.2645G>A	p.Arg882His	10.50
DNMT3A	mutated	c.2645G>A	p.Arg882His	10.60
DNMT3A	mutated	c.2645G>A	p.Arg882His	11.40
DNMT3A	mutated	c.2645G>A	p.Arg882His	12.50
DNMT3A	mutated	c.2645G>A	p.Arg882His	12.70
DNMT3A	mutated	c.2645G>A	p.Arg882His	17.40
DNMT3A	mutated	c.2645G>A	p.Arg882His	17.50
DNMT3A	mutated	c.2645G>A	p.Arg882His	18.30
DNMT3A	mutated	c.2645G>A	p.Arg882His	19.90
DNMT3A	mutated	c.2645G>A	p.Arg882His	22.10
DNMT3A	mutated	c.2645G>A	p.Arg882His	25.10
DNMT3A	mutated	c.2645G>A	p.Arg882His	26.70
DNMT3A	mutated	c.2645G>A	p.Arg882His	28.70
DNMT3A	mutated	c.2645G>A	p.Arg882His	28.70
DNMT3A	mutated	c.2645G>A	p.Arg882His	30.00
DNMT3A	mutated	c.2645G>C	p.Arg882Pro	4.02
DNMT3A	mutated	c.2645G>T	p.Arg882Leu	5.40
DNMT3A	mutated	c.2646_2730del	p.Leu883Valfs*11	12.00
DNMT3A	mutated	c.2648T>A	p.Leu883*	6.30
DNMT3A	mutated	c.2651C>T	p.Ala884Val	11.10
DNMT3A	mutated	c.2653A>T	p.Arg885Trp	28.70
DNMT3A	mutated	c.2663T>C	p.Leu888Pro	11.60
DNMT3A	mutated	c.2668G>A	p.Gly890Ser	17.20
DNMT3A	mutated	c.2673del	p.Ser892Hisfs*14	6.40
DNMT3A	mutated	c.2675C>A	p.Ser892*	17.50
DNMT3A	mutated	c.2675C>T	p.Ser892Leu	2.98
DNMT3A	mutated	c.2677del	p.Trp893Glyfs*13	8.80
DNMT3A	mutated	c.2677T>C	p.Trp893Arg	30.90
DNMT3A	mutated	c.2678G>A	p.Trp893*	2.01
DNMT3A	mutated	c.2678G>C	p.Trp893Ser	27.20
DNMT3A	mutated	c.2679G>A	p.Trp893*	2.08
DNMT3A	mutated	c.2690T>A	p.Val897Asp	2.79
DNMT3A	mutated	c.2693T>C	p.Ile898Thr	4.52
DNMT3A	mutated	c.2695C>G	p.Arg899Gly	3.84
DNMT3A	mutated	c.2695C>T	p.Arg899Cys	2.04
DNMT3A	mutated	c.2695C>T	p.Arg899Cys	2.73
DNMT3A	mutated	c.2695C>T	p.Arg899Cys	2.98
DNMT3A	mutated	c.2695C>T	p.Arg899Cys	3.73
DNMT3A	mutated	c.2695C>T	p.Arg899Cys	4.51
DNMT3A	mutated	c.2695C>T	p.Arg899Cys	4.58

DNMT3A	mutated	c.2695C>T	p.Arg899Cys	8.00
DNMT3A	mutated	c.2695C>T	p.Arg899Cys	10.40
DNMT3A	mutated	c.2695C>T	p.Arg899Cys	19.60
DNMT3A	mutated	c.2695del	p.Arg899Alafs*7	2.49
DNMT3A	mutated	c.2695del	p.Arg899Alafs*7	8.80
DNMT3A	mutated	c.2696_2697delinsCA	p.Arg899Pro	12.00
DNMT3A	mutated	c.2696G>A	p.Arg899His	2.40
DNMT3A	mutated	c.2701C>G	p.Leu901Val	2.06
DNMT3A	mutated	c.2701C>G	p.Leu901Val	3.08
DNMT3A	mutated	c.2702T>C	p.Leu901Pro	4.05
DNMT3A	mutated	c.2702T>G	p.Leu901Arg	5.60
DNMT3A	mutated	c.2704_2706del	p.Phe902del	2.12
DNMT3A	mutated	c.2705_2706del	p.Phe902Cysfs*18	14.80
DNMT3A	mutated	c.2706del	p.Phe902Leufs*4	2.07
DNMT3A	mutated	c.2707G>C	p.Ala903Pro	10.30
DNMT3A	mutated	c.2710_2714del	p.Pro904Glufs*15	2.17
DNMT3A	mutated	c.2710C>A	p.Pro904Thr	2.74
DNMT3A	mutated	c.2710C>T	p.Pro904Ser	2.26
DNMT3A	mutated	c.2711C>A	p.Pro904Gln	3.09
DNMT3A	mutated	c.2711C>A	p.Pro904Gln	8.20
DNMT3A	mutated	c.2711C>G	p.Pro904Arg	4.14
DNMT3A	mutated	c.2711C>T	p.Pro904Leu	2.06
DNMT3A	mutated	c.2711C>T	p.Pro904Leu	2.16
DNMT3A	mutated	c.2711C>T	p.Pro904Leu	2.29
DNMT3A	mutated	c.2711C>T	p.Pro904Leu	2.43
DNMT3A	mutated	c.2711C>T	p.Pro904Leu	2.55
DNMT3A	mutated	c.2711C>T	p.Pro904Leu	2.77
DNMT3A	mutated	c.2711C>T	p.Pro904Leu	3.40
DNMT3A	mutated	c.2711C>T	p.Pro904Leu	4.55
DNMT3A	mutated	c.2711C>T	p.Pro904Leu	5.20
DNMT3A	mutated	c.2711C>T	p.Pro904Leu	5.90
DNMT3A	mutated	c.2711C>T	p.Pro904Leu	6.50
DNMT3A	mutated	c.2711C>T	p.Pro904Leu	6.50
DNMT3A	mutated	c.2711C>T	p.Pro904Leu	9.20
DNMT3A	mutated	c.2711C>T	p.Pro904Leu	10.30
DNMT3A	mutated	c.2711C>T	p.Pro904Leu	12.00
DNMT3A	mutated	c.2711C>T	p.Pro904Leu	16.30
DNMT3A	mutated	c.2711C>T	p.Pro904Leu	20.50
DNMT3A	mutated	c.2711C>T	p.Pro904Leu	25.60
DNMT3A	mutated	c.2714T>A	p.Leu905Gln	11.10
DNMT3A	mutated	c.2714T>C	p.Leu905Pro	4.41
DNMT3A	mutated	c.2718_2719delinsTT	p.Lys906_Glu907delinsAsn*	4.30
DNMT3A	mutated	c.2722T>G	p.Tyr908Asp	3.53
DNMT3A	mutated	c.2723A>G	p.Tyr908Cys	2.05
DNMT3A	mutated	c.2723A>G	p.Tyr908Cys	2.09
DNMT3A	mutated	c.2723A>G	p.Tyr908Cys	2.49
DNMT3A	mutated	c.2723A>G	p.Tyr908Cys	3.81

DNMT3A	mutated	c.2723A>G	p.Tyr908Cys	5.50
DNMT3A	mutated	c.2723A>G	p.Tyr908Cys	12.90
DNMT3A	mutated	c.2723del	p.Tyr908Phefs*14	11.00
DNMT3A	mutated	c.2725T>G	p.Phe909Val	3.98
DNMT3A	mutated	c.2726T>C	p.Phe909Ser	2.14
DNMT3A	mutated	c.2728G>C	p.Ala910Pro	3.34
DNMT3A	mutated	c.2728G>C	p.Ala910Pro	5.00
DNMT3A	mutated	c.2728G>C	p.Ala910Pro	5.80
DNMT3A	mutated	c.2729C>T	p.Ala910Val	9.00
DNMT3A	mutated	c.673_676del	p.Asn225Leufs*90	9.80
DNMT3A	mutated	c.691C>T	p.Gln231*	3.17
DNMT3A	mutated	c.691C>T	p.Gln231*	4.01
DNMT3A	mutated	c.691del	p.Gln231Argfs*85	12.40
DNMT3A	mutated	c.703del	p.Glu235Serfs*81	3.46
DNMT3A	mutated	c.703del	p.Glu235Serfs*81	3.57
DNMT3A	mutated	c.703G>T	p.Glu235*	2.16
DNMT3A	mutated	c.703G>T	p.Glu235*	9.50
DNMT3A	mutated	c.709C>T	p.Gln237*	2.53
DNMT3A	mutated	c.731del	p.Pro244Leufs*72	4.10
DNMT3A	mutated	c.733_734del	p.Pro245Cysfs*7	8.80
DNMT3A	mutated	c.742C>T	p.Gln248*	2.27
DNMT3A	mutated	c.753_759dup	p.Ala254*	14.70
DNMT3A	mutated	c.774_775insTAGC	p.Ala259*	2.61
DNMT3A	mutated	c.780_786del	p.Thr261Serfs*53	12.00
DNMT3A	mutated	c.787G>T	p.Glu263*	5.20
DNMT3A	mutated	c.787G>T	p.Glu263*	5.30
DNMT3A	mutated	c.792del	p.Val265Trpfs*51	4.15
DNMT3A	mutated	c.7G>A	p.Ala3Thr	41.80
DNMT3A	mutated	c.800_804dup	p.Ala269Profs*49	4.34
DNMT3A	mutated	c.805dup	p.Ala269Glyfs*12	17.60
DNMT3A	mutated	c.820del	p.Ala274Profs*42	7.10
DNMT3A	mutated	c.846dup	p.Glu283Argfs*41	4.16
DNMT3A	mutated	c.849del	p.Glu283Aspfs*33	31.70
DNMT3A	mutated	c.866del	p.Gly289Alafs*27	9.50
DNMT3A	mutated	c.872_886del	p.Gly291_Leu295del	11.00
DNMT3A	mutated	c.876del	p.Ile292Metfs*24	3.70
DNMT3A	mutated	c.878_888del	p.Gly293Valfs*27	6.80
DNMT3A	mutated	c.878G>T	p.Gly293Val	5.90
DNMT3A	mutated	c.880del	p.Glu294Serfs*22	5.80
DNMT3A	mutated	c.880G>T	p.Glu294*	4.82
DNMT3A	mutated	c.880G>T	p.Glu294*	10.00
DNMT3A	mutated	c.884T>A	p.Leu295Gln	2.56
DNMT3A	mutated	c.884T>A	p.Leu295Gln	5.00
DNMT3A	mutated	c.886del	p.Val296Cysfs*20	2.64
DNMT3A	mutated	c.886G>A	p.Val296Met	3.74
DNMT3A	mutated	c.886G>A	p.Val296Met	7.90
DNMT3A	mutated	c.886G>A	p.Val296Met	21.40

DNMT3A	mutated	c.886G>A	p.Val296Met	34.90
DNMT3A	mutated	c.886G>C	p.Val296Leu	3.19
DNMT3A	mutated	c.886G>T	p.Val296Leu	2.55
DNMT3A	mutated	c.886G>T	p.Val296Leu	3.30
DNMT3A	mutated	c.886G>T	p.Val296Leu	8.20
DNMT3A	mutated	c.886G>T	p.Val296Leu	16.90
DNMT3A	mutated	c.887T>G	p.Val296Gly	9.20
DNMT3A	mutated	c.890G>A	p.Trp297*	2.15
DNMT3A	mutated	c.890G>A	p.Trp297*	2.94
DNMT3A	mutated	c.890G>A	p.Trp297*	9.50
DNMT3A	mutated	c.890G>A	p.Trp297*	15.30
DNMT3A	mutated	c.890G>C	p.Trp297Ser	12.70
DNMT3A	mutated	c.890G>C	p.Trp297Ser	13.50
DNMT3A	mutated	c.890G>C	p.Trp297Ser	17.10
DNMT3A	mutated	c.891G>A	p.Trp297*	2.67
DNMT3A	mutated	c.891G>A	p.Trp297*	5.40
DNMT3A	mutated	c.891G>A	p.Trp297*	13.90
DNMT3A	mutated	c.892G>A	p.Gly298Arg	3.57
DNMT3A	mutated	c.893G>A	p.Gly298Glu	7.60
DNMT3A	mutated	c.893G>A	p.Gly298Glu	21.40
DNMT3A	mutated	c.894del	p.Lys299Asnfs*17	2.38
DNMT3A	mutated	c.899T>A	p.Leu300Gln	2.20
DNMT3A	mutated	c.901C>T	p.Arg301Trp	2.50
DNMT3A	mutated	c.901C>T	p.Arg301Trp	2.97
DNMT3A	mutated	c.901C>T	p.Arg301Trp	5.70
DNMT3A	mutated	c.905del	p.Gly302Alafs*14	2.05
DNMT3A	mutated	c.905del	p.Gly302Alafs*14	13.50
DNMT3A	mutated	c.905G>A	p.Gly302Asp	3.69
DNMT3A	mutated	c.905G>A	p.Gly302Asp	5.30
DNMT3A	mutated	c.911C>A	p.Ser304Tyr	3.86
DNMT3A	mutated	c.912del	p.Trp305Glyfs*11	3.09
DNMT3A	mutated	c.912del	p.Trp305Glyfs*11	4.42
DNMT3A	mutated	c.914G>A	p.Trp305*	2.01
DNMT3A	mutated	c.915G>A	p.Trp305*	2.32
DNMT3A	mutated	c.915G>A	p.Trp305*	2.51
DNMT3A	mutated	c.915G>A	p.Trp305*	2.55
DNMT3A	mutated	c.915G>A	p.Trp305*	3.24
DNMT3A	mutated	c.915G>A	p.Trp305*	3.49
DNMT3A	mutated	c.915G>A	p.Trp305*	3.53
DNMT3A	mutated	c.915G>A	p.Trp305*	3.68
DNMT3A	mutated	c.915G>A	p.Trp305*	5.60
DNMT3A	mutated	c.915G>A	p.Trp305*	6.40
DNMT3A	mutated	c.915G>A	p.Trp305*	18.80
DNMT3A	mutated	c.915G>A	p.Trp305*	24.10
DNMT3A	mutated	c.917_918insCAG	p.Trp306delinsCysArg	4.59
DNMT3A	mutated	c.917G>A	p.Trp306*	3.15
DNMT3A	mutated	c.917G>A	p.Trp306*	4.08

DNMT3A	mutated	c.918_919insGAAGC	p.Pro307Glufs*11	3.42
DNMT3A	mutated	c.918G>A	p.Trp306*	3.72
DNMT3A	mutated	c.918G>A	p.Trp306*	6.30
DNMT3A	mutated	c.918G>T	p.Trp306Cys	5.20
DNMT3A	mutated	c.919C>A	p.Pro307Thr	2.59
DNMT3A	mutated	c.919C>T	p.Pro307Ser	2.44
DNMT3A	mutated	c.920C>A	p.Pro307Gln	5.60
DNMT3A	mutated	c.920C>A	p.Pro307Gln	5.70
DNMT3A	mutated	c.920C>G	p.Pro307Arg	2.23
DNMT3A	mutated	c.920C>G	p.Pro307Arg	7.40
DNMT3A	mutated	c.920C>T	p.Pro307Leu	2.94
DNMT3A	mutated	c.920C>T	p.Pro307Leu	5.40
DNMT3A	mutated	c.920C>T	p.Pro307Leu	5.90
DNMT3A	mutated	c.920C>T	p.Pro307Leu	18.20
DNMT3A	mutated	c.920C>T	p.Pro307Leu	23.20
DNMT3A	mutated	c.926G>A	p.Arg309His	9.00
DNMT3A	mutated	c.928A>C	p.Ile310Leu	22.50
DNMT3A	mutated	c.928A>T	p.Ile310Phe	6.00
DNMT3A	mutated	c.928dup	p.Ile310Asnfs*14	2.66
DNMT3A	mutated	c.929T>A	p.Ile310Asn	4.79
DNMT3A	mutated	c.929T>A	p.Ile310Asn	8.80
DNMT3A	mutated	c.929T>C	p.Ile310Thr	3.03
DNMT3A	mutated	c.930T>G	p.Ile310Met	7.70
DNMT3A	mutated	c.935C>A	p.Ser312Tyr	2.23
DNMT3A	mutated	c.935C>T	p.Ser312Phe	2.80
DNMT3A	mutated	c.938G>A	p.Trp313*	2.71
DNMT3A	mutated	c.940_941dup	p.Trp314Cysfs*3	2.13
DNMT3A	mutated	c.941G>A	p.Trp314*	2.05
DNMT3A	mutated	c.941G>A	p.Trp314*	3.99
DNMT3A	mutated	c.941G>A	p.Trp314*	5.90
DNMT3A	mutated	c.941G>A	p.Trp314*	7.50
DNMT3A	mutated	c.942_959del	p.Trp314*	6.30
DNMT3A	mutated	c.942G>A	p.Trp314*	12.50
DNMT3A	mutated	c.952del	p.Arg318Glyfs*27	2.11
DNMT3A	mutated	c.954del	p.Ser319Alafs*26	12.20
DNMT3A	mutated	c.958C>T	p.Arg320*	2.11
DNMT3A	mutated	c.958C>T	p.Arg320*	2.64
DNMT3A	mutated	c.958C>T	p.Arg320*	3.20
DNMT3A	mutated	c.958C>T	p.Arg320*	3.35
DNMT3A	mutated	c.958C>T	p.Arg320*	5.00
DNMT3A	mutated	c.958C>T	p.Arg320*	5.00
DNMT3A	mutated	c.958C>T	p.Arg320*	5.40
DNMT3A	mutated	c.958C>T	p.Arg320*	7.50
DNMT3A	mutated	c.958C>T	p.Arg320*	8.00
DNMT3A	mutated	c.958C>T	p.Arg320*	11.00
DNMT3A	mutated	c.958C>T	p.Arg320*	13.10
DNMT3A	mutated	c.959G>A	p.Arg320Gln	2.23

DNMT3A	mutated	c.976C>A	p.Arg326Ser	2.57
DNMT3A	mutated	c.976C>A	p.Arg326Ser	2.67
DNMT3A	mutated	c.976C>T	p.Arg326Cys	2.02
DNMT3A	mutated	c.976C>T	p.Arg326Cys	2.04
DNMT3A	mutated	c.976C>T	p.Arg326Cys	2.23
DNMT3A	mutated	c.976C>T	p.Arg326Cys	2.79
DNMT3A	mutated	c.976C>T	p.Arg326Cys	2.98
DNMT3A	mutated	c.976C>T	p.Arg326Cys	3.09
DNMT3A	mutated	c.976C>T	p.Arg326Cys	3.25
DNMT3A	mutated	c.976C>T	p.Arg326Cys	3.29
DNMT3A	mutated	c.976C>T	p.Arg326Cys	5.40
DNMT3A	mutated	c.976C>T	p.Arg326Cys	5.50
DNMT3A	mutated	c.976C>T	p.Arg326Cys	5.50
DNMT3A	mutated	c.976C>T	p.Arg326Cys	7.60
DNMT3A	mutated	c.976C>T	p.Arg326Cys	8.50
DNMT3A	mutated	c.976C>T	p.Arg326Cys	11.70
DNMT3A	mutated	c.976C>T	p.Arg326Cys	12.80
DNMT3A	mutated	c.976C>T	p.Arg326Cys	18.00
DNMT3A	mutated	c.976C>T	p.Arg326Cys	18.00
DNMT3A	mutated	c.977G>A	p.Arg326His	2.16
DNMT3A	mutated	c.977G>A	p.Arg326His	2.74
DNMT3A	mutated	c.977G>A	p.Arg326His	2.85
DNMT3A	mutated	c.977G>A	p.Arg326His	2.90
DNMT3A	mutated	c.977G>A	p.Arg326His	8.70
DNMT3A	mutated	c.977G>C	p.Arg326Pro	5.00
DNMT3A	mutated	c.977G>T	p.Arg326Leu	3.57
DNMT3A	mutated	c.983T>A	p.Val328Asp	7.60
DNMT3A	mutated	c.983T>G	p.Val328Gly	3.80
DNMT3A	mutated	c.989G>A	p.Trp330*	5.60
DNMT3A	mutated	c.989G>A	p.Trp330*	8.10
DNMT3A	mutated	c.990G>A	p.Trp330*	3.04
DNMT3A	mutated	c.990G>A	p.Trp330*	3.05
DNMT3A	mutated	c.991T>G	p.Phe331Val	12.20
DNMT3A	mutated	c.994G>A	p.Gly332Arg	2.38
DNMT3A	mutated	c.994G>C	p.Gly332Arg	5.40
DNMT3A	mutated	c.996_997delinsC	p.Asp333Thrfs*12	24.00
JAK2	mutated	c.1611_1616del	p.Phe537_Lys539delinsLeu	13.00
JAK2	mutated	c.1666T>G	p.Phe556Val	41.30
JAK2	mutated	c.1666T>G	p.Phe556Val	45.80
JAK2	mutated	c.1666T>G	p.Phe556Val	47.10
JAK2	mutated	c.1849G>T	p.Val617Phe	2.06
JAK2	mutated	c.1849G>T	p.Val617Phe	2.11
JAK2	mutated	c.1849G>T	p.Val617Phe	2.13
JAK2	mutated	c.1849G>T	p.Val617Phe	2.37
JAK2	mutated	c.1849G>T	p.Val617Phe	2.44
JAK2	mutated	c.1849G>T	p.Val617Phe	2.58
JAK2	mutated	c.1849G>T	p.Val617Phe	2.81

JAK2	mutated	c.1849G>T	p.Val617Phe	3.19
JAK2	mutated	c.1849G>T	p.Val617Phe	3.23
JAK2	mutated	c.1849G>T	p.Val617Phe	3.24
JAK2	mutated	c.1849G>T	p.Val617Phe	3.34
JAK2	mutated	c.1849G>T	p.Val617Phe	3.66
JAK2	mutated	c.1849G>T	p.Val617Phe	3.84
JAK2	mutated	c.1849G>T	p.Val617Phe	3.93
JAK2	mutated	c.1849G>T	p.Val617Phe	4.08
JAK2	mutated	c.1849G>T	p.Val617Phe	4.42
JAK2	mutated	c.1849G>T	p.Val617Phe	4.45
JAK2	mutated	c.1849G>T	p.Val617Phe	4.51
JAK2	mutated	c.1849G>T	p.Val617Phe	4.63
JAK2	mutated	c.1849G>T	p.Val617Phe	4.76
JAK2	mutated	c.1849G>T	p.Val617Phe	5.30
JAK2	mutated	c.1849G>T	p.Val617Phe	5.30
JAK2	mutated	c.1849G>T	p.Val617Phe	5.40
JAK2	mutated	c.1849G>T	p.Val617Phe	5.50
JAK2	mutated	c.1849G>T	p.Val617Phe	5.60
JAK2	mutated	c.1849G>T	p.Val617Phe	5.60
JAK2	mutated	c.1849G>T	p.Val617Phe	5.90
JAK2	mutated	c.1849G>T	p.Val617Phe	6.00
JAK2	mutated	c.1849G>T	p.Val617Phe	6.10
JAK2	mutated	c.1849G>T	p.Val617Phe	6.50
JAK2	mutated	c.1849G>T	p.Val617Phe	6.50
JAK2	mutated	c.1849G>T	p.Val617Phe	6.50
JAK2	mutated	c.1849G>T	p.Val617Phe	6.60
JAK2	mutated	c.1849G>T	p.Val617Phe	6.60
JAK2	mutated	c.1849G>T	p.Val617Phe	6.70
JAK2	mutated	c.1849G>T	p.Val617Phe	6.80
JAK2	mutated	c.1849G>T	p.Val617Phe	6.80
JAK2	mutated	c.1849G>T	p.Val617Phe	7.50
JAK2	mutated	c.1849G>T	p.Val617Phe	7.50
JAK2	mutated	c.1849G>T	p.Val617Phe	8.00
JAK2	mutated	c.1849G>T	p.Val617Phe	9.30
JAK2	mutated	c.1849G>T	p.Val617Phe	9.70
JAK2	mutated	c.1849G>T	p.Val617Phe	10.10
JAK2	mutated	c.1849G>T	p.Val617Phe	10.40
JAK2	mutated	c.1849G>T	p.Val617Phe	10.50
JAK2	mutated	c.1849G>T	p.Val617Phe	10.80
JAK2	mutated	c.1849G>T	p.Val617Phe	11.00
JAK2	mutated	c.1849G>T	p.Val617Phe	11.40
JAK2	mutated	c.1849G>T	p.Val617Phe	15.00
JAK2	mutated	c.1849G>T	p.Val617Phe	16.60
JAK2	mutated	c.1849G>T	p.Val617Phe	17.80
JAK2	mutated	c.1849G>T	p.Val617Phe	21.60
JAK2	mutated	c.1849G>T	p.Val617Phe	22.40
JAK2	mutated	c.1849G>T	p.Val617Phe	22.80

JAK2	mutated	c.1849G>T	p.Val617Phe	23.70
JAK2	mutated	c.1849G>T	p.Val617Phe	24.00
JAK2	mutated	c.1849G>T	p.Val617Phe	24.20
JAK2	mutated	c.1849G>T	p.Val617Phe	25.50
JAK2	mutated	c.1849G>T	p.Val617Phe	25.80
JAK2	mutated	c.1849G>T	p.Val617Phe	25.90
JAK2	mutated	c.1849G>T	p.Val617Phe	26.20
JAK2	mutated	c.1849G>T	p.Val617Phe	27.40
JAK2	mutated	c.1849G>T	p.Val617Phe	33.20
JAK2	mutated	c.1849G>T	p.Val617Phe	33.70
JAK2	mutated	c.1849G>T	p.Val617Phe	36.40
JAK2	mutated	c.1849G>T	p.Val617Phe	38.40
JAK2	mutated	c.1849G>T	p.Val617Phe	39.50
JAK2	mutated	c.1849G>T	p.Val617Phe	41.90
JAK2	mutated	c.1849G>T	p.Val617Phe	48.50
JAK2	mutated	c.2569A>G	p.Lys857Glu	2.11
JAK2	mutated	c.3151A>G	p.Ile1051Val	2.33
JAK2	mutated	c.515A>G	p.His172Arg	6.80
JAK2	mutated	c.9G>A	p.Met3Ile	2.77
MPL	mutated	c.1040del	p.Pro347Hisfs*22	38.00
MPL	mutated	c.1069C>T	p.Arg357*	40.40
MPL	mutated	c.10T>A	p.Trp4Arg	11.00
MPL	mutated	c.1512_1529del	p.Ser505_Leu510del	26.20
MPL	mutated	c.1544G>T	p.Trp515Leu	4.10
MPL	mutated	c.1694C>T	p.Pro565Leu	2.80
MPL	mutated	c.1694C>T	p.Pro565Leu	6.10
MPL	mutated	c.1771T>A	p.Tyr591Asn	5.00
MPL	mutated	c.1774C>T	p.Arg592*	2.59
MPL	mutated	c.1774C>T	p.Arg592*	43.00
MPL	mutated	c.1775G>A	p.Arg592Gln	3.90
MPL	mutated	c.1775G>A	p.Arg592Gln	27.80
MPL	mutated	c.1775G>A	p.Arg592Gln	42.10
MPL	mutated	c.1775G>A	p.Arg592Gln	44.60
MPL	mutated	c.1775G>A	p.Arg592Gln	44.90
MPL	mutated	c.1775G>A	p.Arg592Gln	45.60
MPL	mutated	c.230del	p.Cys77Serfs*3	39.50
MPL	mutated	c.230del	p.Cys77Serfs*3	39.90
MPL	mutated	c.230del	p.Cys77Serfs*3	41.50
MPL	mutated	c.356C>T	p.Thr119Ile	2.64
MPL	mutated	c.408dup	p.Ser137Glnfs*27	40.70
MPL	mutated	c.4C>G	p.Pro2Ala	3.37
MPL	mutated	c.4C>T	p.Pro2Ser	3.39
MPL	mutated	c.4C>T	p.Pro2Ser	16.80
MPL	mutated	c.629del	p.Pro210Hisfs*25	40.00
MPL	mutated	c.816G>A	p.Trp272*	41.90
MPL	mutated	c.882_888del	p.Asp295Argfs*72	34.60
PPM1D	mutated	c.1216del	p.Thr406Profs*3	4.12

PPM1D	mutated	c.1216dup	p.Thr406Asnfs*28	3.17
PPM1D	mutated	c.1219_1238del	p.Cys407Metfs*20	8.30
PPM1D	mutated	c.1236delinsAA	p.Pro413Thrfs*21	24.00
PPM1D	mutated	c.1258A>T	p.Lys420*	2.22
PPM1D	mutated	c.1262C>A	p.Ser421*	4.86
PPM1D	mutated	c.1270G>T	p.Glu424*	2.23
PPM1D	mutated	c.1270G>T	p.Glu424*	4.04
PPM1D	mutated	c.1270G>T	p.Glu424*	9.20
PPM1D	mutated	c.1273del	p.Asp425Ilefs*6	3.60
PPM1D	mutated	c.1274del	p.Asp425Valfs*6	4.08
PPM1D	mutated	c.1280_1296del	p.Trp427*	3.31
PPM1D	mutated	c.1280G>A	p.Trp427*	3.01
PPM1D	mutated	c.1280G>A	p.Trp427*	3.18
PPM1D	mutated	c.1280G>A	p.Trp427*	3.24
PPM1D	mutated	c.1280G>A	p.Trp427*	3.75
PPM1D	mutated	c.1280G>A	p.Trp427*	4.63
PPM1D	mutated	c.1280G>A	p.Trp427*	9.40
PPM1D	mutated	c.1281G>A	p.Trp427*	4.72
PPM1D	mutated	c.1281G>A	p.Trp427*	5.00
PPM1D	mutated	c.1281G>A	p.Trp427*	8.00
PPM1D	mutated	c.1337C>G	p.Ser446*	3.17
PPM1D	mutated	c.1337C>G	p.Ser446*	5.10
PPM1D	mutated	c.1337C>G	p.Ser446*	5.60
PPM1D	mutated	c.1337C>G	p.Ser446*	6.00
PPM1D	mutated	c.1340del	p.Glu447Glyfs*4	2.34
PPM1D	mutated	c.1347_1348delinsA	p.Phe449Leufs*2	2.63
PPM1D	mutated	c.1349del	p.Leu450*	2.08
PPM1D	mutated	c.1349del	p.Leu450*	2.33
PPM1D	mutated	c.1349del	p.Leu450*	2.62
PPM1D	mutated	c.1349del	p.Leu450*	2.98
PPM1D	mutated	c.1349del	p.Leu450*	3.51
PPM1D	mutated	c.1349del	p.Leu450*	4.34
PPM1D	mutated	c.1349T>G	p.Leu450*	4.10
PPM1D	mutated	c.1354del	p.Val452Phefs*5	3.47
PPM1D	mutated	c.1358C>A	p.Ser453*	5.40
PPM1D	mutated	c.1368del	p.Ile456Metfs*9	2.66
PPM1D	mutated	c.1372C>T	p.Arg458*	3.08
PPM1D	mutated	c.1372C>T	p.Arg458*	5.30
PPM1D	mutated	c.1372C>T	p.Arg458*	7.60
PPM1D	mutated	c.1372C>T	p.Arg458*	12.30
PPM1D	mutated	c.1383_1387del	p.Gln462Cysfs*12	3.53
PPM1D	mutated	c.1384C>T	p.Gln462*	2.01
PPM1D	mutated	c.1384C>T	p.Gln462*	3.68
PPM1D	mutated	c.1384C>T	p.Gln462*	4.82
PPM1D	mutated	c.1384C>T	p.Gln462*	5.10
PPM1D	mutated	c.1384C>T	p.Gln462*	9.30
PPM1D	mutated	c.1384C>T	p.Gln462*	10.00

PPM1D	mutated	c.1384C>T	p.Gln462*	12.30
PPM1D	mutated	c.1384dup	p.Gln462Profs*14	2.12
PPM1D	mutated	c.1388del	p.Gly463Valfs*2	9.30
PPM1D	mutated	c.1403C>G	p.Ser468*	2.82
PPM1D	mutated	c.1403C>G	p.Ser468*	6.40
PPM1D	mutated	c.1410_1438del	p.Asp470Glufs*9	15.00
PPM1D	mutated	c.1410del	p.Pro471Glnfs*12	2.88
PPM1D	mutated	c.1414G>T	p.Glu472*	2.19
PPM1D	mutated	c.1414G>T	p.Glu472*	4.90
PPM1D	mutated	c.1418del	p.Pro473Hisfs*10	10.80
PPM1D	mutated	c.1418dup	p.Leu474Thrfs*2	3.54
PPM1D	mutated	c.1423del	p.Glu475Lysfs*8	25.10
PPM1D	mutated	c.1423G>T	p.Glu475*	4.66
PPM1D	mutated	c.1425del	p.Glu476Lysfs*7	5.80
PPM1D	mutated	c.1432_1433delinsC	p.Cys478Profs*5	3.30
PPM1D	mutated	c.1432del	p.Cys478Alafs*5	2.88
PPM1D	mutated	c.1432del	p.Cys478Alafs*5	29.80
PPM1D	mutated	c.1433dup	p.Cys478Trpfs*3	9.80
PPM1D	mutated	c.1434C>A	p.Cys478*	2.03
PPM1D	mutated	c.1434C>A	p.Cys478*	2.19
PPM1D	mutated	c.1434C>A	p.Cys478*	2.26
PPM1D	mutated	c.1434C>A	p.Cys478*	3.08
PPM1D	mutated	c.1434C>A	p.Cys478*	3.71
PPM1D	mutated	c.1434C>A	p.Cys478*	5.10
PPM1D	mutated	c.1434C>A	p.Cys478*	6.00
PPM1D	mutated	c.1434C>A	p.Cys478*	7.40
PPM1D	mutated	c.1434C>A	p.Cys478*	16.10
PPM1D	mutated	c.1436del	p.Ala479Valfs*4	7.80
PPM1D	mutated	c.1437del	p.Ala481Profs*2	13.90
PPM1D	mutated	c.1438A>T	p.Lys480*	2.28
PPM1D	mutated	c.1438A>T	p.Lys480*	2.70
PPM1D	mutated	c.1439_1440dup	p.Ala481Lysfs*3	3.55
PPM1D	mutated	c.1440del	p.Ala481Profs*2	2.40
PPM1D	mutated	c.1440dup	p.Ala481Serfs*8	2.16
PPM1D	mutated	c.1440dup	p.Ala481Serfs*8	3.40
PPM1D	mutated	c.1441_1442del	p.Ala481Profs*7	2.72
PPM1D	mutated	c.1443_1444delinsT	p.Leu482*	6.40
PPM1D	mutated	c.1444_1445del	p.Leu482Aspfs*6	4.17
PPM1D	mutated	c.1444del	p.Leu482*	2.66
PPM1D	mutated	c.1444del	p.Leu482*	4.01
PPM1D	mutated	c.1444dup	p.Leu482Profs*7	4.36
PPM1D	mutated	c.1444dup	p.Leu482Profs*7	4.68
PPM1D	mutated	c.1450_1451del	p.Leu484Lysfs*4	4.74
PPM1D	mutated	c.1450_1451del	p.Leu484Lysfs*4	22.50
PPM1D	mutated	c.1451_1453delinsG	p.Leu484Trpfs*4	14.00
PPM1D	mutated	c.1451dup	p.Leu484Phefs*5	4.04
PPM1D	mutated	c.1451T>G	p.Leu484*	2.08

PPM1D	mutated	c.1451T>G	p.Leu484*	2.66
PPM1D	mutated	c.1451T>G	p.Leu484*	12.50
PPM1D	mutated	c.1458dup	p.His487Thrfs*2	3.56
PPM1D	mutated	c.1464_1471del	p.Asp488Glufs*2	2.62
PPM1D	mutated	c.1469_1470insA	p.Asn491Glufs*2	3.59
PPM1D	mutated	c.1469del	p.Leu490*	3.09
PPM1D	mutated	c.1469del	p.Leu490*	7.10
PPM1D	mutated	c.1508C>A	p.Ser503*	2.40
PPM1D	mutated	c.1508C>G	p.Ser503*	2.14
PPM1D	mutated	c.1518del	p.Val507Serfs*7	24.10
PPM1D	mutated	c.1523dup	p.Met508Ilefs*20	15.40
PPM1D	mutated	c.1528C>T	p.Gln510*	2.66
PPM1D	mutated	c.1528C>T	p.Gln510*	2.92
PPM1D	mutated	c.1528C>T	p.Gln510*	9.90
PPM1D	mutated	c.1528del	p.Gln510Lysfs*4	4.15
PPM1D	mutated	c.1528del	p.Gln510Lysfs*4	7.10
PPM1D	mutated	c.1535dup	p.Asn512Lysfs*16	3.28
PPM1D	mutated	c.1535dup	p.Asn512Lysfs*16	3.56
PPM1D	mutated	c.1535dup	p.Asn512Lysfs*16	6.80
PPM1D	mutated	c.1535dup	p.Asn512Lysfs*16	10.10
PPM1D	mutated	c.1537_1544del	p.Leu513Valfs*12	2.57
PPM1D	mutated	c.1538del	p.Leu513*	2.63
PPM1D	mutated	c.1538del	p.Leu513*	6.70
PPM1D	mutated	c.1538T>A	p.Leu513*	2.20
PPM1D	mutated	c.1538T>A	p.Leu513*	4.83
PPM1D	mutated	c.1538T>A	p.Leu513*	6.20
PPM1D	mutated	c.1540A>T	p.Lys514*	5.90
PPM1D	mutated	c.1543del	p.Met515Cysfs*7	8.80
PPM1D	mutated	c.1547_1562del	p.Ser516*	4.44
PPM1D	mutated	c.1547C>A	p.Ser516*	11.00
PPM1D	mutated	c.1547C>G	p.Ser516*	24.00
PPM1D	mutated	c.1561del	p.Met521*	4.42
PPM1D	mutated	c.1561del	p.Met521*	7.70
PPM1D	mutated	c.1567del	p.Ala523Profs*16	2.17
PPM1D	mutated	c.1570C>T	p.Gln524*	3.23
PPM1D	mutated	c.1570C>T	p.Gln524*	6.90
PPM1D	mutated	c.1573G>T	p.Glu525*	4.93
PPM1D	mutated	c.1573G>T	p.Glu525*	7.60
PPM1D	mutated	c.1578_1581del	p.Arg528Profs*10	2.69
PPM1D	mutated	c.1579G>T	p.Glu527*	2.05
PPM1D	mutated	c.1587_1599dup	p.Phe534Profs*6	4.53
PPM1D	mutated	c.1589del	p.Pro530Leufs*9	3.24
PPM1D	mutated	c.1589del	p.Pro530Leufs*9	7.60
PPM1D	mutated	c.1589dup	p.Pro531Serfs*5	5.60
PPM1D	mutated	c.1592del	p.Pro531Glnfs*8	5.20
PPM1D	mutated	c.1592del	p.Pro531Glnfs*8	6.50
PPM1D	mutated	c.1592dup	p.Thr532Asnfs*4	19.40

PPM1D	mutated	c.1594del	p.Thr532Glnfs*7	5.00
PPM1D	mutated	c.1594del	p.Thr532Glnfs*7	5.50
PPM1D	mutated	c.1595del	p.Thr532Lysfs*7	5.00
PPM1D	mutated	c.1598dup	p.Asn533Lysfs*3	7.30
PPM1D	mutated	c.1601_1603del	p.Phe534_Lys535delins*	2.82
PPM1D	mutated	c.1602_1603del	p.Phe534Leufs*17	9.00
PPM1D	mutated	c.1602del	p.Phe534Leufs*5	7.40
PPM1D	mutated	c.1602del	p.Phe534Leufs*5	10.00
PPM1D	mutated	c.1606del	p.Arg536Glyfs*3	4.94
PPM1D	mutated	c.1613T>G	p.Leu538*	2.01
PPM1D	mutated	c.1613T>G	p.Leu538*	7.60
PPM1D	mutated	c.1615G>T	p.Glu539*	3.24
PPM1D	mutated	c.1619del	p.Glu540Glyfs*7	2.55
PPM1D	mutated	c.1619del	p.Glu540Glyfs*7	3.76
PPM1D	mutated	c.1621_1630del	p.Ser541Alafs*3	4.87
PPM1D	mutated	c.1625_1626del	p.Asn542Ilefs*9	2.61
PPM1D	mutated	c.1628del	p.Ser543Leufs*4	2.11
PPM1D	mutated	c.1631del	p.Gly544Alafs*3	4.24
PPM1D	mutated	c.1636del	p.Leu546*	5.80
PPM1D	mutated	c.1636del	p.Leu546*	23.60
PPM1D	mutated	c.1636dup	p.Leu546Profs*6	2.22
PPM1D	mutated	c.1636dup	p.Leu546Profs*6	3.17
PPM1D	mutated	c.1636dup	p.Leu546Profs*6	3.79
PPM1D	mutated	c.1636dup	p.Leu546Profs*6	3.87
PPM1D	mutated	c.1636dup	p.Leu546Profs*6	6.90
PPM1D	mutated	c.1636dup	p.Leu546Profs*6	8.00
PPM1D	mutated	c.1637del	p.Leu546Argfs*2	2.08
PPM1D	mutated	c.1637del	p.Leu546Argfs*2	2.20
PPM1D	mutated	c.1637del	p.Leu546Argfs*2	2.22
PPM1D	mutated	c.1645A>T	p.Lys549*	3.31
PPM1D	mutated	c.1649_1662del	p.His550Leufs*5	23.00
PPM1D	mutated	c.1649del	p.His550Leufs*6	5.80
PPM1D	mutated	c.1654C>T	p.Arg552*	2.04
PPM1D	mutated	c.1654C>T	p.Arg552*	2.16
PPM1D	mutated	c.1654C>T	p.Arg552*	2.24
PPM1D	mutated	c.1654C>T	p.Arg552*	2.33
PPM1D	mutated	c.1654C>T	p.Arg552*	2.83
PPM1D	mutated	c.1654C>T	p.Arg552*	3.24
PPM1D	mutated	c.1654C>T	p.Arg552*	3.54
PPM1D	mutated	c.1654C>T	p.Arg552*	3.64
PPM1D	mutated	c.1654C>T	p.Arg552*	3.71
PPM1D	mutated	c.1654C>T	p.Arg552*	3.77
PPM1D	mutated	c.1654C>T	p.Arg552*	4.59
PPM1D	mutated	c.1654C>T	p.Arg552*	5.40
PPM1D	mutated	c.1654C>T	p.Arg552*	5.50
PPM1D	mutated	c.1654C>T	p.Arg552*	5.80
PPM1D	mutated	c.1654C>T	p.Arg552*	6.00

PPM1D	mutated	c.1654C>T	p.Arg552*	6.90
PPM1D	mutated	c.1654C>T	p.Arg552*	9.10
PPM1D	mutated	c.1654C>T	p.Arg552*	9.80
PPM1D	mutated	c.1654C>T	p.Arg552*	10.90
PPM1D	mutated	c.1654C>T	p.Arg552*	22.90
PPM1D	mutated	c.1654C>T	p.Arg552*	24.70
PPM1D	mutated	c.1654C>T	p.Arg552*	42.40
PPM1D	mutated	c.1658_1670delinsTTAAGCCA	p.Asn553Ilefs*5	2.75
PPM1D	mutated	c.1711C>T	p.Gln571*	2.52
PPM1D	mutated	c.1711C>T	p.Gln571*	4.90
PPM1D	mutated	c.1714C>T	p.Arg572*	2.29
PPM1D	mutated	c.1714C>T	p.Arg572*	2.83
PPM1D	mutated	c.1714C>T	p.Arg572*	3.57
PPM1D	mutated	c.1714C>T	p.Arg572*	5.10
PPM1D	mutated	c.1714C>T	p.Arg572*	5.20
PPM1D	mutated	c.1714C>T	p.Arg572*	5.30
PPM1D	mutated	c.1714C>T	p.Arg572*	5.40
PPM1D	mutated	c.1714C>T	p.Arg572*	6.70
PPM1D	mutated	c.1714C>T	p.Arg572*	8.80
PPM1D	mutated	c.1714C>T	p.Arg572*	9.00
PPM1D	mutated	c.1714C>T	p.Arg572*	23.70
PPM1D	mutated	c.1714C>T	p.Arg572*	32.60
PPM1D	mutated	c.1741C>T	p.Arg581*	2.39
PPM1D	mutated	c.1741C>T	p.Arg581*	2.73
PPM1D	mutated	c.1741C>T	p.Arg581*	3.82
PPM1D	mutated	c.1741C>T	p.Arg581*	3.97
PPM1D	mutated	c.1741C>T	p.Arg581*	6.10
PPM1D	mutated	c.1741C>T	p.Arg581*	8.40
PPM1D	mutated	c.1741C>T	p.Arg581*	11.60
SF3B1	mutated	c.1774G>A	p.Glu592Lys	4.87
SF3B1	mutated	c.1856A>T	p.Asn619Ile	3.93
SF3B1	mutated	c.1861G>A	p.Asp621Asn	16.90
SF3B1	mutated	c.1866G>C	p.Glu622Asp	2.64
SF3B1	mutated	c.1866G>C	p.Glu622Asp	3.14
SF3B1	mutated	c.1866G>C	p.Glu622Asp	3.84
SF3B1	mutated	c.1866G>C	p.Glu622Asp	4.47
SF3B1	mutated	c.1866G>T	p.Glu622Asp	18.50
SF3B1	mutated	c.1866G>T	p.Glu622Asp	21.00
SF3B1	mutated	c.1873C>T	p.Arg625Cys	3.66
SF3B1	mutated	c.1876A>G	p.Asn626Asp	7.40
SF3B1	mutated	c.1986C>A	p.His662Gln	2.27
SF3B1	mutated	c.1986C>A	p.His662Gln	9.70
SF3B1	mutated	c.1986C>A	p.His662Gln	26.70
SF3B1	mutated	c.1986C>G	p.His662Gln	3.09
SF3B1	mutated	c.1986C>G	p.His662Gln	3.17
SF3B1	mutated	c.1986C>G	p.His662Gln	10.30
SF3B1	mutated	c.1986C>G	p.His662Gln	32.70

SF3B1	mutated	c.1987A>G	p.Thr663Ala	15.00
SF3B1	mutated	c.1988C>T	p.Thr663Ile	2.13
SF3B1	mutated	c.1988C>T	p.Thr663Ile	3.35
SF3B1	mutated	c.1988C>T	p.Thr663Ile	7.20
SF3B1	mutated	c.1988C>T	p.Thr663Ile	24.50
SF3B1	mutated	c.1996A>C	p.Lys666Gln	4.62
SF3B1	mutated	c.1996A>C	p.Lys666Gln	5.20
SF3B1	mutated	c.1996A>C	p.Lys666Gln	28.20
SF3B1	mutated	c.1996A>G	p.Lys666Glu	2.71
SF3B1	mutated	c.1996A>G	p.Lys666Glu	6.50
SF3B1	mutated	c.1997A>C	p.Lys666Thr	3.00
SF3B1	mutated	c.1997A>C	p.Lys666Thr	3.99
SF3B1	mutated	c.1997A>C	p.Lys666Thr	5.00
SF3B1	mutated	c.1997A>C	p.Lys666Thr	5.20
SF3B1	mutated	c.1997A>C	p.Lys666Thr	5.50
SF3B1	mutated	c.1997A>C	p.Lys666Thr	6.20
SF3B1	mutated	c.1997A>C	p.Lys666Thr	7.30
SF3B1	mutated	c.1997A>C	p.Lys666Thr	16.20
SF3B1	mutated	c.1997A>C	p.Lys666Thr	26.60
SF3B1	mutated	c.1997A>G	p.Lys666Arg	2.02
SF3B1	mutated	c.1997A>G	p.Lys666Arg	2.16
SF3B1	mutated	c.1997A>G	p.Lys666Arg	3.03
SF3B1	mutated	c.1997A>G	p.Lys666Arg	3.57
SF3B1	mutated	c.1997A>G	p.Lys666Arg	3.70
SF3B1	mutated	c.1997A>G	p.Lys666Arg	4.12
SF3B1	mutated	c.1997A>G	p.Lys666Arg	4.43
SF3B1	mutated	c.1997A>G	p.Lys666Arg	5.10
SF3B1	mutated	c.1997A>G	p.Lys666Arg	5.30
SF3B1	mutated	c.1997A>G	p.Lys666Arg	5.80
SF3B1	mutated	c.1997A>G	p.Lys666Arg	10.00
SF3B1	mutated	c.1997A>G	p.Lys666Arg	11.20
SF3B1	mutated	c.1997A>G	p.Lys666Arg	23.70
SF3B1	mutated	c.1997A>G	p.Lys666Arg	24.80
SF3B1	mutated	c.1997A>G	p.Lys666Arg	28.10
SF3B1	mutated	c.1997A>G	p.Lys666Arg	31.20
SF3B1	mutated	c.1997A>T	p.Lys666Met	2.43
SF3B1	mutated	c.1997A>T	p.Lys666Met	16.50
SF3B1	mutated	c.1997A>T	p.Lys666Met	18.20
SF3B1	mutated	c.1998G>C	p.Lys666Asn	2.01
SF3B1	mutated	c.1998G>C	p.Lys666Asn	2.25
SF3B1	mutated	c.1998G>C	p.Lys666Asn	3.90
SF3B1	mutated	c.1998G>C	p.Lys666Asn	4.44
SF3B1	mutated	c.1998G>C	p.Lys666Asn	10.60
SF3B1	mutated	c.1998G>C	p.Lys666Asn	22.70
SF3B1	mutated	c.1998G>T	p.Lys666Asn	2.07
SF3B1	mutated	c.1998G>T	p.Lys666Asn	2.10
SF3B1	mutated	c.1998G>T	p.Lys666Asn	2.13

SF3B1	mutated	c.1998G>T	p.Lys666Asn	2.27
SF3B1	mutated	c.1998G>T	p.Lys666Asn	2.76
SF3B1	mutated	c.1998G>T	p.Lys666Asn	3.05
SF3B1	mutated	c.1998G>T	p.Lys666Asn	3.31
SF3B1	mutated	c.1998G>T	p.Lys666Asn	3.52
SF3B1	mutated	c.1998G>T	p.Lys666Asn	3.76
SF3B1	mutated	c.1998G>T	p.Lys666Asn	3.90
SF3B1	mutated	c.1998G>T	p.Lys666Asn	18.30
SF3B1	mutated	c.1998G>T	p.Lys666Asn	29.50
SF3B1	mutated	c.1998G>T	p.Lys666Asn	38.50
SF3B1	mutated	c.2017A>G	p.Ile673Val	2.94
SF3B1	mutated	c.2017A>G	p.Ile673Val	5.90
SF3B1	mutated	c.2018T>G	p.Ile673Ser	25.50
SF3B1	mutated	c.2098A>G	p.Lys700Glu	2.03
SF3B1	mutated	c.2098A>G	p.Lys700Glu	2.27
SF3B1	mutated	c.2098A>G	p.Lys700Glu	2.29
SF3B1	mutated	c.2098A>G	p.Lys700Glu	2.38
SF3B1	mutated	c.2098A>G	p.Lys700Glu	2.49
SF3B1	mutated	c.2098A>G	p.Lys700Glu	2.67
SF3B1	mutated	c.2098A>G	p.Lys700Glu	2.81
SF3B1	mutated	c.2098A>G	p.Lys700Glu	2.82
SF3B1	mutated	c.2098A>G	p.Lys700Glu	3.04
SF3B1	mutated	c.2098A>G	p.Lys700Glu	3.25
SF3B1	mutated	c.2098A>G	p.Lys700Glu	3.51
SF3B1	mutated	c.2098A>G	p.Lys700Glu	3.61
SF3B1	mutated	c.2098A>G	p.Lys700Glu	3.63
SF3B1	mutated	c.2098A>G	p.Lys700Glu	3.66
SF3B1	mutated	c.2098A>G	p.Lys700Glu	3.82
SF3B1	mutated	c.2098A>G	p.Lys700Glu	4.00
SF3B1	mutated	c.2098A>G	p.Lys700Glu	4.12
SF3B1	mutated	c.2098A>G	p.Lys700Glu	4.23
SF3B1	mutated	c.2098A>G	p.Lys700Glu	4.59
SF3B1	mutated	c.2098A>G	p.Lys700Glu	4.84
SF3B1	mutated	c.2098A>G	p.Lys700Glu	5.00
SF3B1	mutated	c.2098A>G	p.Lys700Glu	5.10
SF3B1	mutated	c.2098A>G	p.Lys700Glu	6.20
SF3B1	mutated	c.2098A>G	p.Lys700Glu	6.70
SF3B1	mutated	c.2098A>G	p.Lys700Glu	7.30
SF3B1	mutated	c.2098A>G	p.Lys700Glu	7.40
SF3B1	mutated	c.2098A>G	p.Lys700Glu	10.60
SF3B1	mutated	c.2098A>G	p.Lys700Glu	12.70
SF3B1	mutated	c.2098A>G	p.Lys700Glu	14.40
SF3B1	mutated	c.2098A>G	p.Lys700Glu	16.00
SF3B1	mutated	c.2098A>G	p.Lys700Glu	18.00
SF3B1	mutated	c.2098A>G	p.Lys700Glu	18.10
SF3B1	mutated	c.2098A>G	p.Lys700Glu	19.30
SF3B1	mutated	c.2098A>G	p.Lys700Glu	21.30

SF3B1	mutated	c.2098A>G	p.Lys700Glu	34.90
SF3B1	mutated	c.2098A>G	p.Lys700Glu	42.90
SF3B1	mutated	c.2111T>A	p.Ile704Asn	6.00
SF3B1	mutated	c.2149A>G	p.Thr717Ala	4.26
SF3B1	mutated	c.2218G>C	p.Gly740Arg	3.80
SF3B1	mutated	c.2218G>C	p.Gly740Arg	16.90
SF3B1	mutated	c.2219G>A	p.Gly740Glu	3.92
SF3B1	mutated	c.2219G>A	p.Gly740Glu	7.90
SF3B1	mutated	c.2225G>A	p.Gly742Asp	2.00
SF3B1	mutated	c.2225G>A	p.Gly742Asp	2.10
SF3B1	mutated	c.2225G>A	p.Gly742Asp	6.80
SF3B1	mutated	c.2225G>A	p.Gly742Asp	8.30
SF3B1	mutated	c.2225G>A	p.Gly742Asp	10.80
SF3B1	mutated	c.2225G>A	p.Gly742Asp	19.70
SF3B1	mutated	c.2225G>A	p.Gly742Asp	25.40
SF3B1	mutated	c.2230G>C	p.Ala744Pro	6.10
SF3B1	mutated	c.2231C>A	p.Ala744Asp	12.00
SF3B1	mutated	c.2242A>G	p.Lys748Glu	12.60
SF3B1	mutated	c.2244G>T	p.Lys748Asn	17.90
SF3B1	mutated	c.2366T>A	p.Leu789Gln	11.50
SF3B1	mutated	c.2366T>C	p.Leu789Pro	5.20
SRSF2	mutated	c.160T>G	p.Ser54Ala	2.04
SRSF2	mutated	c.161C>A	p.Ser54Tyr	3.37
SRSF2	mutated	c.161C>T	p.Ser54Phe	11.90
SRSF2	mutated	c.170T>A	p.Phe57Tyr	10.50
SRSF2	mutated	c.170T>A	p.Phe57Tyr	12.30
SRSF2	mutated	c.170T>A	p.Phe57Tyr	34.20
SRSF2	mutated	c.279_347del	p.Pro95_Arg117del	5.80
SRSF2	mutated	c.280_281insCTACCCGTGA	p.Arg94Profs*33	11.00
SRSF2	mutated	c.281_283dup	p.Arg94dup	4.90
SRSF2	mutated	c.282_284delinsACG	p.Pro95Arg	19.00
SRSF2	mutated	c.282_284delinsTAT	p.Pro95Ile	5.20
SRSF2	mutated	c.282_284delinsTCT	p.Pro95Leu	2.88
SRSF2	mutated	c.283C>A	p.Pro95Thr	7.50
SRSF2	mutated	c.283C>A	p.Pro95Thr	8.70
SRSF2	mutated	c.283C>A	p.Pro95Thr	8.90
SRSF2	mutated	c.283C>G	p.Pro95Ala	3.13
SRSF2	mutated	c.284_307del	p.Pro95_Arg102del	2.71
SRSF2	mutated	c.284_307del	p.Pro95_Arg102del	6.70
SRSF2	mutated	c.284_307del	p.Pro95_Arg102del	7.80
SRSF2	mutated	c.284_307del	p.Pro95_Arg102del	17.60
SRSF2	mutated	c.284_307del	p.Pro95_Arg102del	23.00
SRSF2	mutated	c.284C>A	p.Pro95His	2.99
SRSF2	mutated	c.284C>A	p.Pro95His	3.71
SRSF2	mutated	c.284C>A	p.Pro95His	3.96
SRSF2	mutated	c.284C>A	p.Pro95His	4.57
SRSF2	mutated	c.284C>A	p.Pro95His	4.69

SRSF2	mutated	c.284C>A	p.Pro95His	5.80
SRSF2	mutated	c.284C>A	p.Pro95His	6.20
SRSF2	mutated	c.284C>A	p.Pro95His	7.30
SRSF2	mutated	c.284C>A	p.Pro95His	7.40
SRSF2	mutated	c.284C>A	p.Pro95His	8.90
SRSF2	mutated	c.284C>A	p.Pro95His	11.70
SRSF2	mutated	c.284C>A	p.Pro95His	12.30
SRSF2	mutated	c.284C>A	p.Pro95His	12.40
SRSF2	mutated	c.284C>A	p.Pro95His	13.20
SRSF2	mutated	c.284C>A	p.Pro95His	13.90
SRSF2	mutated	c.284C>A	p.Pro95His	15.30
SRSF2	mutated	c.284C>A	p.Pro95His	15.30
SRSF2	mutated	c.284C>A	p.Pro95His	22.50
SRSF2	mutated	c.284C>A	p.Pro95His	22.90
SRSF2	mutated	c.284C>A	p.Pro95His	23.00
SRSF2	mutated	c.284C>A	p.Pro95His	23.60
SRSF2	mutated	c.284C>A	p.Pro95His	25.40
SRSF2	mutated	c.284C>A	p.Pro95His	26.40
SRSF2	mutated	c.284C>A	p.Pro95His	26.60
SRSF2	mutated	c.284C>A	p.Pro95His	30.30
SRSF2	mutated	c.284C>A	p.Pro95His	30.80
SRSF2	mutated	c.284C>A	p.Pro95His	31.80
SRSF2	mutated	c.284C>A	p.Pro95His	35.60
SRSF2	mutated	c.284C>A	p.Pro95His	36.90
SRSF2	mutated	c.284C>A	p.Pro95His	37.00
SRSF2	mutated	c.284C>A	p.Pro95His	37.00
SRSF2	mutated	c.284C>A	p.Pro95His	39.90
SRSF2	mutated	c.284C>A	p.Pro95His	41.20
SRSF2	mutated	c.284C>G	p.Pro95Arg	3.17
SRSF2	mutated	c.284C>G	p.Pro95Arg	3.29
SRSF2	mutated	c.284C>G	p.Pro95Arg	3.74
SRSF2	mutated	c.284C>G	p.Pro95Arg	3.95
SRSF2	mutated	c.284C>G	p.Pro95Arg	5.00
SRSF2	mutated	c.284C>G	p.Pro95Arg	5.60
SRSF2	mutated	c.284C>G	p.Pro95Arg	6.60
SRSF2	mutated	c.284C>G	p.Pro95Arg	7.60
SRSF2	mutated	c.284C>G	p.Pro95Arg	8.20
SRSF2	mutated	c.284C>G	p.Pro95Arg	8.90
SRSF2	mutated	c.284C>G	p.Pro95Arg	10.30
SRSF2	mutated	c.284C>G	p.Pro95Arg	17.50
SRSF2	mutated	c.284C>G	p.Pro95Arg	18.60
SRSF2	mutated	c.284C>G	p.Pro95Arg	30.10
SRSF2	mutated	c.284C>G	p.Pro95Arg	30.70
SRSF2	mutated	c.284C>G	p.Pro95Arg	31.20
SRSF2	mutated	c.284C>G	p.Pro95Arg	39.10
SRSF2	mutated	c.284C>T	p.Pro95Leu	2.19
SRSF2	mutated	c.284C>T	p.Pro95Leu	2.35

SRSF2	mutated	c.284C>T	p.Pro95Leu	2.43
SRSF2	mutated	c.284C>T	p.Pro95Leu	3.11
SRSF2	mutated	c.284C>T	p.Pro95Leu	4.81
SRSF2	mutated	c.284C>T	p.Pro95Leu	4.86
SRSF2	mutated	c.284C>T	p.Pro95Leu	6.50
SRSF2	mutated	c.284C>T	p.Pro95Leu	6.60
SRSF2	mutated	c.284C>T	p.Pro95Leu	11.20
SRSF2	mutated	c.284C>T	p.Pro95Leu	12.50
SRSF2	mutated	c.284C>T	p.Pro95Leu	15.40
SRSF2	mutated	c.284C>T	p.Pro95Leu	19.80
SRSF2	mutated	c.284C>T	p.Pro95Leu	20.20
SRSF2	mutated	c.284C>T	p.Pro95Leu	21.20
SRSF2	mutated	c.284C>T	p.Pro95Leu	23.40
SRSF2	mutated	c.284C>T	p.Pro95Leu	23.60
SRSF2	mutated	c.284C>T	p.Pro95Leu	29.80
SRSF2	mutated	c.284C>T	p.Pro95Leu	34.20
SRSF2	mutated	c.284C>T	p.Pro95Leu	35.20
SRSF2	mutated	c.284C>T	p.Pro95Leu	36.40
SRSF2	mutated	c.284C>T	p.Pro95Leu	37.20
SRSF2	mutated	c.284C>T	p.Pro95Leu	39.70
SRSF2	mutated	c.293C>G	p.Ser98*	2.87
SRSF2	mutated	c.295_301del	p.His99Alafs*143	11.00
SRSF2	mutated	c.61C>G	p.Leu21Val	2.03
TET2	mutated	4044+2_4044+20del		4.00
TET2	mutated	c.1013del	p.Asn338Ilefs*9	17.50
TET2	mutated	c.1038_1039dup	p.Ala347Glufs*26	6.30
TET2	mutated	c.1048del	p.Glu350Lysfs*22	12.50
TET2	mutated	c.1081C>T	p.Gln361*	5.70
TET2	mutated	c.1118_1122del	p.Gln373Argfs*15	2.44
TET2	mutated	c.1120_1121del	p.Asn374*	2.85
TET2	mutated	c.1121A>C	p.Asn374Thr	2.33
TET2	mutated	c.1166dup	p.Asp390Glyfs*53	31.10
TET2	mutated	c.1188del	p.Thr397Hisfs*30	2.17
TET2	mutated	c.1198C>T	p.Pro400Ser	3.28
TET2	mutated	c.1200del	p.Pro401Hisfs*26	2.67
TET2	mutated	c.1201_1203del	p.Pro401del	2.63
TET2	mutated	c.1207C>T	p.Gln403*	2.02
TET2	mutated	c.1207C>T	p.Gln403*	34.40
TET2	mutated	c.1213dup	p.Leu405Profs*38	2.20
TET2	mutated	c.1218_1221del	p.Ser407Profs*19	15.00
TET2	mutated	c.1219del	p.Ser407Leufs*20	2.94
TET2	mutated	c.1220del	p.Ser407Phefs*20	4.80
TET2	mutated	c.1238del	p.Pro413Hisfs*14	3.70
TET2	mutated	c.1249C>T	p.Gln417*	3.73
TET2	mutated	c.1256del	p.Pro419Leufs*8	2.91
TET2	mutated	c.1275_1278dup	p.Asn427Serfs*17	3.51
TET2	mutated	c.1294G>T	p.Glu432*	2.82

TET2	mutated	c.1297del	p.Glu433Asnfs*14	15.60
TET2	mutated	c.12dup	p.Arg5*	6.10
TET2	mutated	c.1304del	p.His435Profs*12	2.36
TET2	mutated	c.1311C>A	p.Tyr437*	2.11
TET2	mutated	c.1327del	p.Thr443Glnfs*4	5.20
TET2	mutated	c.1329_1333del	p.Thr444Phefs*9	2.89
TET2	mutated	c.1352del	p.Ile451Lysfs*35	20.70
TET2	mutated	c.1364C>G	p.Pro455Arg	43.90
TET2	mutated	c.1366dup	p.Glu456Glyfs*9	9.90
TET2	mutated	c.1381C>T	p.Gln461*	3.95
TET2	mutated	c.1382del	p.Gln461Argfs*25	32.70
TET2	mutated	c.1384del	p.Ser462Valfs*24	34.20
TET2	mutated	c.1386dup	p.Pro463Serfs*2	12.00
TET2	mutated	c.1388del	p.Pro463Leufs*23	5.20
TET2	mutated	c.1411_1421delinsGGA	p.Ser471Glyfs*5	3.05
TET2	mutated	c.1425del	p.Met475Ilefs*11	5.00
TET2	mutated	c.1429del	p.Ser477Leufs*9	2.15
TET2	mutated	c.1429del	p.Ser477Leufs*9	3.23
TET2	mutated	c.1429del	p.Ser477Leufs*9	10.50
TET2	mutated	c.1432del	p.Glu478Lysfs*8	3.48
TET2	mutated	c.1435dup	p.Arg479Lysfs*5	8.20
TET2	mutated	c.1439del	p.Pro480Leufs*6	2.46
TET2	mutated	c.1448_1457del	p.Asn483Thrfs*11	11.00
TET2	mutated	c.1450dup	p.Cys484Leufs*6	2.42
TET2	mutated	c.1452T>A	p.Cys484*	5.50
TET2	mutated	c.1489A>T	p.Thr497Ser	47.60
TET2	mutated	c.1492_1493del	p.Val498Serfs*5	5.00
TET2	mutated	c.1492dup	p.Val498Glyfs*6	6.70
TET2	mutated	c.1496del	p.Pro499Hisfs*34	5.80
TET2	mutated	c.1510_1513del	p.Lys504Glnfs*28	12.00
TET2	mutated	c.1526C>G	p.Ser509*	8.50
TET2	mutated	c.1534del	p.Leu512Serfs*21	7.30
TET2	mutated	c.1540del	p.His514Ilefs*19	4.08
TET2	mutated	c.1576C>T	p.Gln526*	5.90
TET2	mutated	c.1581del	p.Asp527Glufs*6	2.02
TET2	mutated	c.1583del	p.Asn528Thrfs*5	4.90
TET2	mutated	c.1587_1599del	p.Cys529*	2.49
TET2	mutated	c.1588C>T	p.Gln530*	3.08
TET2	mutated	c.1605_1606insG	p.Lys536Glufs*31	13.60
TET2	mutated	c.1610del	p.Glu537Glyfs*5	12.20
TET2	mutated	c.1614del	p.Glu539Argfs*3	12.00
TET2	mutated	c.1614delA	p.Glu539Argfs*3	6.40
TET2	mutated	c.1620del	p.Leu541*	3.25
TET2	mutated	c.1622T>C	p.Leu541Pro	7.10
TET2	mutated	c.1630C>T	p.Arg544*	2.77
TET2	mutated	c.1630C>T	p.Arg544*	3.01
TET2	mutated	c.1630C>T	p.Arg544*	3.47

TET2	mutated	c.1630C>T	p.Arg544*	3.55
TET2	mutated	c.1630C>T	p.Arg544*	4.22
TET2	mutated	c.1630C>T	p.Arg544*	7.70
TET2	mutated	c.1630C>T	p.Arg544*	11.60
TET2	mutated	c.1630C>T	p.Arg544*	14.60
TET2	mutated	c.1648C>T	p.Arg550*	2.01
TET2	mutated	c.1648C>T	p.Arg550*	2.12
TET2	mutated	c.1648C>T	p.Arg550*	2.18
TET2	mutated	c.1648C>T	p.Arg550*	3.66
TET2	mutated	c.1648C>T	p.Arg550*	3.70
TET2	mutated	c.1648C>T	p.Arg550*	4.36
TET2	mutated	c.1648C>T	p.Arg550*	4.76
TET2	mutated	c.1648C>T	p.Arg550*	5.20
TET2	mutated	c.1648C>T	p.Arg550*	8.20
TET2	mutated	c.1648C>T	p.Arg550*	19.50
TET2	mutated	c.1648C>T	p.Arg550*	21.00
TET2	mutated	c.1648C>T	p.Arg550*	33.50
TET2	mutated	c.1664del	p.Pro555Glnfs*6	2.66
TET2	mutated	c.1669C>T	p.Gln557*	5.90
TET2	mutated	c.1674_1765del	p.Tyr559Serfs*48	3.84
TET2	mutated	c.1677T>A	p.Tyr559*	5.80
TET2	mutated	c.1691G>A	p.Trp564*	3.97
TET2	mutated	c.1691G>A	p.Trp564*	37.60
TET2	mutated	c.1692del	p.Trp564*	11.70
TET2	mutated	c.1692G>A	p.Trp564*	26.60
TET2	mutated	c.1699_1703del	p.Leu567Glyfs*14	7.50
TET2	mutated	c.1712G>A	p.Arg571His	41.60
TET2	mutated	c.1717del	p.His573Thrfs*7	15.90
TET2	mutated	c.1720C>T	p.Gln574*	37.40
TET2	mutated	c.1747G>T	p.Glu583*	2.46
TET2	mutated	c.1760_1761del	p.Pro587Leufs*50	16.10
TET2	mutated	c.1763C>G	p.Ser588*	3.20
TET2	mutated	c.1776T>A	p.Tyr592*	2.62
TET2	mutated	c.1782del	p.Asn595Ilefs*6	2.70
TET2	mutated	c.1795C>T	p.Gln599*	6.10
TET2	mutated	c.1810C>T	p.Gln604*	2.75
TET2	mutated	c.1827del	p.Asn610Thrfs*29	11.70
TET2	mutated	c.1842del	p.Leu615Serfs*24	4.13
TET2	mutated	c.1842del	p.Leu615Serfs*24	4.54
TET2	mutated	c.1842del	p.Leu615Serfs*24	6.90
TET2	mutated	c.1842dup	p.Leu615Alafs*23	3.37
TET2	mutated	c.1842dup	p.Leu615Alafs*23	4.52
TET2	mutated	c.1842dup	p.Leu615Alafs*23	5.10
TET2	mutated	c.1842dup	p.Leu615Alafs*23	6.60
TET2	mutated	c.1842dup	p.Leu615Alafs*23	7.10
TET2	mutated	c.1842dup	p.Leu615Alafs*23	14.40
TET2	mutated	c.1864del	p.Gln622Argfs*17	2.43

TET2	mutated	c.1876C>T	p.Gln626*	2.27
TET2	mutated	c.1894C>T	p.Gln632*	8.00
TET2	mutated	c.1924C>T	p.Gln642*	2.88
TET2	mutated	c.1930C>T	p.Gln644*	3.18
TET2	mutated	c.1945C>T	p.Gln649*	3.72
TET2	mutated	c.1945C>T	p.Gln649*	10.50
TET2	mutated	c.1954C>T	p.Gln652*	18.80
TET2	mutated	c.1955del	p.Gln652Argfs*48	2.45
TET2	mutated	c.1968del	p.Ser657Hisfs*43	13.50
TET2	mutated	c.1972del	p.His658Thrfs*42	2.87
TET2	mutated	c.20_21del	p.Asn7Thrfs*3	2.19
TET2	mutated	c.2028del	p.Cys677Valfs*23	2.72
TET2	mutated	c.2029_2032del	p.Cys677Alafs*22	2.71
TET2	mutated	c.2049del	p.Gln684Asnfs*16	2.48
TET2	mutated	c.2056del	p.Arg686Glufs*14	2.08
TET2	mutated	c.2058_2059del	p.Arg686Serfs*6	4.01
TET2	mutated	c.2068_2083del	p.Gln690Cysfs*5	10.00
TET2	mutated	c.2113C>T	p.Gln705*	2.07
TET2	mutated	c.2116C>T	p.Gln706*	4.54
TET2	mutated	c.2125G>T	p.Glu709*	6.10
TET2	mutated	c.2131G>T	p.Glu711*	9.90
TET2	mutated	c.2147C>G	p.Ser716*	28.80
TET2	mutated	c.2148dup	p.His717Thrfs*6	2.25
TET2	mutated	c.2148dup	p.His717Thrfs*6	12.70
TET2	mutated	c.2176C>T	p.Gln726*	2.28
TET2	mutated	c.2185C>T	p.Gln729*	3.10
TET2	mutated	c.218G>A	p.Arg73His	5.00
TET2	mutated	c.2191C>T	p.Gln731*	2.42
TET2	mutated	c.2200C>T	p.Gln734*	6.50
TET2	mutated	c.2203del	p.Ser735Valfs*16	7.60
TET2	mutated	c.2207C>A	p.Ser736*	2.72
TET2	mutated	c.220del	p.Val74*	7.60
TET2	mutated	c.2215_2216del	p.Pro739Serfs*14	2.17
TET2	mutated	c.2224C>T	p.Gln742*	2.19
TET2	mutated	c.2224C>T	p.Gln742*	7.40
TET2	mutated	c.2227C>T	p.Gln743*	4.36
TET2	mutated	c.2230C>T	p.Gln744*	8.70
TET2	mutated	c.2230C>T	p.Gln744*	25.70
TET2	mutated	c.2230C>T	p.Gln744*	27.10
TET2	mutated	c.2233C>T	p.Gln745*	3.40
TET2	mutated	c.2233C>T	p.Gln745*	17.50
TET2	mutated	c.2236_2237delinsG	p.Gln746Glufs*5	20.00
TET2	mutated	c.2240_2244del	p.Lys747Thrfs*5	3.74
TET2	mutated	c.2249_2252del	p.Ile750Argfs*62	9.90
TET2	mutated	c.2249del	p.Ile750Lysfs*63	14.00
TET2	mutated	c.224del	p.Ser75Ilefs*20	4.89
TET2	mutated	c.2251A>T	p.Lys751*	40.20

TET2	mutated	c.2252_2255del	p.Lys751Ilefs*61	3.10
TET2	mutated	c.2268dup	p.Leu757Thrfs*12	4.24
TET2	mutated	c.2270_2274del	p.Leu757Hisfs*10	7.80
TET2	mutated	c.2272_2276delinsG	p.Gln758Valfs*54	4.08
TET2	mutated	c.2272C>T	p.Gln758*	2.49
TET2	mutated	c.2274del	p.Thr759Leufs*54	28.60
TET2	mutated	c.2280del	p.Pro761Leufs*52	3.34
TET2	mutated	c.2282del	p.Pro761Leufs*52	9.90
TET2	mutated	c.2290C>T	p.Gln764*	3.46
TET2	mutated	c.2290dup	p.Gln764Profs*5	6.10
TET2	mutated	c.2300A>G	p.Asn767Ser	43.60
TET2	mutated	c.2305C>T	p.Gln769*	2.11
TET2	mutated	c.2317G>T	p.Gly773*	11.00
TET2	mutated	c.2321C>A	p.Ser774*	3.24
TET2	mutated	c.2324dup	p.Phe776Leufs*5	14.90
TET2	mutated	c.2339_2349del	p.Lys780Metfs*5	5.00
TET2	mutated	c.2352_2362dup	p.Glu788Valfs*29	2.35
TET2	mutated	c.2362G>T	p.Glu788*	2.43
TET2	mutated	c.2368C>T	p.Gln790*	2.00
TET2	mutated	c.238del	p.Gln80Lysfs*15	6.40
TET2	mutated	c.2400_2401del	p.His800Glnfs*15	10.90
TET2	mutated	c.2414del	p.Gly805Aspfs*8	2.18
TET2	mutated	c.2428C>T	p.Gln810*	5.40
TET2	mutated	c.2428C>T	p.Gln810*	6.40
TET2	mutated	c.2438_2450del	p.Asn813Thrfs*7	4.47
TET2	mutated	c.2449_2455del	p.Ser817Ilefs*5	12.20
TET2	mutated	c.244del	p.Ser82Valfs*13	16.30
TET2	mutated	c.2453del	p.Pro818Leufs*6	3.12
TET2	mutated	c.2456dup	p.Tyr819*	31.00
TET2	mutated	c.2457T>G	p.Tyr819*	2.40
TET2	mutated	c.2474C>A	p.Ser825*	2.45
TET2	mutated	c.2474C>G	p.Ser825*	4.63
TET2	mutated	c.2474C>G	p.Ser825*	7.10
TET2	mutated	c.2474C>G	p.Ser825*	12.20
TET2	mutated	c.2504C>A	p.Ser835*	13.90
TET2	mutated	c.2510dup	p.Asn837Lysfs*9	2.44
TET2	mutated	c.2544_2547dup	p.His850Tyrfs*4	2.07
TET2	mutated	c.2544dup	p.Thr849Tyrfs*4	6.70
TET2	mutated	c.2549_2552del	p.His850Leufs*22	3.01
TET2	mutated	c.2554G>T	p.Glu852*	2.06
TET2	mutated	c.2578C>T	p.Gln860*	2.08
TET2	mutated	c.2596C>T	p.Gln866*	3.81
TET2	mutated	c.2621del	p.Pro874Glnfs*47	2.32
TET2	mutated	c.2626C>T	p.Gln876*	2.31
TET2	mutated	c.262del	p.Cys88Valfs*7	9.20
TET2	mutated	c.2638del	p.His880Thrfs*41	9.10
TET2	mutated	c.2649dup	p.Gln884Serfs*17	2.77

TET2	mutated	c.2662_2665delinsAAGT	p.Gln888_Lys889delinsLys*	4.20
TET2	mutated	c.2662C>T	p.Gln888*	5.30
TET2	mutated	c.266del	p.Leu89Cysfs*6	2.12
TET2	mutated	c.266T>A	p.Leu89*	4.28
TET2	mutated	c.2674C>T	p.Gln892*	2.89
TET2	mutated	c.268C>T	p.Gln90*	2.91
TET2	mutated	c.268C>T	p.Gln90*	5.60
TET2	mutated	c.2718del	p.Met906Ilefs*15	5.50
TET2	mutated	c.2728del	p.Gln910Lysfs*11	2.08
TET2	mutated	c.2737C>T	p.Gln913*	5.20
TET2	mutated	c.2743dup	p.Ala915Glyfs*9	3.30
TET2	mutated	c.2746C>T	p.Gln916*	2.16
TET2	mutated	c.2746C>T	p.Gln916*	4.81
TET2	mutated	c.2746C>T	p.Gln916*	6.10
TET2	mutated	c.2746C>T	p.Gln916*	6.30
TET2	mutated	c.2746C>T	p.Gln916*	10.60
TET2	mutated	c.2746C>T	p.Gln916*	12.30
TET2	mutated	c.2746C>T	p.Gln916*	16.70
TET2	mutated	c.2746C>T	p.Gln916*	30.00
TET2	mutated	c.2757C>G	p.Tyr919*	2.11
TET2	mutated	c.2757C>G	p.Tyr919*	9.20
TET2	mutated	c.2763dup	p.His922Thrfs*2	2.62
TET2	mutated	c.2796_2797insT	p.Gln933Serfs*39	2.73
TET2	mutated	c.2797C>T	p.Gln933*	3.63
TET2	mutated	c.2797C>T	p.Gln933*	4.66
TET2	mutated	c.2797C>T	p.Gln933*	26.00
TET2	mutated	c.2797C>T	p.Gln933*	32.60
TET2	mutated	c.2797C>T	p.Gln933*	47.10
TET2	mutated	c.2812_2866dup	p.Leu956Hisfs*34	19.00
TET2	mutated	c.2814_2815del	p.Gln939Aspfs*32	27.50
TET2	mutated	c.2815C>T	p.Gln939*	2.56
TET2	mutated	c.2846del	p.His949Leufs*4	22.80
TET2	mutated	c.2846dup	p.His949Glnfs*23	11.00
TET2	mutated	c.2848del	p.Ala950Leufs*3	7.60
TET2	mutated	c.285del	p.Lys95Asnfs*18	11.00
TET2	mutated	c.2862G>A	p.Trp954*	3.44
TET2	mutated	c.2871dup	p.Gln958Thrfs*14	3.32
TET2	mutated	c.2871dup	p.Gln958Thrfs*14	5.40
TET2	mutated	c.2878C>G	p.Gln960Glu	6.80
TET2	mutated	c.287del	p.Arg96Profs*17	6.90
TET2	mutated	c.2884C>T	p.Gln962*	3.26
TET2	mutated	c.2899C>T	p.Gln967*	2.55
TET2	mutated	c.2905del	p.Gln969Lysfs*38	2.26
TET2	mutated	c.2935_2954del	p.Arg979Trpfs*23	11.00
TET2	mutated	c.2973del	p.Cys992Valfs*15	6.70
TET2	mutated	c.3021_3022del	p.Lys1008Alafs*9	5.10
TET2	mutated	c.3021dup	p.Lys1008*	2.90

TET2	mutated	c.3025C>T	p.Gln1009*	2.06
TET2	mutated	c.3039del	p.Ala1014Glnfs*19	2.23
TET2	mutated	c.3039del	p.Ala1014Glnfs*19	40.70
TET2	mutated	c.3058C>T	p.Gln1020*	2.65
TET2	mutated	c.3058C>T	p.Gln1020*	9.10
TET2	mutated	c.3058C>T	p.Gln1020*	32.30
TET2	mutated	c.3088C>T	p.Gln1030*	4.89
TET2	mutated	c.3088C>T	p.Gln1030*	5.40
TET2	mutated	c.3100C>T	p.Gln1034*	18.00
TET2	mutated	c.311_312del	p.Ser104Trpfs*15	2.41
TET2	mutated	c.3120dup	p.Phe1041Ilefs*2	6.60
TET2	mutated	c.3120dup	p.Phe1041Ilefs*2	14.80
TET2	mutated	c.3127_3128del	p.His1043*	5.20
TET2	mutated	c.3127dup	p.His1043Profs*2	5.30
TET2	mutated	c.3138del	p.Thr1047Leufs*8	5.60
TET2	mutated	c.3138dup	p.Thr1047Tyrfs*11	3.20
TET2	mutated	c.3141del	p.Leu1048Serfs*7	6.40
TET2	mutated	c.3149C>A	p.Ser1050*	26.60
TET2	mutated	c.3151C>T	p.Gln1051*	7.40
TET2	mutated	c.3151C>T	p.Gln1051*	23.10
TET2	mutated	c.3151C>T	p.Gln1051*	39.90
TET2	mutated	c.3157_3160del	p.Gln1053*	3.23
TET2	mutated	c.3157C>T	p.Gln1053*	2.51
TET2	mutated	c.3177del	p.Pro1061Glnfs*5	3.77
TET2	mutated	c.3186del	p.Thr1063Glnfs*3	6.00
TET2	mutated	c.3188_3194del	p.Thr1063Argfs*17	2.67
TET2	mutated	c.3190dup	p.Val1064Glyfs*4	18.80
TET2	mutated	c.3194del	p.Leu1065*	12.40
TET2	mutated	c.322C>T	p.Gln108*	5.00
TET2	mutated	c.3236del	p.Pro1079Glnfs*3	2.58
TET2	mutated	c.3236del	p.Pro1079Glnfs*3	6.90
TET2	mutated	c.3269del	p.Lys1090Argfs*16	4.51
TET2	mutated	c.3299_3306delinsG	p.Val1100Glyfs*4	5.00
TET2	mutated	c.3300del	p.Leu1101Serfs*5	20.50
TET2	mutated	c.3311_3312del	p.Phe1104Tyrfs*25	9.10
TET2	mutated	c.3311_3312insATAT	p.Phe1104Leufs*27	18.50
TET2	mutated	c.3312_3319del	p.Ile1105Thrfs*22	5.50
TET2	mutated	c.3312dup	p.Ile1105Tyrfs*25	2.20
TET2	mutated	c.3312dup	p.Ile1105Tyrfs*25	5.60
TET2	mutated	c.3320C>A	p.Ser1107*	5.20
TET2	mutated	c.3339dup	p.Thr1114Tyrfs*16	12.20
TET2	mutated	c.3340dup	p.Thr1114Asnfs*16	8.00
TET2	mutated	c.3343_3344del	p.Pro1115Tyrfs*14	5.10
TET2	mutated	c.3344del	p.Pro1115Leufs*2	2.64
TET2	mutated	c.3344del	p.Pro1115Leufs*2	9.50
TET2	mutated	c.3353del	p.Asn1118Ilefs*19	4.26
TET2	mutated	c.3364dup	p.Thr1122Asnfs*8	7.50

TET2	mutated	c.3378_3405del	p.Gln1127*	12.00
TET2	mutated	c.3384T>A	p.Tyr1128*	6.10
TET2	mutated	c.3385_3386del	p.Asp1129Phefs*12	2.92
TET2	mutated	c.3395del	p.Ser1132Phefs*5	2.44
TET2	mutated	c.3404G>A	p.Cys1135Tyr	2.17
TET2	mutated	c.3404G>A	p.Cys1135Tyr	2.60
TET2	mutated	c.3404G>A	p.Cys1135Tyr	2.81
TET2	mutated	c.3404G>A	p.Cys1135Tyr	4.42
TET2	mutated	c.3404G>A	p.Cys1135Tyr	5.70
TET2	mutated	c.3405T>G	p.Cys1135Trp	3.03
TET2	mutated	c.3412C>T	p.Gln1138*	30.30
TET2	mutated	c.3415del	p.Ile1139Leufs*13	29.80
TET2	mutated	c.3421_3422del	p.Glu1141Lysfs*3	35.30
TET2	mutated	c.3443A>G	p.Tyr1148Cys	2.28
TET2	mutated	c.3454G>A	p.Gly1152Arg	8.60
TET2	mutated	c.3461G>A	p.Gly1154Asp	2.42
TET2	mutated	c.3462_3463insA	p.Pro1155Thrfs*2	4.19
TET2	mutated	c.346C>T	p.Gln116*	5.50
TET2	mutated	c.3470_3471del	p.Val1157Glyfs*4	2.47
TET2	mutated	c.3473C>T	p.Ala1158Val	8.80
TET2	mutated	c.3476_3482del	p.Ala1159Glufs*65	2.90
TET2	mutated	c.3480dup	p.Arg1161*	4.05
TET2	mutated	c.3493G>T	p.Glu1165*	33.60
TET2	mutated	c.3495A>T	p.Glu1165Asp	5.90
TET2	mutated	c.3496G>A	p.Glu1166Lys	37.00
TET2	mutated	c.3499A>G	p.Arg1167Gly	3.25
TET2	mutated	c.3500G>A	p.Arg1167Lys	3.08
TET2	mutated	c.3500G>A	p.Arg1167Lys	10.00
TET2	mutated	c.3500G>A	p.Arg1167Lys	10.30
TET2	mutated	c.3508C>T	p.Gln1170*	9.60
TET2	mutated	c.3508C>T	p.Gln1170*	17.40
TET2	mutated	c.3528G>C	p.Arg1176Ser	2.11
TET2	mutated	c.3538_3556dup	p.Glu1186Glyfs*6	20.00
TET2	mutated	c.3545_3556delinsTC	p.Tyr1182Phefs*41	2.44
TET2	mutated	c.3548_3553delCTGGTA	p.Thr1183_Gly1184del	3.29
TET2	mutated	c.3552del	p.Glu1186Lysfs*40	8.60
TET2	mutated	c.3571C>T	p.Gln1191*	5.20
TET2	mutated	c.3571C>T	p.Gln1191*	21.70
TET2	mutated	c.3579T>A	p.Cys1193*	7.80
TET2	mutated	c.3579T>G	p.Cys1193Trp	3.07
TET2	mutated	c.3581C>A	p.Pro1194His	2.74
TET2	mutated	c.3581C>G	p.Pro1194Arg	4.62
TET2	mutated	c.3589A>G	p.Lys1197Glu	2.09
TET2	mutated	c.3589A>G	p.Lys1197Glu	2.26
TET2	mutated	c.3589A>G	p.Lys1197Glu	3.04
TET2	mutated	c.3589A>G	p.Lys1197Glu	6.90
TET2	mutated	c.3589A>T	p.Lys1197*	3.19

TET2	mutated	c.3593G>A	p.Trp1198*	13.10
TET2	mutated	c.35A>G	p.Asn12Ser	5.90
TET2	mutated	c.3618_3619insAC	p.Glu1207Thrfs*20	7.70
TET2	mutated	c.3619G>A	p.Glu1207Lys	2.15
TET2	mutated	c.3622_3649dup	p.Ala1217Glufs*15	8.90
TET2	mutated	c.3626_3636del	p.Leu1209Argfs*10	3.00
TET2	mutated	c.3626T>G	p.Leu1209Arg	11.90
TET2	mutated	c.3629del	p.Leu1210Argfs*16	16.60
TET2	mutated	c.3629T>C	p.Leu1210Pro	2.61
TET2	mutated	c.3629T>G	p.Leu1210Arg	2.07
TET2	mutated	c.3631T>C	p.Cys1211Arg	2.20
TET2	mutated	c.3632G>A	p.Cys1211Tyr	4.07
TET2	mutated	c.3632G>A	p.Cys1211Tyr	10.60
TET2	mutated	c.3632G>A	p.Cys1211Tyr	20.90
TET2	mutated	c.3637G>T	p.Val1213Leu	6.00
TET2	mutated	c.3640C>T	p.Arg1214Trp	2.08
TET2	mutated	c.3640C>T	p.Arg1214Trp	2.39
TET2	mutated	c.3640C>T	p.Arg1214Trp	2.72
TET2	mutated	c.3640C>T	p.Arg1214Trp	6.50
TET2	mutated	c.3640C>T	p.Arg1214Trp	6.50
TET2	mutated	c.3641G>A	p.Arg1214Gln	2.38
TET2	mutated	c.3641G>A	p.Arg1214Gln	29.70
TET2	mutated	c.3641G>A	p.Arg1214Gln	35.40
TET2	mutated	c.3641G>C	p.Arg1214Pro	31.00
TET2	mutated	c.3646C>G	p.Arg1216Gly	23.10
TET2	mutated	c.3646C>T	p.Arg1216*	2.09
TET2	mutated	c.3646C>T	p.Arg1216*	2.11
TET2	mutated	c.3646C>T	p.Arg1216*	2.84
TET2	mutated	c.3646C>T	p.Arg1216*	3.70
TET2	mutated	c.3646C>T	p.Arg1216*	4.39
TET2	mutated	c.3655C>T	p.His1219Tyr	3.12
TET2	mutated	c.3655C>T	p.His1219Tyr	11.60
TET2	mutated	c.3656A>G	p.His1219Arg	2.93
TET2	mutated	c.3656A>T	p.His1219Leu	9.50
TET2	mutated	c.3662G>A	p.Cys1221Tyr	17.50
TET2	mutated	c.3662G>T	p.Cys1221Phe	2.94
TET2	mutated	c.3671C>A	p.Ala1224Glu	31.20
TET2	mutated	c.368del	p.Arg123Leufs*5	7.00
TET2	mutated	c.3697T>G	p.Trp1233Gly	19.60
TET2	mutated	c.3698G>A	p.Trp1233*	24.60
TET2	mutated	c.3699G>A	p.Trp1233*	3.76
TET2	mutated	c.3699G>A	p.Trp1233*	28.20
TET2	mutated	c.3703G>A	p.Gly1235Arg	3.70
TET2	mutated	c.3705_3713del	p.Ile1236_Leu1238del	4.34
TET2	mutated	c.3706_3708del	p.Ile1236del	3.60
TET2	mutated	c.3710del	p.Pro1237Argfs*16	5.30
TET2	mutated	c.3732_3733del	p.Tyr1245Leufs*22	3.92

TET2	mutated	c.3732_3733del	p.Tyr1245Leufs*22	15.00
TET2	mutated	c.3732_3733del	p.Tyr1245Leufs*22	29.50
TET2	mutated	c.3733_3737del	p.Tyr1245Glyfs*21	5.40
TET2	mutated	c.3733_3737del	p.Tyr1245Glyfs*21	10.60
TET2	mutated	c.3733_3737del	p.Tyr1245Glyfs*21	26.80
TET2	mutated	c.3733_3737del	p.Tyr1245Glyfs*21	32.20
TET2	mutated	c.3733T>A	p.Tyr1245Asn	8.00
TET2	mutated	c.3734A>G	p.Tyr1245Cys	2.02
TET2	mutated	c.3734A>G	p.Tyr1245Cys	2.03
TET2	mutated	c.3743T>C	p.Leu1248Pro	9.30
TET2	mutated	c.3743T>C	p.Leu1248Pro	14.60
TET2	mutated	c.3755T>A	p.Leu1252Gln	4.79
TET2	mutated	c.3763dup	p.Tyr1255Leufs*13	25.40
TET2	mutated	c.3765C>G	p.Tyr1255*	5.20
TET2	mutated	c.3765del	p.Tyr1255*	2.95
TET2	mutated	c.3782G>A	p.Arg1261His	3.23
TET2	mutated	c.3782G>A	p.Arg1261His	7.80
TET2	mutated	c.3782G>A	p.Arg1261His	8.30
TET2	mutated	c.3782G>C	p.Arg1261Pro	7.80
TET2	mutated	c.3784C>T	p.Arg1262Trp	19.60
TET2	mutated	c.3788G>A	p.Cys1263Tyr	4.70
TET2	mutated	c.3788G>A	p.Cys1263Tyr	5.20
TET2	mutated	c.3790G>C	p.Ala1264Pro	2.40
TET2	mutated	c.3798_3801del	p.Asn1266Lysfs*96	3.76
TET2	mutated	c.3803_3803+1del	p.splice site mutation	9.60
TET2	mutated	c.3803del	p.Glu1268Glyfs*95	28.20
TET2	mutated	c.3808dup	p.Thr1270Asnfs*30	11.00
TET2	mutated	c.3811_3812insA	p.Cys1271*	32.00
TET2	mutated	c.3812_3813insAGG	p.Cys1271delins*	2.27
TET2	mutated	c.3812dup	p.Cys1271Trpfs*29	3.88
TET2	mutated	c.3812dup	p.Cys1271Trpfs*29	11.20
TET2	mutated	c.3812dup	p.Cys1271Trpfs*29	13.60
TET2	mutated	c.3812G>A	p.Cys1271Tyr	2.96
TET2	mutated	c.3813C>A	p.Cys1271*	2.53
TET2	mutated	c.3813C>A	p.Cys1271*	2.59
TET2	mutated	c.3813C>A	p.Cys1271*	16.20
TET2	mutated	c.3813C>G	p.Cys1271Trp	2.93
TET2	mutated	c.3818G>A	p.Cys1273Tyr	2.14
TET2	mutated	c.3818G>C	p.Cys1273Ser	6.30
TET2	mutated	c.3818G>C	p.Cys1273Ser	6.50
TET2	mutated	c.3818G>C	p.Cys1273Ser	7.40
TET2	mutated	c.3820C>G	p.Gln1274Glu	5.00
TET2	mutated	c.3820C>T	p.Gln1274*	2.46
TET2	mutated	c.3820C>T	p.Gln1274*	5.40
TET2	mutated	c.3821_3822del	p.Gln1274Argfs*25	2.10
TET2	mutated	c.3821_3822del	p.Gln1274Argfs*25	5.60
TET2	mutated	c.3823_3829del	p.Gly1275Ilefs*86	8.10

TET2	mutated	c.3845G>A	p.Gly1282Asp	3.65
TET2	mutated	c.3845G>A	p.Gly1282Asp	7.80
TET2	mutated	c.3848C>A	p.Ala1283Asp	3.15
TET2	mutated	c.3854_3856del	p.Phe1285del	4.12
TET2	mutated	c.3854_3856del	p.Phe1285del	6.10
TET2	mutated	c.3854T>C	p.Phe1285Ser	3.82
TET2	mutated	c.3855_3859del	p.Ser1286Trpfs*12	2.14
TET2	mutated	c.3861del	p.Phe1287Leufs*76	20.90
TET2	mutated	c.3862G>A	p.Gly1288Ser	5.00
TET2	mutated	c.3862G>C	p.Gly1288Arg	10.50
TET2	mutated	c.3863G>A	p.Gly1288Asp	2.03
TET2	mutated	c.3863G>A	p.Gly1288Asp	3.79
TET2	mutated	c.3863G>A	p.Gly1288Asp	4.07
TET2	mutated	c.3866G>C	p.Cys1289Ser	13.70
TET2	mutated	c.3866G>T	p.Cys1289Phe	11.80
TET2	mutated	c.3869C>T	p.Ser1290Leu	5.50
TET2	mutated	c.3877A>G	p.Met1293Val	40.90
TET2	mutated	c.3879dup	p.Tyr1294Valfs*6	9.60
TET2	mutated	c.3880T>A	p.Tyr1294Asn	10.80
TET2	mutated	c.3881A>G	p.Tyr1294Cys	8.40
TET2	mutated	c.3893del	p.Cys1298Leufs*65	3.20
TET2	mutated	c.3893G>C	p.Cys1298Ser	2.42
TET2	mutated	c.3893G>T	p.Cys1298Phe	2.43
TET2	mutated	c.3894dup	p.Lys1299*	2.26
TET2	mutated	c.3895A>G	p.Lys1299Glu	10.40
TET2	mutated	c.3898T>A	p.Phe1300Ile	5.70
TET2	mutated	c.3898T>C	p.Phe1300Leu	5.60
TET2	mutated	c.3898T>G	p.Phe1300Val	2.02
TET2	mutated	c.3902_3908delinsT	p.Ala1301_Ser1303delinsVal	25.00
TET2	mutated	c.3904A>G	p.Arg1302Gly	3.62
TET2	mutated	c.3904A>G	p.Arg1302Gly	17.20
TET2	mutated	c.3905G>A	p.Arg1302Lys	4.44
TET2	mutated	c.3919A>G	p.Arg1307Gly	2.32
TET2	mutated	c.391dup	p.Arg131Lysfs*5	5.20
TET2	mutated	c.392_395del	p.Arg131Ilefs*13	3.09
TET2	mutated	c.3921del	p.Lys1308Serfs*55	2.87
TET2	mutated	c.3921del	p.Lys1308Serfs*55	3.28
TET2	mutated	c.3921del	p.Lys1308Serfs*55	3.33
TET2	mutated	c.3921del	p.Lys1308Serfs*55	9.30
TET2	mutated	c.3926T>G	p.Phe1309Cys	4.22
TET2	mutated	c.3927del	p.Phe1309Leufs*54	8.20
TET2	mutated	c.3927del	p.Phe1309Leufs*54	9.10
TET2	mutated	c.3927T>A	p.Phe1309Leu	3.67
TET2	mutated	c.3927T>A	p.Phe1309Leu	6.50
TET2	mutated	c.3927T>G	p.Phe1309Leu	2.43
TET2	mutated	c.3927T>G	p.Phe1309Leu	3.23
TET2	mutated	c.3934del	p.Asp1314Metfs*49	10.50

TET2	mutated	c.3934dup	p.Leu1312Profs*4	7.90
TET2	mutated	c.3949A>G	p.Lys1317Glu	40.00
TET2	mutated	c.3951del	p.Glu1318Argfs*45	2.42
TET2	mutated	c.3961A>T	p.Lys1321*	2.15
TET2	mutated	c.3965T>A	p.Leu1322Gln	3.27
TET2	mutated	c.3965T>A	p.Leu1322Gln	9.00
TET2	mutated	c.3965T>C	p.Leu1322Pro	2.08
TET2	mutated	c.3967G>T	p.Glu1323*	4.52
TET2	mutated	c.3973_3977del	p.His1325Alafs*12	3.52
TET2	mutated	c.3977del	p.Leu1326Cysfs*37	8.50
TET2	mutated	c.3979C>T	p.Gln1327*	3.47
TET2	mutated	c.3986T>C	p.Leu1329Pro	4.50
TET2	mutated	c.3991A>C	p.Thr1331Pro	31.50
TET2	mutated	c.3997dup	p.Met1333Asnfs*6	21.60
TET2	mutated	c.3998T>C	p.Met1333Thr	4.19
TET2	mutated	c.4009T>A	p.Tyr1337Asn	29.40
TET2	mutated	c.4012A>T	p.Lys1338*	5.00
TET2	mutated	c.4022del	p.Ala1341Aspfs*22	10.00
TET2	mutated	c.4024C>T	p.Pro1342Ser	2.09
TET2	mutated	c.4031C>A	p.Ala1344Glu	2.58
TET2	mutated	c.4034A>G	p.Tyr1345Cys	2.16
TET2	mutated	c.4039_4041del	p.Asn1347del	3.69
TET2	mutated	c.4043A>G	p.Gln1348Arg	5.10
TET2	mutated	c.4043A>G	p.Gln1348Arg	18.60
TET2	mutated	c.4043dup	p.Ile1349Aspfs*2	5.50
TET2	mutated	c.4045A>T	p.Ile1349Phe	6.30
TET2	mutated	c.4045A>T	p.Ile1349Phe	31.10
TET2	mutated	c.4046_4062del	p.Ile1349Serfs*46	3.67
TET2	mutated	c.4053T>A	p.Tyr1351*	4.62
TET2	mutated	c.4054G>T	p.Glu1352*	2.84
TET2	mutated	c.4062_4063del	p.Arg1354Serfs*46	30.80
TET2	mutated	c.4064del	p.Ala1355Aspfs*8	6.70
TET2	mutated	c.4072dup	p.Cys1358Leufs*43	7.70
TET2	mutated	c.4075C>A	p.Arg1359Ser	8.20
TET2	mutated	c.4075C>G	p.Arg1359Gly	2.07
TET2	mutated	c.4075C>T	p.Arg1359Cys	5.20
TET2	mutated	c.4075C>T	p.Arg1359Cys	8.50
TET2	mutated	c.4076del	p.Arg1359Leufs*4	29.60
TET2	mutated	c.4076G>A	p.Arg1359His	2.08
TET2	mutated	c.4076G>A	p.Arg1359His	3.18
TET2	mutated	c.4076G>C	p.Arg1359Pro	3.94
TET2	mutated	c.4081G>T	p.Gly1361Cys	4.56
TET2	mutated	c.4081G>T	p.Gly1361Cys	6.80
TET2	mutated	c.4081G>T	p.Gly1361Cys	9.20
TET2	mutated	c.4081G>T	p.Gly1361Cys	19.30
TET2	mutated	c.4090G>T	p.Glu1364*	17.70
TET2	mutated	c.4096C>T	p.Arg1366Cys	19.00

TET2	mutated	c.4097G>A	p.Arg1366His	2.15
TET2	mutated	c.4097G>A	p.Arg1366His	2.71
TET2	mutated	c.4097G>A	p.Arg1366His	3.75
TET2	mutated	c.4098del	p.Pro1367Hisfs*81	5.10
TET2	mutated	c.4100C>A	p.Pro1367Gln	3.19
TET2	mutated	c.4106C>T	p.Ser1369Leu	27.10
TET2	mutated	c.4107del	p.Val1371Serfs*77	4.35
TET2	mutated	c.4109G>A	p.Gly1370Glu	13.00
TET2	mutated	c.4109G>T	p.Gly1370Val	6.30
TET2	mutated	c.4112T>A	p.Val1371Asp	3.03
TET2	mutated	c.4115C>T	p.Thr1372Ile	2.82
TET2	mutated	c.4121G>A	p.Cys1374Tyr	2.79
TET2	mutated	c.4121G>A	p.Cys1374Tyr	3.07
TET2	mutated	c.4125_4145del	p.Leu1375_His1382delinsPhe	4.55
TET2	mutated	c.4126G>A	p.Asp1376Asn	2.54
TET2	mutated	c.4126G>A	p.Asp1376Asn	2.71
TET2	mutated	c.4126G>A	p.Asp1376Asn	6.40
TET2	mutated	c.4126G>T	p.Asp1376Tyr	4.01
TET2	mutated	c.4129T>G	p.Phe1377Val	3.52
TET2	mutated	c.412C>T	p.Gln138*	3.27
TET2	mutated	c.4132T>C	p.Cys1378Arg	2.63
TET2	mutated	c.4132T>C	p.Cys1378Arg	11.20
TET2	mutated	c.4134T>A	p.Cys1378*	23.40
TET2	mutated	c.4136C>A	p.Ala1379Asp	16.30
TET2	mutated	c.4136C>T	p.Ala1379Val	7.50
TET2	mutated	c.4138C>T	p.His1380Tyr	2.14
TET2	mutated	c.4138C>T	p.His1380Tyr	2.92
TET2	mutated	c.4144C>A	p.His1382Asn	9.20
TET2	mutated	c.4144C>T	p.His1382Tyr	2.40
TET2	mutated	c.4144C>T	p.His1382Tyr	6.40
TET2	mutated	c.4146C>G	p.His1382Gln	2.40
TET2	mutated	c.4150_4151del	p.Asp1384Leufs*16	6.50
TET2	mutated	c.4151A>G	p.Asp1384Gly	2.23
TET2	mutated	c.4151A>G	p.Asp1384Gly	6.90
TET2	mutated	c.4154del	p.Leu1385Cysfs*63	3.73
TET2	mutated	c.4155dup	p.His1386Alafs*15	2.17
TET2	mutated	c.4160A>G	p.Asn1387Ser	4.68
TET2	mutated	c.4165C>T	p.Gln1389*	7.90
TET2	mutated	c.4165C>T	p.Gln1389*	29.80
TET2	mutated	c.4172G>A	p.Gly1391Asp	7.00
TET2	mutated	c.4189A>G	p.Thr1397Ala	7.60
TET2	mutated	c.4190C>T	p.Thr1397Ile	3.86
TET2	mutated	c.421_422dup	p.Asp143Profs*3	2.91
TET2	mutated	c.4210C>T	p.Arg1404*	22.10
TET2	mutated	c.4240C>G	p.Gln1414Glu	11.50
TET2	mutated	c.4241A>G	p.Gln1414Arg	2.46
TET2	mutated	c.4242G>C	p.Gln1414His	5.90

TET2	mutated	c.4242G>T	p.Gln1414His	4.52
TET2	mutated	c.4245_4272dup	p.Asp1425Serfs*10	2.13
TET2	mutated	c.4246C>T	p.His1416Tyr	3.42
TET2	mutated	c.4248C>A	p.His1416Gln	3.92
TET2	mutated	c.4256C>G	p.Pro1419Arg	3.17
TET2	mutated	c.4256del	p.Pro1419Leufs*29	6.90
TET2	mutated	c.4259T>G	p.Leu1420*	22.00
TET2	mutated	c.4263C>G	p.Tyr1421*	34.90
TET2	mutated	c.4287del	p.Phe1429Leufs*19	2.58
TET2	mutated	c.4290del	p.Ser1431Valfs*17	2.93
TET2	mutated	c.4309del	p.Glu1437Argfs*11	2.40
TET2	mutated	c.4317_4319delinsC	p.Lys1439Asnfs*38	14.40
TET2	mutated	c.4317del	p.Lys1439Asnfs*9	12.90
TET2	mutated	c.4317dup	p.Arg1440Thrfs*38	2.32
TET2	mutated	c.4317dup	p.Arg1440Thrfs*38	3.75
TET2	mutated	c.4317dup	p.Arg1440Thrfs*38	5.40
TET2	mutated	c.4317dup	p.Arg1440Thrfs*38	6.60
TET2	mutated	c.4317dup	p.Arg1440Thrfs*38	14.80
TET2	mutated	c.4350del	p.Arg1451Glyfs*7	2.87
TET2	mutated	c.4354C>T	p.Arg1452*	2.37
TET2	mutated	c.4354C>T	p.Arg1452*	3.30
TET2	mutated	c.4354C>T	p.Arg1452*	9.60
TET2	mutated	c.4354C>T	p.Arg1452*	9.80
TET2	mutated	c.4354C>T	p.Arg1452*	12.00
TET2	mutated	c.4354C>T	p.Arg1452*	18.00
TET2	mutated	c.4354C>T	p.Arg1452*	18.30
TET2	mutated	c.4354C>T	p.Arg1452*	22.10
TET2	mutated	c.4361_4377del	p.Val1454Alafs*18	5.00
TET2	mutated	c.4370T>G	p.Leu1457*	2.37
TET2	mutated	c.4388del	p.Thr1463Ilefs*7	29.40
TET2	mutated	c.4390del	p.Cys1464Alafs*6	3.32
TET2	mutated	c.4393C>G	p.Arg1465Gly	10.40
TET2	mutated	c.4393C>T	p.Arg1465*	2.20
TET2	mutated	c.4393C>T	p.Arg1465*	4.26
TET2	mutated	c.4393C>T	p.Arg1465*	6.20
TET2	mutated	c.4393C>T	p.Arg1465*	8.00
TET2	mutated	c.4393C>T	p.Arg1465*	12.00
TET2	mutated	c.4393C>T	p.Arg1465*	32.60
TET2	mutated	c.4396C>T	p.Gln1466*	14.90
TET2	mutated	c.4403_4407del	p.Lys1468Argfs*8	25.80
TET2	mutated	c.4419del	p.Ala1474Leufs*97	27.90
TET2	mutated	c.442A>T	p.Lys148*	2.88
TET2	mutated	c.4434del	p.Lys1478Asnfs*93	12.40
TET2	mutated	c.444_447del	p.Lys148Asnfs*3	3.19
TET2	mutated	c.4457C>G	p.Ser1486*	4.10
TET2	mutated	c.4462A>T	p.Lys1488*	3.44
TET2	mutated	c.4466del	p.Asn1489Metfs*82	2.35

TET2	mutated	c.4468G>T	p.Glu1490*	9.40
TET2	mutated	c.4481C>A	p.Ser1494*	5.40
TET2	mutated	c.4490C>A	p.Ser1497*	7.30
TET2	mutated	c.4501C>T	p.Gln1501*	3.01
TET2	mutated	c.4513G>A	p.Ala1505Thr	44.50
TET2	mutated	c.4516dup	p.Ser1506Lysfs*4	3.13
TET2	mutated	c.4519C>T	p.Gln1507*	2.62
TET2	mutated	c.452_453del	p.Val151Glufs*10	7.90
TET2	mutated	c.4522del	p.Ala1508Leufs*63	2.23
TET2	mutated	c.4522del	p.Ala1508Leufs*63	3.02
TET2	mutated	c.4523del	p.Ala1508Valfs*63	21.10
TET2	mutated	c.4532T>A	p.Leu1511*	27.10
TET2	mutated	c.4532T>A	p.Leu1511*	31.50
TET2	mutated	c.4546_4550del	p.Arg1516Phefs*60	3.42
TET2	mutated	c.4546C>T	p.Arg1516*	2.30
TET2	mutated	c.4546C>T	p.Arg1516*	2.63
TET2	mutated	c.4546C>T	p.Arg1516*	5.60
TET2	mutated	c.4546C>T	p.Arg1516*	5.80
TET2	mutated	c.4546C>T	p.Arg1516*	5.90
TET2	mutated	c.4546C>T	p.Arg1516*	6.20
TET2	mutated	c.4546C>T	p.Arg1516*	6.50
TET2	mutated	c.4546C>T	p.Arg1516*	26.00
TET2	mutated	c.4549_4552delinsTC	p.Leu1517Serfs*60	2.13
TET2	mutated	c.4553C>G	p.Ser1518*	3.50
TET2	mutated	c.4553C>G	p.Ser1518*	16.20
TET2	mutated	c.4559del	p.Pro1520Glnfs*51	32.60
TET2	mutated	c.4567C>T	p.Gln1523*	5.30
TET2	mutated	c.4579C>T	p.Gln1527*	6.90
TET2	mutated	c.4585C>T	p.Gln1529*	2.07
TET2	mutated	c.4585C>T	p.Gln1529*	9.40
TET2	mutated	c.4594C>T	p.Gln1532*	2.56
TET2	mutated	c.4594C>T	p.Gln1532*	3.82
TET2	mutated	c.4595_4602del	p.Gln1532Profs*43	2.79
TET2	mutated	c.4600C>T	p.Gln1534*	5.00
TET2	mutated	c.4604del	p.Pro1535Hisfs*36	32.90
TET2	mutated	c.4609C>T	p.Gln1537*	2.04
TET2	mutated	c.4609C>T	p.Gln1537*	4.63
TET2	mutated	c.4615C>T	p.Gln1539*	2.12
TET2	mutated	c.4615C>T	p.Gln1539*	5.00
TET2	mutated	c.4621C>T	p.Gln1541*	2.00
TET2	mutated	c.4639C>T	p.Gln1547*	7.70
TET2	mutated	c.4642C>T	p.Gln1548*	5.30
TET2	mutated	c.4657C>T	p.Gln1553*	4.44
TET2	mutated	c.4661_4662insTCTAGCAGCAGC AGCCACATCACCCTCAGAC	p.Glu1555Leufs*33	14.00
TET2	mutated	c.4664_4665del	p.Glu1555Valfs*22	3.74
TET2	mutated	c.4664_4665del	p.Glu1555Valfs*22	8.10

TET2	mutated	c.4668dup	p.Val1557Cysfs*21	5.10
TET2	mutated	c.4668dup	p.Val1557Cysfs*21	6.60
TET2	mutated	c.4678dup	p.Tyr1560Leufs*18	3.43
TET2	mutated	c.4703del	p.Pro1568Hisfs*3	6.20
TET2	mutated	c.4714C>T	p.Arg1572Trp	43.30
TET2	mutated	c.4748del	p.Ser1583Tyrfs*13	4.07
TET2	mutated	c.4753_4754del	p.Thr1585Phefs*28	5.00
TET2	mutated	c.4761dup	p.Ile1588Tyrfs*26	2.91
TET2	mutated	c.4761dup	p.Ile1588Tyrfs*26	4.11
TET2	mutated	c.4766del	p.Tyr1589Leufs*7	25.70
TET2	mutated	c.4794T>G	p.Tyr1598*	9.80
TET2	mutated	c.4826del	p.Leu1609*	16.20
TET2	mutated	c.4838del	p.Asn1613Ilefs*3	23.10
TET2	mutated	c.483dup	p.Asp162Argfs*9	2.30
TET2	mutated	c.4853dup	p.Tyr1618*	9.20
TET2	mutated	c.4870C>T	p.Gln1624*	2.88
TET2	mutated	c.4870C>T	p.Gln1624*	34.40
TET2	mutated	c.488del	p.Phe163Serfs*20	6.60
TET2	mutated	c.4893T>A	p.Tyr1631*	6.40
TET2	mutated	c.4894C>T	p.Gln1632*	4.34
TET2	mutated	c.4894C>T	p.Gln1632*	5.00
TET2	mutated	c.4894C>T	p.Gln1632*	6.40
TET2	mutated	c.4894C>T	p.Gln1632*	28.00
TET2	mutated	c.4896_4914del	p.Cys1633Trpfs*56	4.34
TET2	mutated	c.4909del	p.Leu1637Tyrfs*58	10.60
TET2	mutated	c.4909del	p.Leu1637Tyrfs*58	25.60
TET2	mutated	c.4911_4928delinsGCTG	p.Ser1638Leufs*18	31.00
TET2	mutated	c.4931dup	p.Tyr1645Ilefs*16	2.30
TET2	mutated	c.4944del	p.Tyr1649Ilefs*46	2.71
TET2	mutated	c.4954dup	p.Gln1652Profs*9	2.02
TET2	mutated	c.4960C>T	p.Gln1654*	9.20
TET2	mutated	c.4977T>A	p.Tyr1659*	5.30
TET2	mutated	c.4983T>A	p.Tyr1661*	5.50
TET2	mutated	c.499del	p.Ser167Glnfs*16	19.00
TET2	mutated	c.5001_5004del	p.Lys1669Serfs*25	3.80
TET2	mutated	c.5038C>T	p.Gln1680*	5.40
TET2	mutated	c.5068dup	p.Thr1690Asnfs*3	2.22
TET2	mutated	c.507dup	p.Asn170*	2.09
TET2	mutated	c.5084_5085delinsA	p.Gly1695Aspfs*24	7.90
TET2	mutated	c.5095del	p.Gln1699Lysfs*20	5.30
TET2	mutated	c.5095dup	p.Gln1699Profs*14	11.00
TET2	mutated	c.5107G>T	p.Gly1703*	5.40
TET2	mutated	c.5127T>A	p.Cys1709*	6.40
TET2	mutated	c.5138del	p.Pro1713Glnfs*6	9.40
TET2	mutated	c.5142del	p.Asn1714Lysfs*5	23.30
TET2	mutated	c.5146_5147insTG	p.His1716Leufs*4	2.30
TET2	mutated	c.5162_5163delinsGA	p.Leu1721*	23.60

TET2	mutated	c.5166del	p.Pro1723Leufs*22	11.50
TET2	mutated	c.5171dup	p.Tyr1724*	22.10
TET2	mutated	c.5188del	p.Asp1730Metfs*15	2.74
TET2	mutated	c.520_523del	p.Pro174Lysfs*8	3.78
TET2	mutated	c.5219T>A	p.Leu1740*	11.60
TET2	mutated	c.5237_5247del	p.Asn1746Argfs*3	2.27
TET2	mutated	c.5243dup	p.Asn1748Lysfs*5	14.70
TET2	mutated	c.5256_5257insTA	p.Asn1753*	36.10
TET2	mutated	c.5258del	p.Asn1753Metfs*10	6.30
TET2	mutated	c.5258del	p.Asn1753Metfs*10	10.00
TET2	mutated	c.5258dup	p.Asn1753Lysfs*3	5.90
TET2	mutated	c.5323del	p.Ser1775Alafs*45	10.40
TET2	mutated	c.5338del	p.Leu1780Cysfs*40	6.30
TET2	mutated	c.5338del	p.Leu1780Cysfs*40	22.50
TET2	mutated	c.5354del	p.Lys1785Argfs*35	3.72
TET2	mutated	c.5356G>A	p.Glu1786Lys	3.38
TET2	mutated	c.5387G>T	p.Gly1796Val	43.30
TET2	mutated	c.538C>T	p.Gln180*	2.05
TET2	mutated	c.5393C>G	p.Ser1798*	3.66
TET2	mutated	c.5417del	p.His1806Leufs*14	2.65
TET2	mutated	c.5431del	p.Cys1811Valfs*9	9.80
TET2	mutated	c.5437C>T	p.Gln1813*	4.12
TET2	mutated	c.5447dup	p.Leu1816Phefs*6	6.90
TET2	mutated	c.5457_5460del	p.Ser1820Metfs*12	17.60
TET2	mutated	c.5462_5471dup	p.Gln1825Cysfs*2	2.21
TET2	mutated	c.5462dup	p.Asp1821Glufs*3	2.00
TET2	mutated	c.5482C>T	p.Gln1828*	2.39
TET2	mutated	c.5482C>T	p.Gln1828*	29.90
TET2	mutated	c.5486del	p.Pro1829Hisfs*4	11.30
TET2	mutated	c.5493_5494del	p.Leu1832Serfs*13	2.09
TET2	mutated	c.5500_5513del	p.Gln1834Trpfs*7	16.00
TET2	mutated	c.552_553dup	p.Gln185Argfs*23	3.45
TET2	mutated	c.5521G>T	p.Glu1841*	3.62
TET2	mutated	c.5528del	p.Asn1843Thrfs*44	2.78
TET2	mutated	c.5539_5542del	p.Trp1847Glnfs*39	4.00
TET2	mutated	c.5540G>A	p.Trp1847*	4.37
TET2	mutated	c.5554C>T	p.Gln1852*	19.40
TET2	mutated	c.5554C>T	p.Gln1852*	31.50
TET2	mutated	c.5566G>A	p.Asp1856Asn	45.60
TET2	mutated	c.5579G>A	p.Gly1860Glu	7.50
TET2	mutated	c.5581G>A	p.Gly1861Arg	12.20
TET2	mutated	c.5582dup	p.Val1862Serfs*13	8.80
TET2	mutated	c.5582G>A	p.Gly1861Glu	9.60
TET2	mutated	c.5582G>T	p.Gly1861Val	2.57
TET2	mutated	c.5588C>T	p.Ala1863Val	2.36
TET2	mutated	c.5588C>T	p.Ala1863Val	11.60
TET2	mutated	c.5602C>G	p.His1868Asp	3.77

TET2	mutated	c.5603A>G	p.His1868Arg	4.07
TET2	mutated	c.5603A>G	p.His1868Arg	5.30
TET2	mutated	c.5609del	p.Ser1870*	23.30
TET2	mutated	c.5611del	p.Ile1871Phefs*16	3.88
TET2	mutated	c.5615T>G	p.Leu1872Arg	2.24
TET2	mutated	c.5615T>G	p.Leu1872Arg	3.78
TET2	mutated	c.5615T>G	p.Leu1872Arg	21.80
TET2	mutated	c.5618T>A	p.Ile1873Asn	2.01
TET2	mutated	c.5618T>C	p.Ile1873Thr	2.00
TET2	mutated	c.5618T>C	p.Ile1873Thr	2.09
TET2	mutated	c.5618T>C	p.Ile1873Thr	2.48
TET2	mutated	c.5618T>C	p.Ile1873Thr	2.48
TET2	mutated	c.5618T>C	p.Ile1873Thr	2.53
TET2	mutated	c.5618T>C	p.Ile1873Thr	2.63
TET2	mutated	c.5618T>C	p.Ile1873Thr	2.94
TET2	mutated	c.5618T>C	p.Ile1873Thr	3.08
TET2	mutated	c.5618T>C	p.Ile1873Thr	3.69
TET2	mutated	c.5618T>C	p.Ile1873Thr	3.88
TET2	mutated	c.5618T>C	p.Ile1873Thr	4.48
TET2	mutated	c.5618T>C	p.Ile1873Thr	4.83
TET2	mutated	c.5618T>C	p.Ile1873Thr	5.50
TET2	mutated	c.5618T>C	p.Ile1873Thr	7.70
TET2	mutated	c.5618T>C	p.Ile1873Thr	14.20
TET2	mutated	c.5618T>C	p.Ile1873Thr	16.20
TET2	mutated	c.5618T>C	p.Ile1873Thr	24.40
TET2	mutated	c.5618T>C	p.Ile1873Thr	37.30
TET2	mutated	c.5620G>A	p.Glu1874Lys	39.00
TET2	mutated	c.5620G>T	p.Glu1874*	9.80
TET2	mutated	c.5622G>C	p.Glu1874Asp	2.17
TET2	mutated	c.5623T>G	p.Cys1875Gly	3.81
TET2	mutated	c.5627C>G	p.Ala1876Gly	21.50
TET2	mutated	c.5641C>T	p.His1881Tyr	3.51
TET2	mutated	c.5642A>G	p.His1881Arg	2.09
TET2	mutated	c.5642A>G	p.His1881Arg	14.70
TET2	mutated	c.5642A>T	p.His1881Leu	27.60
TET2	mutated	c.5645C>T	p.Ala1882Val	15.60
TET2	mutated	c.5650A>G	p.Thr1884Ala	2.24
TET2	mutated	c.5650A>G	p.Thr1884Ala	2.75
TET2	mutated	c.5650A>G	p.Thr1884Ala	5.30
TET2	mutated	c.5650A>G	p.Thr1884Ala	5.80
TET2	mutated	c.5654del	p.Pro1885Leufs*2	2.55
TET2	mutated	c.5654del	p.Pro1885Leufs*2	4.34
TET2	mutated	c.5666C>G	p.Pro1889Arg	2.95
TET2	mutated	c.5666C>G	p.Pro1889Arg	4.94
TET2	mutated	c.5672G>C	p.Arg1891Thr	4.85
TET2	mutated	c.5673G>C	p.Arg1891Ser	2.52
TET2	mutated	c.5675del	p.Asn1892Ilefs*16	6.30

TET2	mutated	c.5678_5686del	p.His1893_Thr1895del	3.80
TET2	mutated	c.5681C>G	p.Pro1894Arg	2.21
TET2	mutated	c.5681C>G	p.Pro1894Arg	5.20
TET2	mutated	c.5688G>T	p.Arg1896Ser	6.40
TET2	mutated	c.5690T>A	p.Ile1897Asn	20.90
TET2	mutated	c.5690T>G	p.Ile1897Ser	5.40
TET2	mutated	c.5692T>C	p.Ser1898Pro	2.44
TET2	mutated	c.5700_5701dup	p.Phe1901Serfs*8	19.50
TET2	mutated	c.5707C>T	p.Gln1903*	2.17
TET2	mutated	c.5707C>T	p.Gln1903*	4.49
TET2	mutated	c.5711A>C	p.His1904Pro	4.55
TET2	mutated	c.5711A>G	p.His1904Arg	2.01
TET2	mutated	c.5711A>G	p.His1904Arg	3.04
TET2	mutated	c.5711A>G	p.His1904Arg	3.41
TET2	mutated	c.5711A>G	p.His1904Arg	3.49
TET2	mutated	c.5711A>G	p.His1904Arg	5.70
TET2	mutated	c.5720T>A	p.Met1907Lys	3.89
TET2	mutated	c.5734C>A	p.His1912Asn	3.06
TET2	mutated	c.5735A>G	p.His1912Arg	7.70
TET2	mutated	c.5745del	p.Leu1916Phefs*34	3.96
TET2	mutated	c.5750G>A	p.Trp1917*	4.24
TET2	mutated	c.5750G>A	p.Trp1917*	7.40
TET2	mutated	c.5758A>G	p.Lys1920Glu	3.41
TET2	mutated	c.5776C>T	p.Arg1926Cys	2.97
TET2	mutated	c.5776C>T	p.Arg1926Cys	28.50
TET2	mutated	c.5776del	p.Arg1926Valfs*24	39.50
TET2	mutated	c.5801A>C	p.Lys1934Thr	2.33
TET2	mutated	c.582_583del	p.Asp194Glufs*7	2.12
TET2	mutated	c.5828A>T	p.Lys1943Ile	2.29
TET2	mutated	c.5876C>G	p.Thr1959Ser	42.00
TET2	mutated	c.5908_5921del	p.Ser1970Aspfs*40	4.42
TET2	mutated	c.5955_5962del	p.Thr1986Ilefs*26	2.96
TET2	mutated	c.5973C>G	p.Phe1991Leu	2.54
TET2	mutated	c.625_626dup	p.Asn209Lysfs*42	6.70
TET2	mutated	c.629del	p.Gly210Valfs*40	2.52
TET2	mutated	c.640del	p.Ser214Leufs*36	27.60
TET2	mutated	c.646del	p.Ser216Leufs*34	2.64
TET2	mutated	c.651del	p.Val218Trpfs*32	2.33
TET2	mutated	c.651del	p.Val218Trpfs*32	20.50
TET2	mutated	c.668G>A	p.Gly223Asp	2.07
TET2	mutated	c.676del	p.Leu226Trpfs*24	7.00
TET2	mutated	c.685dup	p.Thr229Asnfs*25	2.53
TET2	mutated	c.685dup	p.Thr229Asnfs*25	2.60
TET2	mutated	c.685dup	p.Thr229Asnfs*25	2.87
TET2	mutated	c.685dup	p.Thr229Asnfs*25	4.74
TET2	mutated	c.685dup	p.Thr229Asnfs*25	8.10
TET2	mutated	c.715dup	p.Ser239Phefs*15	22.60

TET2	mutated	c.727C>T	p.Gln243*	3.73
TET2	mutated	c.744C>A	p.His248Gln	45.40
TET2	mutated	c.750del	p.Asn250Lysfs*43	12.40
TET2	mutated	c.759del	p.Asn253Lysfs*40	3.56
TET2	mutated	c.761_797dup	p.His266Glnfs*5	3.03
TET2	mutated	c.762_765del	p.Ser254Argfs*38	2.24
TET2	mutated	c.795_798dup	p.Pro267Serfs*16	2.26
TET2	mutated	c.800del	p.Pro267Hisfs*26	3.72
TET2	mutated	c.803C>A	p.Ser268*	25.20
TET2	mutated	c.82_83del	p.Glu28Thrfs*14	7.30
TET2	mutated	c.822del	p.Asn275Ilefs*18	2.82
TET2	mutated	c.822del	p.Asn275Ilefs*18	3.71
TET2	mutated	c.822del	p.Asn275Ilefs*18	3.77
TET2	mutated	c.822del	p.Asn275Ilefs*18	4.39
TET2	mutated	c.822del	p.Asn275Ilefs*18	5.70
TET2	mutated	c.822del	p.Asn275Ilefs*18	6.10
TET2	mutated	c.822del	p.Asn275Ilefs*18	8.30
TET2	mutated	c.832C>T	p.Gln278*	2.55
TET2	mutated	c.832C>T	p.Gln278*	5.00
TET2	mutated	c.840dup	p.Asn281*	2.52
TET2	mutated	c.846dup	p.Glu283*	6.20
TET2	mutated	c.903del	p.Asp302Ilefs*6	3.95
TET2	mutated	c.918dup	p.Leu307Thrfs*24	8.20
TET2	mutated	c.931del	p.Leu311*	23.60
TET2	mutated	c.932_933del	p.Leu311Glnfs*19	6.50
TET2	mutated	c.943del	p.Ser315Profs*32	2.29
TET2	mutated	c.945del	p.Gln317Argfs*30	2.02
TET2	mutated	c.945del	p.Gln317Argfs*30	2.23
TET2	mutated	c.945del	p.Gln317Argfs*30	6.00
TET2	mutated	c.950del	p.Gln317Argfs*30	12.40
TET2	mutated	c.951G>C	p.Gln317His	44.00
TET2	mutated	c.961C>T	p.Gln321*	29.70
TET2	mutated	c.970C>T	p.Gln324*	5.50
TET2	mutated	c.970C>T	p.Gln324*	40.00
TET2	mutated	c.973C>T	p.Gln325*	3.29
TET2	mutated	c.978del	p.Lys326Asnfs*21	4.34
TET2	mutated	c.987del	p.Phe329Leufs*18	2.59
TET2	mutated	c.990_993del	p.Glu330Aspfs*16	2.72
TET2	mutated	c.992del	p.Ile331Asnfs*16	4.54
TP53	mutated	c.1010G>T	p.Arg337Leu	4.34
TP53	mutated	c.102dup	p.Pro36Alafs*7	3.44
TP53	mutated	c.267_269delinsTT	p.Trp91Glyfs*32	9.00
TP53	mutated	c.267del	p.Ser90Profs*33	3.80
TP53	mutated	c.293C>T	p.Pro98Leu	42.70
TP53	mutated	c.314del	p.Gly105Alafs*18	2.88
TP53	mutated	c.358A>G	p.Lys120Glu	2.26
TP53	mutated	c.374C>G	p.Thr125Arg	2.33

TP53	mutated	c.375G>T	p.Thr125Thr	18.30
TP53	mutated	c.377A>G	p.Tyr126Cys	3.45
TP53	mutated	c.377A>G	p.Tyr126Cys	5.90
TP53	mutated	c.395A>G	p.Lys132Arg	2.83
TP53	mutated	c.395A>G	p.Lys132Arg	3.86
TP53	mutated	c.396dup	p.Met133Aspfs*16	12.00
TP53	mutated	c.400T>C	p.Phe134Leu	16.40
TP53	mutated	c.403T>C	p.Cys135Arg	5.20
TP53	mutated	c.404G>A	p.Cys135Tyr	15.60
TP53	mutated	c.418_429del	p.Thr140_Val143del	5.10
TP53	mutated	c.422G>A	p.Cys141Tyr	24.20
TP53	mutated	c.434T>C	p.Leu145Pro	3.16
TP53	mutated	c.451C>A	p.Pro151Thr	4.69
TP53	mutated	c.455C>T	p.Pro152Leu	33.30
TP53	mutated	c.460G>A	p.Gly154Ser	46.70
TP53	mutated	c.469G>T	p.Val157Phe	2.27
TP53	mutated	c.473G>A	p.Arg158His	5.10
TP53	mutated	c.480G>A	p.Met160Ile	43.00
TP53	mutated	c.488A>G	p.Tyr163Cys	2.20
TP53	mutated	c.488A>G	p.Tyr163Cys	2.25
TP53	mutated	c.488A>G	p.Tyr163Cys	3.81
TP53	mutated	c.490A>G	p.Lys164Glu	5.20
TP53	mutated	c.490A>G	p.Lys164Glu	13.70
TP53	mutated	c.517G>A	p.Val173Met	3.49
TP53	mutated	c.517G>T	p.Val173Leu	9.30
TP53	mutated	c.517G>T	p.Val173Leu	18.50
TP53	mutated	c.524G>A	p.Arg175His	2.10
TP53	mutated	c.524G>A	p.Arg175His	2.33
TP53	mutated	c.524G>A	p.Arg175His	3.03
TP53	mutated	c.524G>A	p.Arg175His	3.40
TP53	mutated	c.524G>A	p.Arg175His	6.30
TP53	mutated	c.524G>A	p.Arg175His	6.90
TP53	mutated	c.524G>A	p.Arg175His	19.80
TP53	mutated	c.524G>A	p.Arg175His	24.70
TP53	mutated	c.526T>C	p.Cys176Arg	6.20
TP53	mutated	c.527G>A	p.Cys176Tyr	3.59
TP53	mutated	c.529_546del	p.Pro177_Cys182del	24.00
TP53	mutated	c.530C>T	p.Pro177Leu	2.16
TP53	mutated	c.536A>C	p.His179Pro	6.00
TP53	mutated	c.578A>G	p.His193Arg	2.34
TP53	mutated	c.578A>G	p.His193Arg	11.00
TP53	mutated	c.584T>C	p.Ile195Thr	2.43
TP53	mutated	c.587G>C	p.Arg196Pro	4.63
TP53	mutated	c.627_644del	p.Arg209_His214del	21.00
TP53	mutated	c.634T>A	p.Phe212Ile	5.40
TP53	mutated	c.637C>T	p.Arg213*	6.10
TP53	mutated	c.637C>T	p.Arg213*	7.10

TP53	mutated	c.639del	p.His214Ilefs*33	3.56
TP53	mutated	c.641A>G	p.His214Arg	4.89
TP53	mutated	c.643A>G	p.Ser215Gly	2.65
TP53	mutated	c.646G>A	p.Val216Met	2.82
TP53	mutated	c.646G>A	p.Val216Met	3.44
TP53	mutated	c.646G>A	p.Val216Met	6.70
TP53	mutated	c.650T>G	p.Val217Gly	2.83
TP53	mutated	c.653T>A	p.Val218Glu	3.22
TP53	mutated	c.657_658insATATGCC	p.Tyr220Ilefs*4	23.10
TP53	mutated	c.659A>C	p.Tyr220Ser	3.19
TP53	mutated	c.659A>G	p.Tyr220Cys	2.31
TP53	mutated	c.659A>G	p.Tyr220Cys	2.69
TP53	mutated	c.659A>G	p.Tyr220Cys	3.29
TP53	mutated	c.659A>G	p.Tyr220Cys	3.45
TP53	mutated	c.659A>G	p.Tyr220Cys	5.90
TP53	mutated	c.659A>G	p.Tyr220Cys	47.90
TP53	mutated	c.695T>C	p.Ile232Thr	3.30
TP53	mutated	c.709A>G	p.Met237Val	3.68
TP53	mutated	c.711G>A	p.Met237Ile	2.23
TP53	mutated	c.711G>A	p.Met237Ile	2.33
TP53	mutated	c.712_729delTGTAACAGTTCCTG CATG	p.Cys238_Met243del	3.52
TP53	mutated	c.713G>A	p.Cys238Tyr	2.16
TP53	mutated	c.713G>A	p.Cys238Tyr	5.10
TP53	mutated	c.713G>A	p.Cys238Tyr	10.50
TP53	mutated	c.713G>A	p.Cys238Tyr	15.20
TP53	mutated	c.713G>A	p.Cys238Tyr	35.70
TP53	mutated	c.718A>G	p.Ser240Gly	2.29
TP53	mutated	c.718A>G	p.Ser240Gly	2.88
TP53	mutated	c.722C>T	p.Ser241Phe	2.64
TP53	mutated	c.725G>T	p.Cys242Phe	2.47
TP53	mutated	c.728T>C	p.Met243Thr	7.80
TP53	mutated	c.733G>A	p.Gly245Ser	2.77
TP53	mutated	c.733G>A	p.Gly245Ser	2.83
TP53	mutated	c.734G>A	p.Gly245Asp	4.88
TP53	mutated	c.734G>A	p.Gly245Asp	5.10
TP53	mutated	c.734G>T	p.Gly245Val	5.20
TP53	mutated	c.736A>C	p.Met246Leu	3.65
TP53	mutated	c.736A>G	p.Met246Val	2.18
TP53	mutated	c.736A>G	p.Met246Val	2.80
TP53	mutated	c.737T>A	p.Met246Lys	2.48
TP53	mutated	c.740_742delinsCCT	p.Asn247_Arg248delinsThrTrp	4.20
TP53	mutated	c.742C>T	p.Arg248Trp	2.59
TP53	mutated	c.742C>T	p.Arg248Trp	2.92
TP53	mutated	c.742C>T	p.Arg248Trp	3.48
TP53	mutated	c.742C>T	p.Arg248Trp	5.30
TP53	mutated	c.742C>T	p.Arg248Trp	18.40

TP53	mutated	c.743G>A	p.Arg248Gln	3.23
TP53	mutated	c.743G>A	p.Arg248Gln	5.10
TP53	mutated	c.743G>A	p.Arg248Gln	23.40
TP53	mutated	c.747G>T	p.Arg249Ser	6.20
TP53	mutated	c.749_750del	p.Pro250Hisfs*13	3.82
TP53	mutated	c.749C>T	p.Pro250Leu	2.70
TP53	mutated	c.752T>G	p.Ile251Ser	18.70
TP53	mutated	c.763A>C	p.Ile255Leu	4.25
TP53	mutated	c.783-2A>C	p.splice site mutation	5.30
TP53	mutated	c.794T>C	p.Leu265Pro	15.60
TP53	mutated	c.796G>A	p.Gly266Arg	2.57
TP53	mutated	c.808T>C	p.Phe270Leu	6.10
TP53	mutated	c.808T>G	p.Phe270Val	8.80
TP53	mutated	c.814G>A	p.Val272Met	6.30
TP53	mutated	c.817C>T	p.Arg273Cys	2.19
TP53	mutated	c.817C>T	p.Arg273Cys	4.61
TP53	mutated	c.817C>T	p.Arg273Cys	5.30
TP53	mutated	c.817C>T	p.Arg273Cys	11.10
TP53	mutated	c.818G>A	p.Arg273His	2.24
TP53	mutated	c.818G>A	p.Arg273His	2.67
TP53	mutated	c.818G>A	p.Arg273His	2.78
TP53	mutated	c.818G>A	p.Arg273His	3.27
TP53	mutated	c.818G>A	p.Arg273His	6.40
TP53	mutated	c.818G>A	p.Arg273His	21.20
TP53	mutated	c.821T>G	p.Val274Gly	2.86
TP53	mutated	c.824G>A	p.Cys275Tyr	5.60
TP53	mutated	c.833C>G	p.Pro278Arg	15.80
TP53	mutated	c.838A>G	p.Arg280Gly	6.10
TP53	mutated	c.840A>C	p.Arg280Ser	5.60
TP53	mutated	c.842A>G	p.Asp281Gly	3.68
TP53	mutated	c.844C>T	p.Arg282Trp	2.23
TP53	mutated	c.844C>T	p.Arg282Trp	2.56
TP53	mutated	c.844C>T	p.Arg282Trp	3.27
TP53	mutated	c.845G>T	p.Arg282Leu	43.60
TP53	mutated	c.848G>C	p.Arg283Pro	2.44
TP53	mutated	c.854A>G	p.Glu285Gly	3.78
TP53	mutated	c.856G>A	p.Glu286Lys	4.40
TP53	mutated	c.949del	p.Gln317Serfs*28	4.29
TP53	mutated	c.958A>T	p.Lys320*	4.74
TP53	mutated	c.979del	p.Tyr327Ilefs*18	20.90
U2AF1	mutated	c.100T>G	p.Ser34Ala	2.46
U2AF1	mutated	c.100T>G	p.Ser34Ala	2.62
U2AF1	mutated	c.101C>A	p.Ser34Tyr	3.97
U2AF1	mutated	c.101C>A	p.Ser34Tyr	21.80
U2AF1	mutated	c.101C>T	p.Ser34Phe	2.61
U2AF1	mutated	c.101C>T	p.Ser34Phe	3.16
U2AF1	mutated	c.101C>T	p.Ser34Phe	5.40

U2AF1	mutated	c.101C>T	p.Ser34Phe	6.80
U2AF1	mutated	c.101C>T	p.Ser34Phe	10.80
U2AF1	mutated	c.101C>T	p.Ser34Phe	11.00
U2AF1	mutated	c.467G>A	p.Arg156His	3.12
U2AF1	mutated	c.467G>A	p.Arg156His	3.44
U2AF1	mutated	c.467G>A	p.Arg156His	6.30
U2AF1	mutated	c.470A>C	p.Gln157Pro	2.24
U2AF1	mutated	c.470A>C	p.Gln157Pro	2.38
U2AF1	mutated	c.470A>C	p.Gln157Pro	2.38
U2AF1	mutated	c.470A>C	p.Gln157Pro	2.40
U2AF1	mutated	c.470A>C	p.Gln157Pro	2.75
U2AF1	mutated	c.470A>C	p.Gln157Pro	2.95
U2AF1	mutated	c.470A>C	p.Gln157Pro	3.78
U2AF1	mutated	c.470A>C	p.Gln157Pro	4.07
U2AF1	mutated	c.470A>C	p.Gln157Pro	4.24
U2AF1	mutated	c.470A>C	p.Gln157Pro	5.50
U2AF1	mutated	c.470A>C	p.Gln157Pro	5.50
U2AF1	mutated	c.470A>C	p.Gln157Pro	5.70
U2AF1	mutated	c.470A>C	p.Gln157Pro	6.30
U2AF1	mutated	c.470A>C	p.Gln157Pro	7.80
U2AF1	mutated	c.470A>C	p.Gln157Pro	10.00
U2AF1	mutated	c.470A>C	p.Gln157Pro	10.20
U2AF1	mutated	c.470A>C	p.Gln157Pro	14.60
U2AF1	mutated	c.470A>C	p.Gln157Pro	17.50
U2AF1	mutated	c.470A>G	p.Gln157Arg	3.21
U2AF1	mutated	c.470A>G	p.Gln157Arg	3.31
U2AF1	mutated	c.470A>G	p.Gln157Arg	4.00
U2AF1	mutated	c.470A>G	p.Gln157Arg	5.70
U2AF1	mutated	c.470A>G	p.Gln157Arg	6.10
U2AF1	mutated	c.470A>G	p.Gln157Arg	6.10
U2AF1	mutated	c.470A>G	p.Gln157Arg	6.20
U2AF1	mutated	c.470A>G	p.Gln157Arg	11.50
U2AF1	mutated	c.470A>G	p.Gln157Arg	12.90
U2AF1	mutated	c.470A>G	p.Gln157Arg	14.00
U2AF1	mutated	c.470A>G	p.Gln157Arg	21.00
U2AF1	mutated	c.470A>G	p.Gln157Arg	22.00
U2AF1	mutated	c.470A>G	p.Gln157Arg	32.80
U2AF1	mutated	c.472_477dup	p.Tyr158_Glu159dup	4.11
U2AF1	mutated	c.472_477dup	p.Tyr158_Glu159dup	5.40
U2AF1	mutated	c.476_481dup	p.Glu159_Met160dup	18.50
ZRSR2	mutated	c.1030A>T	p.Arg344*	37.80
ZRSR2	mutated	c.1044del	p.Leu348Phefs*?	2.04
ZRSR2	mutated	c.1120delinsAC	p.Tyr374Thrfs*11	8.00
ZRSR2	mutated	c.1122C>G	p.Tyr374*	21.60
ZRSR2	mutated	c.1167C>G	p.Tyr389*	4.45
ZRSR2	mutated	c.1207del	p.Arg403Glyfs*?	2.85
ZRSR2	mutated	c.1212del	p.Gly404Glyfs*?	5.60

ZRSR2	mutated	c.1291_1302del	p.Asp432_Arg435del	2.47
ZRSR2	mutated	c.1361G>C	p.Arg454Pro	2.54
ZRSR2	mutated	c.1434del	p.Ser478Serfs*?	4.20
ZRSR2	mutated	c.283del	p.Ala95Argfs*13	11.10
ZRSR2	mutated	c.283G>C	p.Ala95Pro	6.70
ZRSR2	mutated	c.294dup	p.Arg99Thrfs*46	10.70
ZRSR2	mutated	c.31G>T	p.Glu11*	2.01
ZRSR2	mutated	c.328C>T	p.Gln110*	8.20
ZRSR2	mutated	c.358_359del	p.Glu120Argfs*24	24.70
ZRSR2	mutated	c.376C>T	p.Arg126*	6.00
ZRSR2	mutated	c.46A>T	p.Lys16*	3.10
ZRSR2	mutated	c.485del	p.Phe162Serfs*3	6.70
ZRSR2	mutated	c.515G>A	p.Cys172Tyr	2.83
ZRSR2	mutated	c.515G>A	p.Cys172Tyr	6.70
ZRSR2	mutated	c.515G>T	p.Cys172Phe	2.01
ZRSR2	mutated	c.559T>A	p.Cys187Ser	2.78
ZRSR2	mutated	c.572A>C	p.His191Pro	7.00
ZRSR2	mutated	c.572A>G	p.His191Arg	2.70
ZRSR2	mutated	c.591T>G	p.Ser197Arg	3.74
ZRSR2	mutated	c.598del	p.Leu200Phefs*38	45.90
ZRSR2	mutated	c.703C>T	p.Gln235*	6.40
ZRSR2	mutated	c.73G>T	p.Glu25*	5.60
ZRSR2	mutated	c.752G>A	p.Gly251Glu	2.33
ZRSR2	mutated	c.752G>A	p.Gly251Glu	3.44
ZRSR2	mutated	c.758T>G	p.Val253Gly	4.39
ZRSR2	mutated	c.794A>G	p.His265Arg	3.41
ZRSR2	mutated	c.79C>G	p.Arg27Gly	6.90
ZRSR2	mutated	c.803G>A	p.Gly268Asp	6.30
ZRSR2	mutated	c.803G>A	p.Gly268Asp	8.70
ZRSR2	mutated	c.811T>A	p.Tyr271Asn	5.80
ZRSR2	mutated	c.817C>T	p.Gln273*	4.64
ZRSR2	mutated	c.838T>C	p.Cys280Arg	2.48
ZRSR2	mutated	c.840C>G	p.Cys280Trp	3.77
ZRSR2	mutated	c.847G>A	p.Ala283Thr	13.30
ZRSR2	mutated	c.896G>A	p.Cys299Tyr	2.46
ZRSR2	mutated	c.916del	p.Arg306Glyfs*?	2.20
ZRSR2	mutated	c.920G>A	p.Trp307*	4.43
ZRSR2	mutated	c.938G>T	p.Gly313Val	3.66
ZRSR2	mutated	c.979A>G	p.Asn327Asp	8.70

Supplemental Table 2 – List of 329 unique CHIP mutations identified in MISSION based on deep-DNA-sequencing. Provided are gene name, confirmation of CHIP mutation, change on DNA level, change on amino acid (AA) level and variant allele frequency (VAF). Polymorphisms, variants, synonymous and uncertain mutations were excluded.

Gene	CHIP	DNA	AA	VAF
ASXL1	mutated	c.1534C>T	p.Gln512*	4.10
ASXL1	mutated	c.1564C>T	p.Gln522*	4.70
ASXL1	mutated	c.1585C>T	p.Gln529*	16.30
ASXL1	mutated	c.1720-2A>G	p.splice site mutation	6.30
ASXL1	mutated	c.1749G>A	p.Trp583*	31.50
ASXL1	mutated	c.1762C>T	p.Gln588*	5.50
ASXL1	mutated	c.1772dup	p.Tyr591*	3.00
ASXL1	mutated	c.1772dup	p.Tyr591*	5.20
ASXL1	mutated	c.1900_1922del	p.Glu635Argfs*15	6.60
ASXL1	mutated	c.1900_1922del	p.Glu635Argfs*15	7.60
ASXL1	mutated	c.1900_1922del	p.Glu635Argfs*15	11.00
ASXL1	mutated	c.1900_1922del	p.Glu635Argfs*15	19.60
ASXL1	mutated	c.1934dup	p.Gly646Trpfs*12	7.00
ASXL1	mutated	c.1934dup	p.Gly646Trpfs*12	10.80
ASXL1	mutated	c.1934dup	p.Gly646Trpfs*12	11.20
ASXL1	mutated	c.1934dup	p.Gly646Trpfs*12	12.40
ASXL1	mutated	c.1934dup	p.Gly646Trpfs*12	16.00
ASXL1	mutated	c.1934dup	p.Gly646Trpfs*12	16.60
ASXL1	mutated	c.1934dup	p.Gly646Trpfs*12	18.60
ASXL1	mutated	c.2077C>T	p.Arg693*	2.10
ASXL1	mutated	c.2083C>T	p.Gln695*	2.60
ASXL1	mutated	c.2290del	p.Leu764Tyrfs*8	5.50
ASXL1	mutated	c.2302C>T	p.Gln768*	22.60
ASXL1	mutated	c.2324T>G	p.Leu775*	14.10
ASXL1	mutated	c.2387G>A	p.Trp796*	16.30
ASXL1	mutated	c.2468del	p.Leu823*	6.10
ASXL1	mutated	c.2528_2529insCT	p.Thr844*	2.20
ASXL1	mutated	c.2676del	p.Asn893Thrfs*15	9.60
ASXL1	mutated	c.2989G>T	p.Glu997*	7.80
ASXL1	mutated	c.3554dup	p.Thr1186Hisfs*7	2.70
BCOR	mutated	c.4616dup	p.Asn1540Glufs*16	7.00
CALR	mutated	c.1154_1155insTTGTC	p.Lys385Asnfs*47	28.80
CBL	mutated	c.1009del	p.Tyr337Ilefs*13	5.00
CBL	mutated	c.1102T>C	p.Tyr368His	11.50
CBL	mutated	c.1129A>G	p.Thr377Ala	8.00
CBL	mutated	c.1145A>G	p.Lys382Arg	4.30
CBL	mutated	c.1211G>A	p.Cys404Tyr	3.00
CBL	mutated	c.1211G>A	p.Cys404Tyr	4.20
CBL	mutated	c.1211G>A	p.Cys404Tyr	9.10
CBL	mutated	c.1211G>A	p.Cys404Tyr	29.20
CBL	mutated	c.1254C>A	p.Phe418Leu	6.70
CBL	mutated	c.1268T>A	p.Ile423Asn	23.00

CBL	mutated	c.1694del	p.Leu565Cysfs*50	3.50
DNMT3A	mutated	c.1014+1G>T	p.splice site mutation	4.00
DNMT3A	mutated	c.1040T>C	p.Leu347Pro	9.60
DNMT3A	mutated	c.1058_1066del	p.Ala353_Gln356delinsGlu	11.00
DNMT3A	mutated	c.1077_1078dup	p.Asn360Thrfs*48	2.10
DNMT3A	mutated	c.1136G>A	p.Arg379His	3.30
DNMT3A	mutated	c.1152_1155del	p.Phe384Leufs*22	8.00
DNMT3A	mutated	c.1156del	p.Val386Cysfs*21	3.00
DNMT3A	mutated	c.1223_1226del	p.Glu408Glyfs*242	5.10
DNMT3A	mutated	c.1229C>T	p.Ala410Val	5.50
DNMT3A	mutated	c.1238dup	p.Phe414Leufs*7	2.70
DNMT3A	mutated	c.1429G>C	p.Glu477Gln	5.50
DNMT3A	mutated	c.1481G>A	p.Cys494Tyr	2.90
DNMT3A	mutated	c.1489T>C	p.Cys497Arg	2.20
DNMT3A	mutated	c.1498del	p.Leu500Serfs*151	2.30
DNMT3A	mutated	c.1507dup	p.Thr503Asnfs*43	14.20
DNMT3A	mutated	c.1517A>G	p.His506Arg	2.60
DNMT3A	mutated	c.1543C>T	p.Gln515*	19.40
DNMT3A	mutated	c.1555-2A>T	p.splice site mutation	4.80
DNMT3A	mutated	c.1592A>G	p.Asp531Gly	6.10
DNMT3A	mutated	c.1628G>C	p.Gly543Ala	21.60
DNMT3A	mutated	c.1637T>A	p.Val546Glu	3.80
DNMT3A	mutated	c.1640T>A	p.Leu547His	4.10
DNMT3A	mutated	c.1657_1659del	p.Asn553del	8.90
DNMT3A	mutated	c.1726_1729delinsC	p.Ile576_Lys577delinsGln	22.60
DNMT3A	mutated	c.1903C>T	p.Arg635Trp	2.80
DNMT3A	mutated	c.1969G>A	p.Val657Met	5.30
DNMT3A	mutated	c.1972G>T	p.Asp658Tyr	10.00
DNMT3A	mutated	c.1979A>G	p.Tyr660Cys	4.50
DNMT3A	mutated	c.1998_1999del	p.Cys666*	2.20
DNMT3A	mutated	c.1998T>G	p.Cys666Trp	2.90
DNMT3A	mutated	c.2007dup	p.Ile670Hisfs*43	20.60
DNMT3A	mutated	c.2024_2026dup	p.Val675_Arg676insLeu	4.70
DNMT3A	mutated	c.2053G>C	p.Gly685Arg	15.00
DNMT3A	mutated	c.2057A>G	p.Asp686Gly	2.30
DNMT3A	mutated	c.2063G>A	p.Arg688His	2.30
DNMT3A	mutated	c.2084T>C	p.Ile695Thr	2.00
DNMT3A	mutated	c.2095G>C	p.Gly699Arg	2.30
DNMT3A	mutated	c.2095G>C	p.Gly699Arg	16.70
DNMT3A	mutated	c.2098C>A	p.Pro700Thr	4.70
DNMT3A	mutated	c.2099C>T	p.Pro700Leu	2.40
DNMT3A	mutated	c.2104G>T	p.Asp702Tyr	11.80
DNMT3A	mutated	c.2171A>G	p.Tyr724Cys	2.20
DNMT3A	mutated	c.2171A>G	p.Tyr724Cys	5.70
DNMT3A	mutated	c.2183G>A	p.Gly728Asp	5.60
DNMT3A	mutated	c.2185C>T	p.Arg729Trp	13.90
DNMT3A	mutated	c.2185C>T	p.Arg729Trp	30.80
DNMT3A	mutated	c.2192T>A	p.Phe731Tyr	5.00
DNMT3A	mutated	c.2204A>C	p.Tyr735Ser	8.50

DNMT3A	mutated	c.2204A>G	p.Tyr735Cys	11.30
DNMT3A	mutated	c.2204A>G	p.Tyr735Cys	22.80
DNMT3A	mutated	c.2206C>T	p.Arg736Cys	4.80
DNMT3A	mutated	c.2206C>T	p.Arg736Cys	9.50
DNMT3A	mutated	c.2206C>T	p.Arg736Cys	9.60
DNMT3A	mutated	c.2228C>T	p.Pro743Leu	12.20
DNMT3A	mutated	c.2245C>T	p.Arg749Cys	4.40
DNMT3A	mutated	c.2245C>T	p.Arg749Cys	5.60
DNMT3A	mutated	c.2245C>T	p.Arg749Cys	6.30
DNMT3A	mutated	c.2259G>A	p.Trp753*	5.20
DNMT3A	mutated	c.2264T>C	p.Phe755Ser	4.30
DNMT3A	mutated	c.2302delG	p.Asp768Thrfs*11	2.30
DNMT3A	mutated	c.2309C>T	p.Ser770Leu	2.90
DNMT3A	mutated	c.2311C>G	p.Arg771Gly	3.60
DNMT3A	mutated	c.2311C>T	p.Arg771*	4.90
DNMT3A	mutated	c.2311C>T	p.Arg771*	15.30
DNMT3A	mutated	c.2312G>T	p.Arg771Leu	23.70
DNMT3A	mutated	c.2320G>T	p.Glu774*	11.30
DNMT3A	mutated	c.2333T>G	p.Val778Gly	2.80
DNMT3A	mutated	c.2333T>G	p.Val778Gly	6.50
DNMT3A	mutated	c.2339T>C	p.Ile780Thr	2.20
DNMT3A	mutated	c.2339T>C	p.Ile780Thr	5.40
DNMT3A	mutated	c.2387del	p.Gly796Valfs*6	2.20
DNMT3A	mutated	c.2389A>T	p.Asn797Tyr	2.70
DNMT3A	mutated	c.2390A>G	p.Asn797Ser	17.00
DNMT3A	mutated	c.2395C>T	p.Pro799Ser	5.00
DNMT3A	mutated	c.2404A>T	p.Asn802Tyr	5.10
DNMT3A	mutated	c.2462del	p.His821Leufs*4	3.00
DNMT3A	mutated	c.2524C>T	p.Gln842*	2.40
DNMT3A	mutated	c.2524C>T	p.Gln842*	3.70
DNMT3A	mutated	c.2533del	p.Asp845Thrfs*8	3.00
DNMT3A	mutated	c.2550del	p.Phe851Serfs*2	3.10
DNMT3A	mutated	c.2578T>C	p.Trp860Arg	6.20
DNMT3A	mutated	c.2644C>T	p.Arg882Cys	2.60
DNMT3A	mutated	c.2644C>T	p.Arg882Cys	32.20
DNMT3A	mutated	c.2645G>A	p.Arg882His	2.20
DNMT3A	mutated	c.2645G>A	p.Arg882His	2.50
DNMT3A	mutated	c.2645G>A	p.Arg882His	3.20
DNMT3A	mutated	c.2666T>C	p.Leu889Pro	2.20
DNMT3A	mutated	c.2679G>C	p.Trp893Cys	3.50
DNMT3A	mutated	c.2695C>T	p.Arg899Cys	2.00
DNMT3A	mutated	c.2695C>T	p.Arg899Cys	2.20
DNMT3A	mutated	c.2695C>T	p.Arg899Cys	4.70
DNMT3A	mutated	c.2705del	p.Phe902Serfs*4	8.20
DNMT3A	mutated	c.884T>A	p.Leu295Gln	2.30
DNMT3A	mutated	c.893G>A	p.Gly298Glu	2.60
DNMT3A	mutated	c.901C>T	p.Arg301Trp	2.20
DNMT3A	mutated	c.905G>T	p.Gly302Val	13.90
DNMT3A	mutated	c.914G>A	p.Trp305*	2.50

DNMT3A	mutated	c.915G>A	p.Trp305*	26.40
DNMT3A	mutated	c.929T>C	p.Ile310Thr	3.00
DNMT3A	mutated	c.976C>T	p.Arg326Cys	3.60
DNMT3A	mutated	c.976C>T	p.Arg326Cys	8.40
DNMT3A	mutated	c.976C>T	p.Arg326Cys	20.50
GNAS	mutated	c.2531G>A	p.Arg844His	2.90
GNB1	mutated	c.169A>G	p.Lys57Glu	13.90
IDH1	mutated	c.395G>A	p.Arg132His	43.70
JAK2	mutated	c.1849G>T	p.Val617Phe	2.00
JAK2	mutated	c.1849G>T	p.Val617Phe	2.30
JAK2	mutated	c.1849G>T	p.Val617Phe	2.60
JAK2	mutated	c.1849G>T	p.Val617Phe	4.50
JAK2	mutated	c.1849G>T	p.Val617Phe	5.50
JAK2	mutated	c.1849G>T	p.Val617Phe	38.70
KRAS	mutated	c.35G>A	p.Gly12Asp	4.30
PPM1D	mutated	c.1270G>T	p.Glu424*	5.90
PPM1D	mutated	c.1349del	p.Leu450*	4.40
PPM1D	mutated	c.1372C>T	p.Arg458*	17.40
PPM1D	mutated	c.1382del	p.Val461Alafs*4	2.40
PPM1D	mutated	c.1434C>A	p.Cys478*	8.80
PPM1D	mutated	c.1528C>T	p.Gln510*	2.80
PPM1D	mutated	c.1566dup	p.Ala523Serfs*5	10.00
PPM1D	mutated	c.1609del	p.Thr537Hisfs*2	34.00
PPM1D	mutated	c.1649del	p.His550Leufs*6	6.90
PPM1D	mutated	c.1654C>T	p.Arg552*	2.70
PPM1D	mutated	c.1654C>T	p.Arg552*	13.60
PPM1D	mutated	c.1714C>T	p.Arg572*	2.80
RAD21	mutated	c.144+1G>T	p.splice site mutation	6.10
SF3B1	mutated	c.1996A>C	p.Lys666Gln	4.80
SF3B1	mutated	c.1997A>G	p.Lys666Arg	7.80
SF3B1	mutated	c.1998G>C	p.Lys666Asn	8.10
SF3B1	mutated	c.1998G>T	p.Lys666Asn	5.40
SF3B1	mutated	c.1998G>T	p.Lys666Asn	23.90
SF3B1	mutated	c.1998G>T	p.Lys666Asn	26.70
SF3B1	mutated	c.1998G>T	p.Lys666Asn	27.10
SF3B1	mutated	c.2098A>G	p.Lys700Glu	13.80
SF3B1	mutated	c.2098A>G	p.Lys700Glu	23.00
SMC1A	mutated	c.2131C>T	p.Arg711Trp	4.70
SMC3	mutated	c.3598G>A	p.Val1200Met	6.00
SRSF2	mutated	c.170T>A	p.Phe57Tyr	2.00
SRSF2	mutated	c.170T>A	p.Phe57Tyr	2.10
SRSF2	mutated	c.284C>A	p.Pro95His	23.10
SRSF2	mutated	c.284C>A	p.Pro95His	32.00
SRSF2	mutated	c.284C>G	p.Pro95Arg	18.20
SRSF2	mutated	c.284C>T	p.Pro95Leu	41.80
SRSF2	mutated	c.287C>T	p.Pro96Leu	7.40
TET2	mutated	c.1028_1046del	p.Thr343Metfs*23	2.40
TET2	mutated	c.1061C>A	p.Ser354*	17.50
TET2	mutated	c.1201_1203del	p.Pro401del	2.70

TET2	mutated	c.1201_1203del	p.Pro401del	2.80
TET2	mutated	c.1219del	p.Ser407Leufs*20	27.70
TET2	mutated	c.1249C>T	p.Gln417*	4.40
TET2	mutated	c.1430del	p.Ser477Leufs*9	3.20
TET2	mutated	c.1588C>T	p.Gln530*	5.90
TET2	mutated	c.1630C>T	p.Arg544*	3.80
TET2	mutated	c.1630C>T	p.Arg544*	25.00
TET2	mutated	c.1699_1703del	p.Leu567Glyfs*14	2.70
TET2	mutated	c.1800_1801dup	p.Thr601Argfs*39	6.30
TET2	mutated	c.1803del	p.Ser602Profs*37	16.50
TET2	mutated	c.1842del	p.Leu615Serfs*24	3.00
TET2	mutated	c.1863_1879del	p.Gln622Glyfs*10	3.80
TET2	mutated	c.2255_2261del	p.Asn752Argfs*59	2.10
TET2	mutated	c.2276del	p.Thr759Ilefs*54	5.50
TET2	mutated	c.2370_2382dup	p.Ser795Valfs*11	6.60
TET2	mutated	c.2375C>G	p.Ser792*	17.40
TET2	mutated	c.2662C>T	p.Gln888*	2.60
TET2	mutated	c.2662C>T	p.Gln888*	6.00
TET2	mutated	c.2674C>T	p.Gln892*	7.60
TET2	mutated	c.2746C>T	p.Gln916*	4.60
TET2	mutated	c.2746C>T	p.Gln916*	5.10
TET2	mutated	c.2749C>T	p.Gln917*	27.00
TET2	mutated	c.2839C>T	p.Gln947*	19.20
TET2	mutated	c.2884C>T	p.Gln962*	29.50
TET2	mutated	c.2896C>T	p.Gln966*	2.00
TET2	mutated	c.2926C>T	p.Gln976*	2.00
TET2	mutated	c.2926C>T	p.Gln976*	16.30
TET2	mutated	c.2944A>T	p.Lys982*	38.90
TET2	mutated	c.3119T>G	p.Leu1040*	2.90
TET2	mutated	c.3127del	p.His1043Ilefs*12	21.00
TET2	mutated	c.3287del	p.Thr1096Lysfs*10	2.60
TET2	mutated	c.3369del	p.Val1124Serfs*13	2.00
TET2	mutated	c.3409+1G>A	p.splice site mutation	2.60
TET2	mutated	c.3415del	p.Ile1139Leufs*13	3.10
TET2	mutated	c.3415del	p.Ile1139Leufs*13	6.90
TET2	mutated	c.3522_3523insG	p.Ile1175Aspfs*2	19.00
TET2	mutated	c.3523A>T	p.Ile1175Phe	18.70
TET2	mutated	c.3524_3526delinsCTT	p.Ile1175_Arg1176delinsThrTrp	5.90
TET2	mutated	c.3530T>G	p.Ile1177Ser	7.40
TET2	mutated	c.3578G>A	p.Cys1193Tyr	2.60
TET2	mutated	c.3640C>T	p.Arg1214Trp	4.70
TET2	mutated	c.3732_3733del	p.Tyr1245Leufs*22	4.80
TET2	mutated	c.3734A>G	p.Tyr1245Cys	39.60
TET2	mutated	c.3755T>C	p.Leu1252Pro	4.30
TET2	mutated	c.3782G>A	p.Arg1261His	2.00
TET2	mutated	c.3785G>A	p.Arg1262Gln	2.00
TET2	mutated	c.3822G>C	p.Gln1274His	5.70
TET2	mutated	c.3863G>A	p.Gly1288Asp	2.00
TET2	mutated	c.3863G>A	p.Gly1288Asp	3.30

TET2	mutated	c.3866G>T	p.Cys1289Phe	2.20
TET2	mutated	c.3894dup	p.Lys1299*	13.50
TET2	mutated	c.3904A>G	p.Arg1302Gly	5.70
TET2	mutated	c.3968del	p.Glu1323Glyfs*40	37.80
TET2	mutated	c.4015A>T	p.Lys1339*	23.30
TET2	mutated	c.4030G>A	p.Ala1344Thr	8.00
TET2	mutated	c.4042del	p.Gln1348Argfs*15	7.60
TET2	mutated	c.4075C>A	p.Arg1359Ser	4.70
TET2	mutated	c.4076G>A	p.Arg1359His	2.90
TET2	mutated	c.4081G>C	p.Gly1361Arg	4.90
TET2	mutated	c.4082G>A	p.Gly1361Asp	2.90
TET2	mutated	c.4103_4116del	p.Phe1368Cysfs*28	19.20
TET2	mutated	c.4131_4132del	p.Phe1377Leufs*23	21.50
TET2	mutated	c.4132T>C	p.Cys1378Arg	19.60
TET2	mutated	c.4133G>A	p.Cys1378Tyr	26.30
TET2	mutated	c.4136C>T	p.Ala1379Val	36.60
TET2	mutated	c.4138C>T	p.His1380Tyr	7.60
TET2	mutated	c.4140T>G	p.His1380Gln	5.90
TET2	mutated	c.4193T>G	p.Leu1398Arg	6.40
TET2	mutated	c.4234G>T	p.Asp1412Tyr	19.30
TET2	mutated	c.4256C>G	p.Pro1419Arg	7.70
TET2	mutated	c.4354C>T	p.Arg1452*	5.00
TET2	mutated	c.4393C>T	p.Arg1465*	2.10
TET2	mutated	c.4393C>T	p.Arg1465*	26.90
TET2	mutated	c.4399del	p.Arg1467Glyfs*3	14.60
TET2	mutated	c.4481C>G	p.Ser1494*	3.60
TET2	mutated	c.4546C>T	p.Arg1516*	3.90
TET2	mutated	c.4546C>T	p.Arg1516*	29.70
TET2	mutated	c.4570C>T	p.Gln1524*	12.30
TET2	mutated	c.4621C>T	p.Gln1541*	18.40
TET2	mutated	c.4624C>T	p.Gln1542*	6.60
TET2	mutated	c.4757C>G	p.Ser1586*	3.00
TET2	mutated	c.4854C>G	p.Tyr1618*	5.00
TET2	mutated	c.4879C>T	p.Gln1627*	2.50
TET2	mutated	c.506_508delinsC	p.His169Profs*6	2.40
TET2	mutated	c.5220dup	p.Pro1741Thrfs*12	2.90
TET2	mutated	c.5271_5272dup	p.Ser1758Phefs*6	28.70
TET2	mutated	c.532G>T	p.Glu178*	29.30
TET2	mutated	c.5413_5420del	p.Asn1805*	3.70
TET2	mutated	c.5454_5458del	p.Leu1819*	9.80
TET2	mutated	c.5467_5472delinsCC	p.Asn1823Profs*9	25.70
TET2	mutated	c.5482C>T	p.Gln1828*	2.10
TET2	mutated	c.5500C>T	p.Gln1834*	4.20
TET2	mutated	c.5541G>A	p.Trp1847*	2.10
TET2	mutated	c.5541G>A	p.Trp1847*	3.10
TET2	mutated	c.5543C>A	p.Ser1848*	4.20
TET2	mutated	c.5551_5554del	p.Glu1851Argfs*35	4.00
TET2	mutated	c.5603A>G	p.His1868Arg	2.10
TET2	mutated	c.5615T>A	p.Leu1872His	3.30

TET2	mutated	c.561dup	p.Lys188Glufs*4	18.90
TET2	mutated	c.5621A>T	p.Glu1874Val	2.10
TET2	mutated	c.5636A>C	p.Glu1879Ala	11.50
TET2	mutated	c.5642A>G	p.His1881Arg	2.50
TET2	mutated	c.5690T>G	p.Ile1897Ser	4.10
TET2	mutated	c.5720T>A	p.Met1907Lys	11.10
TET2	mutated	c.661_667del	p.Thr221Valfs*27	5.80
TET2	mutated	c.662_663insTC	p.Gly223Metfs*28	2.20
TET2	mutated	c.763C>T	p.Gln255*	6.50
TET2	mutated	c.822del	p.Asn275Ilefs*18	2.00
TET2	mutated	c.840_841insTT	p.Asn281Leufs*13	4.10
TET2	mutated	c.897dup	p.Asp300*	4.90
TP53	mutated	c.223C>G	p.Pro75Ala	4.70
TP53	mutated	c.329G>C	p.Arg110Pro	5.20
TP53	mutated	c.376T>C	p.Tyr126His	5.90
TP53	mutated	c.464C>T	p.Thr155Ile	3.20
TP53	mutated	c.473G>T	p.Arg158Leu	4.40
TP53	mutated	c.530C>T	p.Pro177Leu	2.50
TP53	mutated	c.533A>C	p.His178Pro	2.90
TP53	mutated	c.541C>T	p.Arg181Cys	45.90
TP53	mutated	c.584T>C	p.Ile195Thr	11.40
TP53	mutated	c.586C>T	p.Arg196*	24.20
TP53	mutated	c.658T>C	p.Tyr220His	2.40
TP53	mutated	c.668C>T	p.Pro223Leu	2.10
TP53	mutated	c.734G>A	p.Gly245Asp	4.60
TP53	mutated	c.745A>G	p.Arg249Gly	2.60
TP53	mutated	c.997del	p.Arg333Valfs*12	2.50
U2AF1	mutated	c.101C>T	p.Ser34Phe	7.00
U2AF1	mutated	c.470A>C	p.Gln157Pro	3.20
U2AF1	mutated	c.470A>C	p.Gln157Pro	9.80
U2AF1	mutated	c.470A>G	p.Gln157Arg	4.60
U2AF1	mutated	c.470A>G	p.Gln157Arg	23.60
U2AF1	mutated	c.470A>G	p.Gln157Arg	37.10
ZRSR2	mutated	c.1017del	p.Trp340Glyfs*?	20.90
ZRSR2	mutated	c.1141dup	p.Arg381Lysfs*4	16.90
ZRSR2	mutated	c.1223dup	p.His408Glnfs*20	5.70
ZRSR2	mutated	c.376C>T	p.Arg126*	17.50
ZRSR2	mutated	c.398_399del	p.Glu133Glyfs*11	8.30
ZRSR2	mutated	c.593del	p.Pro198Leufs*40	4.50
ZRSR2	mutated	c.80G>T	p.Arg27Leu	6.80
ZRSR2	mutated	c.83dup	p.Lys29Glufs*26	4.60
ZRSR2	mutated	c.860_864delinsAAT	p.Phe287*	2.40

Supplemental Table 3 – Patient and histological characteristics of TET2 mutation carriers and matched controls without CHIP mutation from MISSION. Matching between cases and controls was based on age and sex. Plus-minus values are mean ± SD. Absolute numbers are provided as n (%). Statistical testing was performed using the Welch two-sample t-test for continuous variables and Fisher's exact test for categorical variables. BMI: body mass index. PMI: post-mortem interval.

Baseline characteristics	TET2 CHIP (n=26)	no CHIP (n=13)	p-value
Age (years)	79.9 ± 6.3	79.4 ± 7.6	0.8
Female Sex	9 (35)	4	0.8
BMI (kg/m²)	26.3 ± 4.3	26.3 ± 2.9	1.0
Height (cm)	1.67 ± 0.1	1.68 ± 0.1	0.8
Weight (kg)	74.1 ± 15.4	74.1 ± 8.1	1.0
PMI (h)	39.6 ± 15.8	42.2 ± 17.5	0.6

Histological Features	TET2 CHIP (n=26)	no CHIP (n=13)	p-value
Overall plaque phenotypes			0.18
Fibrous	5 (19)	7	
Fibroatheroma	16 (62)	6	
Atheroma	5 (19)	2	
Necrotic core size			0.04
% of plaque size	16 ± 9	11 ± 6	
Leukocytes in the plaque (binned)			0.01
none or minor	7 (27)	9	
moderate or heavy	19 (73)	4	
Macrophages in the plaque (binned)			0.09
none or minor	9 (35)	9	
moderate or heavy	17 (65)	4	
SMC in the plaque (binned)			0.73
none or minor	18 (69)	8	
moderate or heavy	8 (31)	5	
Collagen in the plaque (binned)			0.50
none or minor	16 (62)	6	
moderate or heavy	10 (38)	7	
Elastin integrity (binned)			0.04
Elastin score 0-1	10 (38)	10	
Elastin score 2-3	16 (62)	3	
Fat in the plaque (binned)			0.48
none or minor	17 (65)	10	
moderate or heavy	9 (35)	3	
Calcification in the plaque (binned)			0.03

none or minor	8 (31)	9	
moderate or heavy	18 (69)	4	

Supplemental Table 4 – Patient characteristics of TET2 mutation carriers with CHIP-affected macrophages and matched controls without CHIP mutation from STARNET. Analyses were performed within the matched cohort using linear regression models (for quantitative variables) via limma and Matrix eQTL, and logistic regression models (for categorical variables), adjusted for age, sex, BMI, dyslipidemia, and kidney function. Plus-minus values are mean ± SD. BMI: body mass index.

Baseline characteristics	TET2 CHIP (n=3)	TET2 non-CHIP (=21)	p-value
Age (years)	60.3 ± 12.0	61.8 ± 8.2	0.8
Male sex	3	21	1.0
BMI (kg/m²)	28.0 ± 4.9	28.6 ± 5.0	0.9
Arterial hypertension	2	14 of 20	1.0
Dyslipidemia or statin use	3	20 of 20	1.0
Smoking (ever)	2	7 of 20	0.5
Diabetes	0	4 of 20	1.0
Prior myocardial infarction	1	7 of 20	1.0
Prior stroke	0	0 of 20	1.0

Supplemental Table 5 – Patient characteristics of TET2 mutation carriers and matched controls without CHIP mutation underoing carotid endarteriectomy from AtheroExpress. Analyses were performed within the matched cohort using linear regression models (for quantitative variables) via limma and Matrix eQTL, and logistic regression models (for categorical variables), adjusted for age, sex, BMI, dyslipidemia and kidney function. Plus-minus values are mean ± SD. BMI: body mass index. CAD: coronary artery disease. FU: follow-up. MI: myocardial infarction.

Baseline characteristics	TET2 CHIP (n=30)	TET2 non-CHIP (=90)	p-value
Age (years)	73.1 ± 8.7	72.9 ± 7.2	1.0
Male sex	21 (70)	64 (71.1)	1.0
BMI (kg/m²)	26.6 ± 3.5	26.5 ± 3.5	0.9
Systolic pressure (mmHg)	153.6 ± 26.4	153.0 ± 22.5	1.0
Statin use	23 (77)	72 (80.0)	0.8
Smoking	7 (23)	22 (24.4)	1.0
Diabetes	11 (37)	29 (32.2)	0.7
History of CAD	13 (43)	34 (37.7)	0.7
Prior MI or stroke	7 (23)	10 (11.1)	0.1
CV event during FU	12 (40)	18 (20.0)	0.05

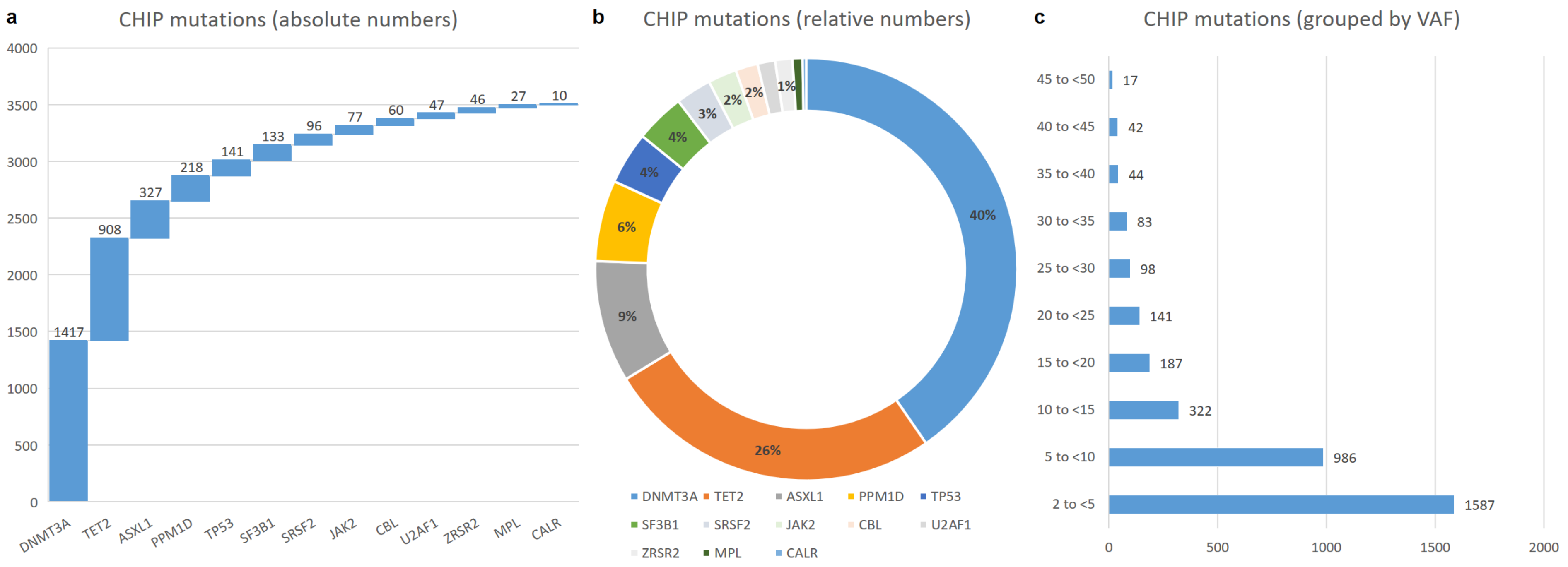

Supplemental Figure 1 – Distribution of 3,507 unique CHIP-associated mutations identified in the clinical cohort from Munich using targeted deep DNA sequencing. **a**, absolute number of CHIP mutations detected per gene. **b**, proportional distribution of CHIP mutations across genes, expressed as relative frequencies. **c**, CHIP mutations grouped by variant allele frequency (VAF).

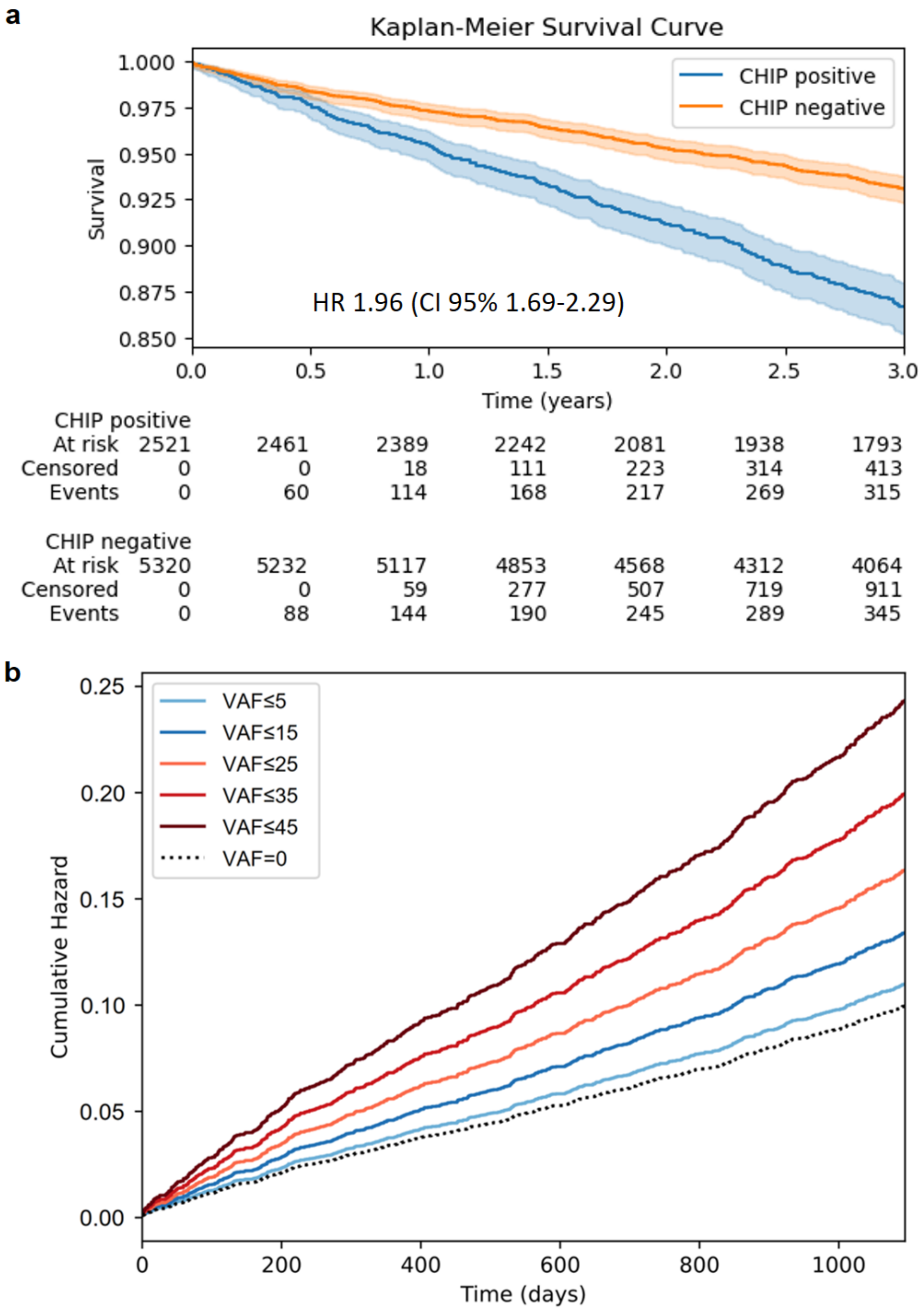

Supplemental Figure 2 – CHIP is associated with increased mortality risk in CAD patients. a, Kaplan-Meier curves show the cumulative survival probability in the overall CHIP CAD vs. no CHIP CAD patients (unmatched) over 3 years for all CHIP patients with a VAF≥2%. The shaded areas represent the 95% confidence interval around the survival estimate. **b**, cumulative hazard for mortality is provided for different VAFs in the propensity matched cohort. CI: confidence interval; HR: Hazard ratio; VAF: Variant allele frequency.

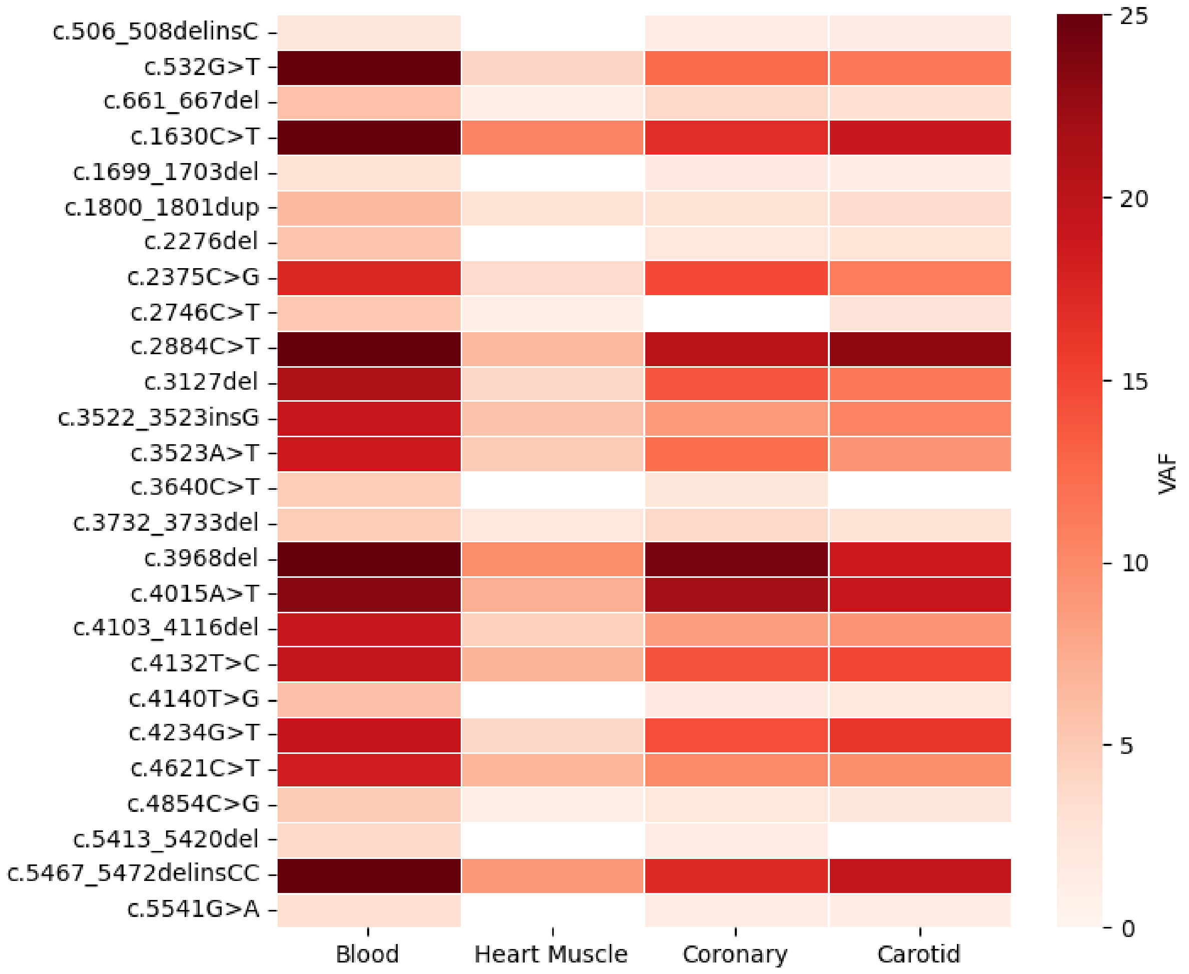

Supplemental Figure 3 – Heat map representation of TET2 CHIP mutations in 26 CAD patients, identified through deep DNA sequencing. To validate these CHIP mutations in cardiovascular-relevant tissues, DNA from left ventricular heart muscle, atherosclerotic coronary, and carotid samples was screened. Variant allele frequency (VAF) is displayed, ranging from 0 (white) to 25 (dark red). Tissues without detectable mutations are shown in white.

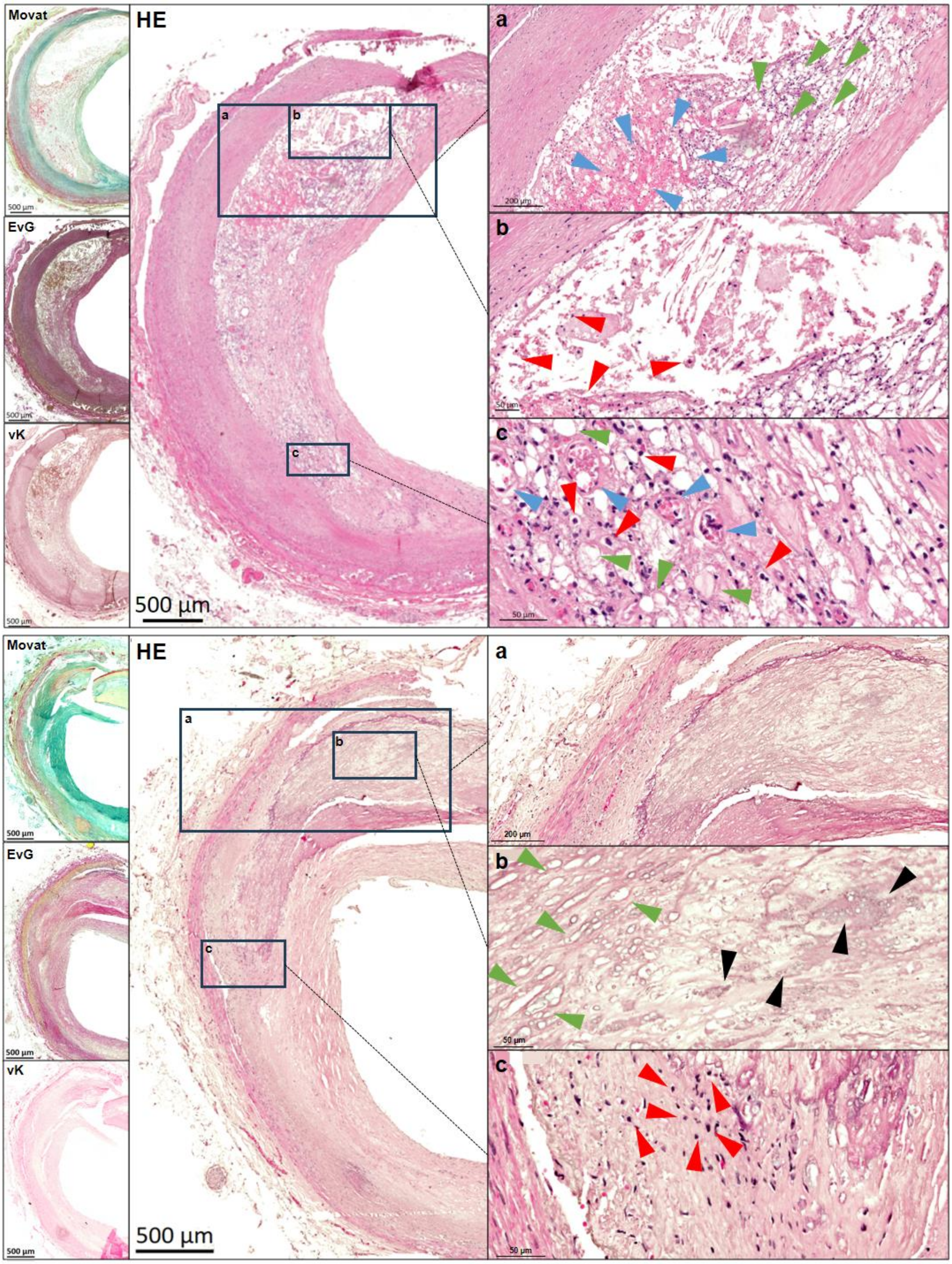

Supplemental Figure 4 – CHIP-positive tissues were morphologically associated with a more severe atherosclerotic phenotype. Human coronary artery plaque of a TET2 CHIP (upper panel) and non-CHIP mutation carrier (lower panel). Provided are Movat pentachrome (Movat), Elastica van Gieson (EvG), von Kossa Silver (vK) and Hematoxylin and Elastin (HE)

stainings. **Upper panel: a**, large necrotic core area with disrupted cholesterol-rich regions, increased lipid pools (green triangles), and intraplaque hemorrhage (blue triangles). **b**, fibrocalcific necrotic core with inflammatory cells (red triangles) and cholesterol deposits. Early calcification is evident in vK stain. **c**, highly inflamed shoulder region with numerous immune cells (red triangles) transforming into foam cells and secreting lipids (green triangles), along with elevated neovascularization (blue triangles). **Lower panel: a**, dense necrotic core with a fibrous area. Early calcification appears dark purple, while the necrotic core area is colored light purple. **b**, Section showing a necrotic core area with cholesterol clefts, lipids (green triangles), and cell debris (black triangles). **c**, light to medium inflamed shoulder region characterized by circular immune cells.

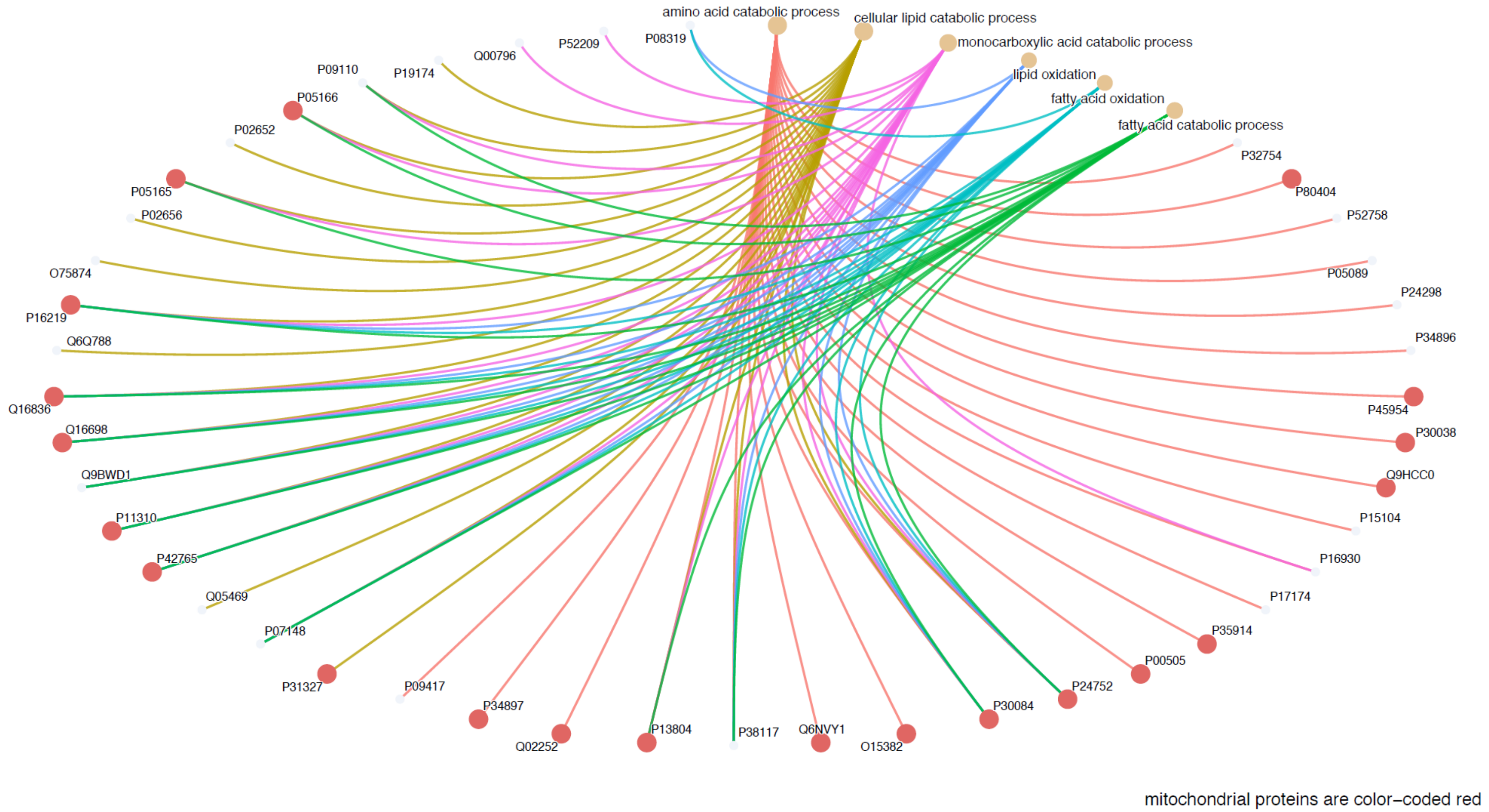

Supplemental Figure 5 – Gene Set Enrichment Analysis (GSEA) based on differentially expressed tissue proteins from human plaque affected arteries between TET2 CHIP mutation carriers and matched controls. Provided are enriched terms in which more than 50% of the leading proteins consist of mitochondrial proteins. Differential expression based on CHIP status is associated with relevant metabolic traits.

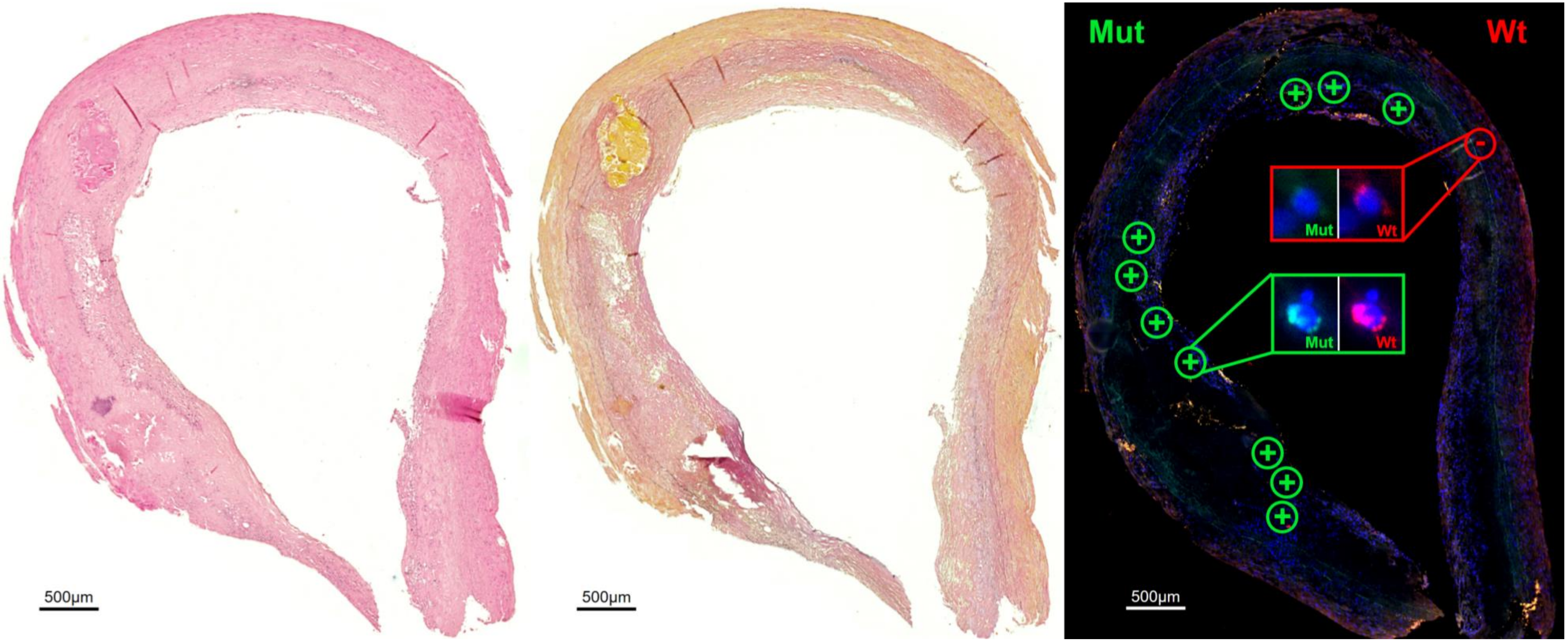

Supplemental Figure 6 – Proximal LAD affected by atherosclerosis of a CHIP mutation carrier. Images derive from immediate neigbor sections. **Left panel** - staining with H&E. **Middle panel** - staining with EvG. **Right panel** - mutaFISH^{TM} to detect a specific DNMT3A CHIP mutation (c.2245C>T). Staining was performed in situ at RNA level. Green circles indicate locations of identified CHIP positive cells. As an example, the red circle highlights a CHIP negative leukocyte. The DNMTA3 mutation c.2245C>T was detected via the green signal (Mut), the DNMT3A wild type via the red (Wt) signal and the cell nuclei (DAPI) via the blue signal. DAPI: 4′.6-Diamidin-2-phenylindole. mutaFISH: mutation-specific Fluorescence In Situ Hybridization.

Adapted mutaFISH^{TM} protocol

Used Kits

- o mutaFISH RNA Probes KIT (Abnova Corporation. Taiwan)
- o mutaFISH^{TM} RNA Accessory KIT (KA4915. Abnova Corporation. Taiwan)

Preparation of RNAse free buffers

All buffers for the mutaFISH protocol have to be nuclease free.

- o **Poly-L-Lysine 1:10:** Prepare 1:10 Poly-L-Lysine in ddH2O.
- o **DEPC H2O:** Use 1 ml DEPC for 1000 ml ddH2O incubate for 1h at RT and autoclave.
- o **DEPC PBS*:** Use 1 ml DEPC for 1000 ml PBS pH 7.4 incubate for 1h at RT and autoclave.
- o **PBST*:** Use autoclaved DEPC PBS and add 1 ml Tween20 after autoclaving. If you use ready-to-use PBST Use DEPC and filter after 1h of incubation at RT.
- o **Permeable Protease buffer:** Use 3mg/ml Pepsin to 0.5 M HCL.
- o **Nuclease Free 1x Citric acid buffer pH6:** dilute nuclease free 10x ready-to-use Citric acid buffer in DEPC H2O or prepare 1x nuclease free buffer pH6.
- o **2x SSC Buffer**:** Dilute ready-to-use nuclease free 20x SSC buffer in DEPC H2O or prepare 2x nuclease free buffer.
- o **70% and 85% EtOH:** Dilute EtOH absolute to 70% and 85%.
- o **3-4% Paraformaldehyde:** Paraformaldehyde solution has to prepared methanol free. Dilute in DEPC-PBS

* here Roti®fair PBST 7.4 and Roti® PBS 7.4 (CarlRoth GmbH&CoKG. Karlsruhe) were used

** here Roti®-Stock 20x SSC (CarlRoth GmbH&CoKG. Karlsruhe) was used

Protocol – Coating with Poly-L-Lysine

1. Let Poly-L-Lysine (1:10 in ddH2O) come to room temperature.
2. Incubate Slides 7min at RT in **1:10 Poly-L-Lysine** for coating.
3. Remove slides from the rack and tap off water droplets.
4. Incubate slides at 56 °C for at least 1h.

Protocol – mutaFISH

Tissue preparation

1. Prepare 3-5 µm thick FFPE sections. air dry sections at heating plate (40 °C).
2. Incubate FFPE sections for 1h at 56 °C.

De-paraffinization and rehydration

1. Rinse slides 2 times in xylene substitute for each 5 min.
2. Immerse slides 2 times in 100% EtOH for each 3 min.
3. Immerse slides 2 times in 85% EtOH for each 3 min.
4. Immerse slides 2 times in 70% EtOH for each 3 min.
5. Wash slides in DEPC-H2O for 1 min and dry shortly at RT.

Target retrieval

1. Preheat heating plate to 75 °C.
2. Create secure bond with wax pen around the tissue sections.
3. Wash slides in DEPC-PBS for 2 min
4. Incubate slides with 1x citric buffer at 75-85 °C on heating plate for 20 min.
   CAVE: Renew citric buffer every 10-15 min – sample should not dry out.

5. Wash twice with 2x SSC buffer for 5 min.

CAVE: If costaining with an antibody should be done perform permeabilization and immunostaining prior to mutaFISH and end up with 20 min fixation in 4% formaldehyde at RT.

Fixation and permeabilization

1. Incubate in 3-4% paraformaldehyde (provided) for 20 min at RT.
2. Immerse slides in 2x SSC buffer for 5 min.
3. Pre-warm permeable buffer (provided in the kit or self-made) to 37 °C.
4. Use permeable buffer or 3 mg/mL Pepsin to 0.1 M HCl) at 37 °C for 30 min.
   TIPP: RNAscope® ***** *Protease III & Protease IV[3] reagents can be used. Here no pre-warming is necessary.*
5. Wash slides in 2x SSC buffer for 5 min.

***** RNAscope® Protease IV (Advanced Cell Diagnostics. Inc.. Canada) was used in this case

Dehydration

1. Immerse slides in 70% EtOH for 1 min.
2. Immerse slides in 85% EtOH for 1 min.
3. Immerse slides in 100% EtOH for 1 min.
4. Immerse slides in fresh PBST for 1 min.

In situ reverse transcription

1. prepare the following mixture on ice and use 100 µl of the mixture per slide.

**Component**	**Amount per slide [µL]**
DEPC-H2O (Kit)	60.5
5x RT Buffer (Kit)	20.0
BSA (Kit)	1.0
dNTP Mix (Kit)	5.0
**RT Primer (individual)**	1.0
RNase Inhibitor (Kit)	2.5
RT Enzyme (Kit)	10.0
**Total volume:**	**100.0**

2. Incubate Slides at 37 °C in humidity oven over night.
   CAVE: Take care that there is enough humidity. Slides should not dry out.
3. Wash shortly in PBST.
4. Immerse slides with fresh PBST 2 times for each 2 min.

Post-fixation and probe hybridization

1. Cover tissue with 3-4% paraformaldehyde (provided) and incubate at 37 °C in humidity Box for 45 min.
   CAVE: Check formaldehyde every 10-15 min – sample should not dry out.
2. Wash slides with fresh PBST 2 times for 2 min.
3. prepare the following mixture on ice and use 100µl per slide.

**Component**	**Amount per slide [µL]**	**Amount for negative control [µl]**
DEPC-H2O (Kit)	32.5	34.5
Formamide (Kit)	20.0	20.0
10x Hybrid Enzyme Buffer (Kit)	10.0	10.0
1 M KCl (Kit)	5.0	5.0

**mutaFISH probe wt (individual)**	1.0	
**mutaFISH probe mutation (individual)**	1.0	
RNase Inhibitor (Kit)	2.5	2.5
RNaseH (Kit)	8.0	8.0
Hybrid Enzyme (Kit)	20.0	20.0
**Total volume:**	**100.0**	**200.0**

4. Incubate at 37 °C in humidity oven for 60 min.
5. Heat up to 45 °C and incubate slides for another 90 min.
6. Immerse slide with fresh PBST 2 times for 2 min.

Amplification

1. Prepare the following mixture on ice and use 100 µl per slide.

Component	Amount per slide [µL]
DEPC-H2O (Kit)	61.5
50% Glycerol (Kit)	10.0
10x DNA Polymerase Buffer (Kit)	10.0
BSA (Kit)	1.0
dNTP mix (Kit)	5.0
RNase Inhibitor (Kit)	2.5
DNA Polymerase (Kit)	10.0
**Total volume:**	**100.0**

2. Incubate slides at 37 °C in humidity oven for 120 min.
3. Wash shortly in PBST.
4. Immerse slide with fresh PBST two times for 1 min.

Detection and counterstain

1. Use 100 µl of the following mixture per slide (prepare on ice).

Component	Amount per slide [µL]
Detection Buffer (Kit)	98.0
**Detection probe for wt (individual)**	1.0
**Detection probe for mutation (individual)**	1.0
**Total volume:**	**100.0**

2. Incubate at 37 °C in humidity oven for 60 min.
3. Wash shortly in PBST.
4. Immerse slide with fresh PBST 2 times for 2 min.
5. Immerse the slide in 70% EtOH for 0.5 min.
6. Immerse the slide in 85% EtOH for 0.5 min.
7. Immerse the slide in 100% EtOH for 0.5 min.
8. Mix 4 µl DAPI with 664 µl DEPC-PBS and apply 100 µl to the sample for 2-3 min at RT.

Sealing

- Immerse slide two times in fresh DEPC-PBS for 1 min.
- Cover slide with Prolong-Gold-Anti-Fade let it dry for 15 min and seal with nail-polish.
- Let dry slides for 1h and proceed with microscopy.
